# Reverse vaccinology-based design of multivalent multiepitope mRNA vaccines targeting key viral proteins of Herpes Simplex Virus type-2

**DOI:** 10.3389/fimmu.2025.1586271

**Published:** 2025-05-20

**Authors:** N. S. Suneesh, Kishore Dhotre, Pratik Mahajan, Debashree Dass, Anwesha Banerjee, Nikhat J. Siddiqi, Abdul Malik, Manali Joshi, Abdul Arif Khan, Vijay Nema, Anupam Mukherjee

**Affiliations:** ^1^ ICMR-National Institute of Translational Virology and AIDS Research, Pune, India; ^2^ AcSIR - Academy of Scientific and Innovative Research, Ghaziabad, India; ^3^ Friedrich-Alexander-Universität Erlangen-Nürnberg (FAU), Erlangen, Germany; ^4^ King Saud University, Riyadh, Saudi Arabia; ^5^ Savitribai Phule Pune University, Pune, India; ^6^ ICMR-National Institute of Research in Tribal Health, Jabalpur, India; ^7^ ICMR-National Institute of Virology, Pune, India

**Keywords:** HSV-2, mRNA vaccine, reverse vaccinology, multiepitope vaccine, immunoinformatics, vaccine design

## Abstract

**Introduction:**

Herpes Simplex Virus type 2 or HSV-2 is a major cause of genital herpes, contributing to increased susceptibility to HIV, encephalitis, and other severe complications. Despite the availability of antiviral therapies such as acyclovir, their effectiveness is limited due to resistance and side effects, emphasizing the urgent need for an effective vaccine.

**Methods:**

This study employed reverse vaccinology and immunoinformatics to design five multivalent, multiepitope mRNA vaccine constructs targeting HSV-2. Four key viral proteins—Glycoprotein B (gB), Ribonucleoside-diphosphate Reductase large subunit (RIR1), Infected Cell Protein 0 (ICP0), and VP23—were selected based on their roles in viral replication and immune evasion. Epitopes for Cytotoxic T Lymphocytes (CD8^+^), Helper T Lymphocytes (CD4^+^), and B cells were predicted and rigorously filtered for antigenicity, non-toxicity, and cytokine induction. Vaccine constructs were designed incorporating 50S ribosomal protein, Human β-defensin 3, and PADRE as adjuvants to enhance immune responses. Structural validation, molecular docking, codon optimization, and physiochemical analysis were performed to assess stability and immunogenic potential.

**Results:**

The vaccine constructs demonstrated favorable physiochemical properties, structural stability, and high antigenicity. Molecular docking revealed strong binding affinities between the predicted epitopes and their respective MHC class I and class II alleles. Proteasomal cleavage analysis confirmed efficient antigen processing, while codon optimization ensured compatibility with the human translational machinery. Computational immune simulations predicted a strong humoral and cellular immune response, including high IgG and IgM levels, robust CD4^+^ and CD8^+^ T-cell activation, and cytokine production.

**Conclusion:**

The rationally designed multiepitope mRNA vaccine constructs exhibit strong antigenic potential, structural stability, and immune-stimulatory properties, positioning them as promising candidates for HSV-2 vaccine development. These findings offer a novel, safe, and effective approach to HSV-2 immunization, warranting further experimental validation and preclinical studies.

## Graphical Abstract

Schematic representation of the *in silico* workflow used for designing multivalent, multiepitope mRNA vaccines (MMEV) against HSV -2.

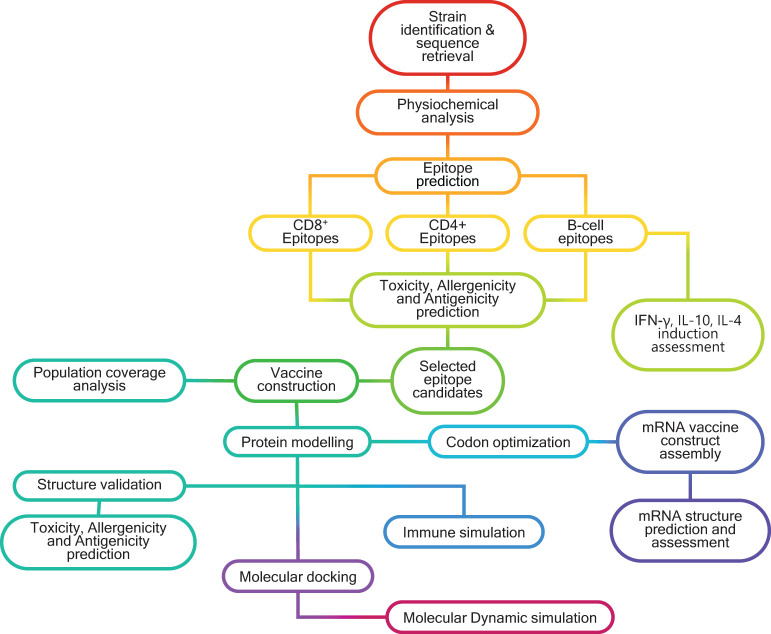

## 1 Introduction

Herpes Simplex Virus type 2 (HSV-2), also known as Human alphaherpesvirus 2 (HHV-2), is a double-stranded DNA virus belonging to the family *Orthoherpesviridae* (1). It is a leading cause of sexually transmitted infections, including genital herpes, herpes stromal keratitis, encephalitis, and meningitis. Beyond its direct clinical impact, HSV-2 poses significant public health challenges by tripling the risk of HIV acquisition and conferring resistance to antiretroviral therapy recommended by the World Health Organization (WHO) (2). Moreover, HSV-2 infection increases transmissibility to sexual partners and neonates, amplifying its societal burden (3). According to WHO estimates, approximately 491.5 million individuals aged 15 to 49 were living with HSV-2 globally in 2016, reflecting the widespread prevalence and enduring threat of this virus (4, 5).

The current treatment landscape relies heavily on antiviral agents such as acyclovir and its prodrug, valacyclovir, which are deoxynucleoside analogs effective against HSV-2 in immunocompetent individuals. However, the emergence of acyclovir-resistant HSV strains, particularly in immunocompromised populations like those living with HIV, undermines the efficacy of these treatments (6, 7). Furthermore, these antivirals are associated with adverse side effects, including nausea, appetite loss, and neutropenia, and they fail to prevent symptomatic outbreaks or asymptomatic viral shedding, leading to frequent recurrences (8, 9). The extensive and prolonged use of these drugs has further diminished their effectiveness, highlighting the critical need for alternative strategies, particularly an effective vaccine against HSV-2 (10).

Various vaccine development strategies have been explored for HSV-2, including live-attenuated, subunit, nucleic acid-based, and replication-defective virus vaccines; however, none have yet been licensed for clinical use. While many of these approaches have shown potential in eliciting immune responses, they face challenges in achieving durable immunity, complete protection from viral shedding, and latency reactivation (11). The advent of mRNA vaccine technology has revolutionized vaccinology, offering a platform for rapid and safe vaccine development. Since its initial exploration in 1987 with cationic liposome-based mRNA transfection (12), mRNA vaccines have demonstrated remarkable efficacy against several pathogens, notably with the success of COVID-19 vaccines.

Among the most promising approaches is the design of multiepitope-based mRNA vaccines, which target multiple antigenic sites to elicit broad and robust immune responses (13). This strategy is particularly advantageous for pathogens like HSV-2, which exhibit high genetic variability and intricate immune evasion mechanisms (14–16). Compared to single-antigen vaccines, multiepitope vaccines address the challenges posed by genetic diversity and are safer and more adaptable. Immunoinformatics has played a pivotal role in advancing multiepitope vaccine design, enabling the precise identification and optimization of immunogenic epitopes (17).

In this study, we utilized reverse vaccinology and immunoinformatics to develop multivalent multiepitope mRNA vaccine candidates targeting HSV-2. Four viral proteins - Glycoprotein B (gB), Ribonucleoside-diphosphate Reductase large subunit (RIR1), Infected Cell Protein 0 (ICP0), and capsid protein VP23 - were selected for their critical roles in viral replication, immune evasion, and pathogenesis. Vaccine constructs were designed to incorporate epitopes from three globally relevant HSV-2 strains: HG52, SD90e, and S333. The outcomes of this study present promising vaccine candidates that hold the potential to address the persistent global burden of HSV-2.

## 2 Materials and methods

### 2.1 Sequence retrieval and consensus sequence generation

Sequences of the HSV-2 viral proteins - gB, RIR1, ICP0, and VP23 - were obtained from the NCBI Protein database (https://www.ncbi.nlm.nih.gov/protein/) and UniProt database (https://www.uniprot.org/) using the accession IDs (**Supplementary Table 1**). These sequences were retrieved for three geographically diverse HSV-2 strains: HG52 (UK), SD90e (South Africa), and S333 (USA). The consensus sequences for each protein were generated using the EMBOSS Con tool (https://www.ebi.ac.uk/jdispatcher/msa/emboss_cons?stype=protein), and these consensus sequences were subsequently utilized for downstream analyses. The overall workflow for designing a multivalent multiepitope vaccine against HSV-2 is illustrated in the Graphical Abstract.

### 2.2 Prediction and filtration of CD8^+^ cytotoxic T-lymphocyte epitopes

CTL epitopes were predicted using the Immune Epitope Database and Analysis Resource (IEDB; https://www.iedb.org/). The MHC-I binding tool (http://tools.iedb.org/mhci/) was employed to predict epitopes, using the NetMHCpan-4.1 EL and NetMHCpan-4.1 BA server, which applies Artificial Neural Networks (ANNs) trained on experimental data. The protein sequences, formatted in FASTA, were analyzed with the human MHC source species, and the full HLA reference set was used with a 9-mer peptide length. The output included nine variables: allele, sequence length, core peptide, start and end positions, IC50 value, percentile rank, and binding score. This data was saved for further analysis.

### 2.3 Prediction and filtration of CD4^+^ helper T-lymphocyte epitopes

Prediction of MHC class II binding epitopes was performed similarly to the MHC class I analysis. The MHC-II binding tool (http://tools.iedb.org/mhcii/) was used with the NetMHCIIpan-4.1 server as the prediction method. The full HLA allele reference list was selected, and the peptide length was set to 15-mer. The resulting dataset included predicted peptides, their binding HLA alleles, IC50 values, and percentile ranks, analogous to the MHC-I binding predictions.

### 2.4 Prediction and filtration of B-cell epitopes

Linear B-cell epitopes were predicted using the ABCpred server (http://crdd.osdd.net/raghava/abcpred/). ABCpred utilizes a combination of Recurrent Neural Networks and Feed-Forward Neural Networks to predict linear B-cell epitopes (18). The threshold was set to 0.51, with all other parameters kept at default settings.

### 2.5 Filtering the predicted epitopes

The datasets generated from IEDB were imported into R Studio, where thresholds were applied to identify the most promising epitopes. For the Eluted Ligands (EL) datasets of both MHC-I and MHC-II binding predictions, peptides with a percentile rank of ≤0.5% for MHC-I and ≤2% for MHC-II were selected as strong binders (SBs). For the Binding Affinity (BA) datasets, epitopes were further filtered based on allele-specific IC50 cutoff values, with 500 nM set as the threshold for MHC-II alleles. Subsequently, the filtered EL and BA datasets were merged, identifying common peptides suitable for vaccine development (19).

### 2.6 Validation of epitopes for antigenicity, allergenicity, toxicity and cytokine production

All filtered epitopes were first evaluated for antigenicity using the VaxiJen v2.0 server (https://www.ddg-pharmfac.net/vaxijen/VaxiJen/VaxiJen.html), which employs an alignment-independent method to predict antigenicity based on the physiochemical properties of peptide sequences (20). The epitopes were batch-submitted in FASTA format with the target organism set to “virus” and a threshold value of 0.4. Subsequently, allergenicity prediction was performed using AllerTop v2.0 (https://www.ddg-pharmfac.net/AllerTOP/), which applies the *k*-nearest neighbor (*k*NN) algorithm for prediction with 85.3% accuracy and five-fold cross-validation (21).

To determine whether the sequences were toxic, the ToxinPred server (https://webs.iiitd.edu.in/raghava/toxinpred/multi_submit.php) was utilized. Peptides were submitted in FASTA format using the Support Vector Machine (SVM)-based prediction method with default parameters. Additionally, the ability of HTL epitopes to induce IFN-γ production was assessed using the IFNepitope server (http://crdd.osdd.net/raghava/ifnepitope/predict.php), with sequences submitted in FASTA format and SVM-based prediction applied. IL4Pred (https://webs.iiitd.edu.in/raghava/il4pred/multiple_test.php) and IL10Pred (https://webs.iiitd.edu.in/raghava/il10pred/predict3.php) were also employed to evaluate the potential of sequences to induce IL-4 and IL-10 production, respectively. Only epitopes deemed antigenic, non-allergenic, non-toxic, and capable of inducing cytokine production were selected as candidates for vaccine construction.

### 2.7 Assembly of vaccine construct

**Figure 1:**
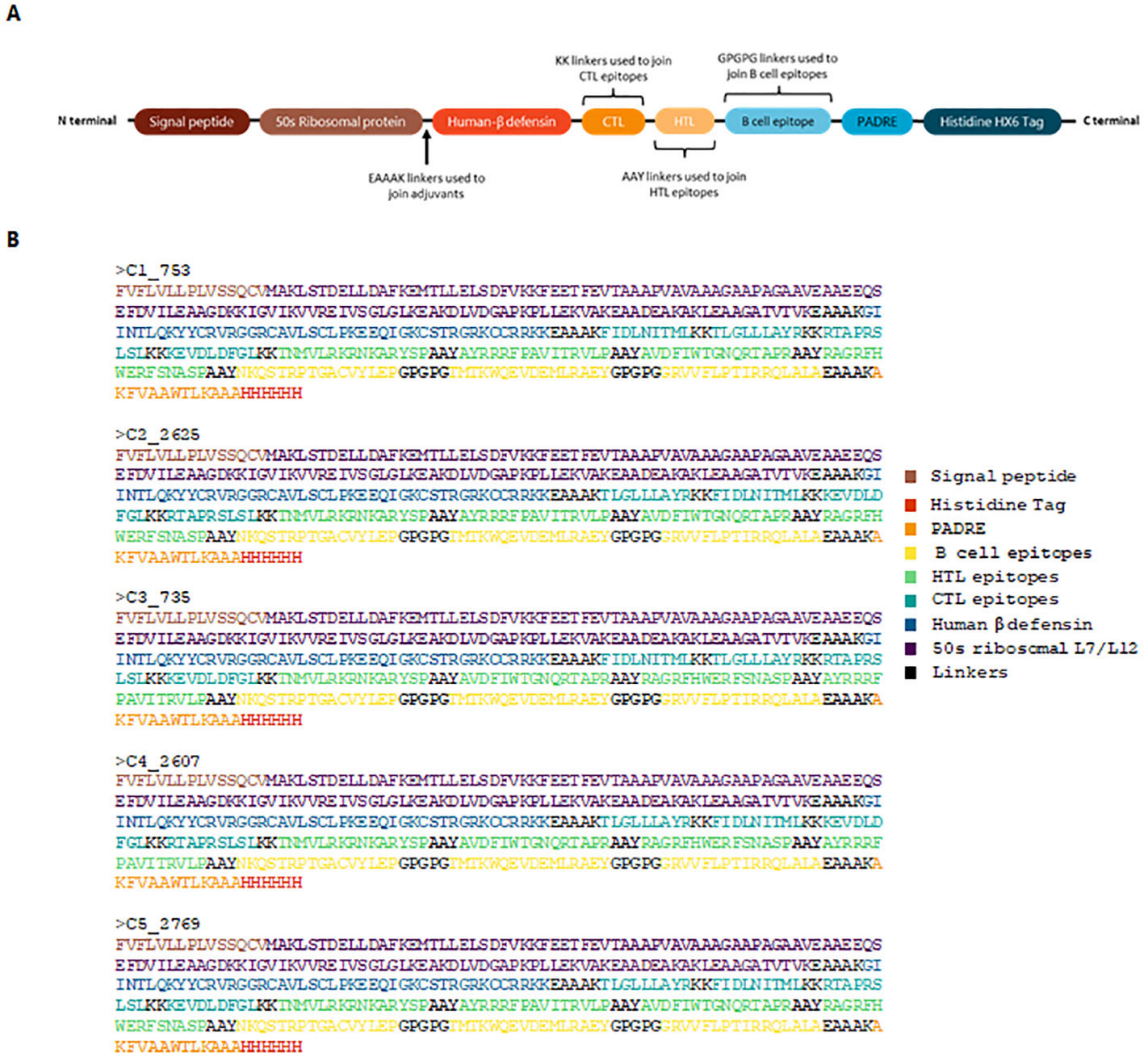
Schematic representation and sequence of the designed vaccine construct. **(A)** Structural assembly illustrating the arrangement of adjuvants, linkers, and epitopes. The 50S ribosomal protein and Human β-defensin 3 are positioned at the N-terminal, linked by EAAAK, while the PADRE sequence is incorporated at the C-terminal. CTL, HTL, and B-cell epitopes are arranged sequentially. A His-tag is included for purification. **(B)** Final protein sequences of the top five vaccine constructs.

Epitopes that satisfied the criteria for antigenicity, non-allergenicity, non-toxicity, and cytokine induction were used to assemble vaccine constructs using PyCharm. The code for vaccine assembly is publicly available on GitHub (https://github.com/Cerberus35/VaxBuilder/tree/main). Three adjuvants were incorporated: the 50S ribosomal protein from *Mycobacterium tuberculosis* (UniProt ID: G8FRW4), Human β-defensin 3 (GIINTLQKYYCRVRGGRCAVLSCLPKEEQIGKCSTRGRKCCRRKK), and the PADRE sequence (AKFVAAWTLKAAA). The first two adjuvants were positioned at the N-terminal and linked using an EAAAK linker, while the PADRE sequence was added to the C-terminal, also linked via an EAAAK linker. The constructs were finalized with a His-tag for purification purposes. The epitopes were arranged from N-terminal to C-terminal in the following order: CTL, HTL, and B-cell epitopes. The arrangement of the vaccine constructs is illustrated in **Figure 1**.

### 2.8 Analysis of vaccine antigenicity, allergenicity, toxicity and other physiological properties

The antigenicity of the vaccine constructs was assessed using VaxiJen v2.0, allergenicity was evaluated with AllerTop v2.0, and toxicity was predicted using ToxinPred, as described earlier. The sequences, in plain format, were then analyzed for physiochemical properties using the ExpasyProtParam tool (https://web.expasy.org/protparam/). Solubility predictions were conducted using the SOLpro server within the SCRATCH protein prediction suite (https://scratch.proteomics.ics.uci.edu/). SOLpro is a computational tool designed to estimate the likelihood of a protein being soluble upon overexpression in *Escherichia coli* solely based on its primary sequence. This tool employs a two-tier machine learning approach utilizing SVMs to provide solubility probabilities and binary classifications (soluble or insoluble) based solely on primary sequence data (22).

### 2.9 Discontinuous B-cell epitope prediction

Discontinuous B-cell epitopes, formed as a result of protein folding, are recognized when residues spatially converge due to tertiary structure formation. These epitopes were predicted using the ElliPro server (http://tools.iedb.org/ellipro/), which integrates Thornton’s method with MODELLER for residue clustering and Jmol for visualization of epitope data (23). The predictions provided insights into the immunogenic regions of the folded vaccine constructs.

### 2.10 Analysis of proteasomal cleavage, surface accessibility and glycosylation sites

Proteasomal cleavage sites in the vaccine constructs were predicted using the NetChop 3.1 server (https://services.healthtech.dtu.dk/services/NetChop-3.1/), which applies a neural network-based method to identify cleavage sites critical for the generation of CTL epitopes (24). To assess the surface accessibility of amino acids in the vaccine constructs, the NetSurfP 3.0 web server (https://services.healthtech.dtu.dk/services/NetSurfP-3.0/) was employed. This tool utilizes a pre-trained ESM-1b language model for sequence encoding, offering high-speed and accurate predictions of relative surface accessibility (RSA) and classification of residues as either buried or exposed (25). NetNGlyc 1.0 Server was used to predict N-linked glycosylation sites in the vaccine constructs (https://services.healthtech.dtu.dk/services/NetNGlyc-1.0/). This tool identifies Asn-Xaa-Ser/Thr motifs (sequons) and evaluates their likelihood of glycosylation using an artificial neural network (26), the constructs were submitted in FASTA format, and predictions were run with the default threshold of 0.5. These properties were calculated to better understand the structural characteristics and immunogenic potential of the vaccine constructs.

### 2.11 Structure prediction of the assembled vaccine construct

The secondary structure of the constructed protein was predicted using PSIPRED (http://bioinf.cs.ucl.ac.uk/psipred/) and SOPMA (https://npsa-prabi.ibcp.fr/cgi-bin/npsa_automat.pl?page=/NPSA/npsa_sopma.html). For *ab initio* structure prediction, the sequence was submitted to the Robetta server (https://robetta.bakerlab.org/), which employs deep learning methods such as trRosetta and RoseTTAFold for structure prediction (27, 28). The Robetta pipeline used the *ab initio* method, which does not rely on homology modeling but instead predicts structures based on the physical properties of atoms. Simultaneously, the AlphaFold server (https://alphafoldserver.com/) was employed to predict the structure of the designed vaccine candidates. The models generated by Robetta and AlphaFold were compared and analyzed using ChimeraX 1.8 matchmaker, and the model with the lowest Root Mean Square Deviation (RMSD) value was selected for further validation.

### 2.12 Validation of vaccine structure

The structural validation of the predicted protein models was performed using Ramachandran plots generated by the MOLprobity server (http://molprobity.biochem.duke.edu/). Additionally, the ProSA-web server (https://prosa.services.came.sbg.ac.at/prosa.php) was employed to evaluate the model’s energy profile in comparison with experimentally validated structures from the Protein Data Bank (PDB). These steps ensured that the predicted protein structures were biologically and structurally accurate.

### 2.13 Codon optimization of the protein construct and mRNA construct assembly

The protein sequence of the vaccine was reverse-translated and reverse-transcribed into its corresponding DNA sequence followed by codon optimization using the GenScript codon adaptation tool (https://www.genscript.com/tools/gensmart-codon-optimization), with the target organism set to human. This step optimized the codons to match the host organism’s codon usage preference. The resulting DNA sequence was further analyzed using GenRCA (https://www.genscript.com/tools/rare-codon-analysis) to calculate the Codon Adaptation Index (CAI), tRNA Adaptation Index (tAI), and Effective Number of Codons (ENC).

Following codon adaptation, the DNA sequence was transcribed into RNA using the Biomodel tool (https://biomodel.uah.es/en/lab/cybertory/analysis/trans.htm). Manual assembly of the mRNA vaccine construct included the addition of a 5′ cap 1 structure, also known as trinucleotide cap1 (5′-cap (m7(3′OMeG)(5′)ppp(5′)(2′OMeA)pG), to ensure ribosomal recruitment and prevent mRNA degradation (29). The 5′ untranslated region (UTR) was derived from the human β-globin (HBB) sequence, retrieved from the UCSC genome browser (ID: ENST00000335295.4), as it is known to enhance translation efficiency (30). A Kozak sequence (5′-GCCACC-3′) was incorporated to further optimize translation initiation (31).

Downstream of the 5′UTR, the start codon was placed, followed by the mRNA vaccine coding sequence. The 3′UTR region, retrieved from GitHub repository (https://github.com/NAalytics/Assemblies-of-putative-SARS-CoV2-spike-encoding-mRNA-sequences-for-vaccines-BNT-162b2-and-mRNA-1273), was included to mimic the design of the Moderna mRNA-1273 COVID-19 vaccine (32). Additionally, a segmented poly(A) tail was incorporated to enhance mRNA stability (33).

### 2.14 mRNA secondary structure analysis

The mRNA secondary structure was analyzed using the RNAFold server (http://rna.tbi.univie.ac.at/cgi-bin/RNAWebSuite/RNAfold.cgi), which predicts the minimum free energy (MFE, ΔG in kcal/mol) of RNA structures. Lower MFE values indicate greater stability of the mRNA structure (34). The mRNA-1273 Moderna vaccine and Pfizer-BNT 162b2, both of which have demonstrated efficacy, were used as positive controls to benchmark the MFE values of the designed mRNA constructs.

### 2.15 Molecular docking

Peptides were constructed using ChimeraX 1.8 and docked with their corresponding HLA class receptors. Molecular docking analyses for the predicted epitopes and their corresponding HLA alleles were performed using the ClusPro 2.0 server (https://cluspro.bu.edu), which utilizes rigid-body docking via PIPER (35–39). The PDB structures of the MHC class I and II HLA alleles – HLA-A* 02:06 (PDB ID: 3OXR), HLA-DRB1* 04:01 (PDB ID: 5NI9), HLA-B* 57:01 (PDB ID: 5VUF), and HLA-B* 44:03 (PDB ID: 3DX7) - were retrieved from the Protein Data Bank. Ligands (epitopes) were constructed and refined using UCSF ChimeraX 1.8 (https://www.cgl.ucsf.edu/chimerax/). Protein-protein interaction studies between the modeled vaccine constructs and TLR4/MD2 proteins were also conducted using the ClusPro 2.0 pipeline. The best-docked structures were further analyzed with ChimeraX 1.8.

### 2.16 Molecular dynamics simulation

Molecular dynamics (MD) simulations were performed for best docked pose for C5_2769-TLR4/MD2 complex and C4_2607-TLR2 complex after Molecular Docking using the GROMACS (GROningen Machine for Chemical Simulations) version 2021.3 simulation package (https://ftp.gromacs.org/gromacs/gromacs-2021.3.tar.gz) to evaluate the structural behavior and stability of the vaccine constructs in a biological context (40). Ligand parameters were derived from the CHARMM General Force Field server (https://cgenff.com/about/) (41). The systems were solvated in a cuboid box with an explicit TIP3P water model, maintaining a margin of 15 Å, and neutralized with Na^+^ and Cl^-^ counter-ions. Electrostatic and Van der Waals interactions were calculated with a long-range cutoff of 12 Å. Following solvation and neutralization, energy minimization was performed for 50,000 steps to relieve any steric clashes in the system. Equilibration was conducted at 300 K under the NVT ensemble for 100 ps, followed by pressure coupling in the NPT ensemble for 1000 ps. Triplicate 100 ns production simulations were carried out to capture the dynamics of the system. Root mean square deviations (RMSD), root mean square fluctuations (RMSF), and Radius of Gyration (Rg) were analyzed to understand the structural dynamics. Visualization and analysis of the simulation trajectories were conducted using VMD and GROMACS. This analysis provided insights into the dynamic properties and interactions of the vaccine construct and TLR complexes under simulated physiological conditions.

### 2.17 Immune simulation

Immune response simulations were conducted using the C-ImmSim server (http://c-immsim.iac.rm.cnr.it, alias http://kraken.iac.rm.cnr.it/C-IMMSIM) to assess the immunogenic potential of the vaccine constructs. Default parameters were employed, and the reaction volume was set to 10 μL. The simulation provided detailed predictions of humoral and cellular immune responses elicited by the vaccine constructs.

## 3 Results

### 3.1 Multiple sequence alignment, physiochemical analysis and consensus sequence generation for epitope prediction

**Figure 2:**
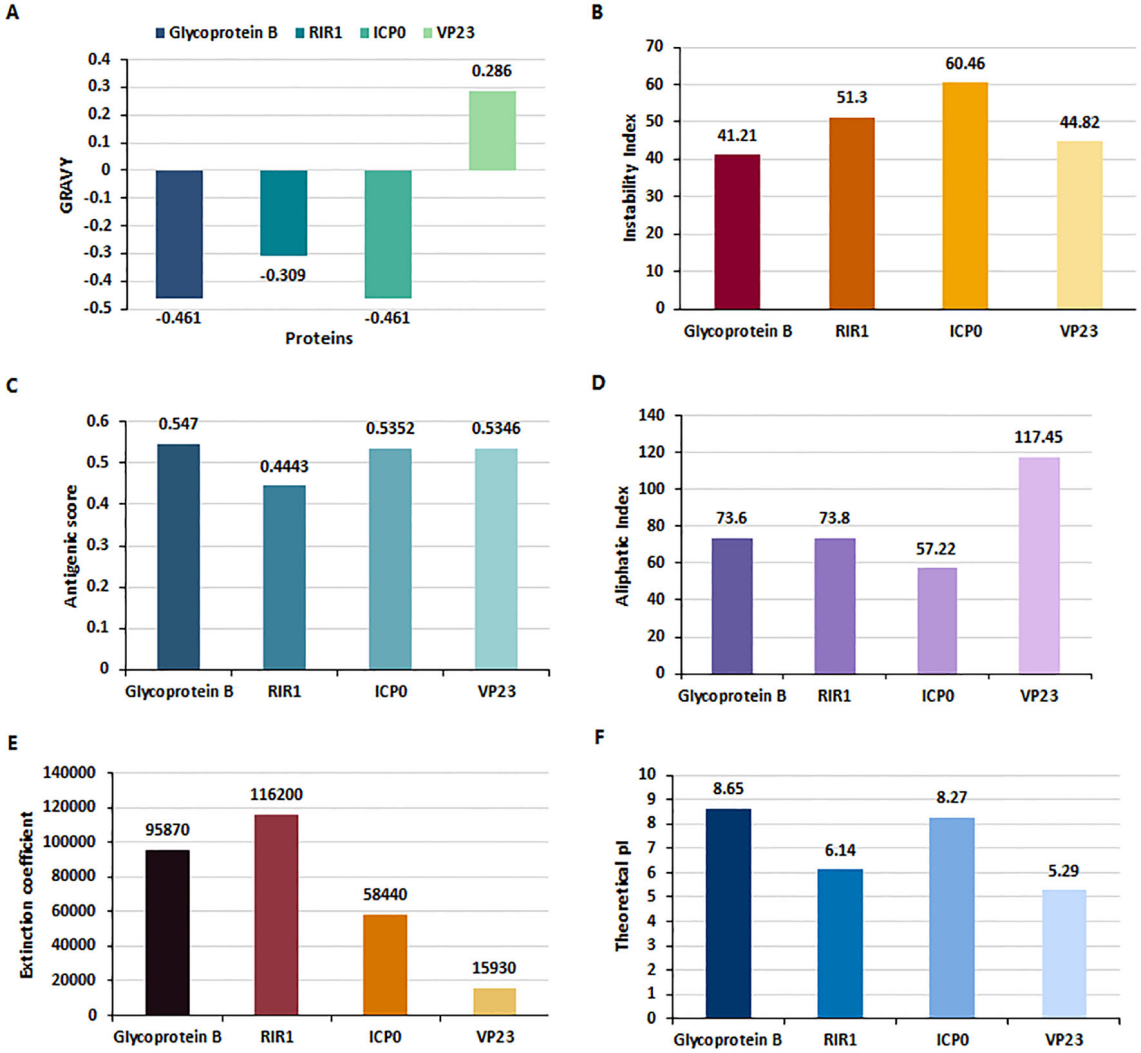
Physiochemical properties of HSV-2 proteins analyzed using ExpasyProtParam. **(A)** Grand Average of Hydropathy (GRAVY) index values indicate that Glycoprotein B (-0.461), RIR1 (-0.309), and ICP0 (-0.461) are hydrophilic, whereas VP23 (0.286) is hydrophobic. **(B)** Instability Index values suggest that RIR1 (51.3) and ICP0 (60.46) are unstable, while Glycoprotein B (41.21) and VP23 (44.82) exhibit borderline stability. **(C)** Antigenicity scores reveal strong immunogenic potential for Glycoprotein B (0.547), ICP0 (0.5352), and VP23 (0.5346), whereas RIR1 (0.4443) has a lower antigenicity score. **(D)** Aliphatic index values, indicative of thermostability, show VP23 as the most stable (117.45), followed by RIR1 (73.8), Glycoprotein B (73.6), and ICP0 (57.22). **(E)** Extinction coefficient values at 280 nm, reflecting protein concentration, are highest for RIR1 (116200), followed by Glycoprotein B (95870), ICP0 (58440), and VP23 (15930). **(F)** Theoretical isoelectric point (pI) values classify Glycoprotein B (8.65) and ICP0 (8.27) as basic proteins, while RIR1 (6.14) and VP23 (5.29) are acidic.

The Multiple Sequence Alignment (MSA) results revealed approximately 99% similarity among the sequences of the four proteins - gB, RIR1, ICP0, and VP23 - from the three HSV-2 strains (HG52, SD90e, and S333), as determined by the percentage identity matrix generated through Clustalω. The physiochemical properties of these four HSV proteins were analyzed using the ExpasyProtParam tool (**Figure 2**), and their molecular weights are presented in **Supplementary Table 1**. The Grand Average of Hydropathy (GRAVY) values indicated that Glycoprotein B (-0.461), RIR1 (-0.309), and ICP0 (-0.461) are hydrophilic, while VP23 (0.286) is hydrophobic (**Figure 2A**). The instability index values were 41.21 for Glycoprotein B, 51.3 for RIR1, 60.46 for ICP0, and 44.82 for VP23, suggesting that ICP0 and RIR1 are the least stable (**Figure 2B**). Antigenicity analysis revealed that Glycoprotein B had the highest score (0.547), followed closely by ICP0 (0.5352) and VP23 (0.5346), all indicating strong immunogenic potential. In contrast, RIR1 exhibited lower antigenicity with a score of 0.4443 (**Figure 2C**). The aliphatic index values, which indicate thermal stability, showed VP23 with the highest value (117.45), followed by RIR1 (73.8), Glycoprotein B (73.6), and ICP0 (57.22) (**Figure 2D**). The extinction coefficient values, which reflect protein concentration in solution, varied significantly: RIR1 (116200), Glycoprotein B (95870), ICP0 (58440), and VP23 (15930) (**Figure 2E**). The theoretical isoelectric point (pI) values suggested that Glycoprotein B (8.65) and ICP0 (8.27) are basic, whereas RIR1 (6.14) and VP23 (5.29) are acidic (**Figure 2F**). These findings provide critical insights into the stability, solubility, and immunogenic properties of these viral proteins, informing their potential as vaccine candidates.

### 3.2 Cytotoxic T lymphocyte epitope prediction and selection

**Figure 3:**
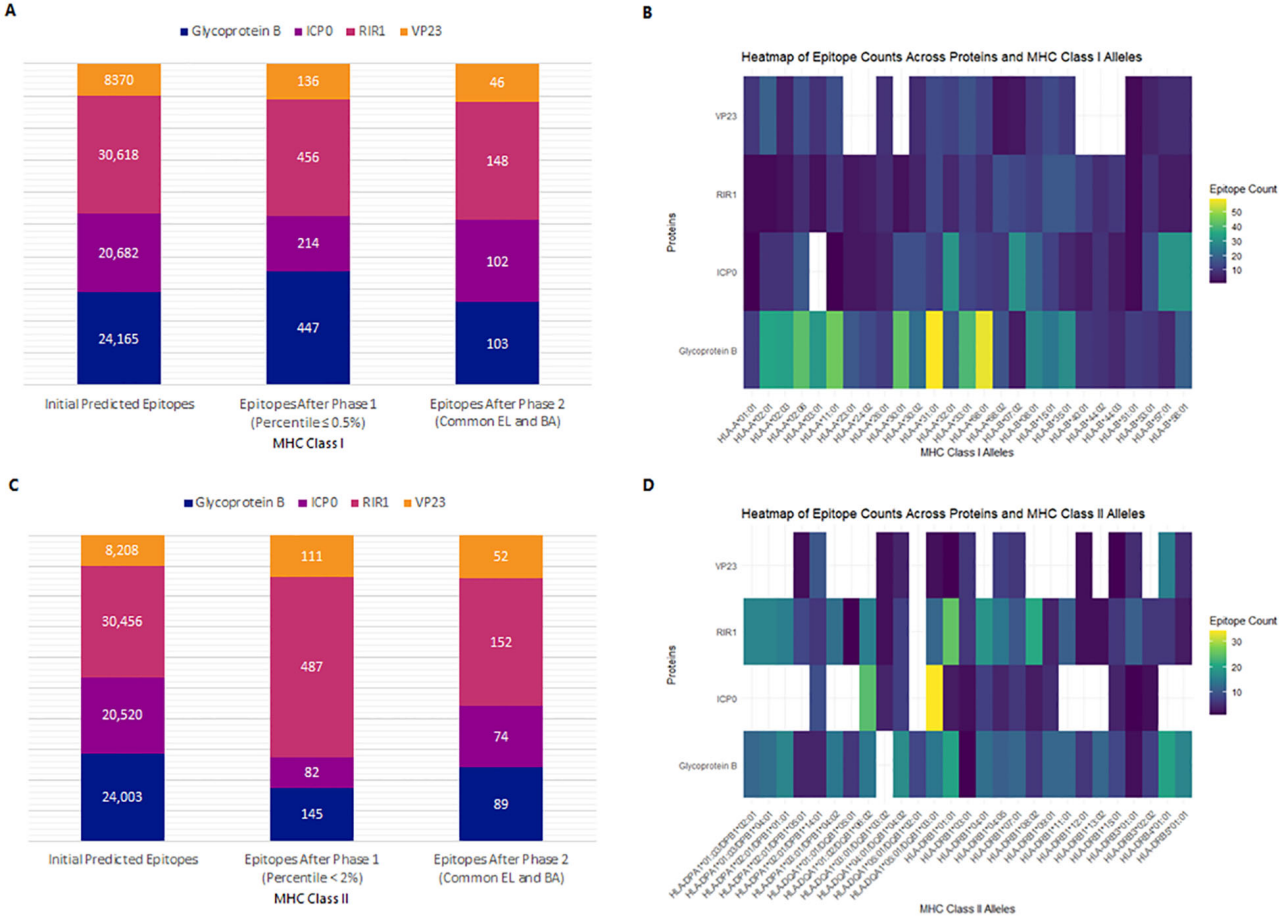
Prediction and selection of MHC-I and MHC-II epitopes for Glycoprotein B, ICP0, RIR1, and VP23. The final epitope set comprises strong binders common to both EL and BA prediction results. **(A)** Number of predicted MHC-I epitopes after sequential filtering. **(B)** Heatmap displaying the distribution of MHC-I epitopes across different alleles, highlighting immunodominance patterns for each viral protein. **(C)** Number of predicted MHC-II epitopes following the selection process. **(D)** Heatmap illustrating the distribution of MHC-II epitopes across various alleles, depicting relative binding preferences for each viral protein.

NetMHCpan 4.1 EL and NetMHCpan 4.1 BA predicted 24,165 epitopes for gB, 20,682 for ICP0, 30,618 for RIR1, and 8,370 for VP23 (**Figure 3A**). The datasets were processed in R Studio, where NetMHCpan 4.1 EL data was filtered to select epitopes with a percentile rank ≤ 0.5%, identifying strong MHC Class I binders. Similarly, NetMHCpan 4.1 BA data was refined using IC50 values specific to each MHC allele (19, 42). After merging the EL and BA datasets, common epitopes were grouped by their corresponding alleles and converted into FASTA format using PyCharm. The first filtering phase identified 447 epitopes for gB, 214 for ICP0, 456 for RIR1, and 136 for VP23. Further refinement confirmed 103, 102, 148, and 46 epitopes for gB, ICP0, RIR1, and VP23, respectively, as strong MHC Class I binders (**Figure 3A**). These epitopes were subsequently analyzed for antigenicity, allergenicity, and toxicity, yielding 55 antigenic epitopes for gB, 22 for ICP0, 33 for RIR1, and 16 for VP23 (**Supplementary Data 1**). For vaccine development, the top five antigenic epitopes from each protein were selected as final CTL candidates. A heatmap was generated to illustrate the distribution of epitope counts across different MHC Class I alleles for gB, ICP0, RIR1, and VP23, showcasing the immunodominance of specific proteins across various MHC alleles (**Figure 3B**).

### 3.3 Helper T lymphocyte epitope prediction and selection

Similarly, NetMHCIIpan 4.1 EL and NetMHCIIpan 4.1 BA predicted 24,003 epitopes for gB, 20,520 for ICP0, 30,456 for RIR1, and 8,208 for VP23 (**Figure 3C**). Data processing in R Studio applied a percentile rank <2% and IC50 values <500 nM as filtering thresholds to identify strong MHC Class II binders. After initial filtering, 145 epitopes were identified for gB, 82 for ICP0, 487 for RIR1, and 111 for VP23. Merging common epitopes from the EL and BA datasets resulted in 89, 74, 152, and 52 epitopes for gB, ICP0, RIR1, and VP23, respectively (**Figure 3C**). Further evaluations for antigenicity, allergenicity, toxicity, and cytokine induction (IFN-γ, IL-4, IL-10) narrowed the selection to 36 antigenic epitopes for gB, 28 for ICP0, 42 for RIR1, and 24 for VP23 (**Supplementary Data 1**). A heatmap illustrating the distribution of epitope counts across different MHC Class II alleles for gB, ICP0, RIR1, and VP23 highlights the immunodominance of specific proteins across various MHC alleles (**Figure 3D**).

### 3.4 Linear B-cell epitope prediction and selection

A total of 91 epitopes for gB, 87 for ICP0, 118 for RIR1, and 33 for VP23 were initially predicted (**Supplementary Data 1**). These epitopes were then analyzed for antigenicity, allergenicity, toxicity, and cytokine induction capabilities (IFN-γ, IL-4, IL-10). Only those that were non-toxic, non-allergenic, antigenic, and positive for all three cytokines induction were selected. This stringent filtering resulted in one B-cell epitope for gB, one for ICP0, 12 for RIR1, and two for VP23 (**Supplementary Data 1**).

### 3.5 Discontinuous B-cell epitope prediction

**Figure 4:**
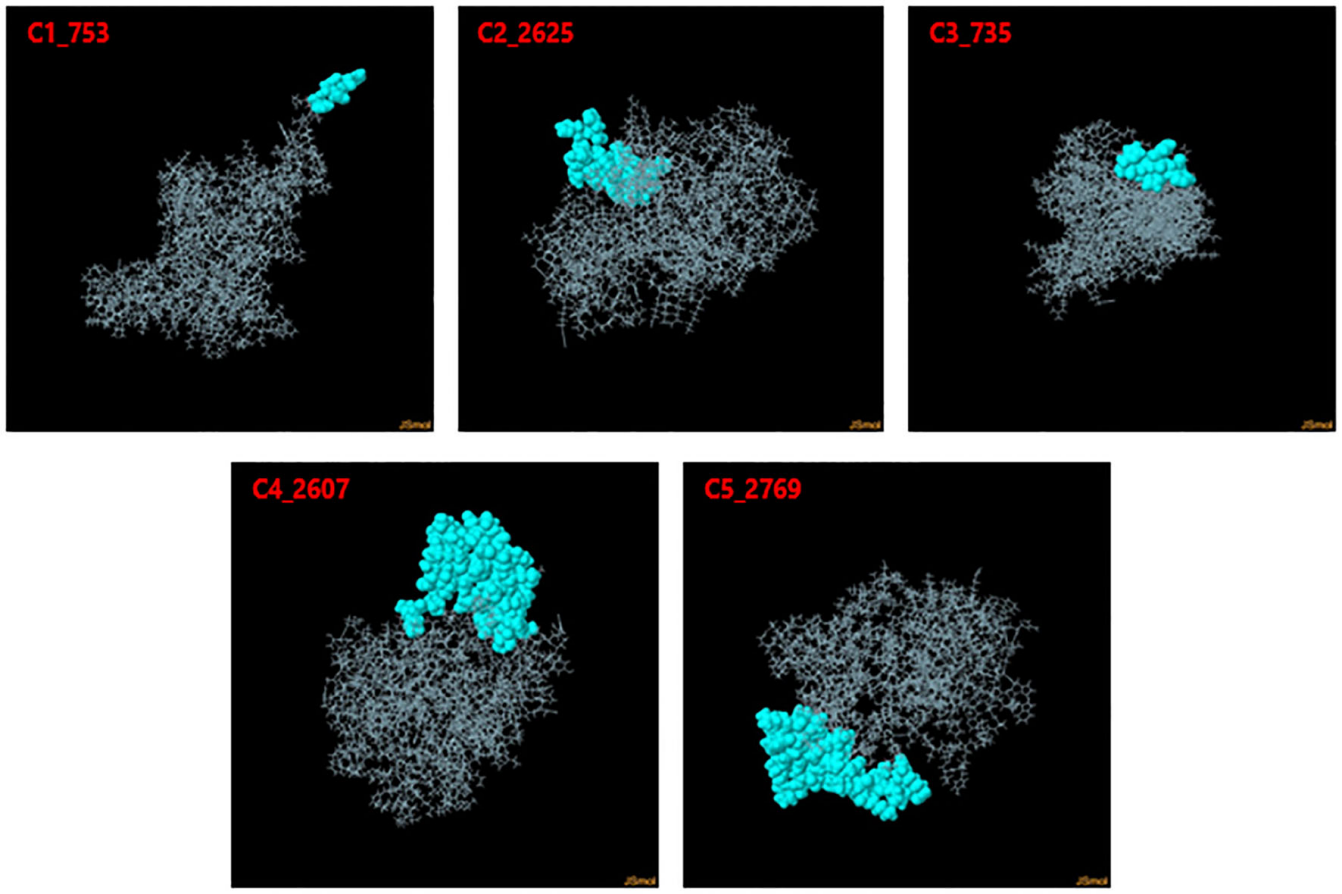
3D representation of top-scoring discontinuous B-cell epitopes in the five vaccine constructs. Predicted epitopes (cyan) were identified using ElliPro, based on structural protrusion and solvent accessibility. The remaining protein structure, representing the B-cell receptor (BCR), is shown in grey.

Discontinuous B-cell epitopes for the five vaccine constructs (C1_753, C2_2625, C3_735, C4_2607, and C5_2769) were predicted using the ElliPro server (**Figure 4**). This tool evaluates epitopes based on protein structure geometry and residue clustering, providing scores indicative of epitope protrusion and solvent accessibility (**Supplementary Data 2**). For C1_753, five epitopes were identified with scores ranging from 0.713 (largest cluster, 87 residues) to 0.739 (smallest cluster, 7 residues). C2_2625 exhibited six epitopes with scores between 0.718 (largest cluster, 57 residues) and 0.747 (smallest cluster, 3 residues). C3_735 had eight discontinuous epitopes, with scores from 0.722 (largest cluster, 54 residues) to 0.606 (smallest cluster, 3 residues). C4_2607 yielded six epitopes, ranging from 0.644 (largest cluster, 92 residues) to 0.587 (smallest cluster, 4 residues). C5_2769 produced seven epitopes with scores between 0.702 (smallest cluster, 5 residues) and 0.723 (largest cluster, 48 residues). **Table 1** highlights the top-scoring epitopes for each construct, emphasizing geometrically protruding and accessible regions. **Figure 4** presents the 3D representation of the top-scoring epitopes.

**Table 1:** Top-scoring discontinuous B-cell epitopes.

Vaccine Constructs	Top epitopes	Score	Cluster size (Residues)
C1_735	A:H395, A:H396, A:H397, A:H398	0.994	4
C2_753	A:A377, A:A378, A:K379, A:A380, A:K381, A:F382, A:V383, A:A384, A:A385, A:W386, A:T387, A:L388, A:K389, A:A390, A:A391, A:A392, A:H393, A:H394, A:H395, A:H396, A:H397, A:H398	0.891	22
C3_2607	A:H395, A:H396, A:H397, A:H398	0.981	4
C4_2625	A:P8, A:L9, A:V10, A:S11, A:S12, A:Q13, A:C14, A:V15, A:M16, A:A17, A:K18, A:L19, A:S20, A:T21, A:D22, A:E23, A:L24, A:L25, A:D26, A:A27, A:F28, A:K29, A:E30, A:M31, A:T32, A:L33, A:L34, A:E35, A:L36, A:S37, A:D38, A:F39, A:V40, A:F43, A:C183, A:S184, A:T185, A:R186, A:G187, A:R188, A:C190, A:C191, A:R192, A:R193, A:K194, A:K195, A:E196	0.776	47
C5_2769	A:K176, A:E177, A:E178, A:Q179, A:I180, A:G181, A:K182, A:C183, A:S184, A:T185, A:R186, A:G187, A:R188, A:P332, A:G333, A:P334, A:G335, A:P336, A:M348, A:L349, A:A351, A:E352, A:Y353, A:G354, A:P355, A:G356, A:P357, A:G358, A:G359, A:R360, A:V361, A:V362, A:F363, A:L364, A:P365, A:T366, A:I367, A:R368, A:Q370	0.759	39

### 3.6 Population coverage analysis

**Figure 5:**
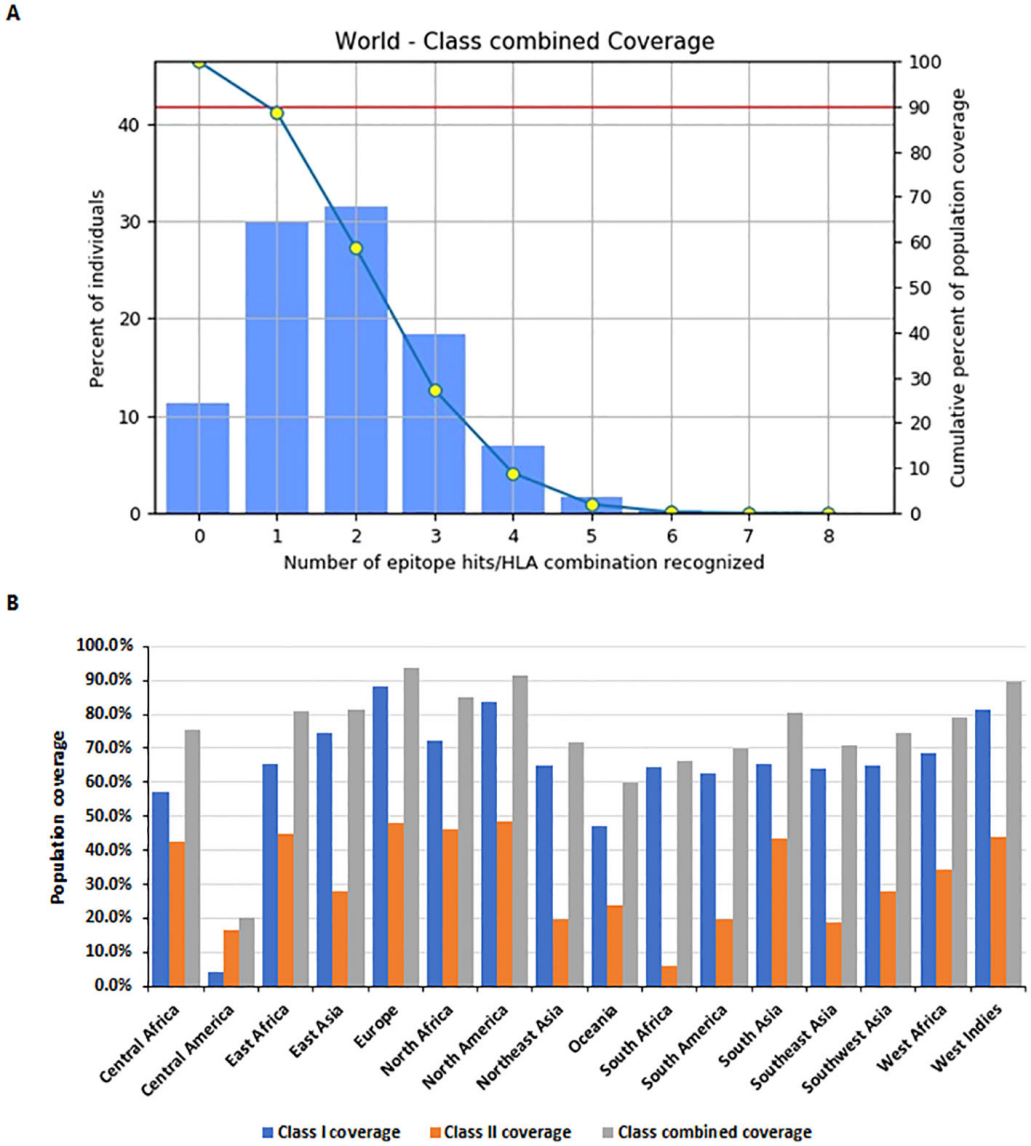
Population coverage analysis of selected MHC epitopes. **(A)** Combined population coverage for MHC Class I and Class II epitopes. **(B)** Regional population coverage distribution across different world populations.

Population coverage analysis was conducted using the IEDB population coverage tool (http://tools.iedb.org/population/) for the selected MHC Class I and Class II epitopes (**Figure 5**). The global combined coverage for MHC Class I and II epitopes was 88.64% (**Figure 5A**). Europe exhibited the highest coverage at 93.92%, with an average combined hit of 2.02, indicative of broad epitope representation. North America followed with 91.63% coverage, while Central America had the lowest combined coverage at 20.04%. For MHC Class I, the global coverage was 80.25%, with Europe achieving the highest coverage at 88.33%, followed by North America at 83.8%, and Central America showing the lowest at 4.14%. For MHC Class II, global coverage stood at 42.5%, with North America (48.03%) and Europe (47.93%) ranking the highest, whereas South Africa exhibited the lowest coverage at 5.91% (**Figure 5B**).

### 3.7 Construction and physiochemical analysis of vaccine

Vaccine constructs were assembled using PyCharm, employing various permutations of selected CTL (**Table 2**), HTL (**Table 3**), and linear B-cell epitopes (**Supplementary Data 1**), yielding 3,456 designs (Assembly schematic shown in **Figure 1A**). All constructs were evaluated for allergenicity, antigenicity, and toxicity, confirming that they were non-allergenic, antigenic, and non-toxic. The top five constructs (**Figure 1B**), selected based on antigenicity thresholds, underwent further physiochemical analysis using ExpasyProtParam. The molecular weight of all five constructs was 41.86 kDa, and the theoretical pI was calculated to be 9.78, classifying them as basic proteins. The GRAVY index of -0.259 indicated hydrophilic properties, suggesting high solubility. The instability index of 34.75 classified the vaccine constructs as stable proteins. These findings support the feasibility of the selected constructs for further evaluation and development (**Supplementary Data 3**).

**Table 2:** Top selected CTL epitopes selected for vaccine construction based on top antigenicity score.

Viral Protein	CTL epitopes (Start-end)	Interacting MHC-class I alleles from NetMHCIPan-4.1 EL	IEDB score	Interacting MHC-class I alleles from NetMHCIPan-4.1 BA	IC50 (Nm)	Antigenicity score	Allergenicity class	Toxicity class
gB	FIDLNITML (666-674)	HLA-A*02:06	0.760108	HLA-A*02:06	37.59	2.0483	Non-allergen	Non-toxic
		HLA-A*02:01	0.714389	HLA-A*02:01	63.76			
		HLA-A*02:03	0.360524	HLA-A*02:03	189.29			
		HLA-B*08:01	0.236097	-	-			
RIR1	KEVDLDFGL (454-462)	HLA-B*40:01	0.940433	HLA-B*40:01	14.3	3.0403	Non-allergen	Non-toxic
		HLA-B*44:03	0.390784	HLA-B*44:03	585.68			
		HLA-B*44:02	0.227882	-	-			
ICP0	RTAPRSLSL (217-225)	HLA-A*32:01	0.853665	HLA-A*30:01	27.84	1.3656	Non-allergen	Non-toxic
		HLA-A*30:01	0.793404	HLA-A*32:01	31.69			
		HLA-B*07:02	0.774316	HLA-B*07:02	32.09			
		HLA-B*57:01	0.684753	HLA-B*58:01	223.96			
		HLA-B*58:01	0.606154	HLA-B*08:01	385			
		HLA-B*08:01	0.491403	HLA-B*15:01	464.79			
		HLA-A*02:06	0.379616	HLA-B*57:01	580.76			
		HLA-A*30:02	0.34412	HLA-A*30:02	874.73			
VP23	TLGLLLAYR (60-68)	HLA-A*33:01	0.296143	HLA-A*31:01	53.4	1.3897	Non-allergen	Non-toxic
		-	-	HLA-A*68:01	87.66			
		-	-	HLA-A*33:01	95.81			

**Table 3:** Top selected HTL epitopes selected for vaccine construction based on top antigenicity score.

Viral Protein	HTL epitopes (Start-end)	Interacting MHC-class I alleles from NetMHCIIPan-4.1 EL	IEDB score	Interacting MHC-class I alleles from NetMHCIIPan-4.1 BA	IC50 (Nm)	Antigenicity score	Allergenicity class	Toxicity class
gB	TNMVLRKR NKARYSP (876-890)	HLA-DRB1*13:02	0.6768	HLA-DRB1*11:01	68.45	1.0582	Non-allergen	Non-toxic
				HLA-DRB5*01:01	131.34			
				HLA-DRB1*13:02	198.46			
				HLA-DRB4*01:01	276.84			
RIR1	RAGRFHWER FSNASP (931-945)	HLA-DPA1*02:01/DPB1*05:01	0.3744	HLA-DPA1*02:01/DPB1*01:01	37.4	0.7292	Non-allergen	Non-toxic
		HLA-DPA1*02:01/DPB1*01:01	0.5265	HLA-DPA1*01:03/DPB1*04:01	20.51			
		HLA-DPA1*03:01/DPB1*04:02	0.7566	HLA-DPA1*01:03/DPB1*02:01	19.46			
		HLA-DPA1*01:03/DPB1*04:01	0.8902	HLA-DPA1*03:01/DPB1*04:02	37.38			
		HLA-DPA1*01:03/DPB1*02:01	0.9322	HLA-DPA1*02:01/DPB1*05:01	229.9			
ICP0	AVDFIWTGN QRTAPR (207-221)	HLA-DRB1*07:01	0.7357	HLA-DRB1*04:01	46.79	1.0626	Non-allergen	Non-toxic
		HLA-DRB1*04:01	0.7536					
		HLA-DRB3*02:02	0.4018					
VP23	AYRRRFPAV ITRVLP (66-80)	HLA-DPA1*02:01/DPB1*14:01	0.1371	HLA-DPA1*02:01/DPB1*14:01	106.33	0.5913	Non-allergen	Non-toxic
				HLA-DQA1*04:01/DQB1*04:02	50.72			

### 3.8 High helical content, favorable Z-scores, and stable energetics indicate robust vaccine constructs

**Figure 6:**
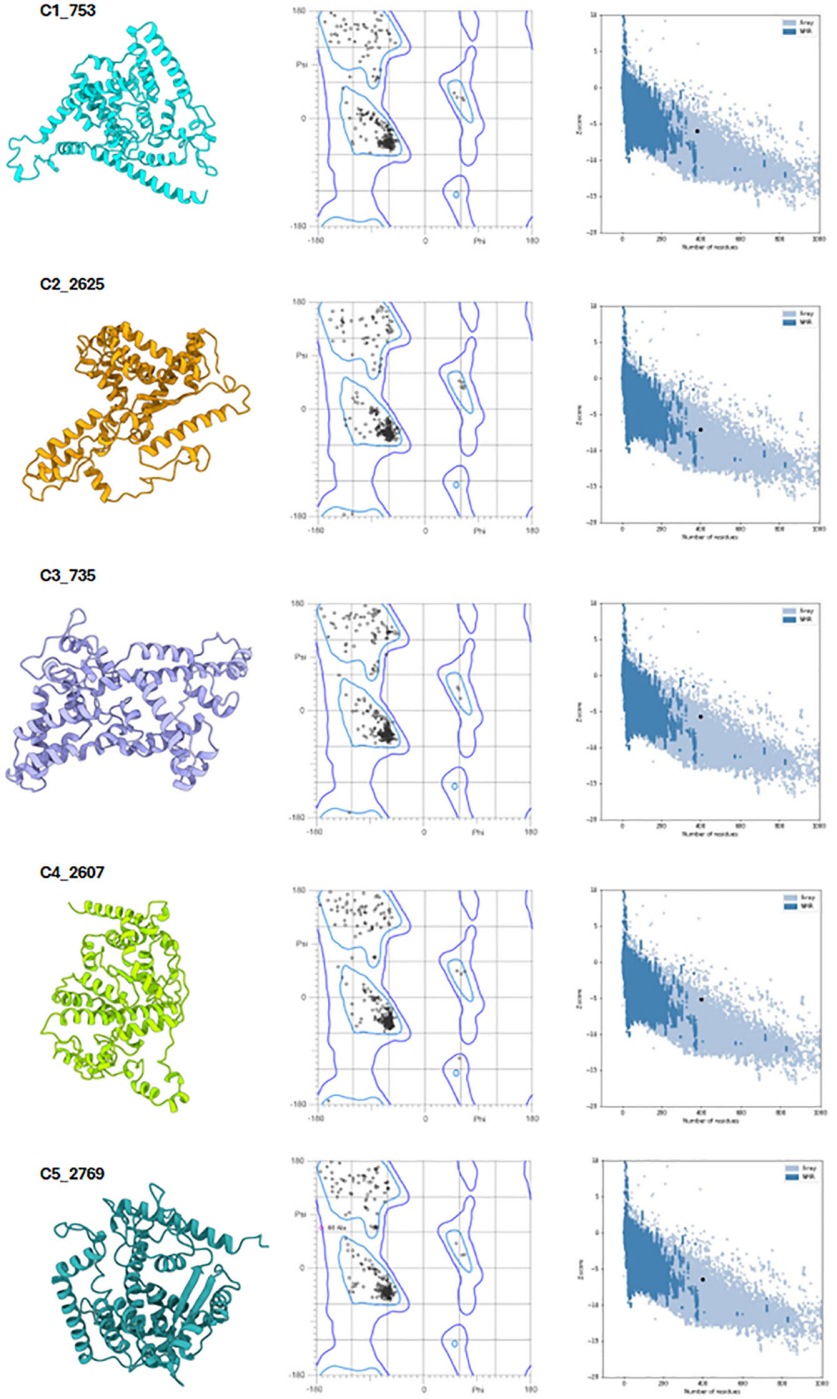
Structural validation of the vaccine constructs. Left panel: Predicted tertiary structures of the top five vaccine constructs. Middle panel: Ramachandran plots illustrating residue distribution, with 98–99.5% of residues in favored regions and 100% in allowed regions. Right panel: ProSA Z-score plots confirming structural integrity, with all constructs falling within the range of experimentally validated protein structures.

**Figure 7:**
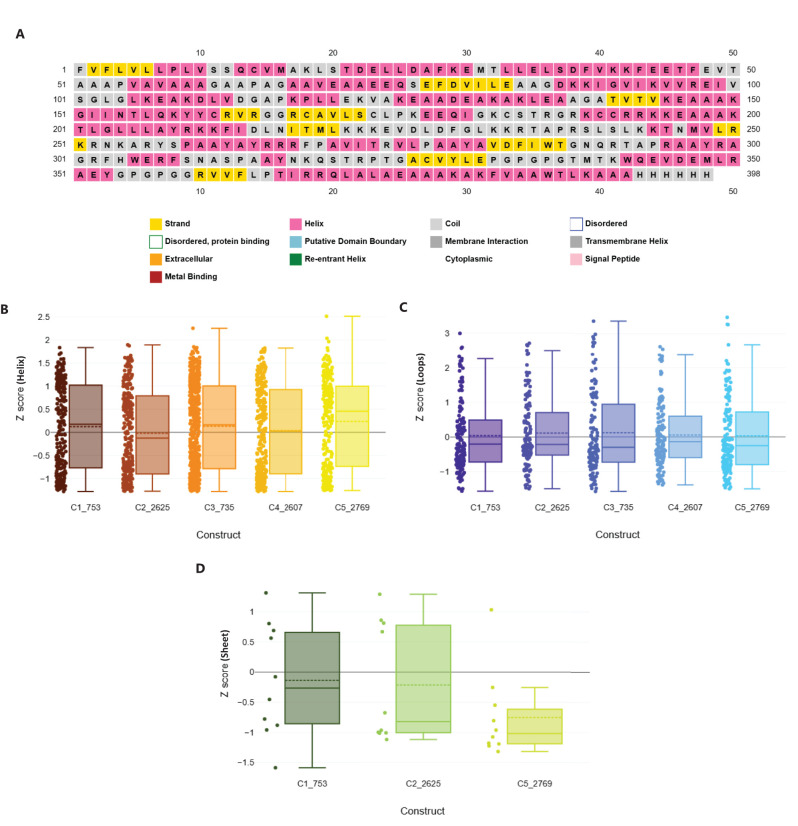
Structural geometry and quality assessment of vaccine constructs. **(A)** PSIPRED secondary structure prediction of construct C2_2625, representative of the five constructs. **(B)** Box plot of Rama-Z scores for helix-forming residues across all constructs, confirming favorable geometry. **(C)** Distribution of Rama-Z scores in loop regions, indicating minimal deviations. **(D)** Rama-Z scores for sheet regions, demonstrating overall structural stability.

Following assembly, the vaccines were modelled using the *ab initio* method via RosettaFold and AlphaFold (**Figure 6**, left panel). Secondary structure prediction via PSIPRED and SOPMA revealed high alpha-helical content ranging from 51.51% to 57.76%, signifying stable, ordered structures (**Supplementary Table 2**). The constructs were free of transmembrane helices, signal peptides, and disordered domains (**Figure 7A**, **Supplementary Data 4**). The tertiary structures of the vaccine constructs were modelled as previously described, and further analyzed for structural accuracy (43). Structural validation using MOLprobity confirmed that 98%–99.5% of residues in all constructs were within the favored Ramachandran plot regions, with 99.7%–100% in allowed regions (**Figure 6**, middle panel). No outliers were found in C2_2625 or C3_735, while C1_753, C4_2607, and C5_2769 each exhibited one minor outlier (**Supplementary Data 5**).

Further analysis using ProSA confirmed the structural integrity, with Z-scores ranging from -5.2 to -7.09, all within the experimentally validated Protein Data Bank (PDB) distribution (**Figure 6**, right panel) (44, 45). Among the constructs, C2_2625 exhibited the most favorable Z-score (-7.09), indicating high stability, while C4_2607 had the least favorable (-5.2) (**Supplementary Data 6**).

### 3.9 Exceptional protein structural integrity and favorable geometries in vaccine constructs

Geometric analysis using MOLprobity confirmed high structural quality across all constructs (46). Rotamer analysis revealed that over 98% of rotamers in each construct were in the favored category. Construct C3_735 had 307 favored rotamers (99.68%), while construct C1_753 contained 306 (99.35%). Similarly, constructs C4_2607, C2_2625, and C5_2769 exhibited high percentages of favored rotamers, with C5_2769 showing 98.38% due to a single poor rotamer (0.32%).

Rama-Z scores for all constructs remained within the acceptable range (<2), confirming favorable geometries: C3_735 (0.71 ± 0.38), C1_753 (1.94 ± 0.42), C4_2607 (-0.17 ± 0.41), C2_2625 (0.56 ± 0.41), and C5_2769 (1.25 ± 0.42) (**Figures 7B–D**). No Cβ deviations exceeding 0.25 Å were observed, indicating precise atomic positioning. Bond and angle deviations were minimal, with the highest incidence of bad bonds recorded at 0.10% in C2_2625, while C3_735, C1_753, C4_2607, and C5_2769 had the lowest rate (0.03%). Bad angles were also infrequent, with C4_2607 exhibiting the highest rate at 0.26% and C5_2769 the lowest at 0.12% (**Supplementary Data 5**).

### 3.10 Evaluation of proteasomal cleavage, surface exposure, solubility, and glycosylation in mRNA vaccine Candidates

NetChop-3.1 analysis identified proteasomal cleavage sites in all constructs, ensuring efficient peptide generation for MHC-I presentation. Constructs C1_753 and C2_2625 contained 133 cleavage sites, C3_735 and C4_2607 had 132, while C5_2769 exhibited 131 (**Supplementary Data 7**), confirming their suitability for effective proteasomal processing. NetSurfP-3.0 predictions indicated high surface accessibility across all constructs, with exposed residues comprising 77–79% of the total and buried residues accounting for 21–23%. Specifically, constructs C1_753, C2_2625, and C3_735 exhibited exposed residue percentages of 77.14%, 77.89%, and 77.89%, respectively, whereas C4_2607 and C5_2769 displayed slightly higher values at 79.15% and 77.64% (**Supplementary Data 7**).

Potential N-linked glycosylation sites were predicted using the NetNGlyc 1.0 server. All constructs, each comprising 397 amino acids, contained the canonical Asn-Xaa-Ser/Thr sequon. A conserved glycosylation site was identified in the “NITM” motif across all constructs. In constructs C1_753 and C3_735, this site was located at position 204, while in C2_2625, C4_2607, and C5_2769, it appeared at position 215. This NITM site exhibited high glycosylation potential (scores: 0.6417–0.6438), with strong algorithmic consensus. These findings indicate a single, conserved, and likely glycosylated site across all constructs (**Supplementary Data 7**).

Solubility analysis using SOLpro predicted all constructs to be soluble, with solubility probabilities surpassing the threshold value of 0.45. Each construct attained a solubility score above 0.660, confirming their favorable solubility profiles for experimental applications (**Supplementary Data 8**).

### 3.11 Evaluation of codon optimization and mRNA vaccine stability analysis

All vaccine constructs were 1,149 bp in length, with a GC content of approximately 60%. Codon Adaptation Index (CAI) values ranged from 0.92 to 0.93 after optimization, indicating a strong alignment with the host organism’s preferred codon usage (47). Among the constructs, C753 and C2625 exhibited the highest CAI scores (0.93), reflecting optimal codon adaptation. Effective Number of Codons (ENC) values ranged from 31.83 to 32.66, demonstrating a high degree of codon bias (48). The tRNA Adaptation Index (tAI), which evaluates how a gene’s codon usage corresponds to the availability of tRNAs in the host cell (49, 50), was consistently measured at 0.41 across all constructs, suggesting efficient codon utilization and compatibility with the host’s translational machinery (**Supplementary Table 3**, **Supplementary Data 9**).

**Figure 8:**
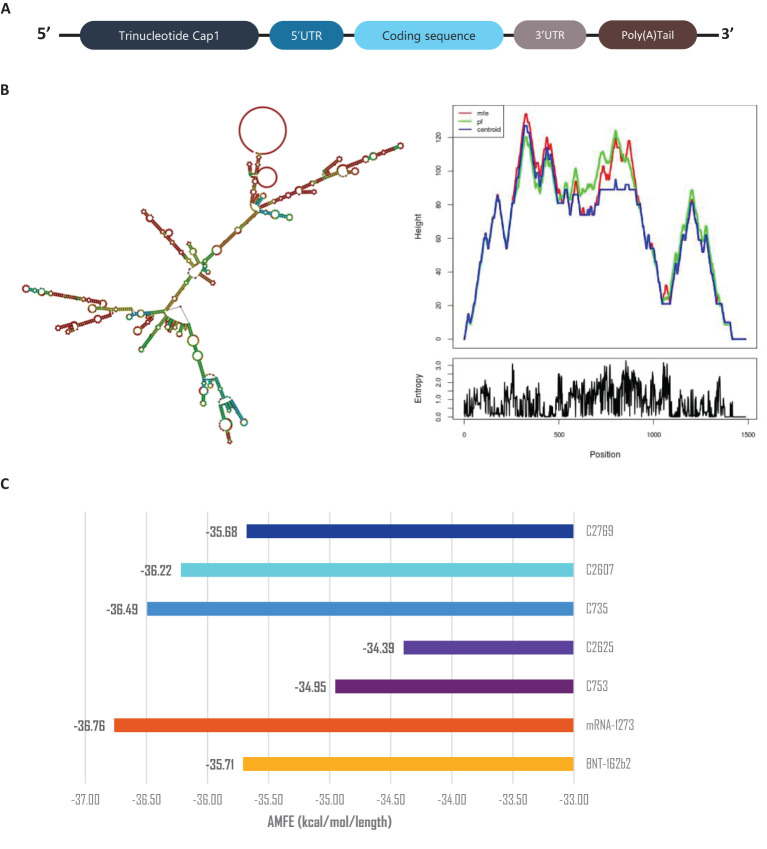
mRNA stability and secondary structure analysis. **(A)** Schematic representation of mRNA vaccine assembly. **(B)** Predicted mRNA secondary structure of construct C3_735, with corresponding mountain plot visualization, representative of the five constructs. **(C)** Adjusted Minimum Free Energy (AMFE) comparison of the five vaccine constructs, benchmarked against mRNA-1273 and BNT162b2, demonstrating comparable stability. AMFE normalizes MFE to account for sequence length differences.

The secondary structures of the mRNA constructs were predicted and analyzed after assembling the mRNA with Cap’1, Poly-A tail, and UTRs (**Figure 8A**) using Vienna RNAFold (**Figure 8B** and **Supplementary Data 10**) (34, 51). Minimum Free Energy (MFE) values were normalized using Adjusted Minimum Free Energy (AMFE) to enable comparisons between constructs of varying lengths (52). Benchmarking against Pfizer’s BNT162b2 and Moderna’s mRNA-1273 vaccines, which exhibited AMFE values of -35.71 kcal/mol and -36.76 kcal/mol, respectively (53, 54), confirmed the structural stability of the designed constructs. The AMFE values for the vaccine constructs ranged from -34.39 kcal/mol (C2_2625) to -36.49 kcal/mol (C3_735), indicating a stability profile comparable to the benchmark vaccines (**Figure 8C**). Among all constructs, C3_735 demonstrated the highest stability.

### 3.12 Molecular docking between Epitopes and their corresponding HLA class alleles

Molecular docking analysis demonstrated strong binding interactions between the selected epitopes and their respective HLA class alleles. Docking of HLA-B44:03 (PDB ID: 3DX7) with the epitope KEVDLDFGL resulted in a cluster comprising 232 members, with a representative weighted score of -469.3 and the lowest energy score of -545.7. Similarly, HLA-B57:01 (PDB ID: 5VUF) docked with the epitope RTAPRSLSL formed a cluster with 539 members, achieving a representative weighted score of -584.7 and the lowest energy score of -677.4. The interaction between HLA-A 02:06 (PDB ID: 3OXR) and the epitope FIDLNITML produced a cluster of 816 members, with a representative weighted score of -581.5 and the lowest energy score of -702.6. Likewise, docking of HLA-DRB1*04:01 (PDB ID: 5NI9) with the epitope AVDFIWTGNQRTAPR yielded a cluster of 173 members, with a representative weighted score of -779.9 and the lowest energy score of -811.5 (**Supplementary Data 11**).

**Figure 9:**
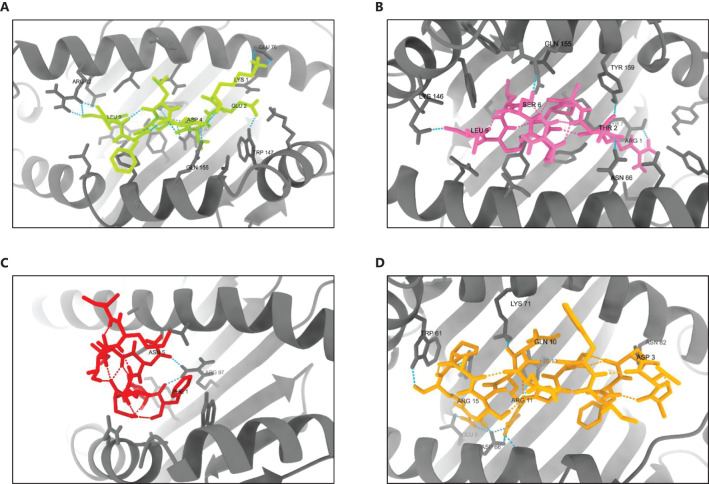
3D structures of the best-docked poses of selected epitopes with their corresponding HLA alleles. **(A)** HLA-B44:03 bound to KEVDLDFGL, **(B)** HLA-B57:01 bound to RTAPRSLSL, **(C)** HLA-A02:06 bound to FIDLNITML, and **(D)** HLA-DRB104:01 bound to AVDFIWTGNQRTAPR. Docking interactions were visualized and analyzed using UCSF ChimeraX 1.8.

The 3D structures of the docked poses, shown in **Figure 9**, provide insights into the spatial arrangements and binding orientations of the epitopes within the HLA binding grooves. Further analysis of protein-ligand interactions using PDBsum, along with detailed LIGPLOT representations (**Supplementary Data 12**), confirmed the robustness of these interactions and underscored the immunogenic potential of the selected epitopes.

### 3.13 Molecular docking between vaccine constructs and TLR2 and TLR4/MD2 complex

**Figure 10:**
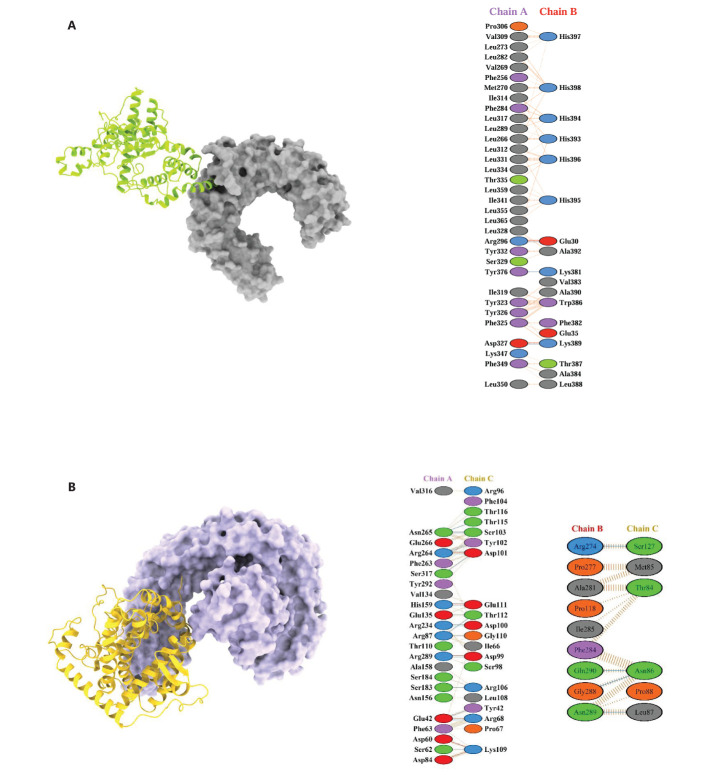
3D docked poses and protein-protein interactions of vaccine constructs with immune receptors. **(A)** TLR2 (Chain A) complexed with C4_2607 (Chain B) and **(B)** TLR4 complexed with C5_2769. These models are shown as representatives of the five designed vaccine constructs.

The molecular docking analysis demonstrated significant binding interactions between the vaccine constructs and the TLR2 and TLR4/MD2 complexes (**Figures 10A, B**). For TLR2, docking with the C3_735 construct resulted in a cluster of 202 members, with a representative weighted score of -1126.1 and the lowest energy score of -1443.1. Similarly, TLR2 docked with C1_753 formed a cluster with 81 members, yielding a representative weighted score of -875.4 and the lowest energy score of -946.1. The largest cluster size was observed for TLR2 with C4_2607, comprising 277 members, with a representative weighted score of -1268.6 and the lowest energy score of -1568.9. Additionally, TLR2 docking with C2_2625 produced a cluster of 140 members, achieving a representative weighted score of -1018.0 and the lowest energy score of -1039.3, while TLR2 docked with C5_2769 resulted in a cluster of 141 members, with a representative weighted score of -1093.1 and the lowest energy score of -1336.4.

For TLR4, docking with C3_735 identified a cluster of 127 members, with a representative weighted score of -912.5 and the lowest energy score of -1157.8. Similarly, TLR4 with C1_753 formed a cluster of 45 members, yielding a representative weighted score of -808.0 and the lowest energy score of -914.6. Docking of TLR4 with C4_2607 generated a cluster with 140 members, a representative weighted score of -958.1, and the lowest energy score of -1204.2. TLR4 interaction with C2_2625 produced a cluster of 57 members, with a representative weighted score of -972.1 and the lowest energy score of -1131.2, while TLR4 docked with C5_2769 resulted in a cluster of 57 members, achieving a representative weighted score of -1111.8 and the lowest energy score of -1176.2.

These docking results highlight strong binding interactions across all vaccine constructs with both TLR2 and TLR4, reinforcing their potential to effectively stimulate innate immune responses.

### 3.14 Molecular dynamics simulation

**Figure 11:**
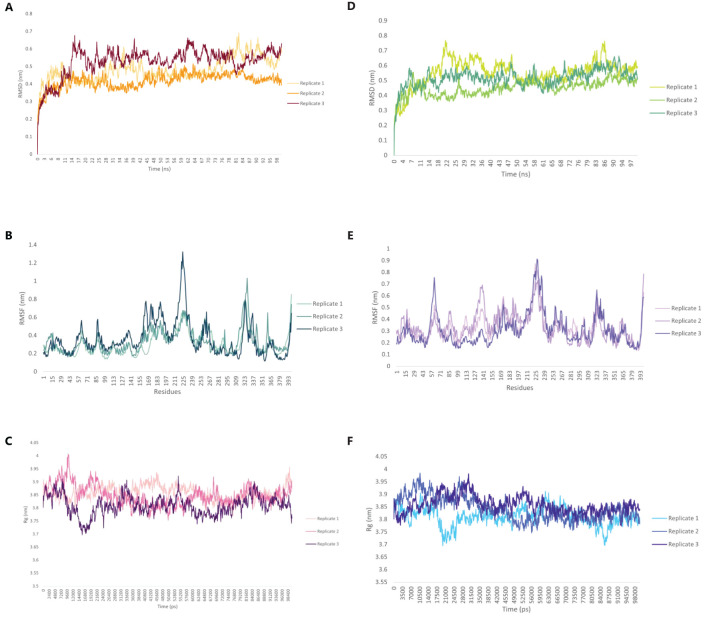
Structural dynamics of the TLR4/MD2-C5_2769 or TLR2-C4_2607 complexes. Root Mean Square Deviation (RMSD) plot showing the structural stability of the **(A)** TLR4/MD2-C5_2769 and **(B)** TLR2-C4_2607 complexes over a 100 ns simulation. Root Mean Square Fluctuation (RMSF) plots illustrating the flexibility of individual residues in the **(C)** TLR4/MD2-C5_2769 and **(D)** TLR2-C4_2607 complexes. Radius of Gyration (Rg) plots depicting the compactness of the **(E)** TLR4/MD2-C5_2769 and **(F)** TLR2-C4_2607 complexes, indicating their structural integrity throughout the 100 ns (100,000 ps) simulation.

Molecular dynamics (MD) simulations were conducted to evaluate the structural stability and dynamic behavior of the C5_2769-TLR4/MD2 and C4_2607-TLR2 complexes (**Figure 11**). The average root mean square deviation (RMSD) values for the C5_2769-TLR4/MD2 complex were 0.480 nm, 0.417 nm and 0.527 nm for replicates 1, 2 and 3, respectively (**Figure 11A**). Similarly, the C4_2607-TLR2 complex exhibited RMSD values of 0.551 nm, 0.448 nm, and 0.515 nm across the three replicates (**Figure 11B**), indicating stable conformational dynamics throughout the simulation. Root mean square fluctuations (RMSF) analysis highlighted minimal residue flexibility in both complexes, with slightly higher fluctuations observed in loop regions and terminal residues, which is expected due to their inherent flexibility (**Figures 11C, D**). These results further confirm the overall structural stability of both complexes over the 100 ns simulation period. To evaluate the overall compactness of the complexes, the radius of gyration (Rg) was analyzed. The average Rg values for the C5_2769-TLR4/MD2 complex were 3.858 nm, 3.847 nm, and 3.809 nm across replicates 1, 2, and 3, respectively (**Figure 11E**). For the C4_2607-TLR2 complex, the average Rg values were 3.809 nm, 3.843 nm, and 3.857 nm across the three replicates (**Figure 11F**). These findings suggest that both complexes maintained stable structural integrity throughout the simulation.

### 3.15 Immune simulation reveals strong antibody and T-cell responses to vaccine constructs

The immune responses elicited by the five vaccine constructs were assessed using the C-ImmSim simulation tool, which has been validated against clinical data (55, 56). Antigen (Ag) levels rapidly exceeded 600,000 counts/mL following administration, with constructs C2_2625 and C5_2769 exhibiting the highest levels (>650,000 counts/mL), indicating strong initial immune activation. Peak IgM and IgG responses were observed between 10 and 15 days post-vaccination, with construct C5_2769 eliciting the highest combined antibody response (>10,000 counts/mL), closely followed by C2_2625. Isolated IgM responses ranged between 5,000 and 8,000 counts/mL, while IgG1 and IgG2 levels reached approximately 2,000 counts/mL by days 15–20, demonstrating a potent humoral response.

Memory B cells (B-mem, y2) exceeded 200 cells/mm³ across all constructs by day 5 and remained at this level throughout the observation period, suggesting a sustained immunological memory. B cell population dynamics revealed distinct trends, with presenting B cells (y2) peaking at >350 cells/mm³ by day 5 before declining to 0 by days 9–10, marking a transition to active B cell states. Active B cells initially declined below 100 cells/mm³ between days 0–5, followed by a peak of >300–400 cells/mm³ between days 5–10, stabilizing thereafter. Plasma B lymphocytes showed construct-dependent variations, with IgM-producing plasma cells peaking at 6–9 cells/mm³ between days 5–10 before declining to undetectable levels by days 25–30. Similarly, IgG1-producing plasma cells peaked at >2 cells/mm³ between days 5–10 and decreased to zero after day 25, indicating a transient yet strong humoral response.

CD4^+^ T helper (TH) cells exhibited exponential memory expansion, stabilizing at >300–350 cells/mm³ across all constructs. Non-memory CD4^+^ cells increased from baseline levels (1,000–1,500 cells/mm³) to peaks exceeding 4,000 cells/mm³ between days 5–10 before gradually declining after day 15. CD8^+^ cytotoxic T cells displayed a rapid increase, reaching 1,150 cells/mm³ between days 10–15, followed by a slight decline while remaining above baseline levels through day 35.

Dendritic cells (DCs) in the presenting-2 state peaked at 50 cells/mm³ by day 5 before declining, while presenting-1 DCs remained stable at baseline levels. Macrophage (MA) populations exhibited dynamic behavior, with presenting-2 macrophages increasing to >100 cells/mm³, while resting macrophages initially dropped to near zero between days 0–5 before recovering post-day 5.

**Figure 12:**
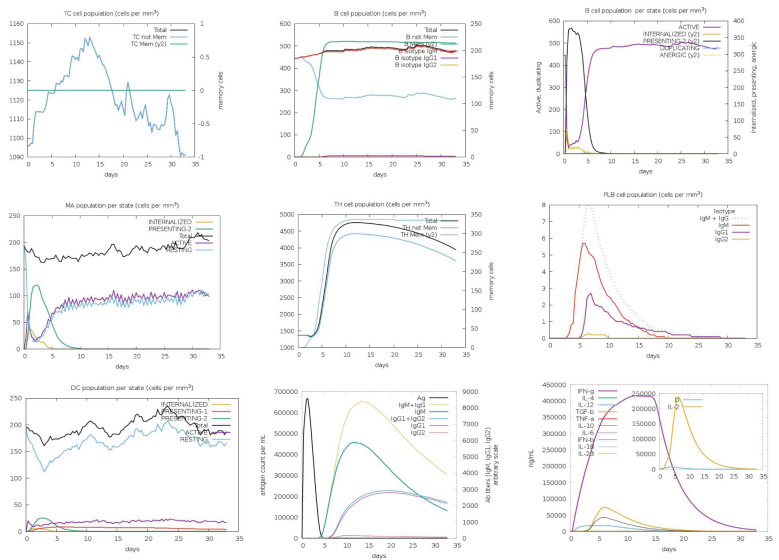
Immune response profile of vaccine construct C3_735. The immune response dynamics induced by C3_735 are shown as a representative of the five designed vaccine constructs. Antigen (Ag) levels exceeded 600,000 counts/mL, indicating strong initial activation. IgM and IgG responses peaked between days 10–15, with IgG1 and IgG2 reaching approximately 2,000 counts/mL. Memory B cells (B-mem) maintained levels above 200 cells/mm³, ensuring long-term immune protection. CD4+ T helper cells exhibited exponential growth, stabilizing at >300–350 cells/mm³, while CD8+ cytotoxic T cells peaked at 1,150 cells/mm³ between days 10–15. Cytokine responses included IFN-γ levels surpassing 400,000 ng/mL and IL-2 peaking at 250,000 ng/mL, indicating a robust immune activation.

Cytokine analysis indicated significant immune activation. Interferon-gamma (IFN-γ) levels peaked above 400,000 ng/mL between days 10–15, reflecting heightened immune stimulation before gradually decreasing. Transforming growth factor-beta (TGF-β) levels increased to >50,000 ng/mL between days 0–5 before declining. Interleukin-2 (IL-2) levels surged sharply to 200,000–250,000 ng/mL by day 5, returning to baseline levels between days 15–20. Collectively, these findings highlight the robust and well-coordinated immune responses induced by the vaccine constructs (**Figure 12**).

## 4 Discussion

HSV-2 poses a significant public health challenge as a leading cause of genital infections and a gateway virus to other sexually transmitted diseases. Despite its widespread prevalence, there is currently no licensed vaccine available for HSV-2 (57). The development of an effective vaccine against HSV-2 remains a formidable challenge due to several intrinsic features of the virus’s biology and its sophisticated immune evasion strategies. One of the key mechanisms is the downregulation of MHC class I molecules, which impairs the activation of CD8⁺ cytotoxic T lymphocytes and compromises the host’s capacity to generate a robust and sustained immune response (58). Additionally, HSV-2 establishes latency in the nervous system and undergoes periodic reactivation, further complicating vaccine design. The virus also exhibits considerable genetic variability, with a mutation rate higher than that of many other DNA viruses, posing significant obstacles to the development of a broadly protective and universally effective vaccine.

Several HSV-2 vaccine candidates, such as Herpevac (gD2), GEN-003, VCL-HB01, and the Chiron recombinant gB2/gD2 vaccine initially showed promise but ultimately failed to demonstrate significant protection or meet efficacy benchmarks in clinical trials. These shortcomings were largely attributed to limited immunogenicity and a narrow antigenic focus (11, 59–61). These failures highlight the urgent need for vaccine strategies capable of eliciting broader and more robust immune responses. In response, current efforts have pivoted toward next-generation platforms, particularly mRNA-based vaccines. Moderna’s mRNA-1608 (a monovalent vaccine targeting a single glycoprotein) and BioNTech’s BNT163 (a trivalent vaccine encoding three glycoproteins) are both in preclinical or early clinical evaluation, alongside five additional vaccine candidates utilizing diverse technological platforms (62).

The use of multivalent multiepitope mRNA vaccines has emerged as a promising strategy. These vaccines combine the ability to encode multiple epitopes within a single construct to activate robust humoral and cellular immune responses (17, 63, 64). This study employed a reverse vaccinology approach to design and develop five multivalent multiepitope mRNA vaccines targeting HSV-2. Four viral proteins—Glycoprotein B, ICP0, RIR1, and VP23—were selected as targets for vaccine development based on their essential roles in viral replication, immune evasion, and persistence (65–68). In contrast to earlier strategies that primarily focused on single antigens or limited epitope selections (such as glycoprotein D-based vaccines), our multiepitope design aims to elicit a broader and potentially cross-protective immune response. To enhance the global applicability of the vaccine constructs, we incorporated sequence data from three geographically diverse and prevalent HSV-2 strains.

Extensive predictions using NetMHCpan 4.1 identified thousands of potential Cytotoxic T Lymphocyte (CD8^+^) epitopes. Rigorous filtering reduced these to 55 epitopes for Glycoprotein B, 22 for ICP0, 33 for RIR1, and 16 for VP23, demonstrating strong binding capabilities. Similarly, Helper T Lymphocyte (CD4^+^) epitope predictions underwent stringent analysis, resulting in 36 epitopes for Glycoprotein B, 28 for ICP0, 42 for RIR1, and 24 for VP23 after evaluations for antigenicity, allergenicity, toxicity, and cytokine induction. Linear B-cell epitope predictions were refined to one epitope each for Glycoprotein B and ICP0, 12 for RIR1, and 2 for VP23. Discontinuous B-cell epitope predictions via ElliPro identified high-quality epitopes characterized by geometric protrusion and solvent accessibility, supported by 3D structural visualizations. Population coverage analysis revealed combined Class I and II coverage of 88.64%, with higher representation in Europe and North America, confirming the constructs’ broad applicability across different populations (69).

Vaccine assembly yielded 3,456 constructs based on various permutations and combinations. Three adjuvants—50S ribosomal protein from *Mycobacterium tuberculosis* (UniProt ID: G8FRW4), Human β-defensin 3 (GIINTLQKYYCRVRGGRCAVLSCLPKEEQIGKCSTRGRKCCRRKK), and PADRE sequence (AKFVAAWTLKAAA)—were incorporated to enhance immune responses. These adjuvants have been shown to promote dendritic cell maturation, T-cell-mediated cytotoxicity, and Th1 polarization (70–72). The vaccine constructs were linked using EAAKK, KK, AAY, and GPGPG linkers as previously described (73). The final constructs consisted of 396 amino acid residues and exhibited favorable physiochemical properties, including molecular weights of approximately 41.86 kDa, theoretical pI values suggesting basic protein types, and hydrophilic GRAVY index values. Stability analysis revealed low instability indices and positive solubility predictions, indicating potential suitability for vaccine development.

Secondary structure predictions using PSIPRED and SOPMA showed a predominance of α-helices, indicating structural stability (74). Tertiary structure evaluations with MOLprobity revealed high-quality structures, with 99.2% to 100% of residues falling within favored and allowed regions on the Ramachandran plot. All constructs displayed acceptable Rama-Z scores and favorable Z-scores, confirming structural integrity and low-energy conformations (75, 76). Geometric analyses highlighted minimal bond and angle deviations, further supporting the constructs’ stability and accuracy.

Proteasomal cleavage is vital for triggering a CD8^+^ T cell response, as it processes antigens into fragments for MHC class I presentation in the endoplasmic reticulum (77). Using NetChop 3.1, we verified that all five vaccine constructs could generate peptides suitable for MHC-I presentation. The analysis revealed 133 cleavage sites in constructs C1_753 and C2_2625, 132 in C3_735 and C4_2607, and 131 in C5_2769, confirming their ability to elicit a cytotoxic T-cell response. The surface accessibility of protein residues plays a critical role in antigenicity, as exposed regions are more likely to interact with immune components like antibodies. Antigenic determinants often coincide with surface regions accessible to large probes, such as antibody domains (78). The surface accessibility predictions, indicated that 77-79% of the residues were exposed, reinforced the constructs’ ability to be recognized by the immune system. Moreover, the solubility predictions confirmed that all constructs would remain stable in physiological conditions, further supporting their potential for vaccine development.

Codon optimization analysis revealed high compatibility with the host organism, with all constructs exhibiting high Codon Adaptation Index (CAI) values (0.92-0.93). The stability of the mRNA vaccine constructs were benchmarked with established vaccines, such as those developed by Pfizer and Moderna based on the Adjusted Minimum Free Energy (AMFE) value (53, 54), further indicating the feasibility of developing an mRNA-based vaccine using these constructs.

The molecular docking studies between epitopes and their corresponding Class I and II HLA alleles demonstrated strong binding affinities, highlighting the immunogenic potential of the selected epitopes. Molecular docking between vaccine constructs with TLR2 and TLR4/MD2 complex exhibited significant binding energies, which suggests strong interactions with immune receptors. All five potential vaccine constructs developed against HSV-2 demonstrate good metrics in terms of antigenicity, immune profiles, and protein and mRNA stability. These findings show the potential of these constructs as candidates for further development of effective vaccines against HSV-2. The MD simulations provided critical insights into the stability and dynamic behavior of the vaccine constructs in complex with TLR receptors. The low RMSD values, stable Rg measurements, and minimal RMSF fluctuations collectively indicate that the vaccine constructs C4_2607 and C5_2769 forms a stable interaction with TLR2 and TLR4/MD2, respectively. These findings support the structural viability of the designed constructs, highlighting their potential for further *in vitro* and *in vivo* evaluations.

The limitation of this study is the reliance on *in silico* predictions for epitope identification and vaccine construct design, which may not fully account for the complexity of immune responses *in vitro* and *in vivo*. While the stability and antigenicity of the vaccine constructs were evaluated computationally, further experimental validation through immunological assays and animal model testing is needed to confirm their efficacy and safety. Additionally, the population coverage analysis revealed the lowest coverage in Central America, which could be addressed by incorporating epitopes that bind to HLA alleles more commonly expressed in this population. Although the mRNA constructs demonstrated good stability, they may be detected by pathogen recognition receptors such as TLR3 and RLRs, and this can be mitigated by substituting all uridine in the mRNA constructs with N1-methylpseudouridine (m1ψ), which has been shown to reduce mRNA-induced immunogenicity which is a critical factor in mRNA vaccine design and also to enhance translation efficiency (79, 80).

In conclusion, the five multivalent multiepitope mRNA vaccine constructs developed against HSV-2 in this study exhibits favorable antigenicity, strong immune profiles, and excellent protein and mRNA stability. These promising characteristics position them as strong candidates for further development, offering potential for effective HSV-2 vaccination strategies.

## Data Availability

The original contributions presented in the study are included in the article/**Supplementary Material**. Further inquiries can be directed to the corresponding author.
