# Supplementary material for: Reverse vaccinology-based design of multivalent multiepitope mRNA vaccines targeting key viral proteins of Herpes Simplex Virus type-2
Source: Front Immunol. 2025 May 20;16:1586271. doi: 10.3389/fimmu.2025.1586271 (PMC12130045; doi:10.3389/fimmu.2025.1586271)
Supplement: Supplementary file 1 [file DataSheet1.zip › Supplementary Data_22-04-2025/Supplementary Data 10.pdf]

RNAfold WebServer

1 Enter Input Parameters 2 View Results

[Home|New job|Help]

Results for minimum free energy prediction

The optimal secondary structure in dot-bracket notation with a minimum free energy of **-519.40** kcal/mol is given below.
[color by base-pairing probability | color by positional entropy | no coloring]

```
1       GAGACAUUUGCUUCUGACACAACUGUGUUCACUAGCAACCUCAAACAGACACCGCCACCAUGUUCGUGUUCCUGGUGCUGCUGCCCCUGGUGAGCAGCCAGUGCGUGAUGGCCAAACUGAGCACCGACGAGCUGCUGGACGCCUUCAAGGAGAUGACC
160     GCUCGAGCUGUCUGAUUUUGUGAAGAAGUUUGAGGAGACAUUCGAGGUGACCGCCGCCGCUCCUGUGGCCGUGGCCGCCGCAGGAGCUGCUCCUGCUGGCGCCGCCGUGGAAGCCGCCGAAGAGCAGAGCGAGUUCGACGUGAUCCUGGAAGCCGCUG
320     GACAAGAAGAUCGGCGUGAUCAAGGUGGUGCGGGAAAUCGUGUCCGGCCUCGGCCUGAAGGAAGCCAAGGACCUGGUGGACGGCGCUCCAAAGCCUCUGCUGGAAAAGGUGGCCAAGGAAGCCGCUGAUGAGGCCAAAGCCAAGCUGGAAGCCGCCGG
480     CUACAGUGACCGUGAAGGAGGCCGCUGCCAAAGGCAUCAUCAACACCCUGCAGAAAUACUACUGCAGAGUGCGGGGCGGACGGUGCGCCGUGCUGUCCUGCCUGCCUAAGGAAGAGCAAAUCGGAAAGUGCAGCACAAGAGGCAGAAAAUGUUGUAGA
640     GAAAAAGGAGGCUGCCGCCAAGUUCAUCGACCUGAAUAUCACCAUGCUGAAAAAGACCCUGGGCCUGCUGCUGGCCUACCGGAAGAAGAGAACCGCCCCCAGAAGCCUGAGCCUGAAGAAAAAGGAAGUGGACCUGGAUUUCGGCCUGAAGAAGACCA
800     AUGGUCCUGCGGAAGCGGAACAAGGCCAGAUACAGCCCUGCCGCCUACGCCUACAGAAGGCGCUUCCCCGCCGUGAUUACAAGAGUGCUGCCUGCCGCUUAUGCCGUUGACUUCAUCUGGACCGGCAACCAGAGAACAGCCCCCAGAGCCGCUUAUCG
960     CCGGACGGUUCCACUGGGAGAGAUUCAGCAAUGCCAGCCCCGCUGCCUACAACAAGCAGUCUACAAGACCUACCGGAGCAUGCGUGUACCUGGAACCUGGCCCUGGCCCAGGCACCAUGACCAAGUGGCAGGAGGUGGAUGAGAUGCUGAGAGCCGAG
1120    CGGCCCCGGACCUGGCGGCAGAGUCGUCUUUCUGCCCACCAUCAGAAGACAGCUGGCCCUGGCCGAGGCUGCCGCCAAAGCCAAGUUCGUGGCCGCAUGGACACUGAAGGCCGCCGCCCACCACCACCACCACCAUUGAUAAUAGGCUGGAGCCUCGG
1280    GCCUAGCUUCUUGCCCCUUGGGCCUCCCCCCAGCCCCUCCUCCCCUUCCUGCACCCGUACCCCCGUGGUCUUUGAAUAAAGUCUGAGUGGGCGGCAAAAAAAAAAAAAAAAAAAAAAAAAAAAAAAAAGCAUAUGACUAAAAAAAAAAAAAAAAAAAAAAA
1440    AAAAAAAAAAAAAAAAAAAAAAAAAAAAAAAAAAAAAAAAAAAAA
```

```
1       .((.(((.(((((..(((((....))))).....((..((.((..(((((..((((.....(((((.....(((((((((((.........)))))(((((......))...))))))))))))))))(((((.((.((((.((((.(((...
160     .(((((((.(((..........)))))))))).))...((((.(((..((((.(((((....))))).(((((.((((((((((...))))))).)))))))).))))..)))..))))((((.......))))..)))..)))).)))).)))))
320     )..........)))).(((((.((.((((((((...(((((....((((.((((...(((..((((.((.((.((((.(((..(((((...(((((((((......(((((.......)))))(((((.(((..(((...(((((......)))))
480     )).(((((.((........)).)))))).....))).)))))).(((.((((((........)))))).)))((((((((((((....)))).))))))))(((.....)))..((.....))........))))...))))))......(((((((.
640     (.....((.(((((..((...(((((......)))))).......((((((.....(((((((((.......))))))..........(((((.((......((((((.(((........)))(((((((..(((((((.((((..((((..(((((
800     .)))))((((....))))....(((((((........)))..)))).(((((.....)))))))))...)))).)))..((.((((((.....)).)))).))..((((..(((.(((((........)))))))))))..)))).).))))))).)))
960     ).)).))))))....))).....))))))).)))))))))).))).))))))))....((((...))))....)))))...))).)))).))..)))))))))))))).)))).)))))......((((((((.(((.(((..((((...((((..
1120    ))))........(((.(((((((......)))))))..)))....((((.((((((((...(((((((((.(((((........((((((((....)))))))......(((....)))..........................))).)))))))))
1280    )))).))))))))((((...))))......)))).)))).))).))))))))).)))))))).)).)))))..........))))))..))))..))...........................)))))).))).))..........................
1440    ............................................
```

You can download the minimum free energy (MFE) structure in [Vienna Format| Ct Format]. You can get thermodynamic details on this structure by submitting to our RNAeval web server.

Results for thermodynamic ensemble prediction

The free energy of the thermodynamic ensemble is **-537.90** kcal/mol.

The frequency of the MFE structure in the ensemble is **0.00** %.

The ensemble diversity is **282.44**.

You may look at the **dot plot** containing the base pair probabilities [EPS|PDF|IMAGE CONVERTER].

The centroid secondary structure in dot-bracket notation with a minimum free energy of **-461.00** kcal/mol is given below.
[color by base-pairing probability | color by positional entropy | no coloring]

```
1       GAGACAUUUGCUUCUGACACAACUGUGUUCACUAGCAACCUCAAACAGACACCGCCACCAUGUUCGUGUUCCUGGUGCUGCUGCCCCUGGUGAGCAGCCAGUGCGUGAUGGCCAAACUGAGCACCGACGAGCUGCUGGACGCCUUCAAGGAGAUGACC
160     GCUCGAGCUGUCUGAUUUUGUGAAGAAGUUUGAGGAGACAUUCGAGGUGACCGCCGCCGCUCCUGUGGCCGUGGCCGCCGCAGGAGCUGCUCCUGCUGGCGCCGCCGUGGAAGCCGCCGAAGAGCAGAGCGAGUUCGACGUGAUCCUGGAAGCCGCUG
320     GACAAGAAGAUCGGCGUGAUCAAGGUGGUGCGGGAAAUCGUGUCCGGCCUCGGCCUGAAGGAAGCCAAGGACCUGGUGGACGGCGCUCCAAAGCCUCUGCUGGAAAAGGUGGCCAAGGAAGCCGCUGAUGAGGCCAAAGCCAAGCUGGAAGCCGCCGG
480     CUACAGUGACCGUGAAGGAGGCCGCUGCCAAAGGCAUCAUCAACACCCUGCAGAAAUACUACUGCAGAGUGCGGGGCGGACGGUGCGCCGUGCUGUCCUGCCUGCCUAAGGAAGAGCAAAUCGGAAAGUGCAGCACAAGAGGCAGAAAAUGUUGUAGA
640     GAAAAAGGAGGCUGCCGCCAAGUUCAUCGACCUGAAUAUCACCAUGCUGAAAAAGACCCUGGGCCUGCUGCUGGCCUACCGGAAGAAGAGAACCGCCCCCAGAAGCCUGAGCCUGAAGAAAAAGGAAGUGGACCUGGAUUUCGGCCUGAAGAAGACCA
800     AUGGUCCUGCGGAAGCGGAACAAGGCCAGAUACAGCCCUGCCGCCUACGCCUACAGAAGGCGCUUCCCCGCCGUGAUUACAAGAGUGCUGCCUGCCGCUUAUGCCGUUGACUUCAUCUGGACCGGCAACCAGAGAACAGCCCCCAGAGCCGCUUAUCG
960     CCGGACGGUUCCACUGGGAGAGAUUCAGCAAUGCCAGCCCCGCUGCCUACAACAAGCAGUCUACAAGACCUACCGGAGCAUGCGUGUACCUGGAACCUGGCCCUGGCCCAGGCACCAUGACCAAGUGGCAGGAGGUGGAUGAGAUGCUGAGAGCCGAG
1120    CGGCCCCGGACCUGGCGGCAGAGUCGUCUUUCUGCCCACCAUCAGAAGACAGCUGGCCCUGGCCGAGGCUGCCGCCAAAGCCAAGUUCGUGGCCGCAUGGACACUGAAGGCCGCCGCCCACCACCACCACCACCAUUGAUAAUAGGCUGGAGCCUCGG
1280    GCCUAGCUUCUUGCCCCUUGGGCCUCCCCCCAGCCCCUCCUCCCCUUCCUGCACCCGUACCCCCGUGGUCUUUGAAUAAAGUCUGAGUGGGCGGCAAAAAAAAAAAAAAAAAAAAAAAAAAAAAAAAAGCAUAUGACUAAAAAAAAAAAAAAAAAAAAAAA
1440    AAAAAAAAAAAAAAAAAAAAAAAAAAAAAAAAAAAAAAAAAAAAA
```

```
1       .((.(((.(((((...((((....)))).................................................((((((((((((..(((..(((.(((....((........))....))).))).)))..))))..............
160     .(((((((.(((..........)))))))))).)).................(((((....))))).((((.((((((((((...))))))).))))))))...................................((((......(((...)
320     ........))))......(((((.((.((((((((...(((((....((((.((((...(((..((((.((.((.((((.(((..(((((...(((((((((......(((((.......))))).(((((.(((..(((...(((((......)))))
480     )).(((((.((........)).)))))).....))).))))))..(((.((((((........)))))).)))((((((((((((....)))).))))))))...................................)))).....))))))).....(((((((.
640     (.....((.(((((.......(((((......))))).......((((((.....(((((((((.......))))))..........((((((..........((((((.(((........)))(((((((..(((((((.((((..((((..(((((
800     .)))))((((....))))....(((((((........)))..)))).(((((.....)))))))))...)))).)))..((.((((.(.....)..)))).))..((((..(((.(((((........)))))))))))..)))).).))))))).)))
960     )....))))))....))).....)))))))....))))))).))).))))))))....((((...))))....)))))...))).)))).))..)))))))))))))).)))).)))))......((((((((.(((.(((..((((...((((..
1120    ))))............(((((((......)))))))..........((((.((((((((...(((((((((.((..........(((((((....)))))))......(((....)))..........................))))))))))
1280    )))).))))))))((((...))))......)))).)))).))).))))))))).)))))))).)).)))))................)).)))))))..............................)))))).))).))..........................
1440    ............................................
```

You can download the minimum free energy (MFE) structure in [Vienna Format| Ct Format]. You can get thermodynamic details on this structure by submitting to our RNAeval web server.

Graphical output

You may look at the interactive drawing of the MFE structure below. If you do not see the interactive drawing and you are using Internet Explorer, please install the Adobe SVG plugin. **A note on base-pairing probabilities:** The structure below is colored by base-pairing probabilities. For unpaired regions the color denotes the probability of being unpaired.

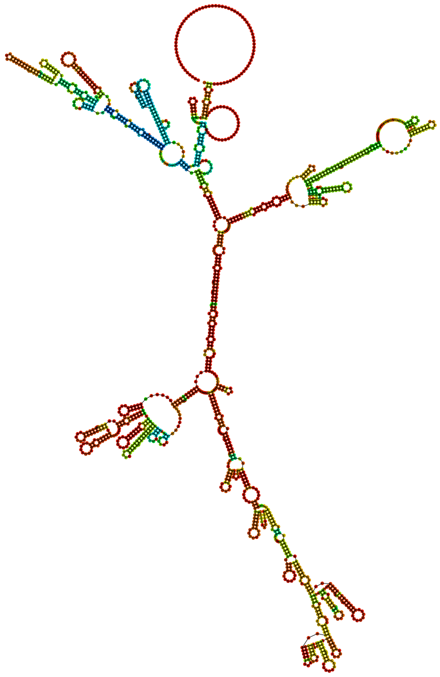

Sequence display options

MFE secondary structure

- ◉ Plain Sequence
- ○ No Sequence

Other display options

- ◉ Base-pair probabilities
- ○ Positional entropy
- ○ None

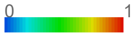

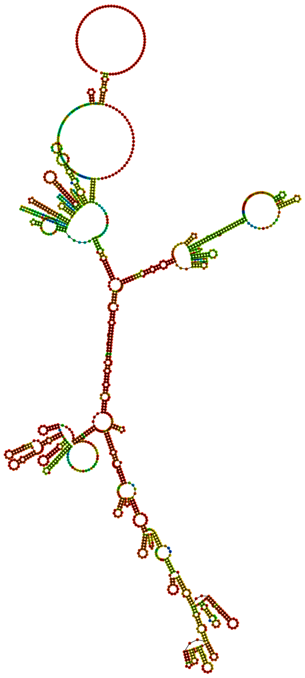

Sequence display options

Centroid secondary structure

- ◉ Plain Sequence
- ○ No Sequence

Other display options

- ◉ Base-pair probabilities
- ○ Positional entropy
- ○ None

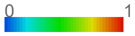

Image description	Download options
MFE plain structure drawing	[EPS | PDF | IMAGE CONVERTER | VIEW IN FORNA]
Centroid plain structure drawing	[EPS | PDF | IMAGE CONVERTER | VIEW IN FORNA]
MFE structure drawing encoding base-pair probabilities	[EPS | PDF | IMAGE CONVERTER | VIEW IN FORNA]
Centroid structure drawing encoding base-pair probabilities	[EPS | PDF | IMAGE CONVERTER | VIEW IN FORNA]
MFE structure drawing encoding positional entropy	[EPS | PDF | IMAGE CONVERTER | VIEW IN FORNA]
Centroid structure drawing encoding positional entropy	[EPS | PDF | IMAGE CONVERTER | VIEW IN FORNA]

Here you find a mountain plot representation of the MFE structure, the thermodynamic ensemble of RNA structures, and the centroid structure. Additionally we present the positional entropy for each position. Download as [EPS|PDF|IMAGE CONVERTER].

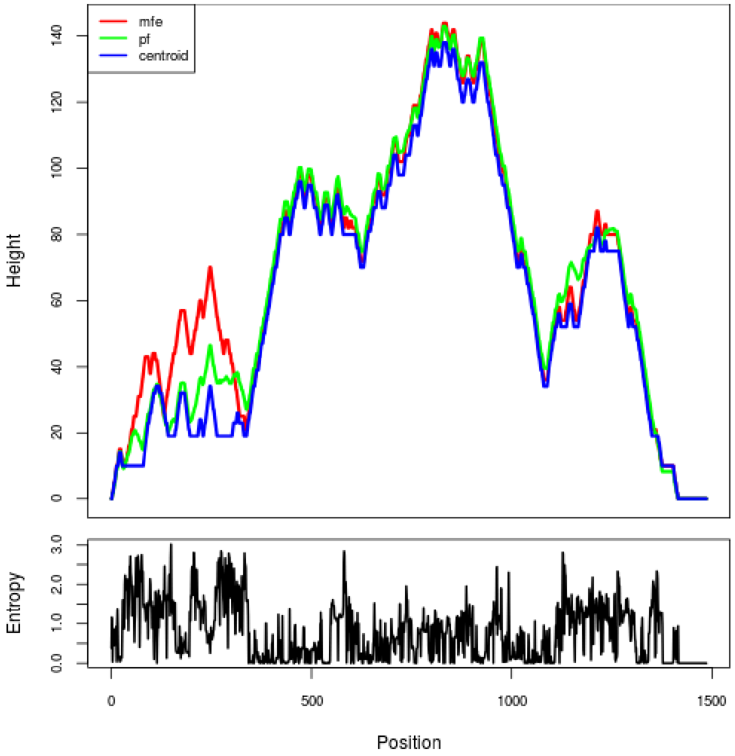

Results have been computed using RNAfold 2.6.3. An equivalent command line call would have been

```
RNAfold -p -d2 --noLP < sequence1.fa > sequence1.out
```

RNA parameters are described in

Mathews DH, Disney MD, Childs JL, Schroeder SJ, Zuker M, Turner DH. (2004) Incorporating chemical modification constraints into a dynamic programming algorithm for prediction of RNA secondary structure. *Proc Natl Acad Sci U S A* 101(19):7287-92.

If you find these results helpful for your work you may want to cite:

OPEN ACCESS OXFORD JOURNALS Gruber AR, Lorenz R, Bernhart SH, Neuböck R, Hofacker IL. **The Vienna RNA Websuite.** Nucleic Acids Research, Volume 36, Issue suppl_2, 1 July 2008, Pages W70-W74, DOI: 10.1093/nar/gkn188

Lorenz, R. and Bernhart, S.H. and Höner zu Siederdissen, C. and Tafer, H. and Flamm, C. and Stadler, P.F. and Hofacker, I.L. "ViennaRNA Package 2.0", Algorithms for Molecular Biology, 6:1 page(s): 26, 2011

RNAfold WebServer

1 Enter Input Parameters 2 View Results

[Home|New job|Help]

Results for minimum free energy prediction

The optimal secondary structure in dot-bracket notation with a minimum free energy of **-511.10** kcal/mol is given below.

[color by base-pairing probability | color by positional entropy | no coloring]

```
1      GAGACAUUUGCUUCUGACACAACUGUGUUCACUAGCAACCUCAAACAGACACCGCCACCAUGUUCGUGUUCCUGGUGCUGCUGCCCCUGGUGAGCAGCCAGUGCGUGAUGGCCAAGCUGAGCACCGACGAGCUGCUGGACGCCUUCAAGGAGAUGACC
160    GCUGGAACUGAGCGACUUUGUGAAGAAAUUCGAGGAAACCUUCGAGGUGACCGCCGCCGCCCCUGUGGCCGUGGCCGCCGCAGGUGCCGCCCCUGCCGGAGCCGCUGUCGAGGCUGCUGAGGAGCAAAGCGAGUUCGACGUGAUCCUGGAGGCCGCCG
320    GAUAAGAAGAUCGGCGUGAUUAAGGUGGUGCGGGAAAUCGUGUCCGGCCUGGGACUGAAGGAAGCUAAGGACCUGGUGGACGGCGCUCCCAAGCCUCUGCUCGAGAAGGUGGCUAAGGAAGCCGCAGAUGAGGCUAAAGCCAAGCUGGAAGCUGCCGG
480    CCACAGUGACAGUCAAGGAGGCCGCCGCUAAAGGCAUCAUCAACACCUUGCAGAAGUACUACUGCAGAGUGCGGGGCGGCCGGUGCGCCGUCCUGUCCUGCCUGCCUAAGGAAGAGCAGAUCGGCAAGUGUAGCACAAGAGGCAGAAAGUGCUGUAGA
640    GAAGAAGGAAGCCGCCGCCAAGACCCUGGGCCUGCUGCUGGCUUAUCGCAAAAAAUUCAUCGACCUGAACAUCACCAUGCUGAAGAAGAAGGAAGUGGACCUGGAUUUCGGCCUCAAAAAGAGAACCGCCCCCAGAAGCCUGUCUCUGAAGAAAACCA
800    AUGGUGCUGCGGAAGCGGAAUAAGGCCAGAUACAGCCCUGCCGCCUACGCCUACAGACGGAGAUUCCCCGCUGUUAUCACCCGGGUGCUGCCUGCCGCCUAUGCCGUGGAUUUUAUCUGGACAGGCAACCAGAGAACAGCCCCUAGAGCCGCCUACCG
960    CCGGCAGAUUCCACUGGGAGAGAUUUUCUAAUGCCAGCCCUGCCGCUUACAACAAGCAGAGCACAAGACCUACCGGCGCCUGCGUGUACCUGGAACCUGGCCCCGGCCCAGGAACCAUGACAAAGUGGCAGGAGGUGGACGAGAUGCUGAGAGCCGAG
1120   CGGCCCUGGCCCAGGAGGAAGAGUGGUGUUCCUGCCCACCAUCCGGAGACAGCUGGCCCUGGCCGAGGCCGCCGCCAAAGCCAAGUUCGUGGCCGCUUGGACCCUGAAAGCCGCAGCCCACCACCACCAUCACCACUGAUAAUAGGCUGGAGCCUCGG
1280   GCCUAGCUUCUUGCCCCUUGGGCCUCCCCCCAGCCCCUCCUCCCCUUCCUGCACCCGUACCCCCGUGGUCUUUGAAUAAAGUCUGAGUGGGCGGCAAAAAAAAAAAAAAAAAAAAAAAAAAAAAAAAAGCAUAUGACUAAAAAAAAAAAAAAAAAAAAAAA
1440   AAAAAAAAAAAAAAAAAAAAAAAAAAAAAAAAAAAAAAAAAAAAAA
```

```
1      .((.(((.(((((..(((((....))))).....((..((.((..(((((...(((((..(((.((((((((((.((((..(((...(((.((((((..((..(((.((((...))))..)))..))..))))))(((((((((((.((((......
160    (((.......)))).)))).)))))....((((((....))))))((((.((((.(((((....))))).)))).))))((((((((((..(((.(((((..(((.(((((((((((((....)))..)))...))))))))).))).)))))..((.(
320    .((.((((((((((((.....((((((((.(((.....(((((.(((((((..((..((....)).))..))((((((.((((..(((...((((((((((......((((((.....)))))).((((((((....)))..(((((......))))
480    ((....(((...)))....))).((......))).)))))..(((.((((((........)))))).)))(((((((((......)))))))))((((.........)))).)))))))..)))....((((((............))))))....
640    ......))).))))))))))......((((((.......))))))..........((((......))))...((((((((..(((.(((((...(((...(((.((((...........)))).)))...)))....))).)))))..)).......
800    ))))))((((....))))....))))).)))))..))))))))))))))).((((.(((........))))))......((.((((....(((((((((.....((((.(((((((((((........))))).........))))))))).....)
960    .))))))....)))).))...)))))))).))))).))..(((.((((.....))))...)))..........)))))))))))))).))....))).)))..))))))))))).)))))))..)))))((((((.(((.(((..((((((.((((..
1120   ))))).))((((((((.((((((((((((...((((...(((..(((..(((((((((..((((((....))..))))..))).)))).))..)))..)))...........))))..))))))....((((....))))....((((((.(((....
1280   ))))))))))))).)).)))))))).....)))))..))).))).)))))))..(((((......)).)))...........)))))..))))..))...........................))))).))).))......................
1440   ............................................
```

You can download the minimum free energy (MFE) structure in [Vienna Format| Ct Format]. You can get thermodynamic details on this structure by submitting to our RNAeval web server.

Results for thermodynamic ensemble prediction

The free energy of the thermodynamic ensemble is **-531.60** kcal/mol.

The frequency of the MFE structure in the ensemble is **0.00** %.

The ensemble diversity is **397.53**.

You may look at the **dot plot** containing the base pair probabilities [EPS|PDF|IMAGE CONVERTER].

The centroid secondary structure in dot-bracket notation with a minimum free energy of **-402.10** kcal/mol is given below.

[color by base-pairing probability | color by positional entropy | no coloring]

```
1      GAGACAUUUGCUUCUGACACAACUGUGUUCACUAGCAACCUCAAACAGACACCGCCACCAUGUUCGUGUUCCUGGUGCUGCUGCCCCUGGUGAGCAGCCAGUGCGUGAUGGCCAAGCUGAGCACCGACGAGCUGCUGGACGCCUUCAAGGAGAUGACC
160    GCUGGAACUGAGCGACUUUGUGAAGAAAUUCGAGGAAACCUUCGAGGUGACCGCCGCCGCCCCUGUGGCCGUGGCCGCCGCAGGUGCCGCCCCUGCCGGAGCCGCUGUCGAGGCUGCUGAGGAGCAAAGCGAGUUCGACGUGAUCCUGGAGGCCGCCG
320    GAUAAGAAGAUCGGCGUGAUUAAGGUGGUGCGGGAAAUCGUGUCCGGCCUGGGACUGAAGGAAGCUAAGGACCUGGUGGACGGCGCUCCCAAGCCUCUGCUCGAGAAGGUGGCUAAGGAAGCCGCAGAUGAGGCUAAAGCCAAGCUGGAAGCUGCCGG
480    CCACAGUGACAGUCAAGGAGGCCGCCGCUAAAGGCAUCAUCAACACCUUGCAGAAGUACUACUGCAGAGUGCGGGGCGGCCGGUGCGCCGUCCUGUCCUGCCUGCCUAAGGAAGAGCAGAUCGGCAAGUGUAGCACAAGAGGCAGAAAGUGCUGUAGA
640    GAAGAAGGAAGCCGCCGCCAAGACCCUGGGCCUGCUGCUGGCUUAUCGCAAAAAAUUCAUCGACCUGAACAUCACCAUGCUGAAGAAGAAGGAAGUGGACCUGGAUUUCGGCCUCAAAAAGAGAACCGCCCCCAGAAGCCUGUCUCUGAAGAAAACCA
800    AUGGUGCUGCGGAAGCGGAAUAAGGCCAGAUACAGCCCUGCCGCCUACGCCUACAGACGGAGAUUCCCCGCUGUUAUCACCCGGGUGCUGCCUGCCGCCUAUGCCGUGGAUUUUAUCUGGACAGGCAACCAGAGAACAGCCCCUAGAGCCGCCUACCG
960    CCGGCAGAUUCCACUGGGAGAGAUUUUCUAAUGCCAGCCCUGCCGCUUACAACAAGCAGAGCACAAGACCUACCGGCGCCUGCGUGUACCUGGAACCUGGCCCCGGCCCAGGAACCAUGACAAAGUGGCAGGAGGUGGACGAGAUGCUGAGAGCCGAG
1120   CGGCCCUGGCCCAGGAGGAAGAGUGGUGUUCCUGCCCACCAUCCGGAGACAGCUGGCCCUGGCCGAGGCCGCCGCCAAAGCCAAGUUCGUGGCCGCUUGGACCCUGAAAGCCGCAGCCCACCACCACCAUCACCACUGAUAAUAGGCUGGAGCCUCGG
1280   GCCUAGCUUCUUGCCCCUUGGGCCUCCCCCCAGCCCCUCCUCCCCUUCCUGCACCCGUACCCCCGUGGUCUUUGAAUAAAGUCUGAGUGGGCGGCAAAAAAAAAAAAAAAAAAAAAAAAAAAAAAAAAGCAUAUGACUAAAAAAAAAAAAAAAAAAAAAAA
1440   AAAAAAAAAAAAAAAAAAAAAAAAAAAAAAAAAAAAAAAAAAAAAA
```

```
1      .((.(((.(((((..(((((....)))).)....((..((.((..(((((.....(((..((..(((.(((((((.((((..(((........(((((..(....(....((...))......)....)..)))))......................
160    ...........................((((((....))))))((((.((((.(((((....))))).)))).))))((((((((((.......(((((..(((.((((((...(((.........)))...))))))))).))).)).....((.(
320    (...((((((((((((.....((((((((.(((.....(((((.(((((((..((..((....)).))..))((((((.((((..(((...((((((((((......((((((.....)))))).((((.(((....)))..(((((......))))
480    ((....((.....))....))).(((......)))..)))))..(((.((((((........)))))).)))((((((((((......))))))))))...................)))))))..)))....((((((............))))))....
640    ......))).))))))))))................................((((......))))...((((((((................................(((.....)))..................................
800    ))))))((((....))))....))))).)))))..))))))))))))))).((((.(((........))))))...(.(((..(((....(((((.(((.....((((.(....(((((........)))))..............)))))).....)
960    ..)))))....))).))).).))))))))..)))).)).......(((.......)))..................)))))))))...............)))..))))))))))))..))).))..)))((((((((.(((.(((..((((((.((((..
1120   ))))).))((((((((.(((((...((((........))))..........(((((((...((((((((...........................................................((((....))))...........))))))
1280   ))).)))).))).)).))))))))).....)))))..))).))).))))))))((((((......)).)))...........)))))..))))..))...........................))))).))).))......................
1440   ............................................
```

You can download the minimum free energy (MFE) structure in [Vienna Format| Ct Format]. You can get thermodynamic details on this structure by submitting to our RNAeval web server.

Graphical output

You may look at the interactive drawing of the MFE structure below. If you do not see the interactive drawing and you are using Internet Explorer, please install the Adobe SVG plugin. **A note on base-pairing probabilities:** The structure below is colored by base-pairing probabilities. For unpaired regions the color denotes the probability of being unpaired.

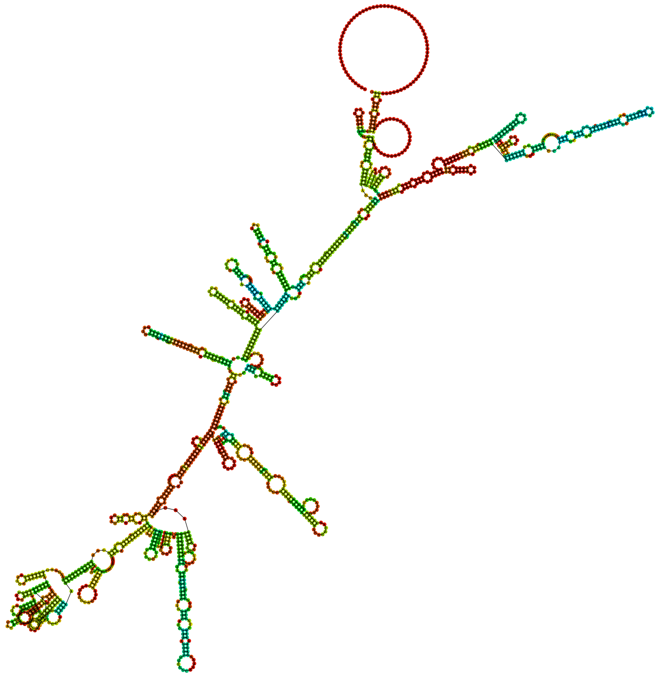

MFE secondary structure

Sequence display options

- (•) Plain Sequence
- ( ) No Sequence

Other display options

- (•) Base-pair probabilities
- ( ) Positional entropy
- ( ) None

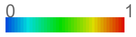

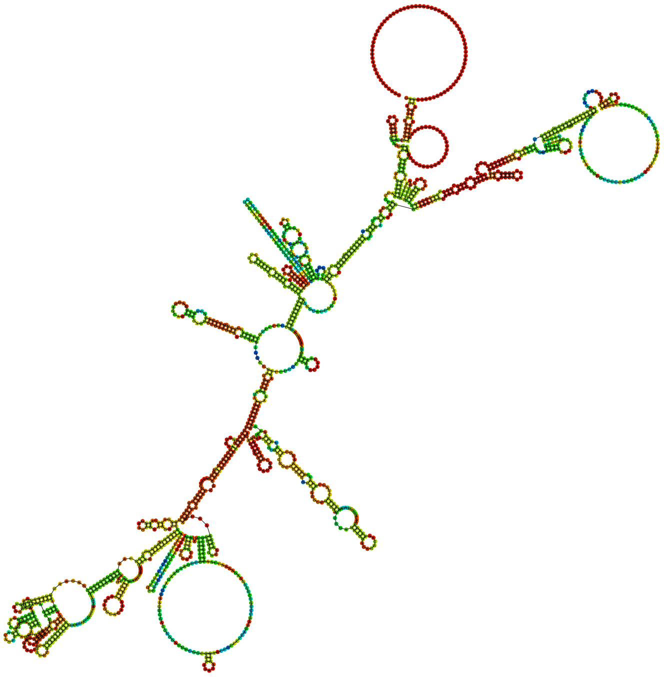

Centroid secondary structure

Sequence display options

- (•) Plain Sequence
- ( ) No Sequence

Other display options

- (•) Base-pair probabilities
- ( ) Positional entropy
- ( ) None

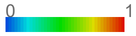

Image description	Download options
MFE plain structure drawing	[EPS | PDF | IMAGE CONVERTER | VIEW IN FORNA]
Centroid plain structure drawing	[EPS | PDF | IMAGE CONVERTER | VIEW IN FORNA]
MFE structure drawing encoding base-pair probabilities	[EPS | PDF | IMAGE CONVERTER | VIEW IN FORNA]
Centroid structure drawing encoding base-pair probabilities	[EPS | PDF | IMAGE CONVERTER | VIEW IN FORNA]
MFE structure drawing encoding positional entropy	[EPS | PDF | IMAGE CONVERTER | VIEW IN FORNA]
Centroid structure drawing encoding positional entropy	[EPS | PDF | IMAGE CONVERTER | VIEW IN FORNA]

Here you find a mountain plot representation of the MFE structure, the thermodynamic ensemble of RNA structures, and the centroid structure. Additionally we present the positional entropy for each position. Download as [EPS|PDF|IMAGE CONVERTER].

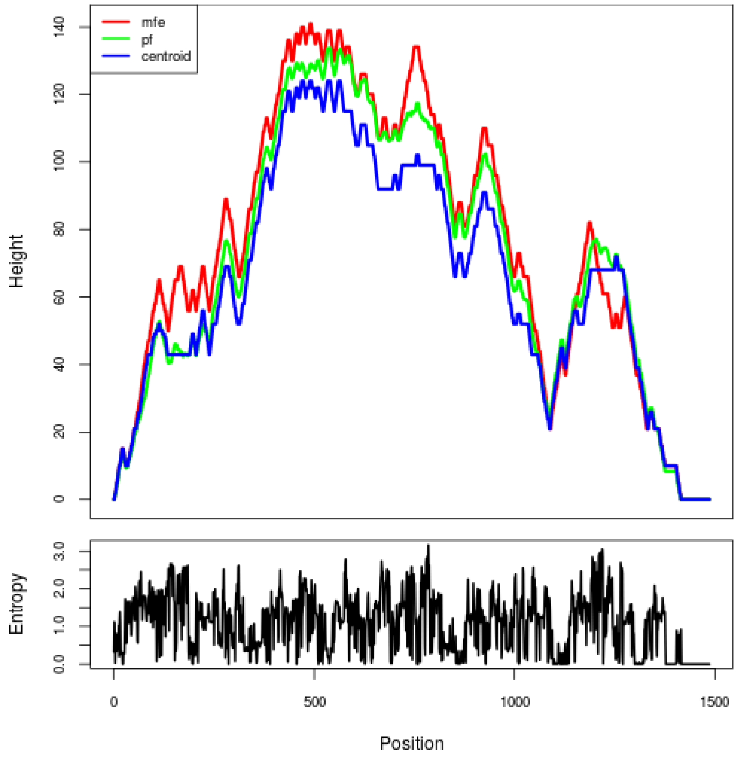

Results have been computed using RNAfold 2.6.3. An equivalent command line call would have been

`RNAfold -p -d2 --noLP < sequence1.fa > sequence1.out`

RNA parameters are described in

Mathews DH, Disney MD, Childs JL, Schroeder SJ, Zuker M, Turner DH. (2004) Incorporating chemical modification constraints into a dynamic programming algorithm for prediction of RNA secondary structure. *Proc Natl Acad Sci U S A* 101(19):7287-92.

If you find these results helpful for your work you may want to cite:

Gruber AR, Lorenz R, Bernhart SH, Neuböck R, Hofacker IL.
The Vienna RNA Websuite. Nucleic Acids Research, Volume 36, Issue suppl_2, 1 July 2008, Pages W70-W74, DOI: 10.1093/nar/gkn188

Lorenz, R. and Bernhart, S.H. and Höner zu Siederdissen, C. and Tafer, H. and Flamm, C. and Stadler, P.F. and Hofacker, I.L. "ViennaRNA Package 2.0", Algorithms for Molecular Biology, 6:1 page(s): 26, 2011

Institute for Theoretical Chemistry | University of Vienna | rna@tbi.univie.ac.at

RNAfold WebServer

1 Enter Input Parameters 2 View Results

[Home|New job|Help]

Results for minimum free energy prediction

The optimal secondary structure in dot-bracket notation with a minimum free energy of **-542.30** kcal/mol is given below.

[color by base-pairing probability | color by positional entropy | no coloring]

```
1       GAGACAUUUGCUUCUGACACAACUGUGUUCACUAGCAACCUCAAACAGACACCGCCACCAUGUUCGUGUUCCUGGUGCUGCUGCCCCUGGUGAGCAGCCAGUGCGUGAUGGCCAAGCUGAGCACUGAUGAGCUGCUGGACGCCUUCAAGGAGAUGACA
160     GCUCGAGCUGAGCGACUUCGUGAAAAAGUUCGAGGAAACCUUCGAGGUGACCGCCGCCGCUCCCGUGGCCGUGGCAGCCGCUGGCGCCGCCCCUGCCGGAGCCGCUGUCGAGGCCGCCGAAGAGCAGUCCGAGUUCGACGUGAUCCUGGAAGCCGCCG
320     GACAAGAAAAUCGGCGUGAUUAAGGUCGUCCGGGAAAUCGUGUCCGGCCUGGGACUGAAGGAAGCAAAGGACCUGGUGGACGGCGCCCCAAAGCCUCUGCUGGAGAAGGUGGCCAAAGAGGCCGCCGACGAGGCCAAAGCCAAGCUGGAGGCUGCCGG
480     CCACCGUGACAGUGAAGGAAGCCGCCGCGAAGGGCAUCAUCAACACCCUGCAAAAGUACUACUGCAGAGUGCGGGGCGGACGCUGCGCCGUGCUGUCUUGUCUGCCUAAGGAAGAGCAGAUCGGCAAGUGUAGCACAAGAGGCAGAAAGUGCUGCAGA
640     AAAGAAAGAAGCCGCCGCUAAGUUCAUCGACCUGAAUAUCACCAUGCUGAAGAAGACACUGGGCCUGCUGCUGGCCUACCGGAAGAAGCGGACAGCCCCGCGGAGCCUGUCUCUGAAGAAGAAAGAAGUGGAUCUGGAUUUUGGCCUCAAAAAGACCA
800     AUGGUGCUGAGAAAGAGAAACAAGGCCAGAUACAGCCCUGCUGCCUAUGCCGUGGACUUCAUCUGGACCGGCAAUCAGAGAACCGCCCCUAGAGCCGCUUAUAGGGCCGGCAGAUUUCACUGGGAGAGAUUCAGCAACGCCAGCCCCGCUGCCUACGC
960     ACAGACGGCGGUUCCCCGCCGUUAUCACAAGAGUGCUGCCCGCCGCUUACAACAAGCAGAGCACCAGACCUACCGGCGCAUGCGUGUACCUGGAACCCGGCCCUGGACCUGGGACCAUGACCAAGUGGCAGGAGGUGGAUGAGAUGCUGCGGGCCGAG
1120    CGGCCCAGGCCCUGGAGGCAGAGUGGUGUUCCUGCCUACAAUCAGAAGGCAGCUGGCCCUGGCUGAGGCCGCCGCUAAGGCCAAAUUUGUGGCCGCCUGGACCCUGAAGGCCGCUGCCCACCAUCACCACCACCACUGAUAAUAGGCUGGAGCCUCGG
1280    GCCUAGCUUCUUGCCCCUUGGGCCUCCCCCCAGCCCCUCCUCCCCUUCCUGCACCCGUACCCCCGUGGUCUUUGAAUAAAGUCUGAGUGGGCGGCAAAAAAAAAAAAAAAAAAAAAAAAAAAAAAAAAGCAUAUGACUAAAAAAAAAAAAAAAAAAAAAA
1440    AAAAAAAAAAAAAAAAAAAAAAAAAAAAAAAAAAAAAAAAAAAA

1       .((.(((.(((((..(((((....))))).....((..((.((..(((((.....((((((((((.((..((((((((.((((.....((((((.((((((((.....(((...)))..))))))..((((.(((((.((((((.((((........
160     .((((((((..(((....))).....)))))))).)).)))).)))))).)))..)).))))..((((.((((((((.((((((((((((((((((...((((((((((.((((((......(((((.((.((((((((((((((((.(((..((
320     ((........))).)).......)))..)))))))..)))))).))).)).)))))).(((..........))))))))..((((((....((((((((((......((((((.....))))))).....(((....))).))).))))))))...))
480     )).)).))))))))..((......))))....))))......(((.(((((..........))))).))).)))))))..(((((((..(((((....((((((((.....)).))))))))))).))))))).......(((......)))...
640     .........(((....)))..(((((......)))))......((((((.......((((.....(((((((((((.((.....((((......))))....)).)).(((.....)))...(((((.(((((..((((((((.....((((((
800     .)))).)).............)))))))..((((...))))........((((..(((.(((((........))))))))))))..))))).))))))...))))))))))......))))......)))))))).)))))))...)))))).))).
960     )).((((((((....))))))))..(((....)))......)).))))))......))))))))))))....))))).))))).)))(((.(((.....(((((.......))).)).....))).)))((((((((.(((.(((..((((.((((((..
1120    ))))))((((((.((.((((((((((((...(((........)))..((((((.((((..((...((((.(((((.(((......)))))))).))))....))....))))))))))...........)))))))......(((((((.(((....
1280    ))))))))).)))))))..))))))......))))..))).))).))))))))((((((......)).)))...........)))))..))))..))............................))))).))).)).....................
1440    ............................................
```

You can download the minimum free energy (MFE) structure in [Vienna Format| Ct Format]. You can get thermodynamic details on this structure by submitting to our RNAeval web server.

Results for thermodynamic ensemble prediction

The free energy of the thermodynamic ensemble is **-560.79** kcal/mol.

The frequency of the MFE structure in the ensemble is **0.00** %.

The ensemble diversity is **306.34**.

You may look at the **dot plot** containing the base pair probabilities [EPS|PDF|IMAGE CONVERTER].

The centroid secondary structure in dot-bracket notation with a minimum free energy of **-450.54** kcal/mol is given below.

[color by base-pairing probability | color by positional entropy | no coloring]

```
1       GAGACAUUUGCUUCUGACACAACUGUGUUCACUAGCAACCUCAAACAGACACCGCCACCAUGUUCGUGUUCCUGGUGCUGCUGCCCCUGGUGAGCAGCCAGUGCGUGAUGGCCAAGCUGAGCACUGAUGAGCUGCUGGACGCCUUCAAGGAGAUGACA
160     GCUCGAGCUGAGCGACUUCGUGAAAAAGUUCGAGGAAACCUUCGAGGUGACCGCCGCCGCUCCCGUGGCCGUGGCAGCCGCUGGCGCCGCCCCUGCCGGAGCCGCUGUCGAGGCCGCCGAAGAGCAGUCCGAGUUCGACGUGAUCCUGGAAGCCGCCG
320     GACAAGAAAAUCGGCGUGAUUAAGGUCGUCCGGGAAAUCGUGUCCGGCCUGGGACUGAAGGAAGCAAAGGACCUGGUGGACGGCGCCCCAAAGCCUCUGCUGGAGAAGGUGGCCAAAGAGGCCGCCGACGAGGCCAAAGCCAAGCUGGAGGCUGCCGG
480     CCACCGUGACAGUGAAGGAAGCCGCCGCGAAGGGCAUCAUCAACACCCUGCAAAAGUACUACUGCAGAGUGCGGGGCGGACGCUGCGCCGUGCUGUCUUGUCUGCCUAAGGAAGAGCAGAUCGGCAAGUGUAGCACAAGAGGCAGAAAGUGCUGCAGA
640     AAAGAAAGAAGCCGCCGCUAAGUUCAUCGACCUGAAUAUCACCAUGCUGAAGAAGACACUGGGCCUGCUGCUGGCCUACCGGAAGAAGCGGACAGCCCCGCGGAGCCUGUCUCUGAAGAAGAAAGAAGUGGAUCUGGAUUUUGGCCUCAAAAAGACCA
800     AUGGUGCUGAGAAAGAGAAACAAGGCCAGAUACAGCCCUGCUGCCUAUGCCGUGGACUUCAUCUGGACCGGCAAUCAGAGAACCGCCCCUAGAGCCGCUUAUAGGGCCGGCAGAUUUCACUGGGAGAGAUUCAGCAACGCCAGCCCCGCUGCCUACGC
960     ACAGACGGCGGUUCCCCGCCGUUAUCACAAGAGUGCUGCCCGCCGCUUACAACAAGCAGAGCACCAGACCUACCGGCGCAUGCGUGUACCUGGAACCCGGCCCUGGACCUGGGACCAUGACCAAGUGGCAGGAGGUGGAUGAGAUGCUGCGGGCCGAG
1120    CGGCCCAGGCCCUGGAGGCAGAGUGGUGUUCCUGCCUACAAUCAGAAGGCAGCUGGCCCUGGCUGAGGCCGCCGCUAAGGCCAAAUUUGUGGCCGCCUGGACCCUGAAGGCCGCUGCCCACCAUCACCACCACCACUGAUAAUAGGCUGGAGCCUCGG
1280    GCCUAGCUUCUUGCCCCUUGGGCCUCCCCCCAGCCCCUCCUCCCCUUCCUGCACCCGUACCCCCGUGGUCUUUGAAUAAAGUCUGAGUGGGCGGCAAAAAAAAAAAAAAAAAAAAAAAAAAAAAAAAAGCAUAUGACUAAAAAAAAAAAAAAAAAAAAAA
1440    AAAAAAAAAAAAAAAAAAAAAAAAAAAAAAAAAAAAAAAAAAAA

1       .((.(((.(((((..(((((....)))).)....((..((.((..(((((.....((((((((((.((..((((((((.((((.....((((((.((((((((.....(((...)))..))))))..((((.((.((.((((((.((((........
160     .((((((((..(((....))).....)))))))).)).)))).)))))).))...)).))))..((((.((((((((.(((((((((((((.........(((((((.((((((......(((((.((.(((((((((((((((.(((((((
320     ...........))))........)))..)))))))..))))))).)).)).)))))).(((..........))))))))..((((((....((((((((((......(((((((.....)))))))......(((....))).))).))))))))...))
480     )).)).))))))))..........................(((.(((((..........))))).))).))))))))..(((((((..(((((.............................))))).)))))))..........................
640     .........(((....)))..((((........))))........((((((................(((((((((......................................................................................((((((
800     .)))).)).....................................(((.................)))..................................)))))))))................))))))..)))))))...)))))).))).
960     )).((((((((....))))))))..................)).))))))......)))))))))))).....)))))).))))).)))......................................((((((((.(((.(((..((((.((((((..
1120    ))))))((((((.((.((((((((((((...(((........)))..((((((.((((..(((..((((.(((((.............))))).)))).).)).....))))))))))...........)))))))......(((((((.(((....
1280    ))))))))).)))))))..))))))......))))..))).))).))))))))...........................)))))..))))..))............................))))).))).))..........................
1440    ............................................
```

You can download the minimum free energy (MFE) structure in [Vienna Format| Ct Format]. You can get thermodynamic details on this structure by submitting to our RNAeval web server.

Graphical output

You may look at the interactive drawing of the MFE structure below. If you do not see the interactive drawing and you are using Internet Explorer, please install the Adobe SVG plugin. **A note on base-pairing probabilities:** The structure below is colored by base-pairing probabilities. For unpaired regions the color denotes the probability of being unpaired.

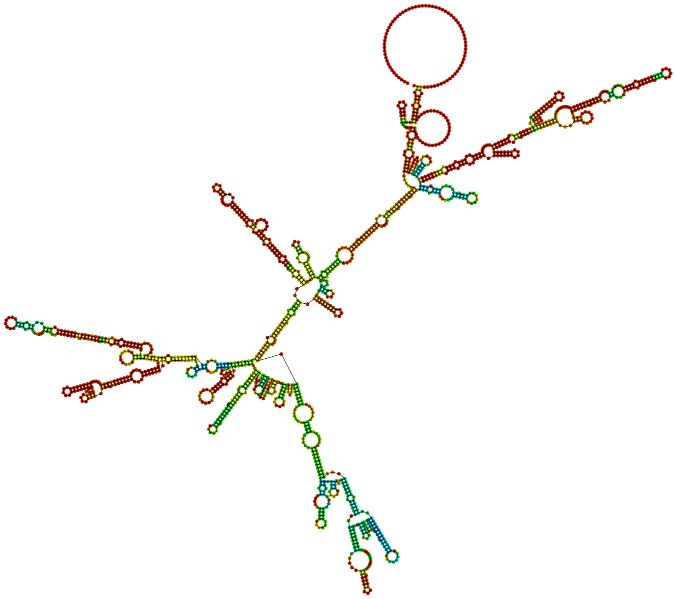

Sequence display options

◉ Plain Sequence
○ No Sequence

MFE secondary structure

Other display options

◉ Base-pair probabilities
○ Positional entropy
○ None

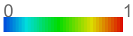

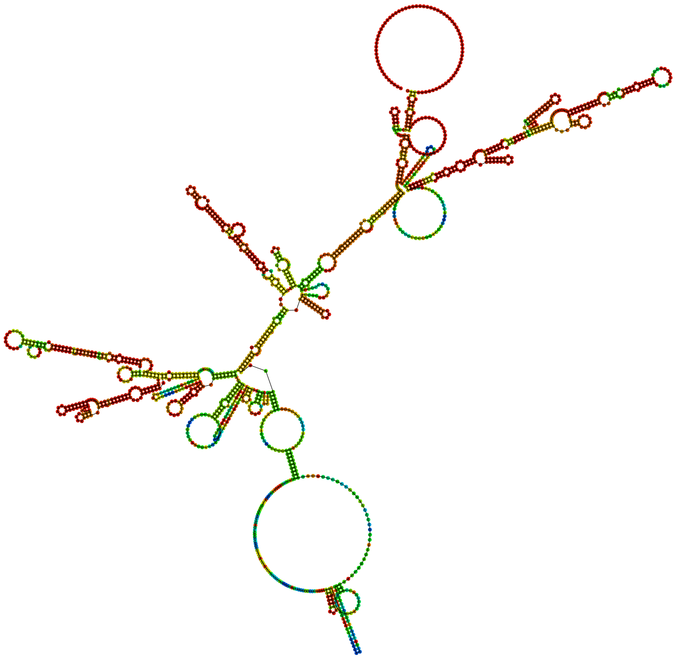

Sequence display options

◉ Plain Sequence
○ No Sequence

Centroid secondary structure

Other display options

◉ Base-pair probabilities
○ Positional entropy
○ None

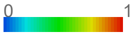

Image description	Download options
MFE plain structure drawing	[EPS | PDF | IMAGE CONVERTER | VIEW IN FORNA]
Centroid plain structure drawing	[EPS | PDF | IMAGE CONVERTER | VIEW IN FORNA]
MFE structure drawing encoding base-pair probabilities	[EPS | PDF | IMAGE CONVERTER | VIEW IN FORNA]
Centroid structure drawing encoding base-pair probabilities	[EPS | PDF | IMAGE CONVERTER | VIEW IN FORNA]
MFE structure drawing encoding positional entropy	[EPS | PDF | IMAGE CONVERTER | VIEW IN FORNA]
Centroid structure drawing encoding positional entropy	[EPS | PDF | IMAGE CONVERTER | VIEW IN FORNA]

Here you find a mountain plot representation of the MFE structure, the thermodynamic ensemble of RNA structures, and the centroid structure. Additionally we present the positional entropy for each position. Download as [EPS|PDF|IMAGE CONVERTER].

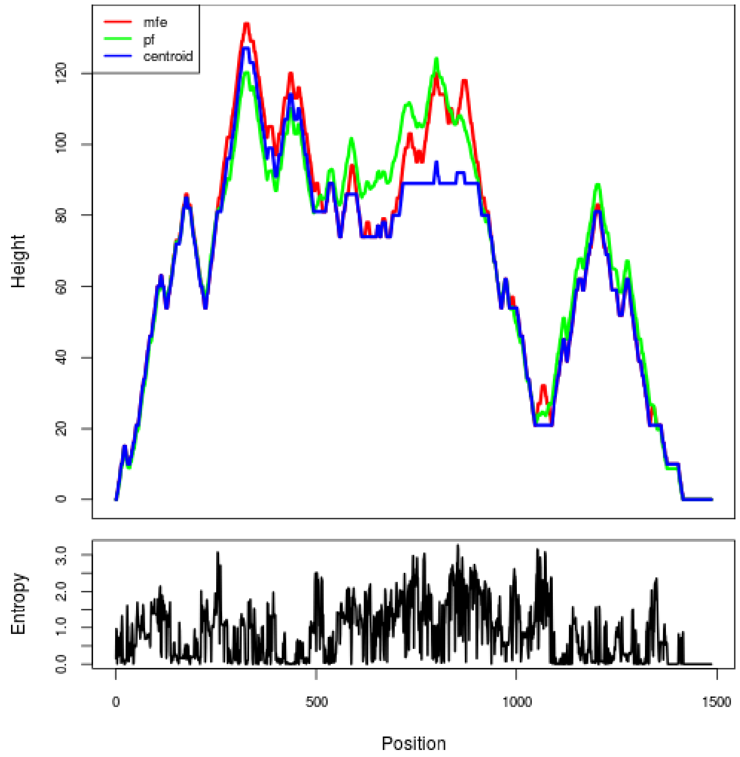

Results have been computed using RNAfold 2.6.3. An equivalent command line call would have been

`RNAfold -p -d2 --noLP < sequence1.fa > sequence1.out`

RNA parameters are described in

Mathews DH, Disney MD, Childs JL, Schroeder SJ, Zuker M, Turner DH. (2004) Incorporating chemical modification constraints into a dynamic programming algorithm for prediction of RNA secondary structure. *Proc Natl Acad Sci U S A* 101(19):7287-92.

If you find these results helpful for your work you may want to cite:

OPEN ACCESS OXFORD JOURNALS Gruber AR, Lorenz R, Bernhart SH, Neuböck R, Hofacker IL. **The Vienna RNA Websuite.** Nucleic Acids Research, Volume 36, Issue suppl_2, 1 July 2008, Pages W70-W74, DOI: 10.1093/nar/gkn188

Lorenz, R. and Bernhart, S.H. and Höner zu Siederdissen, C. and Tafer, H. and Flamm, C. and Stadler, P.F. and Hofacker, I.L. "ViennaRNA Package 2.0", Algorithms for Molecular Biology, 6:1 page(s): 26, 2011

Institute for Theoretical Chemistry | University of Vienna | rna@tbi.univie.ac.at

RNAfold WebServer

1 Enter Input Parameters 2 View Results

[Home|New job|Help]

Results for minimum free energy prediction

The optimal secondary structure in dot-bracket notation with a minimum free energy of **-530.20** kcal/mol is given below.

[color by base-pairing probability | color by positional entropy | no coloring]

```
1     GAGACAUUUGCUUCUGACACAACUGUGUUCACUAGCAACCUCAAACAGACACCGCCACCAUGUUCGUGUUCCUGGUGCUGCUGCCCCUGGUGAGCAGCCAGUGCGUGAUGGCCAAGCUGUCCACAGACGAGCUGCUGGAUGCCUUCAAGGAAAUGACA
160   GCUCGAACUGAGCGACUUCGUGAAGAAGUUUGAGGAAACCUUCGAGGUGACCGCCGCUGCCCCUGUGGCCGUGGCCGCCGCCGGCGCCGCCCCUGCCGGCGCCGCCGUGGAAGCCGCUGAGGAACAGAGCGAGUUCGACGUGAUCCUGGAAGCCGCCCG
320   GAUAAGAAAAUCGGAGUGAUUAAGGUGGUGCGGGAAAUCGUGUCCGGCCUUGGACUGAAGGAGGCCAAGGACCUGGUGGACGGCGCUCCAAAGCCUCUGUUGGAGAAGGUCGCUAAGGAGGCUGCAGAUGAGGCCAAAGCCAAACUGGAGGCCGCCGG
480   CCACCGUGACCGUGAAGGAAGCUGCCGCCAAGGGCAUCAUCAACACCCUGCAGAAGUACUACUGCAGAGUGCGGGGUGGCAGAUGCGCCGUCCUGUCUUGUCUGCCUAAAGAAGAGCAAAUCGGCAAGUGCAGCACAAGAGGCAGAAAGUGCUGUAGA
640   GAAGAAGGAAGCCGCUGCCAAGACCCUGGGCCUGCUGCUCGCCUAUAGAAAGAAGUUCAUCGACCUGAACAUCACCAUGCUGAAGAAAAGAACCGCCCCUAGAAGCCUGAGCCUGAAAAAGAAAGAGGUGGACCUGGACUUCGGCCUGAAGAAAACCA
800   AUGGUGCUGAGAAAGCGGAACAAGGCUAGAUACAGCCCCGCUGCCUACGCCUACCGGAGACGGUUCCCCGCUGUCAUCACAAGAGUGCUGCCAGCCGCUUAUGCCGUGGACUUCAUCUGGACUGGCAACCAGAGAACAGCCCCCAGAGCCGCUUACAG
960   CCGGCAGAUUCCACUGGGAGCGGUUUAGCAAUGCCUCUCCAGCGGCCUACAACAAGCAGAGCACCCGGCCUACAGGCGCCUGCGUGUACCUGGAACCUGGCCCUGGACCUGGCACCAUGACCAAGUGGCAGGAGGUUGAUGAGAUGCUGCGGGCCGAG
1120  CGGCCCUGGACCCGGCGGCCGCGUUGUGUUCCUGCCCACCAUCAGAAGGCAGCUGGCCCUGGCCGAGGCUGCCGCUAAAGCCAAGUUUGUGGCCGCCUGGACACUGAAGGCCGCCGCCCACCACCACCAUCACCACUGAUAAUAGGCUGGAGCCUCGG
1280  GCCUAGCUUCUUGCCCCUUGGGCCUCCCCCCAGCCCCUCCUCCCCUUCCUGCACCCGUACCCCCGUGGUCUUUGAAUAAAGUCUGAGUGGGCGGCAAAAAAAAAAAAAAAAAAAAAAAAAAAAAAAGCAUAUGACUAAAAAAAAAAAAAAAAAAAAAA
1440  AAAAAAAAAAAAAAAAAAAAAAAAAAAAAAAAAAAAAAAAAAAAAA

1     (((....(((((..((((((....))).)))..))))).)))........((((((((((...(((.....((((((.(((((.(((((.((((((((((((((.(((......(((((((.....))).))))))).)))......)).))
160   )((((((((..(((....))).....))))))))....((((((.(((.((.(((((.(((.....))).)))))((.((((((((.......)))))))).)).(((.(((..((((.........))))..))).)))..(((((((......)))
320   )))....((((.....))))..((((((((.((..((((.((((((((((.........)))))).)))).))))...((((.(((((.(((((((.((((.((...)).)))))))))))........(((....)))....)))))))))).)
480   )))))))).))).)))))))..)))))))))).))))).))....(((.((((((........)))))).)))((((((((......))))))))).((((((((((.....)).)))....))))))((((((((............))))))))..
640   ((((.(((.((..((((..((((..(((((((.((((((((((((....((...((((((((((.....((..((.((((((...((..(((((.((......................)).)))))..))...))))))).))..))..((((
800   .))))..(((((.(((((....(((((((...((((...)))).))).))))((((....))))...))))).)).)))..((((.(((((.((((((((...(((((((..((.(((((...(((...........))).)))))(((((((...)
960   .)))).)).))))).)))))))))))..)))..))))))((((.((((..(((((.((((.((.((.........)).)))))))).)))...))......)))))))).((((.(((.........))).))))))))))))))...))..((((((..
1120  ))))))(((...((((((((.....(((((((((((..........))))).((((....))))((((.(((((.(((......)))))))).)))))))))....)))))))).)))........((((....)))).))))))..))))...))
1280  ))))))..))))((((...))))......))))..)))))...))))))))))..)))...))))))(((.((......)).)))..)))))..................................................................
1440  ..............................................
```

You can download the minimum free energy (MFE) structure in [Vienna Format| Ct Format]. You can get thermodynamic details on this structure by submitting to our RNAeval web server.

Results for thermodynamic ensemble prediction

The free energy of the thermodynamic ensemble is **-550.58** kcal/mol.

The frequency of the MFE structure in the ensemble is **0.00** %.

The ensemble diversity is **378.51**.

You may look at the **dot plot** containing the base pair probabilities [EPS|PDF|IMAGE CONVERTER].

The centroid secondary structure in dot-bracket notation with a minimum free energy of **-449.80** kcal/mol is given below.

[color by base-pairing probability | color by positional entropy | no coloring]


```
1     GAGACAUUUGCUUCUGACACAACUGUGUUCACUAGCAACCUCAAACAGACACCGCCACCAUGUUCGUGUUCCUGGUGCUGCUGCCCCUGGUGAGCAGCCAGUGCGUGAUGGCCAAGCUGUCCACAGACGAGCUGCUGGAUGCCUUCAAGGAAAUGACA
160   GCUCGAACUGAGCGACUUCGUGAAGAAGUUUGAGGAAACCUUCGAGGUGACCGCCGCUGCCCCUGUGGCCGUGGCCGCCGCCGGCGCCGCCCCUGCCGGCGCCGCCGUGGAAGCCGCUGAGGAACAGAGCGAGUUCGACGUGAUCCUGGAAGCCGCCCG
320   GAUAAGAAAAUCGGAGUGAUUAAGGUGGUGCGGGAAAUCGUGUCCGGCCUUGGACUGAAGGAGGCCAAGGACCUGGUGGACGGCGCUCCAAAGCCUCUGUUGGAGAAGGUCGCUAAGGAGGCUGCAGAUGAGGCCAAAGCCAAACUGGAGGCCGCCGG
480   CCACCGUGACCGUGAAGGAAGCUGCCGCCAAGGGCAUCAUCAACACCCUGCAGAAGUACUACUGCAGAGUGCGGGGUGGCAGAUGCGCCGUCCUGUCUUGUCUGCCUAAAGAAGAGCAAAUCGGCAAGUGCAGCACAAGAGGCAGAAAGUGCUGUAGA
640   GAAGAAGGAAGCCGCUGCCAAGACCCUGGGCCUGCUGCUCGCCUAUAGAAAGAAGUUCAUCGACCUGAACAUCACCAUGCUGAAGAAAAGAACCGCCCCUAGAAGCCUGAGCCUGAAAAAGAAAGAGGUGGACCUGGACUUCGGCCUGAAGAAAACCA
800   AUGGUGCUGAGAAAGCGGAACAAGGCUAGAUACAGCCCCGCUGCCUACGCCUACCGGAGACGGUUCCCCGCUGUCAUCACAAGAGUGCUGCCAGCCGCUUAUGCCGUGGACUUCAUCUGGACUGGCAACCAGAGAACAGCCCCCAGAGCCGCUUACAG
960   CCGGCAGAUUCCACUGGGAGCGGUUUAGCAAUGCCUCUCCAGCGGCCUACAACAAGCAGAGCACCCGGCCUACAGGCGCCUGCGUGUACCUGGAACCUGGCCCUGGACCUGGCACCAUGACCAAGUGGCAGGAGGUUGAUGAGAUGCUGCGGGCCGAG
1120  CGGCCCUGGACCCGGCGGCCGCGUUGUGUUCCUGCCCACCAUCAGAAGGCAGCUGGCCCUGGCCGAGGCUGCCGCUAAAGCCAAGUUUGUGGCCGCCUGGACACUGAAGGCCGCCGCCCACCACCACCAUCACCACUGAUAAUAGGCUGGAGCCUCGG
1280  GCCUAGCUUCUUGCCCCUUGGGCCUCCCCCCAGCCCCUCCUCCCCUUCCUGCACCCGUACCCCCGUGGUCUUUGAAUAAAGUCUGAGUGGGCGGCAAAAAAAAAAAAAAAAAAAAAAAAAAAAAAAGCAUAUGACUAAAAAAAAAAAAAAAAAAAAAA
1440  AAAAAAAAAAAAAAAAAAAAAAAAAAAAAAAAAAAAAAAAAAAAAA
```


```
1     .((.(((.(((((..(((((....)))).).................................................(((((.(((((.((((((((((...............(((((((.....))).))))..................))
160   )((((((((.................))))))))....((((((.(((.((.(((((.(((.....))).)))))......((((((((....).)))))))).(((.(((..((((.........))))..))).)))..(((((((......)))
320   )))......(((.....)))...((((((......((((.((((((((((.........)))))).)))).))))....(((.(((((.(((((((.((((.((...)).)))))))))))........(((....)))....))))))))).....
480   )))))))).))).)))))))..)))))))))).))))).......(((.((((((........)))))).))).........................((((((((((.....)).)))....))))))).(((((((............)))))))...
640   .....(((..(..((((..((((..(((((((.((((((((((((.........((((((((((.........((.(((((((...((..(((((.((....((....))...........)).)))))..))...))))))).)).......(((.
800   ..)))..(((((.(((((....(((((((...((((...)))).))).))))((((....))))...))))).)).)))..((((.(((((.((((((((...(((((((..((.(((((...(((...........))).)))))(((((((...)
960   .)))).)).))))).)))))))))))..)))..))))))((((.((((..(((((.((((.((...............)))))))).)))...))......)))))))).((((.(((.........))).))))))))))))))).......((((((..
1120  ))))))).((...((((((((.....(((((((((((..........))))).((((....))))((((.(((((..............))))).)))))))))....))))))))).))........((((....)))).))))))..))))...))
1280  ))))))..))))((((...))))......))))..).)))...................................................................................))))).))).))........................
1440  ..............................................
```

You can download the minimum free energy (MFE) structure in [Vienna Format| Ct Format]. You can get thermodynamic details on this structure by submitting to our RNAeval web server.

Graphical output

You may look at the interactive drawing of the MFE structure below. If you do not see the interactive drawing and you are using Internet Explorer, please install the Adobe SVG plugin. **A note on base-pairing probabilities:** The structure below is colored by base-pairing probabilities. For unpaired regions the color denotes the probability of being unpaired.

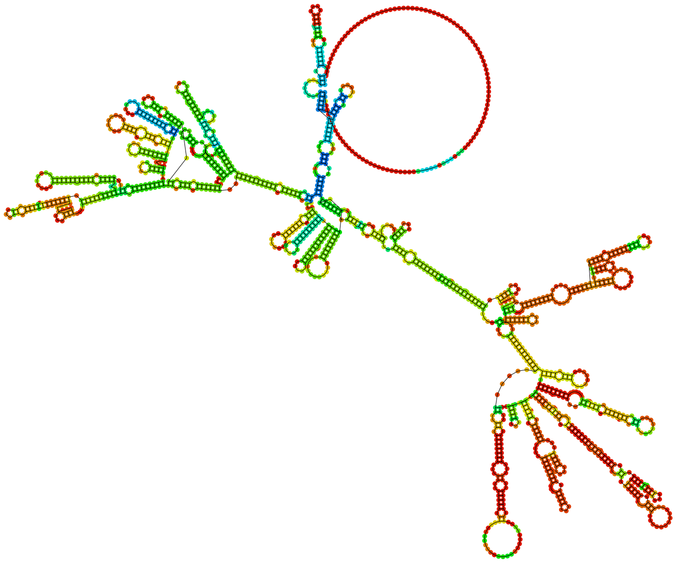

Sequence display options

◯ Plain Sequence
◉ No Sequence

MFE secondary structure

Other display options

◉ Base-pair probabilities
◯ Positional entropy
◯ None

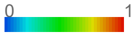

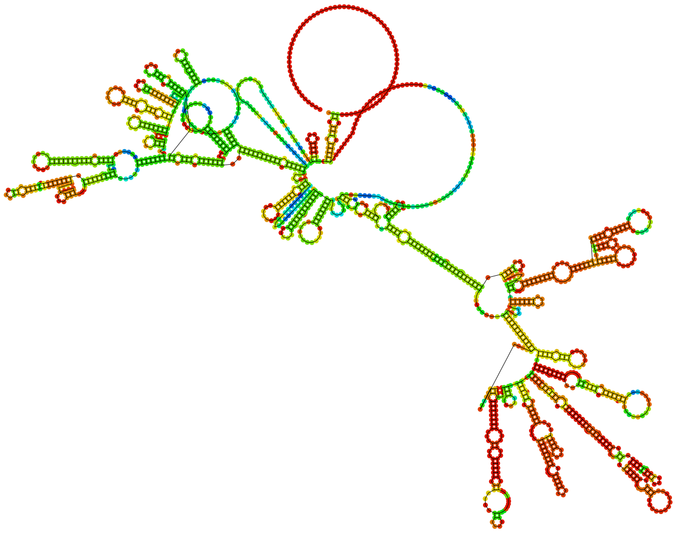

Sequence display options

◯ Plain Sequence
◉ No Sequence

Centroid secondary structure

Other display options

◉ Base-pair probabilities
◯ Positional entropy
◯ None

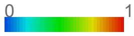

Image description	Download options
MFE plain structure drawing	[EPS | PDF | IMAGE CONVERTER | VIEW IN FORNA]
Centroid plain structure drawing	[EPS | PDF | IMAGE CONVERTER | VIEW IN FORNA]
MFE structure drawing encoding base-pair probabilities	[EPS | PDF | IMAGE CONVERTER | VIEW IN FORNA]
Centroid structure drawing encoding base-pair probabilities	[EPS | PDF | IMAGE CONVERTER | VIEW IN FORNA]
MFE structure drawing encoding positional entropy	[EPS | PDF | IMAGE CONVERTER | VIEW IN FORNA]
Centroid structure drawing encoding positional entropy	[EPS | PDF | IMAGE CONVERTER | VIEW IN FORNA]

Here you find a mountain plot representation of the MFE structure, the thermodynamic ensemble of RNA structures, and the centroid structure. Additionally we present the positional entropy for each position. Download as [EPS|PDF|IMAGE CONVERTER].

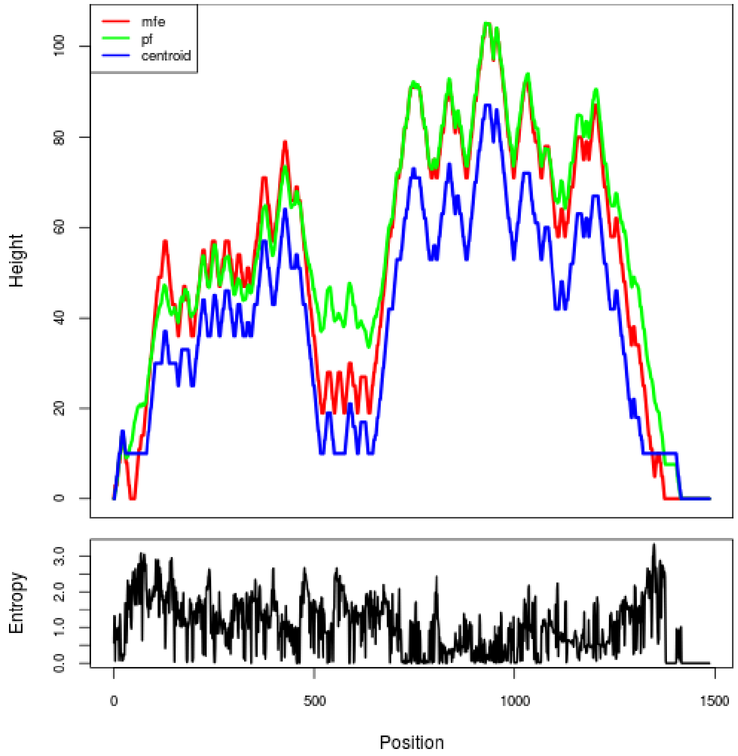

Results have been computed using RNAfold 2.6.3. An equivalent command line call would have been

`RNAfold -p -d2 --noLP < sequence1.fa > sequence1.out`

RNA parameters are described in

Mathews DH, Disney MD, Childs JL, Schroeder SJ, Zuker M, Turner DH. (2004) Incorporating chemical modification constraints into a dynamic programming algorithm for prediction of RNA secondary structure. *Proc Natl Acad Sci U S A* 101(19):7287-92.

If you find these results helpful for your work you may want to cite:

OPEN ACCESS OXFORD JOURNALS

Gruber AR, Lorenz R, Bernhart SH, Neuböck R, Hofacker IL.
The Vienna RNA Websuite. Nucleic Acids Research, Volume 36, Issue suppl_2, 1 July 2008, Pages W70-W74, DOI: 10.1093/nar/gkn188

Lorenz, R. and Bernhart, S.H. and Höner zu Siederdissen, C. and Tafer, H. and Flamm, C. and Stadler, P.F. and Hofacker, I.L. "ViennaRNA Package 2.0", Algorithms for Molecular Biology, 6:1 page(s): 26, 2011

RNAfold WebServer

1 Enter Input Parameters 2 View Results

[Home|New job|Help]

Results for minimum free energy prediction

The optimal secondary structure in dot-bracket notation with a minimum free energy of **-530.20** kcal/mol is given below.

[color by base-pairing probability | color by positional entropy | no coloring]

```
1     GAGACAUUUGCUUCUGACACAACUGUGUUCACUAGCAACCUCAAACAGACACCGCCACCAUGUUCGUGUUCCUGGUGCUGCUGCCCCUGGUGAGCAGCCAGUGCGUGAUGGCCAAGCUGUCCACAGACGAGCUGCUGGAUGCCUUCAAGGAAAUGACA
160   GCUCGAACUGAGCGACUUCGUGAAGAAGUUUGAGGAAACCUUCGAGGUGACCGCCGCUGCCCCUGUGGCCGUGGCCGCCGCCGGCGCCGCCCCUGCCGGCGCCGCCGUGGAAGCCGCUGAGGAACAGAGCGAGUUCGACGUGAUCCUGGAAGCCGCCG
320   GAUAAGAAAAUCGGAGUGAUUAAGGUGGUGCGGGAAAUCGUGUCCGGCCUUGGACUGAAGGAGGCCAAGGACCUGGUGGACGGCGCUCCAAAGCCUCUGUUGGAGAAGGUCGCUAAGGAGGCUGCAGAUGAGGCCAAAGCCAAACUGGAGGCCGCCGG
480   CCACCGUGACCGUGAAGGAAGCUGCCGCCAAGGGCAUCAUCAACACCCUGCAGAAGUACUACUGCAGAGUGCGGGGUGGCAGAUGCGCCGUCCUGUCUUGUCUGCCUAAAGAAGAGCAAAUCGGCAAGUGCAGCACAAGAGGCAGAAAGUGCUGUAGA
640   GAAGAAGGAAGCCGCUGCCAAGACCCUGGGCCUGCUGCUCGCCUAUAGAAAGAAGUUCAUCGACCUGAACAUCACCAUGCUGAAGAAAAGAACCGCCCCUAGAAGCCUGAGCCUGAAAAAGAAAGAGGUGGACCUGGACUUCGGCCUGAAGAAAACCA
800   AUGGUGCUGAGAAAGCGGAACAAGGCUAGAUACAGCCCCGCUGCCUACGCCUACCGGAGACGGUUCCCCGCUGUCAUCACAAGAGUGCUGCCAGCCGCUUAUGCCGUGGACUUCAUCUGGACUGGCAACCAGAGAACAGCCCCCAGAGCCGCUUACAG
960   CCGGCAGAUUCCACUGGGAGCGGUUUAGCAAUGCCUCUCCAGCGGCCUACAACAAGCAGAGCACCCGGCCUACAGGCGCCUGCGUGUACCUGGAACCUGGCCCUGGACCUGGCACCAUGACCAAGUGGCAGGAGGUUGAUGAGAUGCUGCGGGCCGAG
1120  CGGCCCUGGACCCGGCGGCCGCGUUGUGUUCCUGCCCACCAUCAGAAGGCAGCUGGCCCUGGCCGAGGCUGCCGCUAAAGCCAAGUUUGUGGCCGCCUGGACACUGAAGGCCGCCGCCCACCACCACCAUCACCACUGAUAAUAGGCUGGAGCCUCGG
1280  GCCUAGCUUCUUGCCCCUUGGGCCUCCCCCCAGCCCCUCCUCCCCUUCCUGCACCCGUACCCCCGUGGUCUUUGAAUAAAGUCUGAGUGGGCGGCAAAAAAAAAAAAAAAAAAAAAAAAAAAAAAAAAAGCAUAUGACUAAAAAAAAAAAAAAAAAAAAAAA
1440  AAAAAAAAAAAAAAAAAAAAAAAAAAAAAAAAAAAAAAAAAAAAAAA
```

```
1     (((....(((((..((((((....))).)))..))))).)))........((((((((((...(((.....((((((.(((((.(((((.((((((((((((((.(((......((((((((.....))).)))))))).)))......)).))
160   )((((((((..(((....))).....))))))))....((((((.(((.((.(((((.(((.....))).)))))((.((((((((.......)))))))).)).(((.(((..((((.........))))..))).)))..(((((((......)))
320   ))).....((((.....))))..((((((((.((..((((.((((((((((.........))))))..)))).))))...((((.(((((.(((((((.((((.((...)).))))))))))).........(((....)))....)))))))))))).)
480   ))))))).))).))))))..))))))))))).))))).))....(((.((((((........)))))).)))((((((((......))))))))).(((((((((((.....)).)))....))))))((((((((............)))))))).. 
640   ((((.(((.((..((((..((((..(((((((.(((((((((((((....((...((((((((((.....((..((.(((((((...((..(((((.((......................)).)))))..))...)))))))).)).)).)..((((
800   .))))..(((((.(((((....((((((...((((...)))).))).))))((((....))))...))))).)).)))..((((.(((((.((((((((...(((((((..((.(((((...(((...........))).)))))(((((((...)
960   .)))).)).))))).))))))))))..)))..))))))((((.((((..(((((.((((.((.((.........)).)))))).)))...))......)))))))).((((.(((.........))).))))))))))))))...))..((((((..
1120  ))))))(((...((((((((.....((((((((((..........))))).((((....))))((((.(((((.(((......)))))))).)))))))))))....))))))))).)))........((((....)))).)))))).)))))...))
1280  ))))))..))))((((...))))......))))..)))))...))))))))))))..)))...))))))(((.((......)).)))..)))))..................................................................
1440  ...............................................
```

You can download the minimum free energy (MFE) structure in [Vienna Format| Ct Format]. You can get thermodynamic details on this structure by submitting to our RNAeval web server.

Results for thermodynamic ensemble prediction

The free energy of the thermodynamic ensemble is **-550.58** kcal/mol.

The frequency of the MFE structure in the ensemble is **0.00** %.

The ensemble diversity is **378.51**.

You may look at the **dot plot** containing the base pair probabilities [EPS|PDF|IMAGE CONVERTER].

The centroid secondary structure in dot-bracket notation with a minimum free energy of **-449.80** kcal/mol is given below.

[color by base-pairing probability | color by positional entropy | no coloring]


```
1     GAGACAUUUGCUUCUGACACAACUGUGUUCACUAGCAACCUCAAACAGACACCGCCACCAUGUUCGUGUUCCUGGUGCUGCUGCCCCUGGUGAGCAGCCAGUGCGUGAUGGCCAAGCUGUCCACAGACGAGCUGCUGGAUGCCUUCAAGGAAAUGACA
160   GCUCGAACUGAGCGACUUCGUGAAGAAGUUUGAGGAAACCUUCGAGGUGACCGCCGCUGCCCCUGUGGCCGUGGCCGCCGCCGGCGCCGCCCCUGCCGGCGCCGCCGUGGAAGCCGCUGAGGAACAGAGCGAGUUCGACGUGAUCCUGGAAGCCGCCG
320   GAUAAGAAAAUCGGAGUGAUUAAGGUGGUGCGGGAAAUCGUGUCCGGCCUUGGACUGAAGGAGGCCAAGGACCUGGUGGACGGCGCUCCAAAGCCUCUGUUGGAGAAGGUCGCUAAGGAGGCUGCAGAUGAGGCCAAAGCCAAACUGGAGGCCGCCGG
480   CCACCGUGACCGUGAAGGAAGCUGCCGCCAAGGGCAUCAUCAACACCCUGCAGAAGUACUACUGCAGAGUGCGGGGUGGCAGAUGCGCCGUCCUGUCUUGUCUGCCUAAAGAAGAGCAAAUCGGCAAGUGCAGCACAAGAGGCAGAAAGUGCUGUAGA
640   GAAGAAGGAAGCCGCUGCCAAGACCCUGGGCCUGCUGCUCGCCUAUAGAAAGAAGUUCAUCGACCUGAACAUCACCAUGCUGAAGAAAAGAACCGCCCCUAGAAGCCUGAGCCUGAAAAAGAAAGAGGUGGACCUGGACUUCGGCCUGAAGAAAACCA
800   AUGGUGCUGAGAAAGCGGAACAAGGCUAGAUACAGCCCCGCUGCCUACGCCUACCGGAGACGGUUCCCCGCUGUCAUCACAAGAGUGCUGCCAGCCGCUUAUGCCGUGGACUUCAUCUGGACUGGCAACCAGAGAACAGCCCCCAGAGCCGCUUACAG
960   CCGGCAGAUUCCACUGGGAGCGGUUUAGCAAUGCCUCUCCAGCGGCCUACAACAAGCAGAGCACCCGGCCUACAGGCGCCUGCGUGUACCUGGAACCUGGCCCUGGACCUGGCACCAUGACCAAGUGGCAGGAGGUUGAUGAGAUGCUGCGGGCCGAG
1120  CGGCCCUGGACCCGGCGGCCGCGUUGUGUUCCUGCCCACCAUCAGAAGGCAGCUGGCCCUGGCCGAGGCUGCCGCUAAAGCCAAGUUUGUGGCCGCCUGGACACUGAAGGCCGCCGCCCACCACCACCAUCACCACUGAUAAUAGGCUGGAGCCUCGG
1280  GCCUAGCUUCUUGCCCCUUGGGCCUCCCCCCAGCCCCUCCUCCCCUUCCUGCACCCGUACCCCCGUGGUCUUUGAAUAAAGUCUGAGUGGGCGGCAAAAAAAAAAAAAAAAAAAAAAAAAAAAAAAAAAGCAUAUGACUAAAAAAAAAAAAAAAAAAAAAAA
1440  AAAAAAAAAAAAAAAAAAAAAAAAAAAAAAAAAAAAAAAAAAAAAAA
```


```
1     .((.(((.(((((..(((((....)))).)...............................................(((((.(((((.((((((((((................((((((.....))).))))..................))
160   )((((((((.................))))))))....((((((.(((.((.(((((.(((.....))).)))))......((((((((....).))))))).(((.(((..((((.........))))..))).)))..(((((((......)))
320   ))).......(((.....)))...(((((((......((((.((((((((((.........))))))..)))).))))....(((.(((((.(((((((.((((.((...)).))))))))))).........(((....)))....))))))))....
480   ))))))).))).))))))..))))))))))).)))))........(((.((((((........)))))).)))........................((((((((((.....)).)))....)))))).(((((((...........)))))))...
640   .....(((..(..((((..((((..(((((((.((((((((((((.........((((((((((.........((.(((((((...((..(((((.((....((....))...........)).)))))..))...))))))).)).......(((.
800   ..))).((((((.(((((....((((((...((((...)))).))).))))((((....))))...))))).)).)))..((((.(((((.((((((((...(((((((..((.(((((...(((...........))).)))))(((((((...)
960   .)))).)).)))))).))))))))))..)))..))))))((((.((((..(((((.((((.((...............)))))).)))...))......)))))))).((((.(((.........))).))))))))))))))).......(((((..
1120  ))))))).((...((((((((.....((((((((((...........))))).((((....))))((((.(((((............))))).))))))))))....)))))))).)).........((((....)))).)))))))..))))...))
1280  ))))))..))))((((...))))......))))..).)))..........................................................................)))))).))).)).......................
1440  ...............................................
```

You can download the minimum free energy (MFE) structure in [Vienna Format| Ct Format]. You can get thermodynamic details on this structure by submitting to our RNAeval web server.

Graphical output

You may look at the interactive drawing of the MFE structure below. If you do not see the interactive drawing and you are using Internet Explorer, please install the Adobe SVG plugin. **A note on base-pairing probabilities:** The structure below is colored by base-pairing probabilities. For unpaired regions the color denotes the probability of being unpaired.

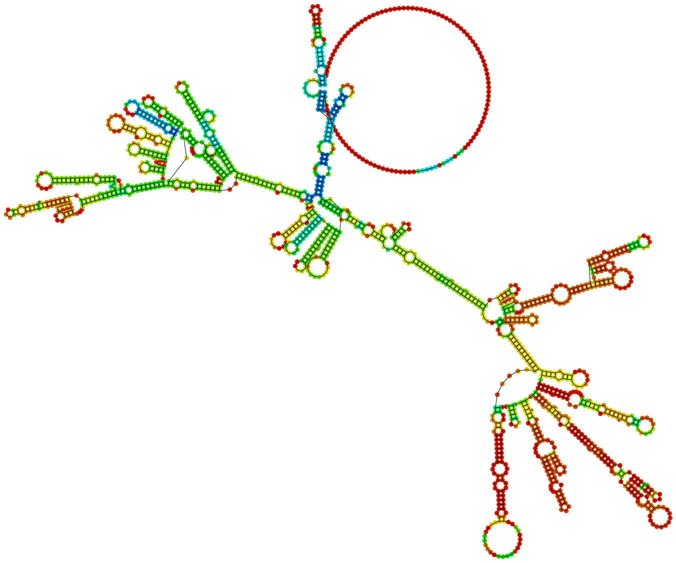

Sequence display options

- ○ Plain Sequence
- ◉ No Sequence

MFE secondary structure

Other display options

- ◉ Base-pair probabilities
- ○ Positional entropy
- ○ None

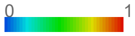

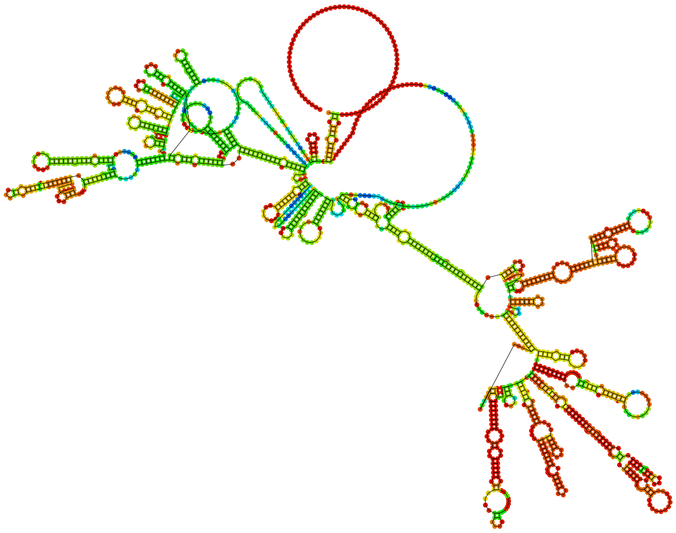

Sequence display options

- ○ Plain Sequence
- ◉ No Sequence

Centroid secondary structure

Other display options

- ◉ Base-pair probabilities
- ○ Positional entropy
- ○ None

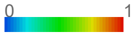

Image description	Download options
MFE plain structure drawing	[EPS | PDF | IMAGE CONVERTER | VIEW IN FORNA]
Centroid plain structure drawing	[EPS | PDF | IMAGE CONVERTER | VIEW IN FORNA]
MFE structure drawing encoding base-pair probabilities	[EPS | PDF | IMAGE CONVERTER | VIEW IN FORNA]
Centroid structure drawing encoding base-pair probabilities	[EPS | PDF | IMAGE CONVERTER | VIEW IN FORNA]
MFE structure drawing encoding positional entropy	[EPS | PDF | IMAGE CONVERTER | VIEW IN FORNA]
Centroid structure drawing encoding positional entropy	[EPS | PDF | IMAGE CONVERTER | VIEW IN FORNA]

Here you find a mountain plot representation of the MFE structure, the thermodynamic ensemble of RNA structures, and the centroid structure. Additionally we present the positional entropy for each position. Download as [EPS|PDF|IMAGE CONVERTER].

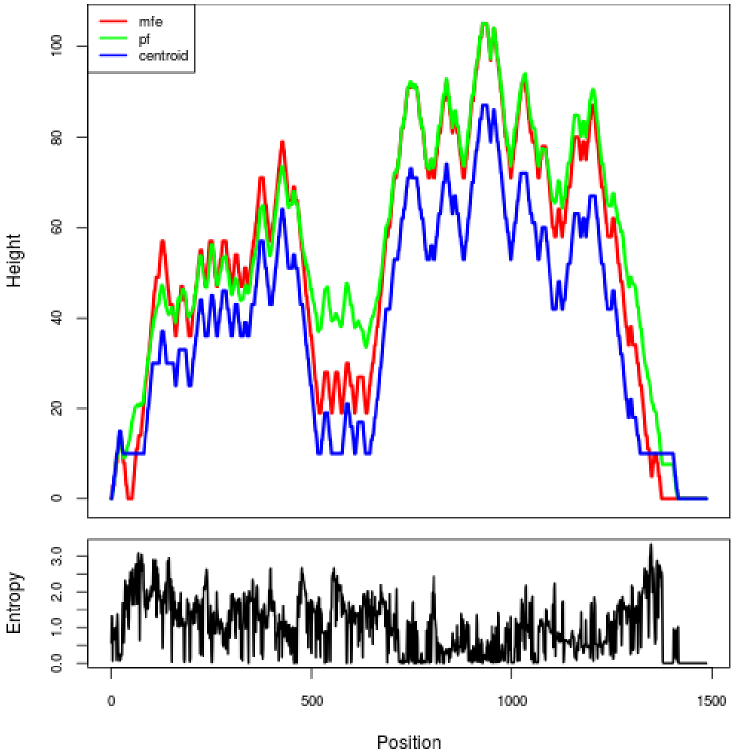

Results have been computed using RNAfold 2.6.3. An equivalent command line call would have been

`RNAfold -p -d2 --noLP < sequence1.fa > sequence1.out`

RNA parameters are described in

Mathews DH, Disney MD, Childs JL, Schroeder SJ, Zuker M, Turner DH. (2004) Incorporating chemical modification constraints into a dynamic programming algorithm for prediction of RNA secondary structure. *Proc Natl Acad Sci U S A* 101(19):7287-92.

If you find these results helpful for your work you may want to cite:

OPEN ACCESS OXFORD JOURNALS Gruber AR, Lorenz R, Bernhart SH, Neuböck R, Hofacker IL. **The Vienna RNA Websuite.** Nucleic Acids Research, Volume 36, Issue suppl_2, 1 July 2008, Pages W70-W74, DOI: 10.1093/nar/gkn188

Lorenz, R. and Bernhart, S.H. and Höner zu Siederdissen, C. and Tafer, H. and Flamm, C. and Stadler, P.F. and Hofacker, I.L. "ViennaRNA Package 2.0", Algorithms for Molecular Biology, 6:1 page(s): 26, 2011

Institute for Theoretical Chemistry | University of Vienna | rna@tbi.univie.ac.at
