# Supplementary material for: Reverse vaccinology-based design of multivalent multiepitope mRNA vaccines targeting key viral proteins of Herpes Simplex Virus type-2
Source: Front Immunol. 2025 May 20;16:1586271. doi: 10.3389/fimmu.2025.1586271 (PMC12130045; doi:10.3389/fimmu.2025.1586271)
Supplement: Supplementary file 1 [file DataSheet1.zip › Supplementary Data_22-04-2025/Supplementary Data 11.pdf]

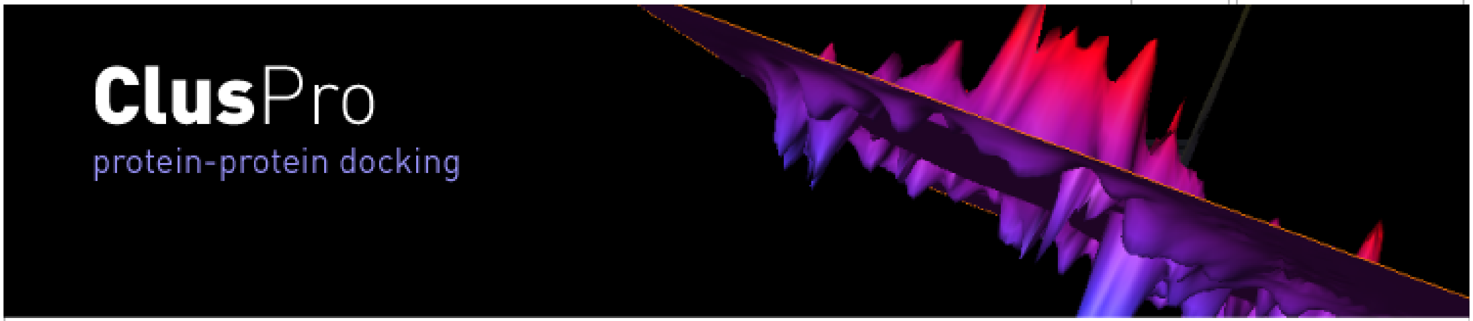

Job Details: 3DX7_KEV

View Models

Balanced | Electrostatic-favored | Hydrophobic-favored | VdW+Elec

Download Model Scores for this Coefficient

Coefficient Weights

See *Kozakov et. al.* in Papers for a description of these terms

$$E = 0.40E_{rep} + -0.40E_{att} + 600E_{elec} + 1.00E_{DARS}$$

Cluster Scores

We strongly encourage you to read the FAQ related to these scores before using them.

Cluster	Members	Representative	Weighted Score
**0**	350	Center	-577.2
		Lowest Energy	-577.2
**1**	232	Center	-469.3
		Lowest Energy	-545.7
**2**	225	Center	-465.4
		Lowest Energy	-550.4
**3**	98	Center	-525.1
		Lowest Energy	-525.1
**4**	87	Center	-473.6
		Lowest Energy	-517.4

ClusPro should only be used for noncommercial purposes.
Vajda Lab and ABC Group
Boston University and Stony Brook University

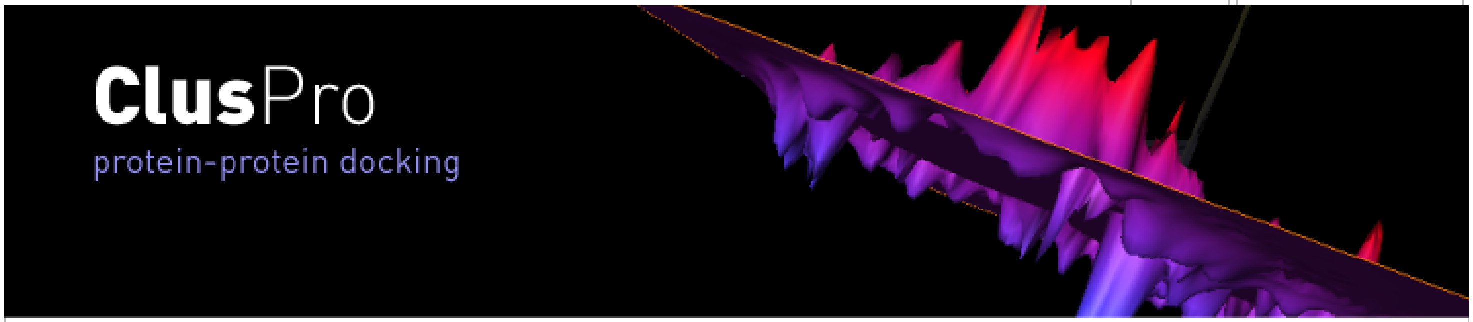

Job Details: 3OXR_FID_2

View Models

Balanced | Electrostatic-favored | Hydrophobic-favored | VdW+Elec

Download Model Scores for this Coefficient

Coefficient Weights

See *Kozakov et. al.* in Papers for a description of these terms

$$E = 0.40E_{rep} + -0.40E_{att} + 600E_{elec} + 1.00E_{DARS}$$

Cluster Scores

We strongly encourage you to read the FAQ related to these scores before using them.

Cluster	Members	Representative	Weighted Score
**0**	816	Center	-581.5
		Lowest Energy	-702.6
**1**	73	Center	-659.7
		Lowest Energy	-659.7
**2**	65	Center	-624.7
		Lowest Energy	-624.7
**3**	45	Center	-566.8
		Lowest Energy	-625.6

ClusPro should only be used for noncommercial purposes.
Vajda Lab and ABC Group
Boston University and Stony Brook University

ClusPro
protein-protein docking

Job Details: 5VUF_RTA_2

View Models

Balanced | Electrostatic-favored | Hydrophobic-favored | VdW+Elec

Download Model Scores for this Coefficient

Coefficient Weights

See *Kozakov et. al.* in Papers for a description of these terms

$$E = 0.40E_{rep} + -0.40E_{att} + 600E_{elec} + 1.00E_{DARS}$$

Cluster Scores

We strongly encourage you to read the FAQ related to these scores before using them.

Cluster	Members	Representative	Weighted Score
**0**	539	Center	-584.7
		Lowest Energy	-677.4
**1**	310	Center	-580.5
		Lowest Energy	-650.2
**2**	91	Center	-577.7
		Lowest Energy	-662.4
**3**	38	Center	-601.9
		Lowest Energy	-628.3
**4**	17	Center	-584.3
		Lowest Energy	-599.5

ClusPro should only be used for noncommercial purposes.
Vajda Lab and ABC Group
Boston University and Stony Brook University

Dimer Classification | Queue | **Results** | Preferences | Downloads | Papers | Help | Contact | Dock | Peptide Docking

sign out

Job Details: AVD_5ni9_3

View Models

Balanced | Electrostatic-favored | Hydrophobic-favored | VdW+Elec

Download Model Scores for this Coefficient

Coefficient Weights

See *Kozakov et. al.* in Papers for a description of these terms

$$E = 0.40E_{rep} + -0.40E_{att} + 600E_{elec} + 1.00E_{DARS}$$

Cluster Scores

We strongly encourage you to read the FAQ related to these scores before using them.

Cluster	Members	Representative	Weighted Score
**0**	254	Center	-776.2
		Lowest Energy	-800.8
**1**	173	Center	-779.9
		Lowest Energy	-811.5
**2**	129	Center	-671.5
		Lowest Energy	-762.4
**3**	112	Center	-715.4
		Lowest Energy	-751.5
**4**	98	Center	-667.8
		Lowest Energy	-787.9
**5**	93	Center	-663.1
		Lowest Energy	-815.7
**6**	78	Center	-675.9
		Lowest Energy	-743.0
**7**	62	Center	-664.1

Cluster	Members	Representative	Weighted Score
		Lowest Energy	-713.3

ClusPro should only be used for noncommercial purposes.
Vajda Lab and ABC Group
Boston University and Stony Brook University
