# Supplementary material for: Reverse vaccinology-based design of multivalent multiepitope mRNA vaccines targeting key viral proteins of Herpes Simplex Virus type-2
Source: Front Immunol. 2025 May 20;16:1586271. doi: 10.3389/fimmu.2025.1586271 (PMC12130045; doi:10.3389/fimmu.2025.1586271)

HLA Docking Interaction plot

LIGPLOT of KEVDLDFGL with HLA-B*44:03

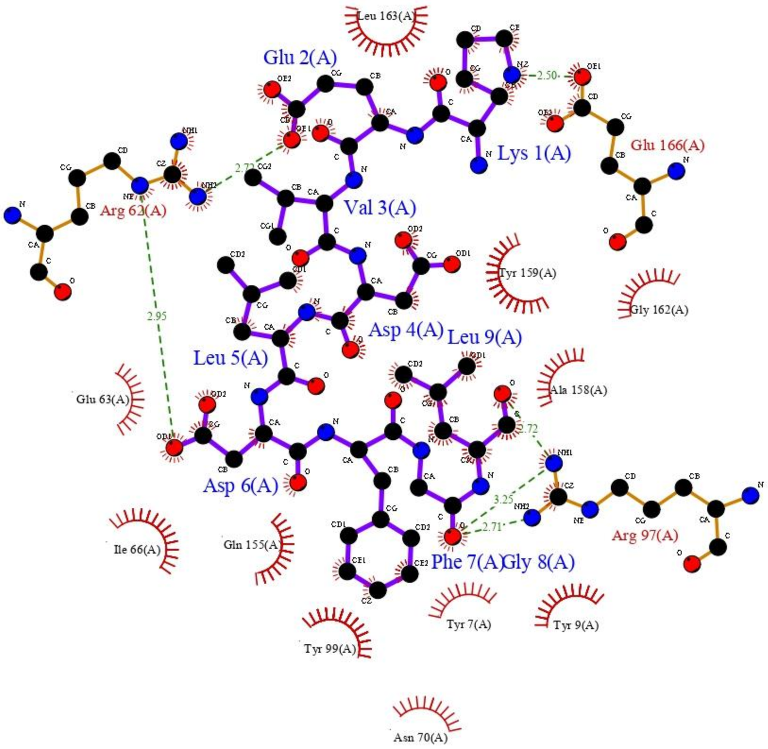

LIGPLOT of RTAPRSLSL with HLA-B*57:01

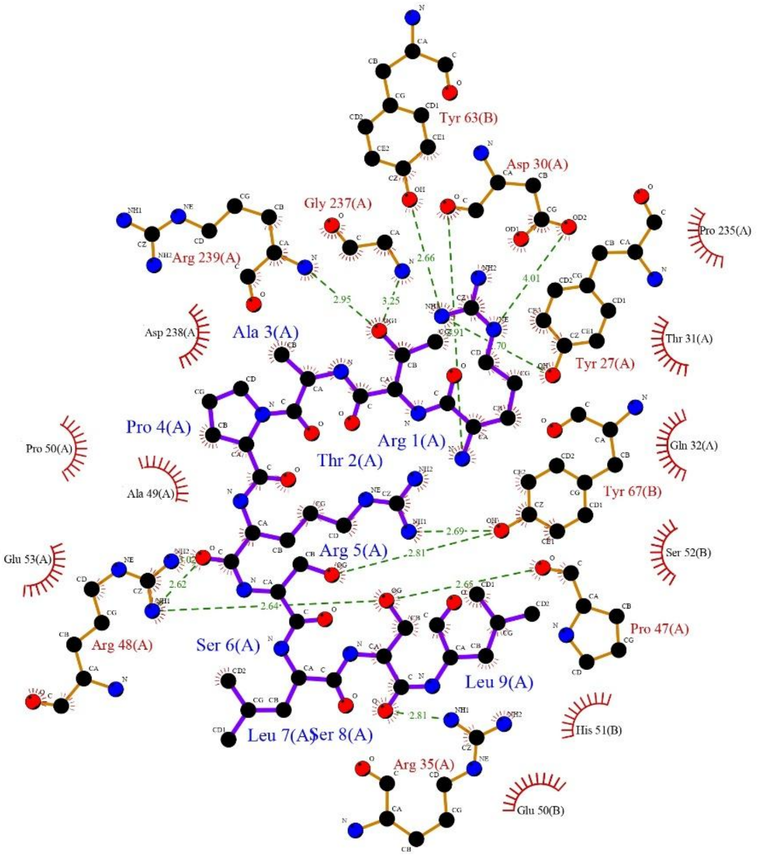

LIGPLOT of FIDLNITML with HLA-A 02:06

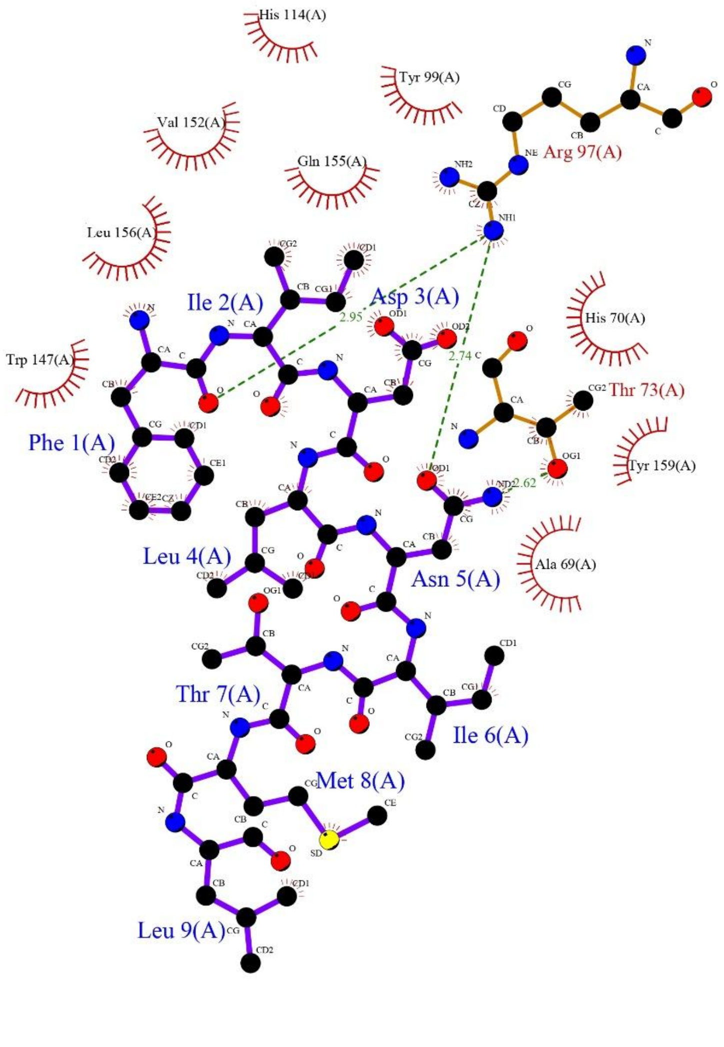

LIGPLOT of AVDFIWTGNQRTAPR with HLA-DRB1*04:01

Chain A – TLR4

Chain B – MD2

Chain C - Epitope

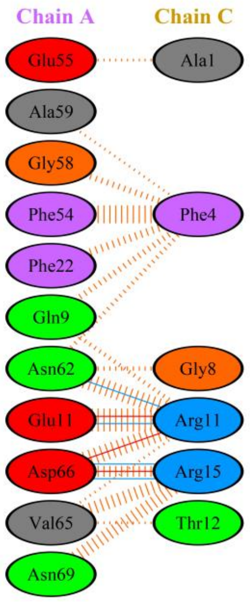

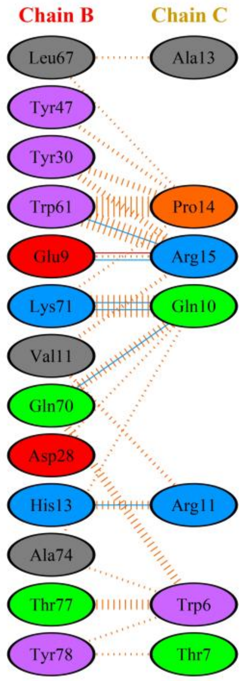

Supplement: Supplementary file 1 [file DataSheet1.zip › Supplementary Data_22-04-2025/Supplementary Data 12A.pdf]
