# Supplementary material for: Reverse vaccinology-based design of multivalent multiepitope mRNA vaccines targeting key viral proteins of Herpes Simplex Virus type-2
Source: Front Immunol. 2025 May 20;16:1586271. doi: 10.3389/fimmu.2025.1586271 (PMC12130045; doi:10.3389/fimmu.2025.1586271)
Supplement: Supplementary file 1 [file DataSheet1.zip › Supplementary Data_22-04-2025/Supplementary Data 2A - C1_753.pdf]

ElliPro: Epitope 3D Structures for filev3jm3hha.pdb

No.	Residues	Number of residues	Score
1	A:A377, A:A378, A:K379, A:A380, A:K381, A:F382, A:V383, A:A384, A:A385, A:W386, A:T387, A:L388, A:K389, A:A390, A:A391, A:A392, A:H393, A:H394, A:H395, A:H396, A:H397, A:H398	22	0.891

JSmol-Rendered PDB Structure

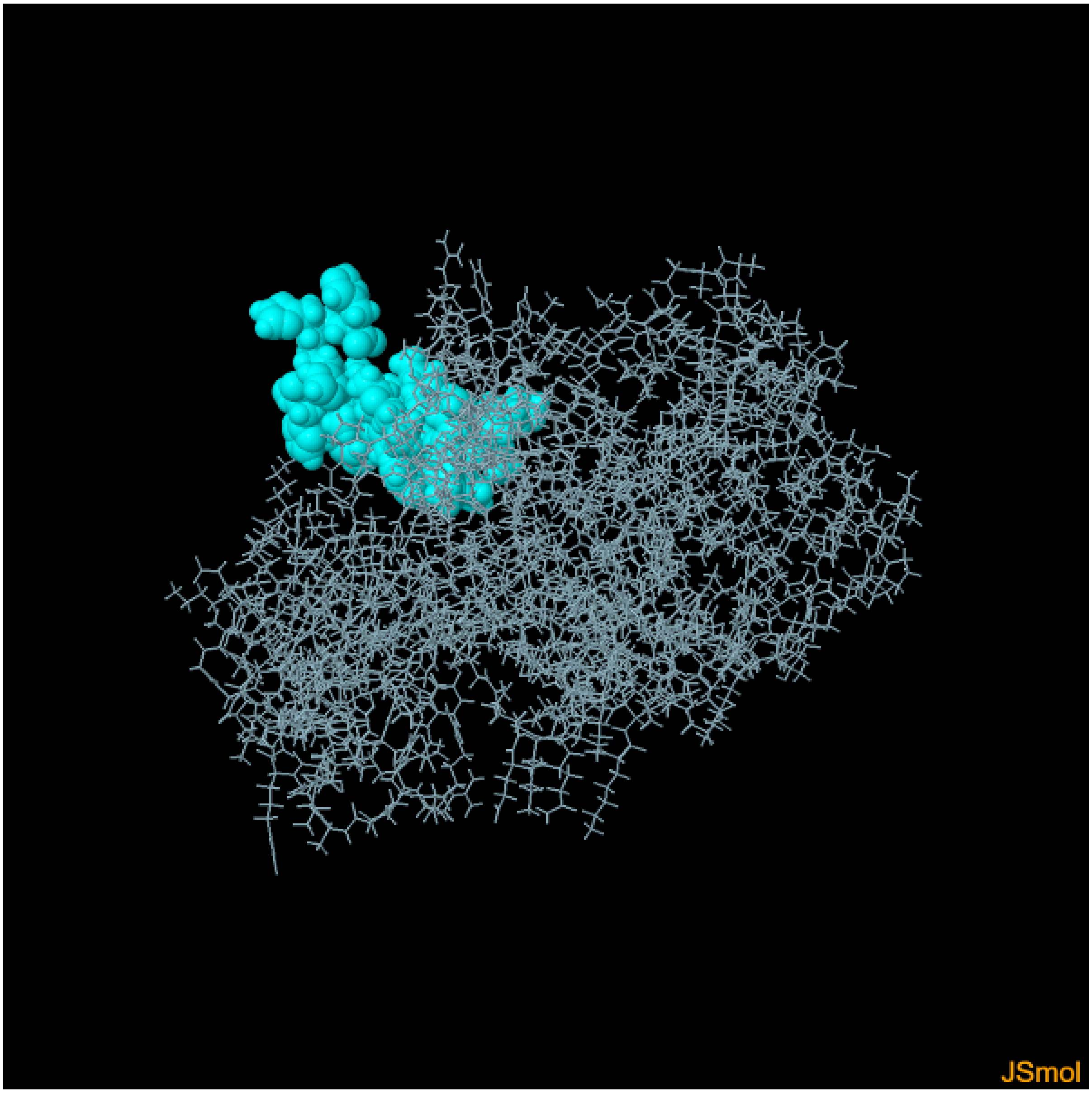

© 2005-2024 IEDB Home

ElliPro: Epitope 3D Structures for filev3jm3hha.pdb

No.	Residues	Number of residues	Score
2	A:A300, A:G301, A:F303, A:H304, A:E306, A:R307, A:F308	7	0.739

JSmol-Rendered PDB Structure

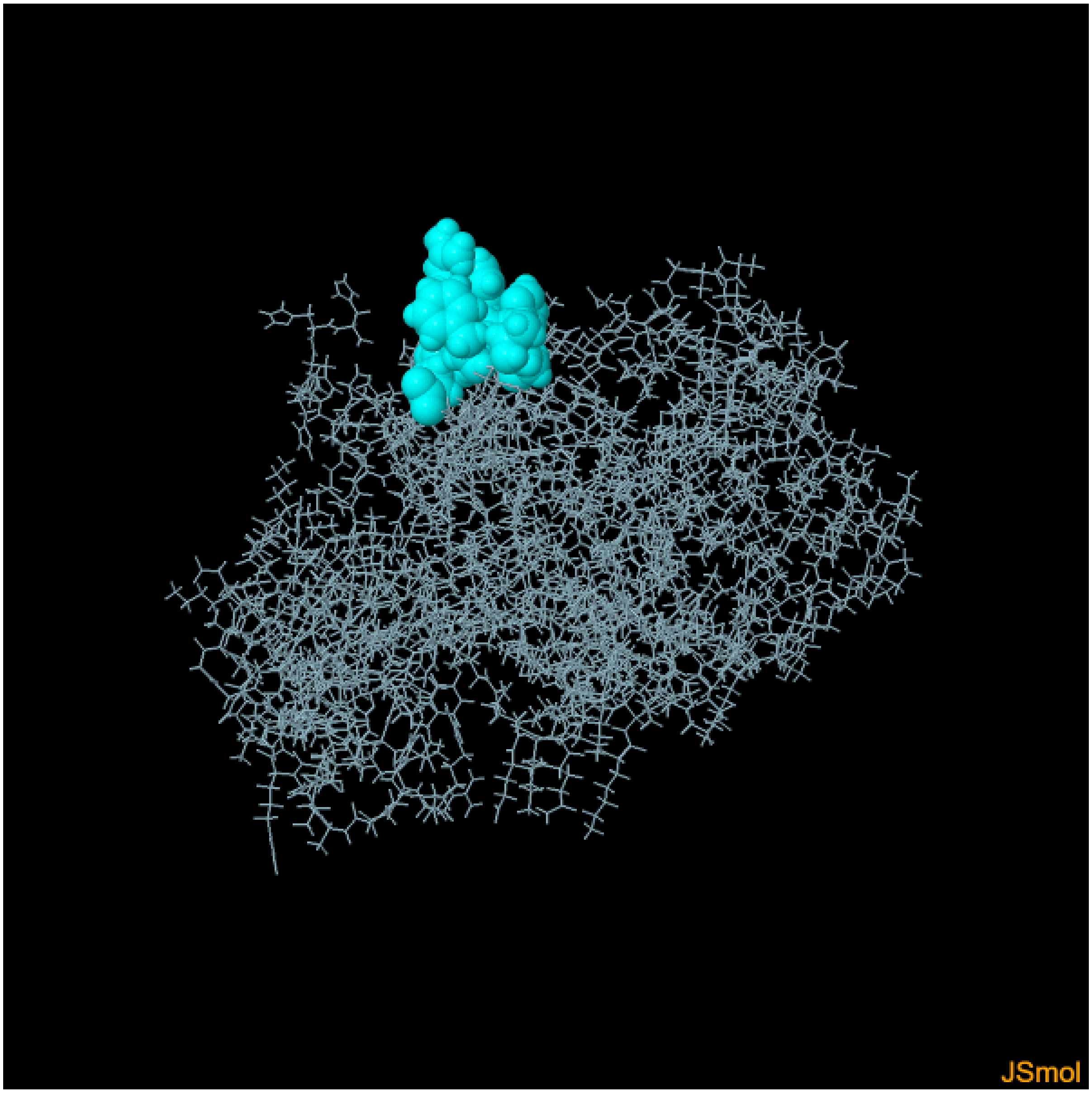

© 2005-2024 IEDB Home

ElliPro: Epitope 3D Structures for filev3jm3hha.pdb

No.	Residues	Number of residues	Score
3	A:F1, A:V2, A:F3, A:L4, A:V5, A:L6, A:L174, A:P175, A:K176, A:E177, A:E178, A:Q179, A:I180, A:G181, A:K182, A:C183, A:S184, A:T185, A:R186, A:G187, A:R188, A:K189, A:C190, A:C191, A:R192, A:A218, A:Y219, A:K221, A:K222, A:R223, A:T224, A:A225, A:P226, A:R227, A:S228, A:L229, A:S230, A:K232, A:K233, A:K234, A:E235, A:V236, A:D237, A:L238, A:D239, A:F240, A:G241, A:L242, A:K243, A:K244, A:T245, A:L249, A:N253, A:K254, A:A255, A:Y257, A:S258, A:A261, A:Y262, A:A263, A:Y264, A:R265, A:R266, A:R267, A:F268, A:P269, A:A270, A:V271, A:I272, A:T273, A:R274, A:V275, A:L276, A:P277, A:A278, A:A279, A:Y280, A:A281, A:V282, A:D283, A:F284, A:I285, A:W286, A:T287, A:N289, A:Q290, A:T324	87	0.713

JSmol-Rendered PDB Structure

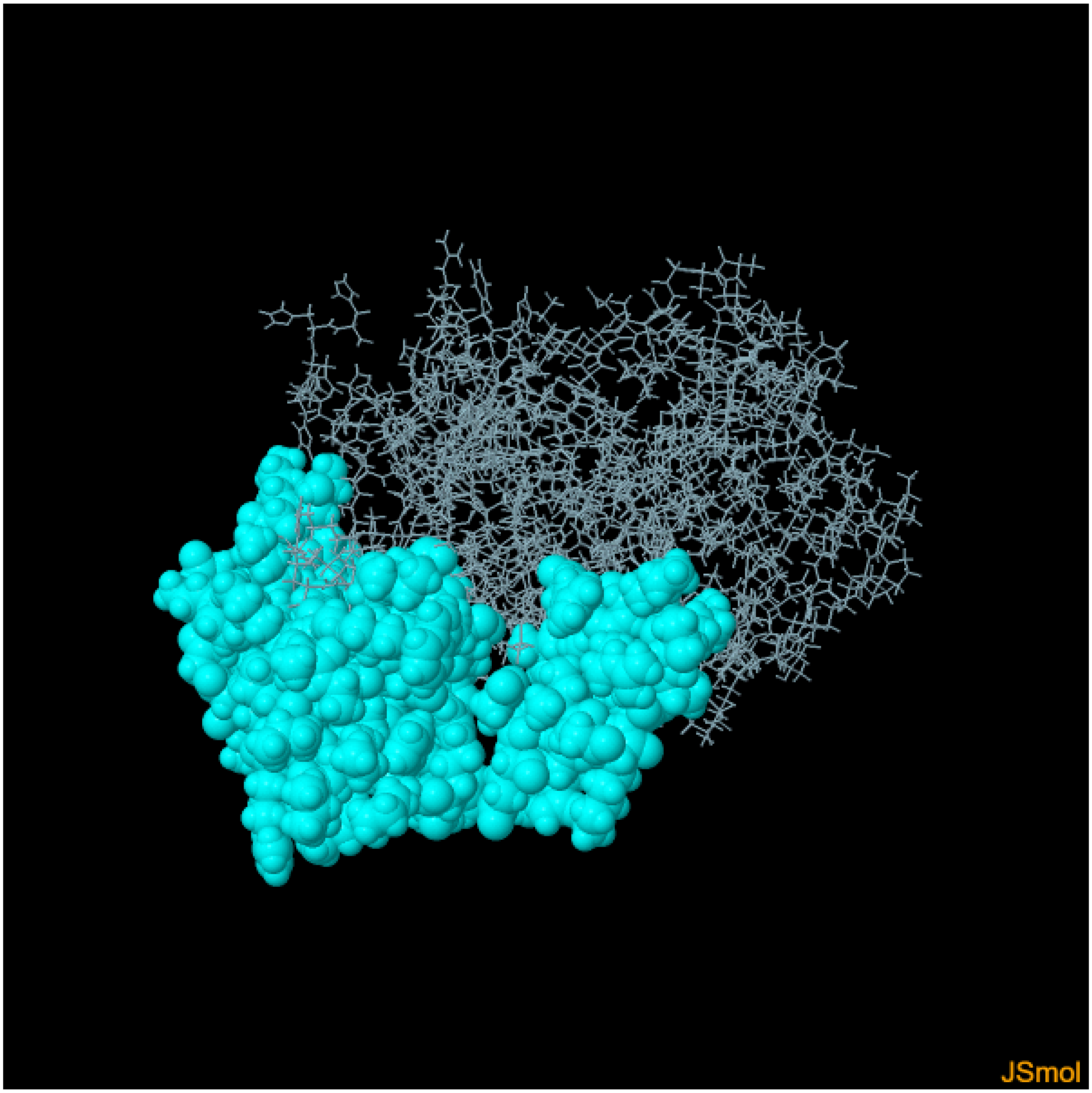

© 2005-2024 IEDB Home

ElliPro: Epitope 3D Structures for filev3jm3hha.pdb

No.	Residues	Number of residues	Score
4	A:R291, A:T292, A:A293, A:P294, A:R295, A:A296, A:A297, A:Y298, A:R299	9	0.678

JSmol-Rendered PDB Structure

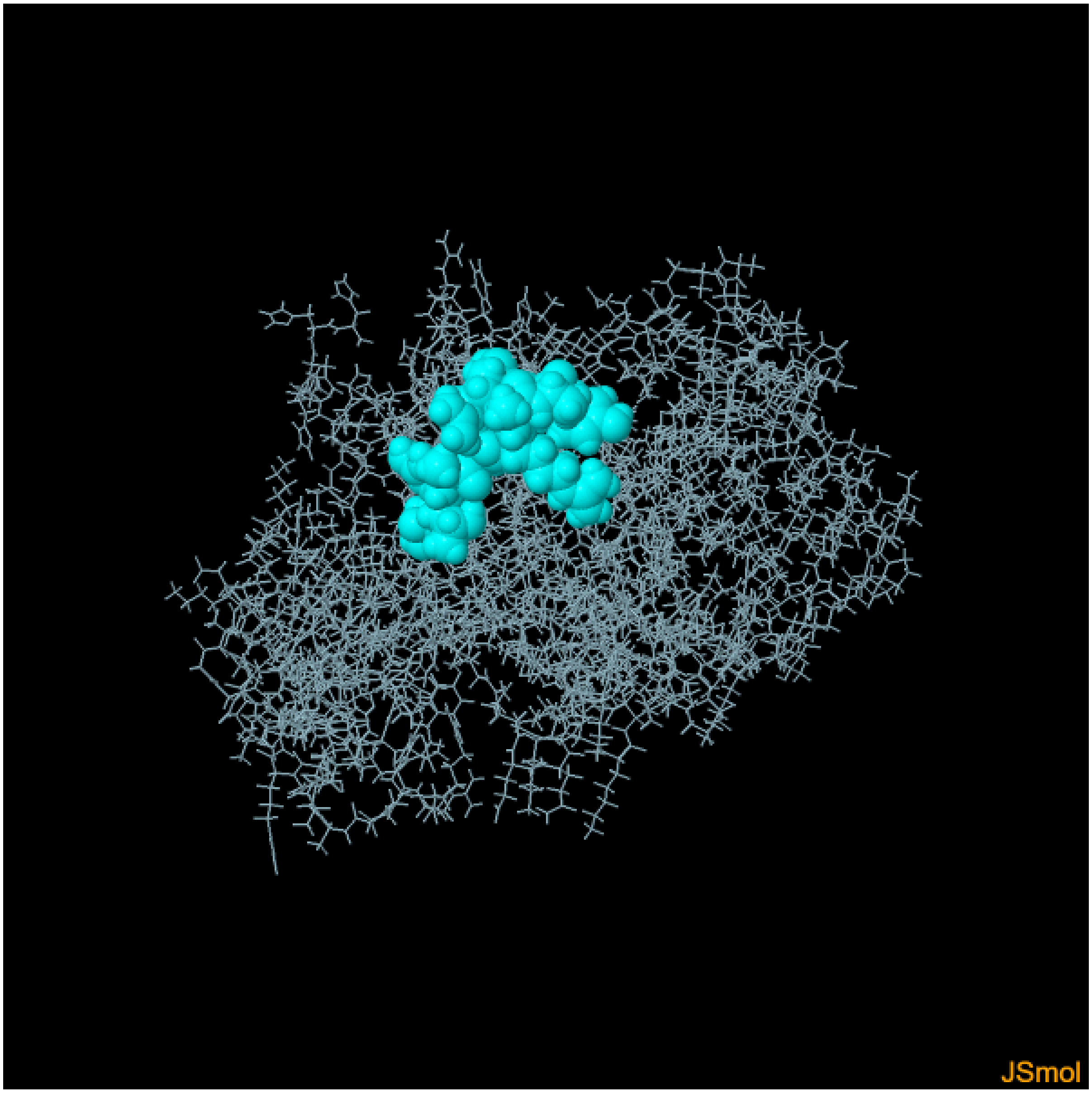

© 2005-2024 IEDB Home

ElliPro: Epitope 3D Structures for filev3jm3hha.pdb

No.	Residues	Number of residues	Score
5	A:A17, A:L19, A:S20, A:T21, A:D22, A:E23, A:L24, A:L25, A:D26, A:K29, A:V40, A:K41, A:K42, A:F43, A:E44, A:E45, A:T46, A:F47, A:V49, A:T50, A:A51, A:A52, A:A53, A:P54, A:V55, A:A58, A:G61, A:A63, A:P64, A:A65, A:G66, A:A67, A:V69, A:E70, A:E73, A:A108, A:K109, A:D110, A:L111, A:V112, A:D113, A:G114, A:A115, A:P116, A:K117, A:P118, A:L119, A:L120, A:E121, A:K122, A:V123, A:A124, A:K125, A:E126, A:A127, A:A128, A:D129, A:E130, A:A131, A:K132, A:A133, A:K134, A:L135, A:E136, A:A137, A:A138, A:G139, A:A140, A:T141, A:V142, A:T143, A:E347, A:M348, A:R350, A:A351, A:E352, A:Y353, A:G354, A:P355, A:G356, A:P357, A:G358, A:G359, A:R360, A:V361	85	0.662

JSmol-Rendered PDB Structure

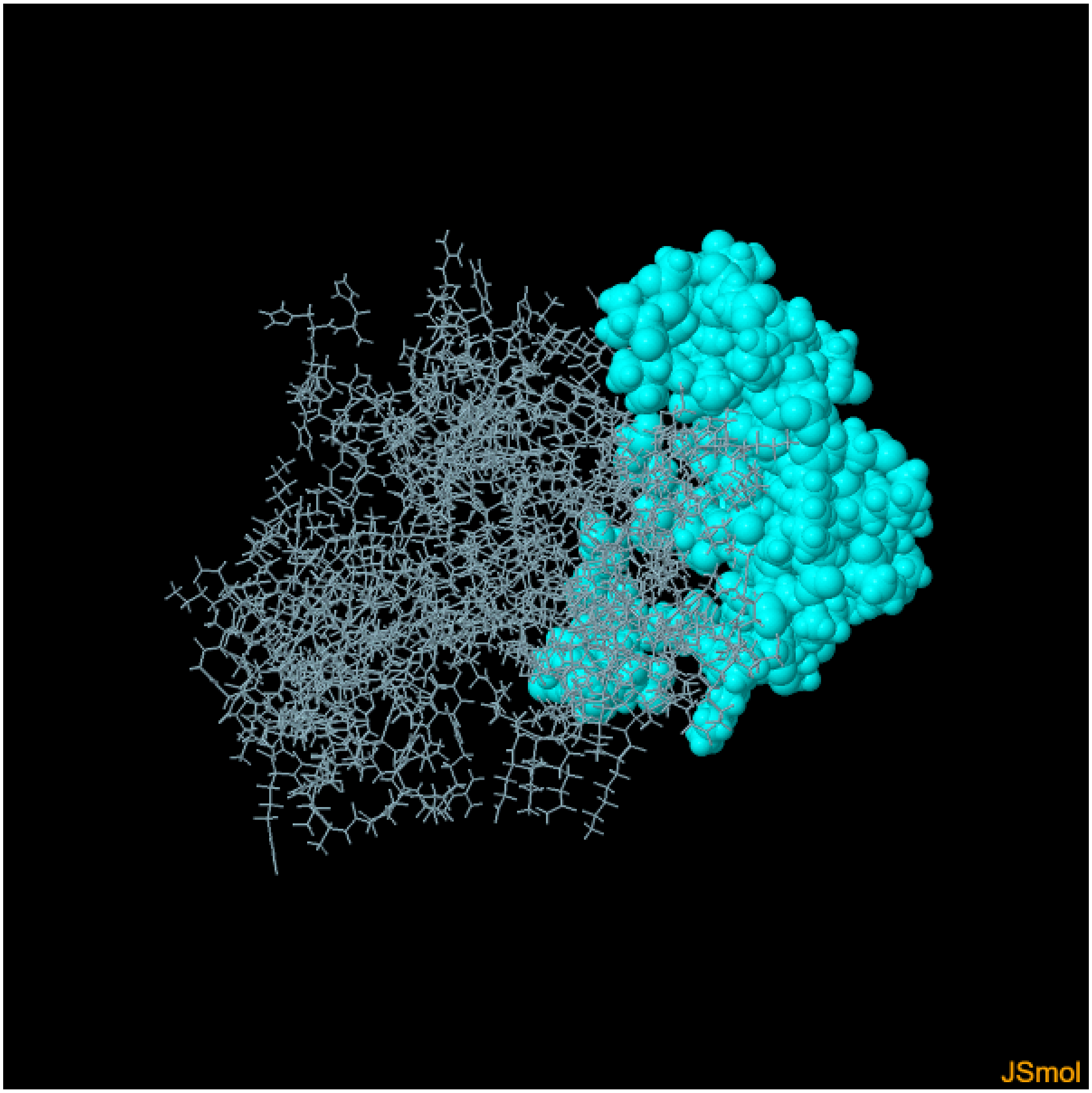

© 2005-2024 IEDB Home
