# Supplementary material for: Reverse vaccinology-based design of multivalent multiepitope mRNA vaccines targeting key viral proteins of Herpes Simplex Virus type-2
Source: Front Immunol. 2025 May 20;16:1586271. doi: 10.3389/fimmu.2025.1586271 (PMC12130045; doi:10.3389/fimmu.2025.1586271)
Supplement: Supplementary file 1 [file DataSheet1.zip › Supplementary Data_22-04-2025/Supplementary Data 2B - C2_2625.pdf]

ElliPro: Epitope 3D Structures for filev18k_cl4.pdb

No.	Residues	Number of residues	Score
1	A:P8, A:L9, A:V10, A:S11, A:S12, A:Q13, A:C14, A:V15, A:M16, A:A17, A:K18, A:L19, A:S20, A:T21, A:D22, A:E23, A:L24, A:L25, A:D26, A:A27, A:F28, A:K29, A:E30, A:M31, A:T32, A:L33, A:L34, A:E35, A:L36, A:S37, A:D38, A:F39, A:V40, A:F43, A:C183, A:S184, A:T185, A:R186, A:G187, A:R188, A:C190, A:C191, A:R192, A:R193, A:K194, A:K195, A:E196	47	0.776

JSmol-Rendered PDB Structure

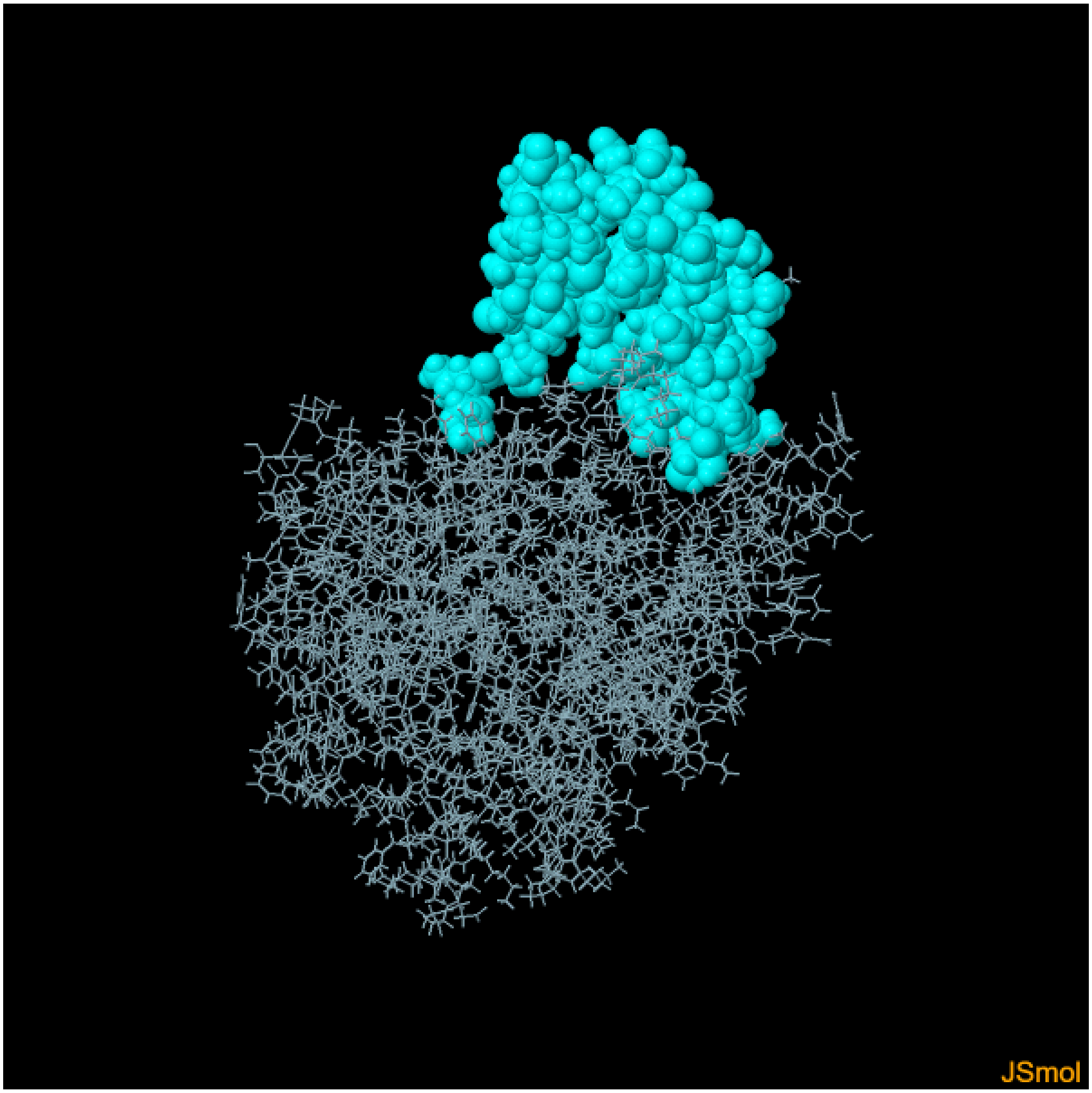

© 2005-2024 IEDB Home

ElliPro: Epitope 3D Structures for filev18k_cl4.pdb

No.	Residues	Number of residues	Score
2	A:W305, A:F308, A:S309	3	0.747

JSmol-Rendered PDB Structure

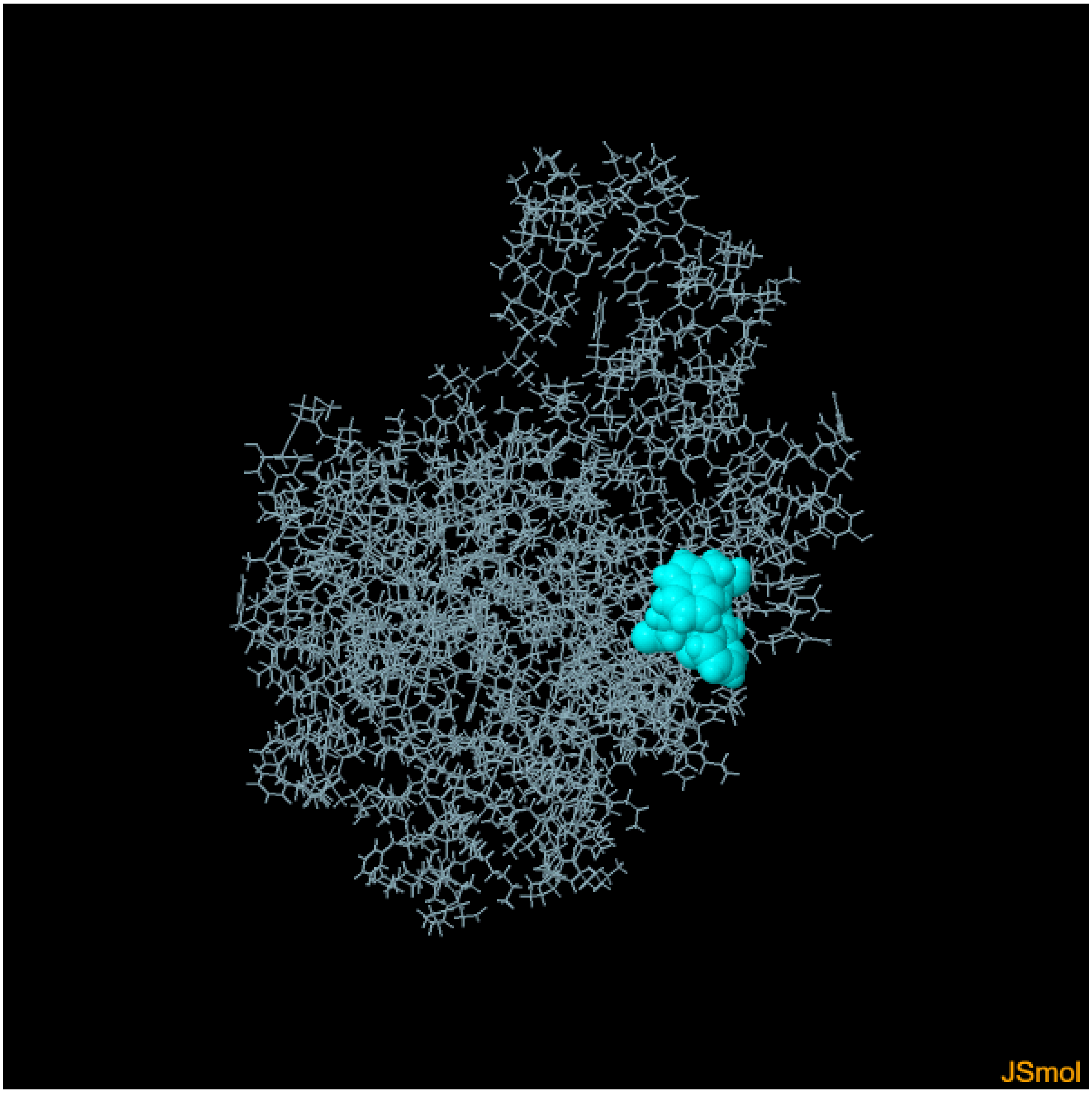

© 2005-2024 IEDB Home

ElliPro: Epitope 3D Structures for filev18k_cl4.pdb

No.	Residues	Number of residues	Score
3	A:Q75, A:S76, A:E77, A:F78, A:D79, A:V80, A:I81, A:L82, A:E83, A:A84, A:A85, A:G86, A:D87, A:K88, A:K89, A:I90, A:G91, A:V92, A:I93, A:K94, A:V95, A:V96, A:R97, A:E98, A:K109, A:D110, A:L111, A:V112, A:D113, A:G114, A:A115, A:P116, A:K117, A:P118, A:L119, A:L120, A:E121, A:K122, A:V123, A:A124, A:K125, A:E126, A:A128, A:D129, A:K132, A:A140, A:T141, A:V142, A:T143, A:V144, A:K145, A:E146	52	0.744

JSmol-Rendered PDB Structure

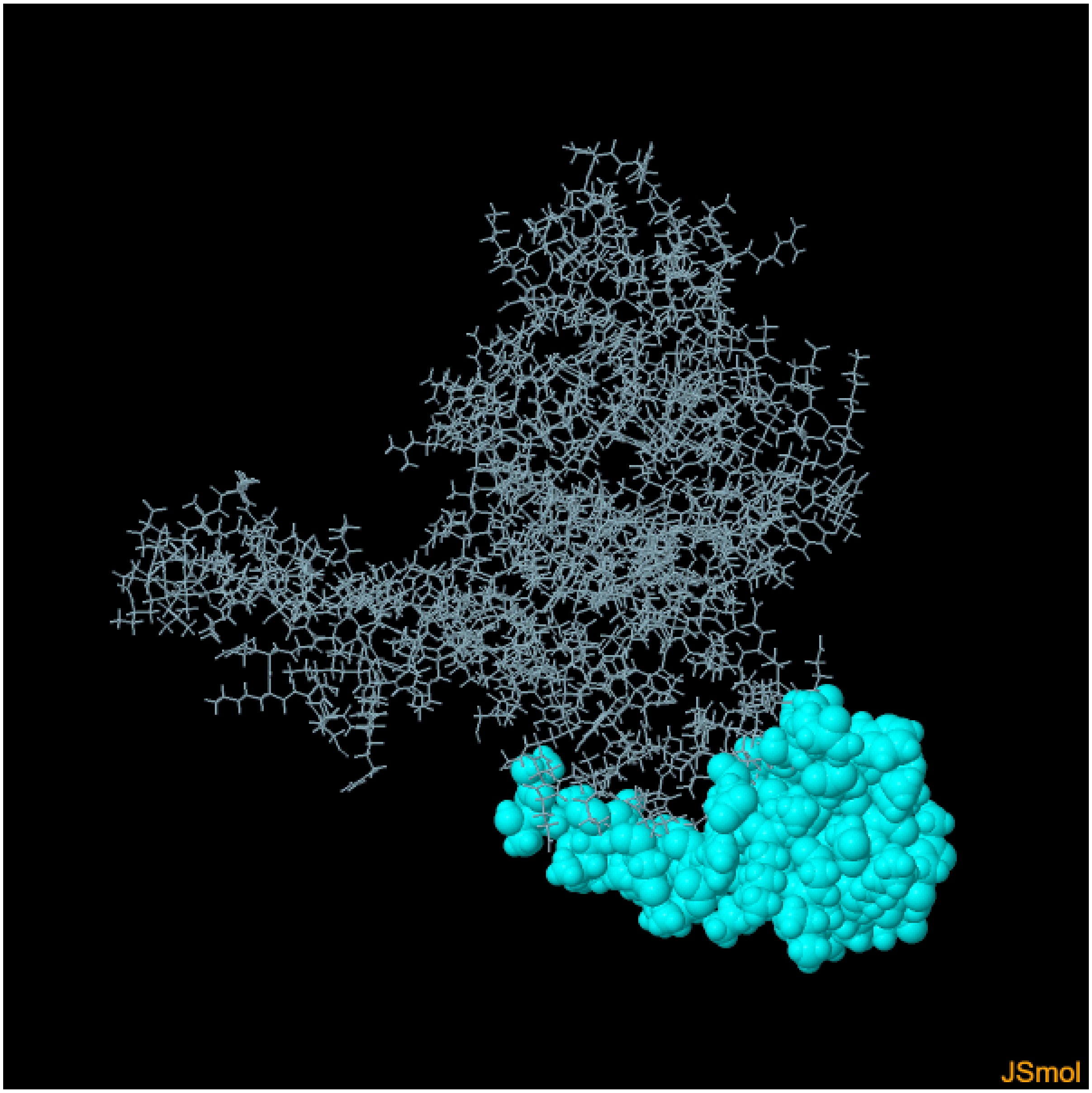

© 2005-2024 IEDB Home

ElliPro: Epitope 3D Structures for filev18k_cl4.pdb

No.	Residues	Number of residues	Score
4	A:K254, A:A255, A:R256, A:Y257, A:L330, A:E331, A:P332, A:G333, A:P334, A:G335, A:P336, A:G337, A:T338, A:M339, A:T340, A:K341, A:E344, A:V345, A:D346, A:E347, A:M348, A:L349, A:R350, A:A351, A:E352, A:Y353, A:G354, A:P355, A:G356, A:P357, A:G358, A:G359, A:R360, A:V361, A:V362, A:F363, A:L364, A:P365, A:T366, A:I367, A:R368, A:R369, A:Q370, A:L371, A:A372, A:L373, A:A374, A:E375, A:A376, A:A377, A:A378, A:K379, A:A380, A:K381, A:F382, A:V383, A:W386	57	0.718

JSmol-Rendered PDB Structure

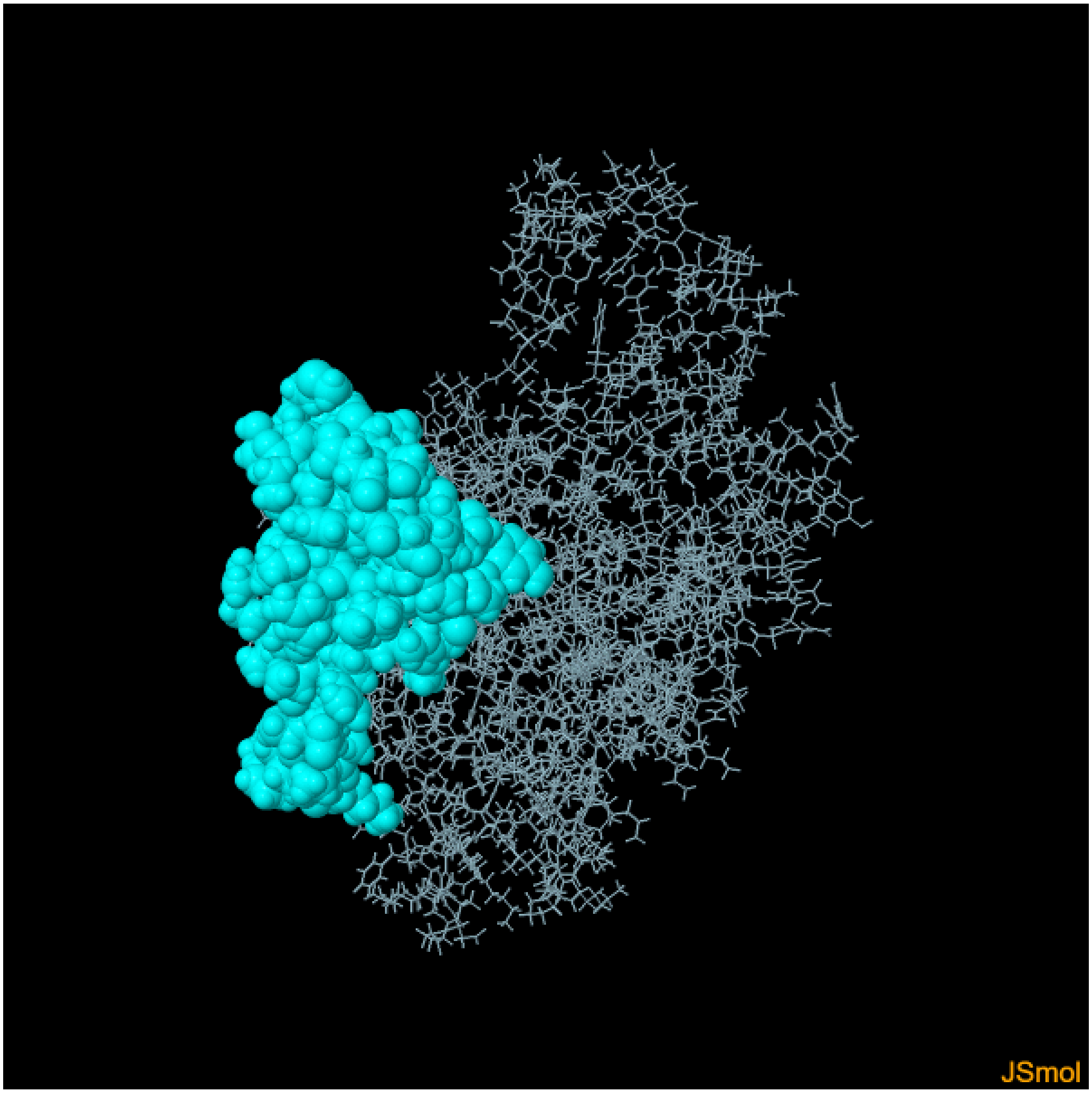

© 2005-2024 IEDB Home

ElliPro: Epitope 3D Structures for filev18k_cl4.pdb

No.	Residues	Number of residues	Score
5	A:D226, A:L227, A:D228, A:G230, A:L231, A:K232, A:K233, A:I285, A:W286, A:T287, A:G288, A:N289, A:Q290, A:R291, A:T292, A:A293, A:P294, A:R295, A:A296, A:A297, A:Y298, A:R299, A:A300, A:G301, A:R302, A:F303, A:H304, A:R307	28	0.705

JSmol-Rendered PDB Structure

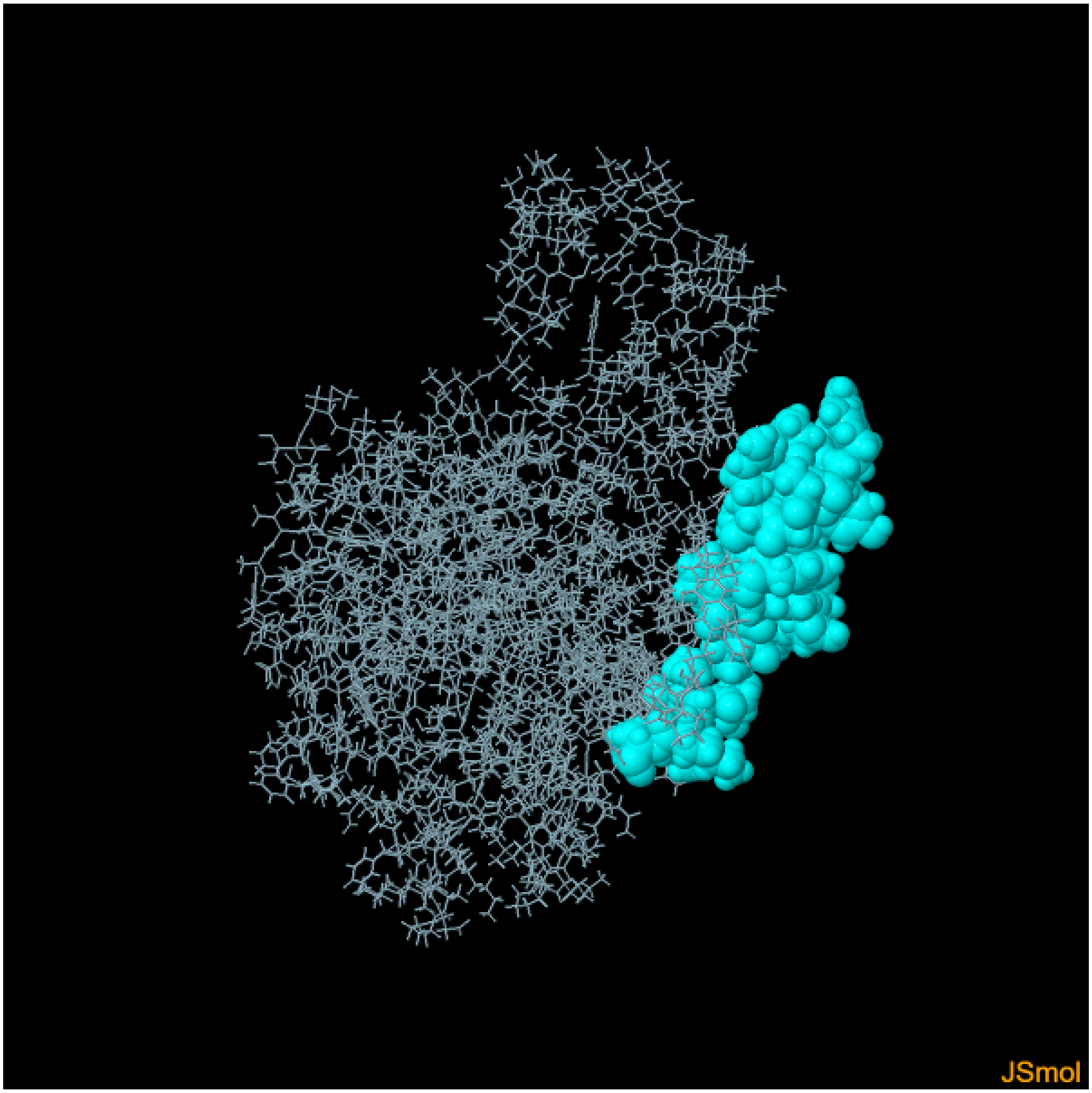

© 2005-2024 IEDB Home

ElliPro: Epitope 3D Structures for filev18k_cl4.pdb

No.	Residues	Number of residues	Score
6	A:A311, A:S312, A:P313, A:A314, A:A315, A:Y316, A:N317, A:K318, A:Q319	9	0.592

JSmol-Rendered PDB Structure

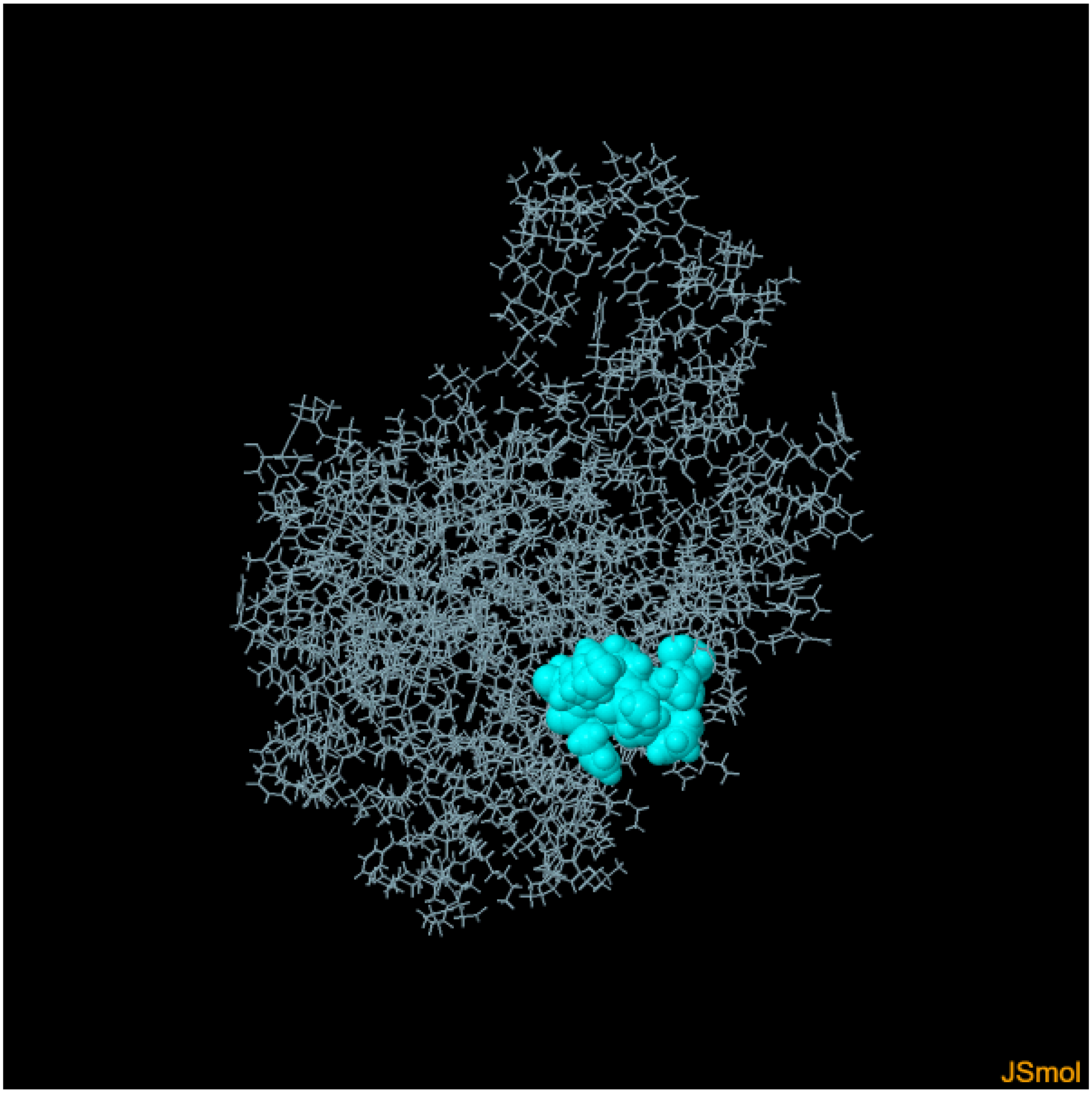

© 2005-2024 IEDB Home
