# Supplementary material for: Reverse vaccinology-based design of multivalent multiepitope mRNA vaccines targeting key viral proteins of Herpes Simplex Virus type-2
Source: Front Immunol. 2025 May 20;16:1586271. doi: 10.3389/fimmu.2025.1586271 (PMC12130045; doi:10.3389/fimmu.2025.1586271)
Supplement: Supplementary file 1 [file DataSheet1.zip › Supplementary Data_22-04-2025/Supplementary Data 2C - C3_735.pdf]

ElliPro: Epitope 3D Structures for filegcfd79qa.pdb

No.	Residues	Number of residues	Score
1	A:H395, A:H396, A:H397, A:H398	4	0.994

JSmol-Rendered PDB Structure

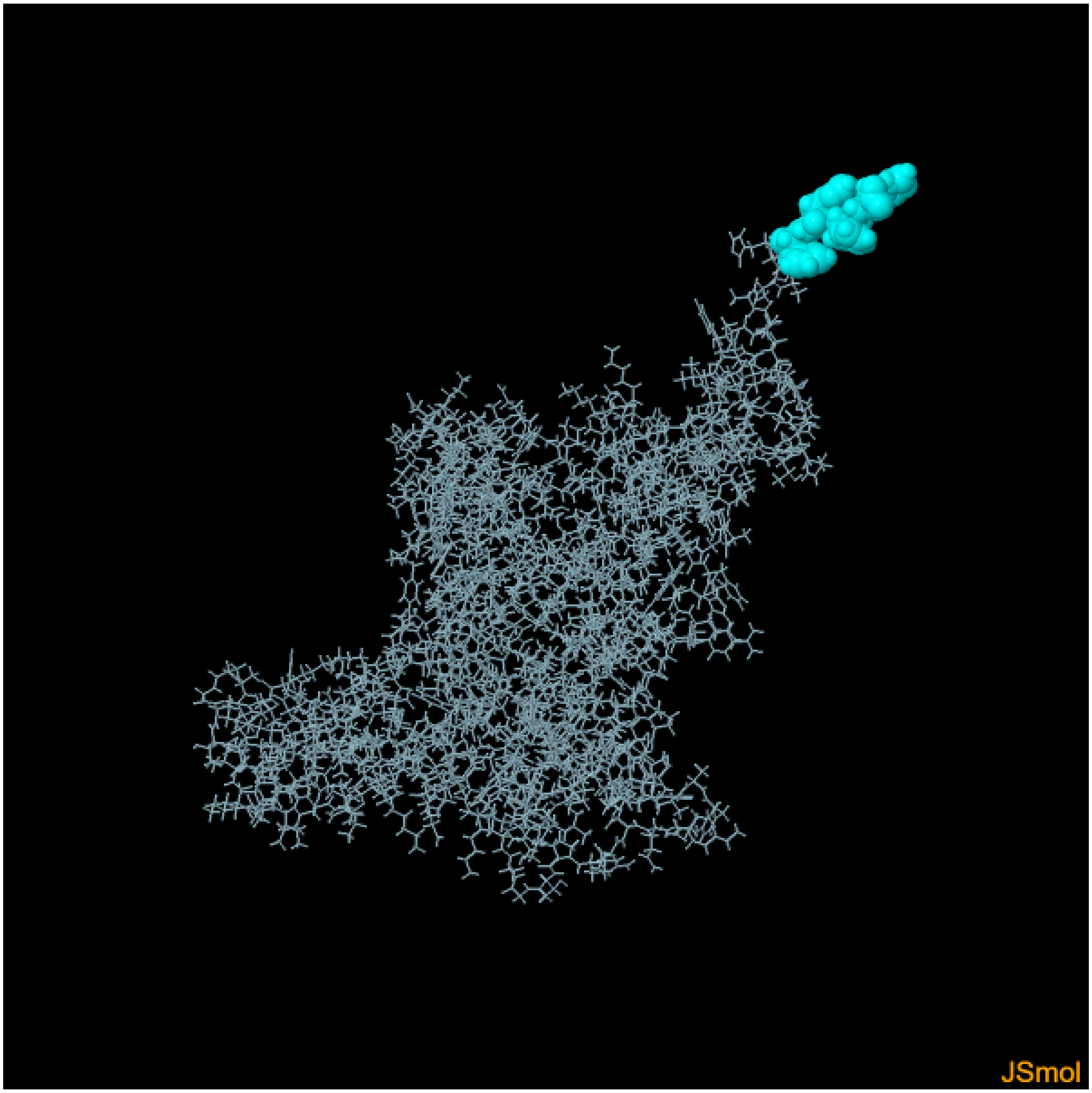

© 2005-2024 IEDB Home

ElliPro: Epitope 3D Structures for filegcfd79qa.pdb

No.	Residues	Number of residues	Score
2	A:Y257, A:S258, A:P259, A:A260, A:A261, A:Y262, A:A263, A:V264, A:D265, A:F266, A:I267, A:W268, A:T269, A:G270, A:N271, A:Q272, A:R273	17	0.798

JSmol-Rendered PDB Structure

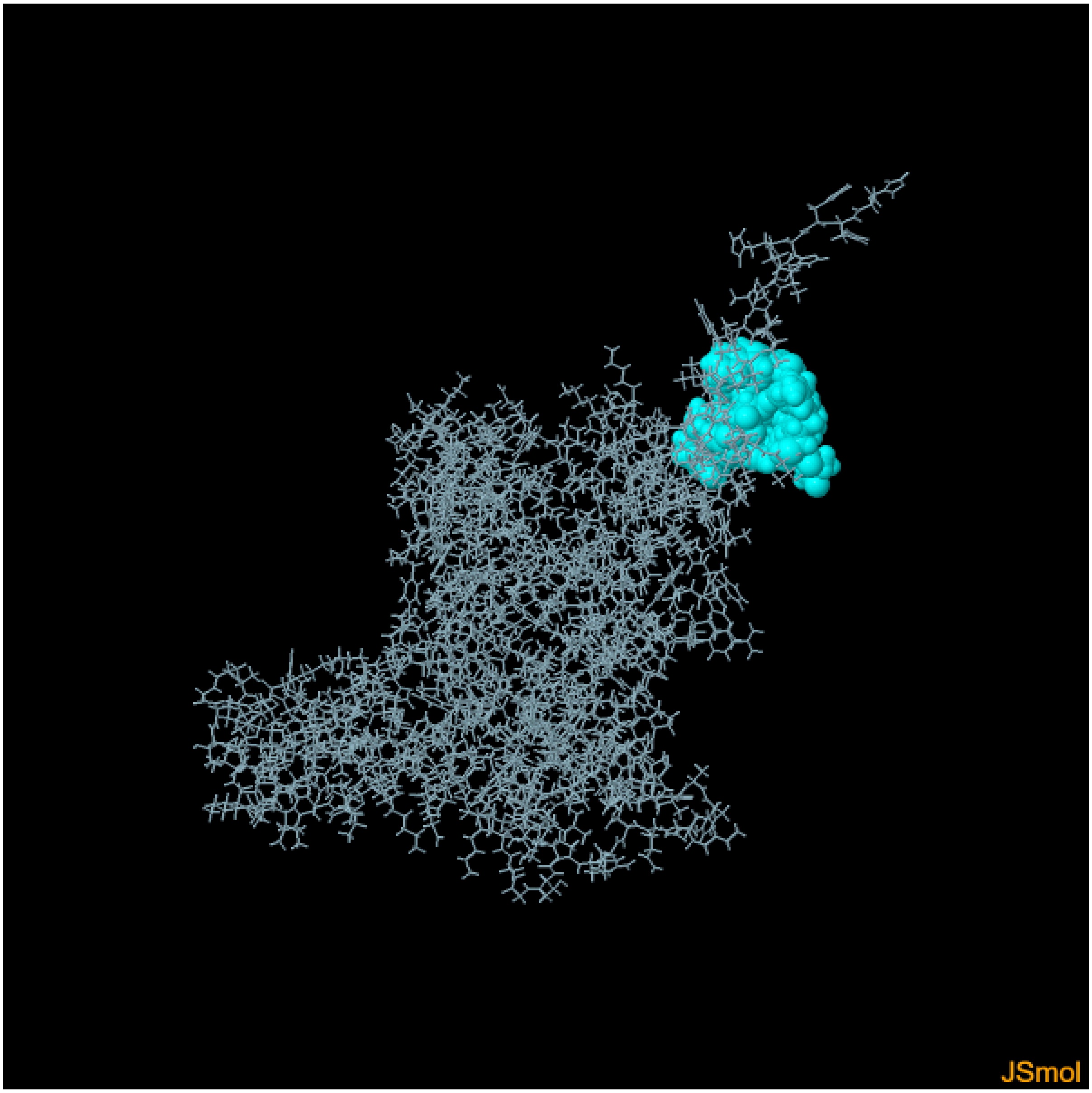

© 2005-2024 IEDB Home

ElliPro: Epitope 3D Structures for filegcfd79qa.pdb

No.	Residues	Number of residues	Score
3	A:T274, A:A275, A:P276, A:R277, A:A278, A:A279, A:A376, A:A377, A:A378, A:K379, A:A380, A:K381, A:F382, A:V383, A:A384, A:A385, A:W386, A:T387, A:L388, A:K389, A:A390, A:A391, A:A392, A:H393, A:H394	25	0.782

JSmol-Rendered PDB Structure

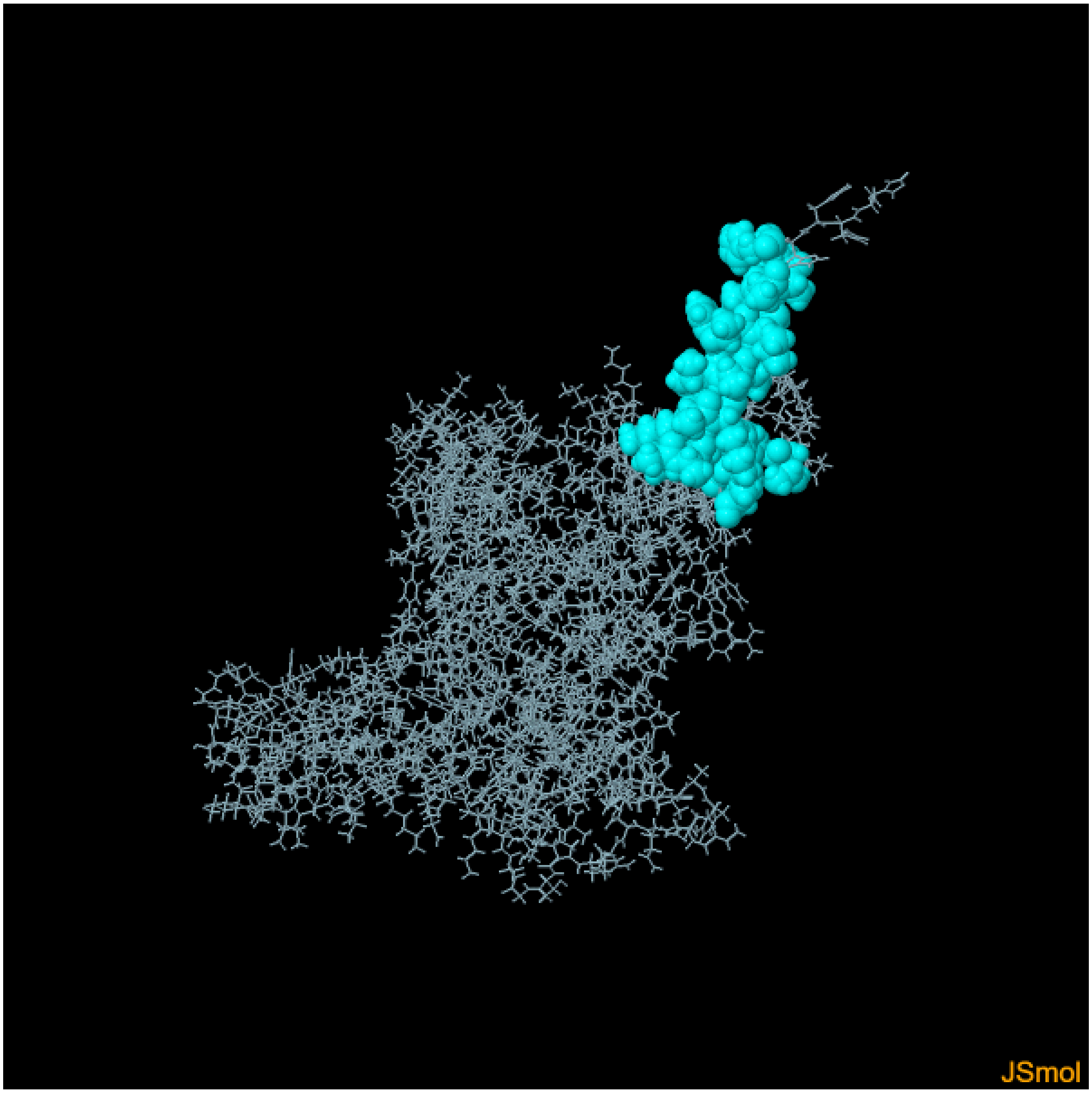

© 2005-2024 IEDB Home

ElliPro: Epitope 3D Structures for filegcfd79qa.pdb

No.	Residues	Number of residues	Score
4	A:D79, A:V80, A:L82, A:E83, A:A84, A:A85, A:G86, A:D87, A:K88, A:K89, A:I90, A:G91, A:V92, A:I93, A:K94, A:V95, A:V96, A:R97, A:E98, A:K125, A:E126, A:A128, A:D129, A:E130, A:A131, A:K132, A:A133, A:K134, A:L135, A:E136, A:A137, A:A138, A:G139, A:A140, A:T141, A:V142, A:T143, A:V144, A:K145, A:E146, A:A147, A:A148	42	0.762

JSmol-Rendered PDB Structure

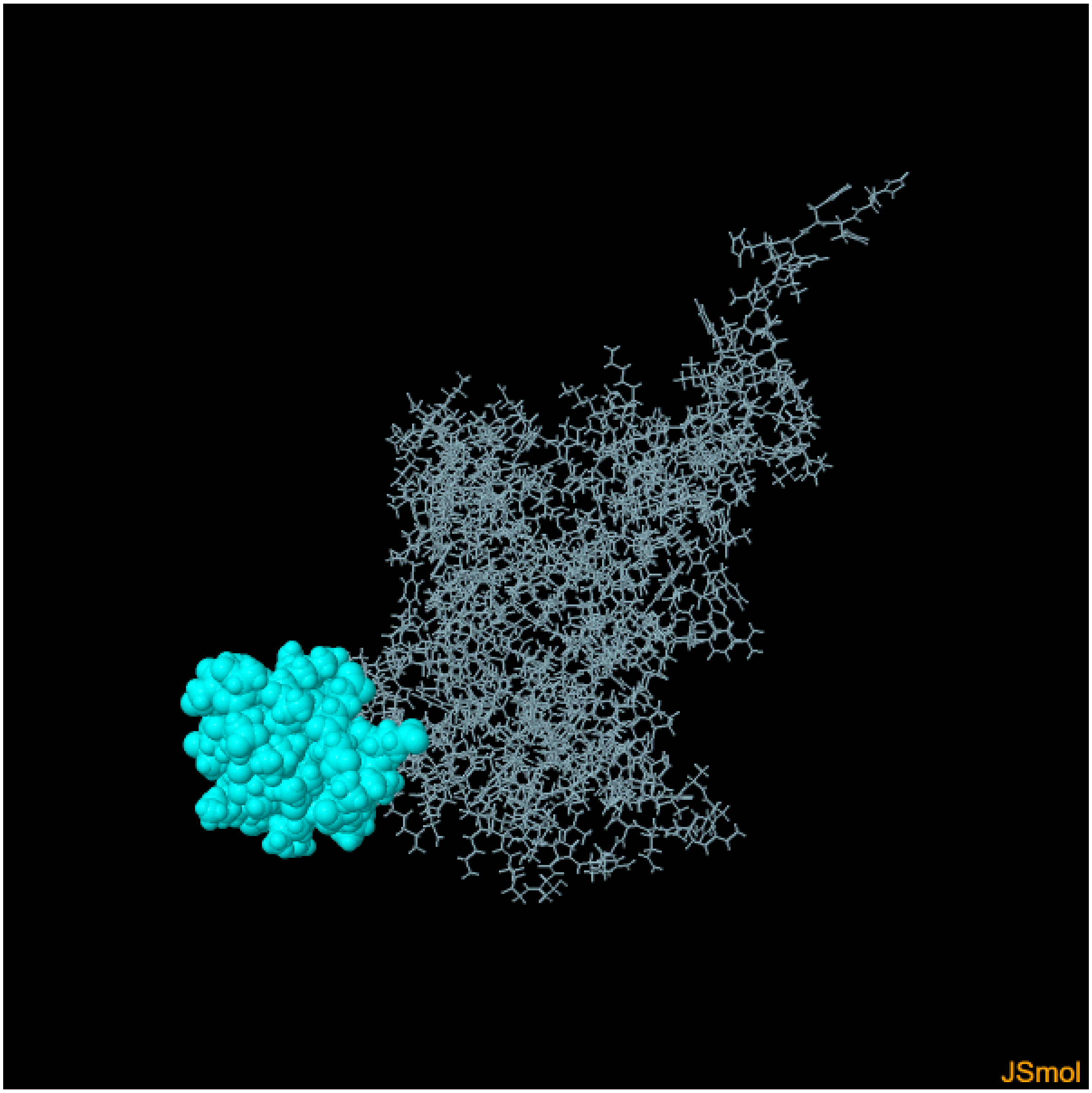

© 2005-2024 IEDB Home

ElliPro: Epitope 3D Structures for filegcfd79qa.pdb

No.	Residues	Number of residues	Score
5	A:C14, A:V15, A:M16, A:A17, A:K18, A:L19, A:S20, A:T21, A:D22, A:E23, A:L24, A:L25, A:D26, A:A27, A:F28, A:K29, A:E30, A:M31, A:T32, A:L33, A:E35, A:L36, A:L215, A:L216, A:L217, A:A218, A:Y219, A:R220, A:K221, A:K222, A:T224, A:A225, A:P226, A:R227, A:S228, A:L229, A:S230, A:L231, A:K232, A:K234, A:E235, A:V236, A:D237, A:L238, A:D239, A:F240, A:G241, A:L242, A:K243, A:K244, A:T245, A:N246, A:M247, A:R256	54	0.722

JSmol-Rendered PDB Structure

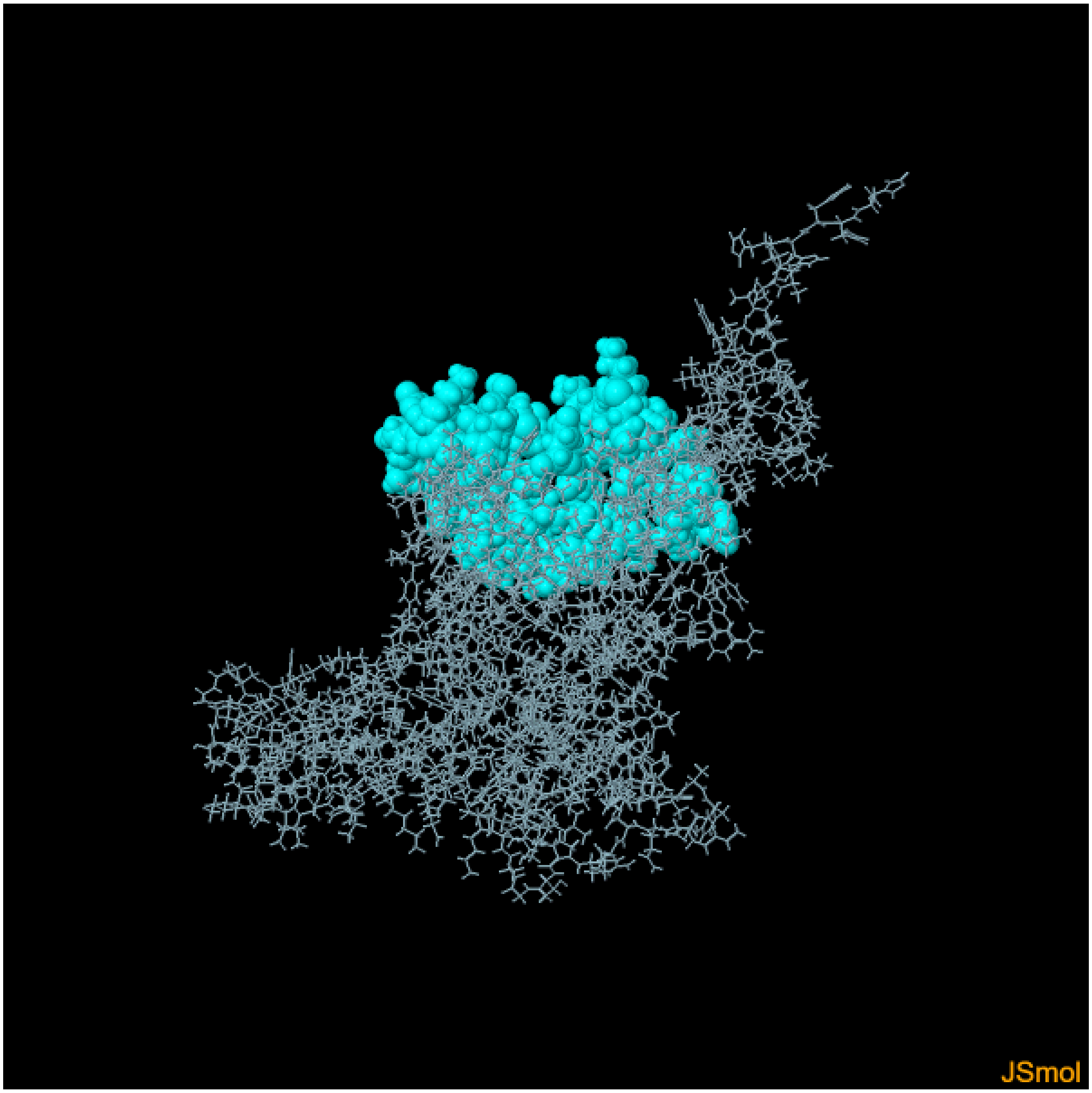

© 2005-2024 IEDB Home

ElliPro: Epitope 3D Structures for filegcfd79qa.pdb

No.	Residues	Number of residues	Score
6	A:K182, A:C183, A:S184, A:T185, A:R186, A:R188, A:R303, A:F304, A:P305, A:A306, A:V307, A:I308, A:T309, A:R310, A:V311, A:L312, A:P313, A:A314, A:A315, A:Y316, A:N317, A:K318, A:Q319, A:S320, A:T321, A:R322, A:G354, A:P355, A:G356, A:P357, A:G358, A:G359, A:R360, A:V361, A:V362, A:F363	36	0.681

JSmol-Rendered PDB Structure

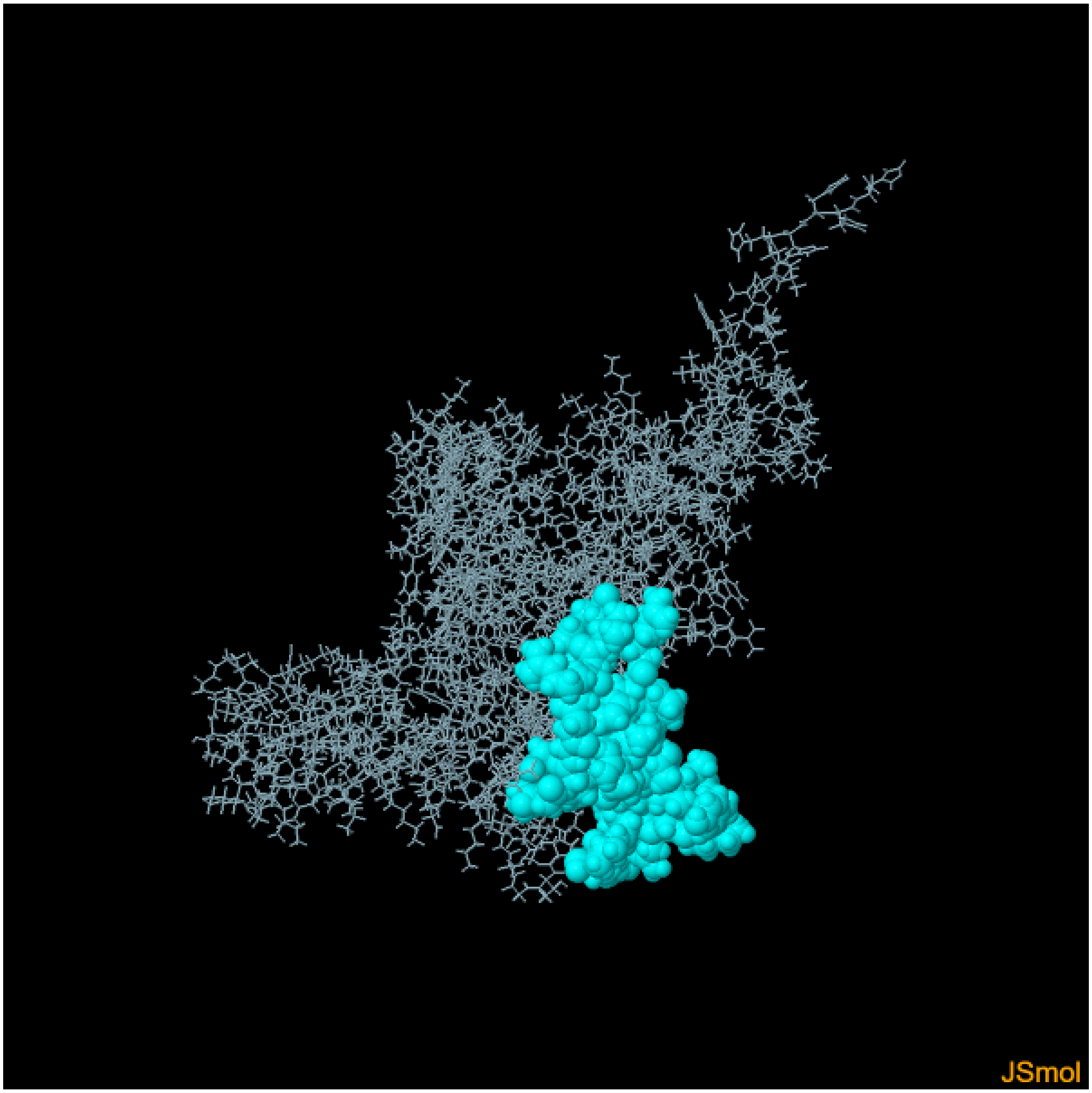

© 2005-2024 IEDB Home

ElliPro: Epitope 3D Structures for filegcfd79qa.pdb

No.	Residues	Number of residues	Score
7	A:W342, A:Q343, A:D346	3	0.606

JSmol-Rendered PDB Structure

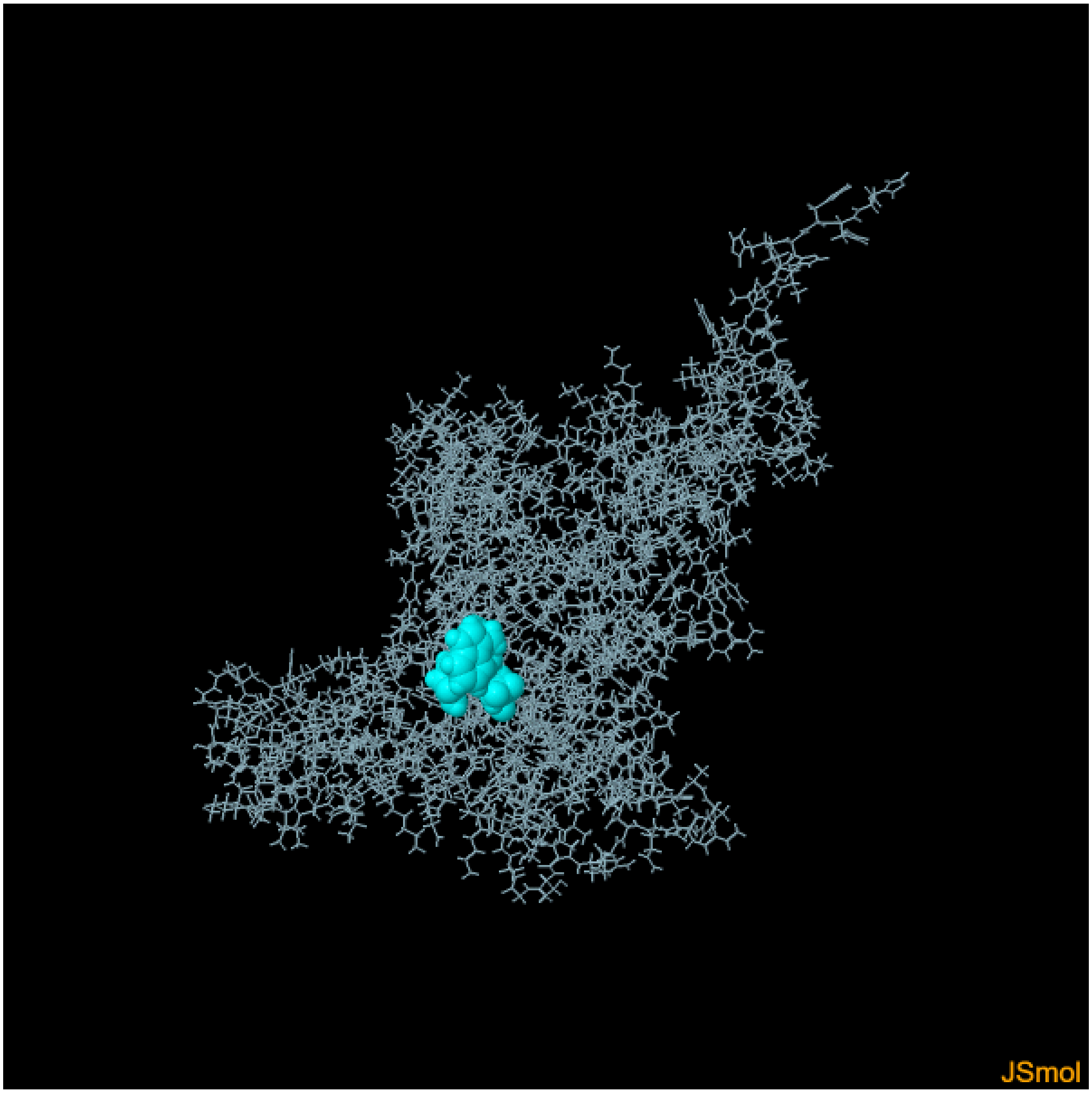

© 2005-2024 IEDB Home

ElliPro: Epitope 3D Structures for filegcfd79qa.pdb

No.	Residues	Number of residues	Score
8	A:R167, A:A169, A:V170, A:L171, A:S172, A:C173, A:L174, A:P175, A:K176, A:E177, A:E178, A:Q179, A:I180, A:G181, A:P323, A:T324, A:G325, A:A326, A:C327, A:V328	20	0.559

JSmol-Rendered PDB Structure

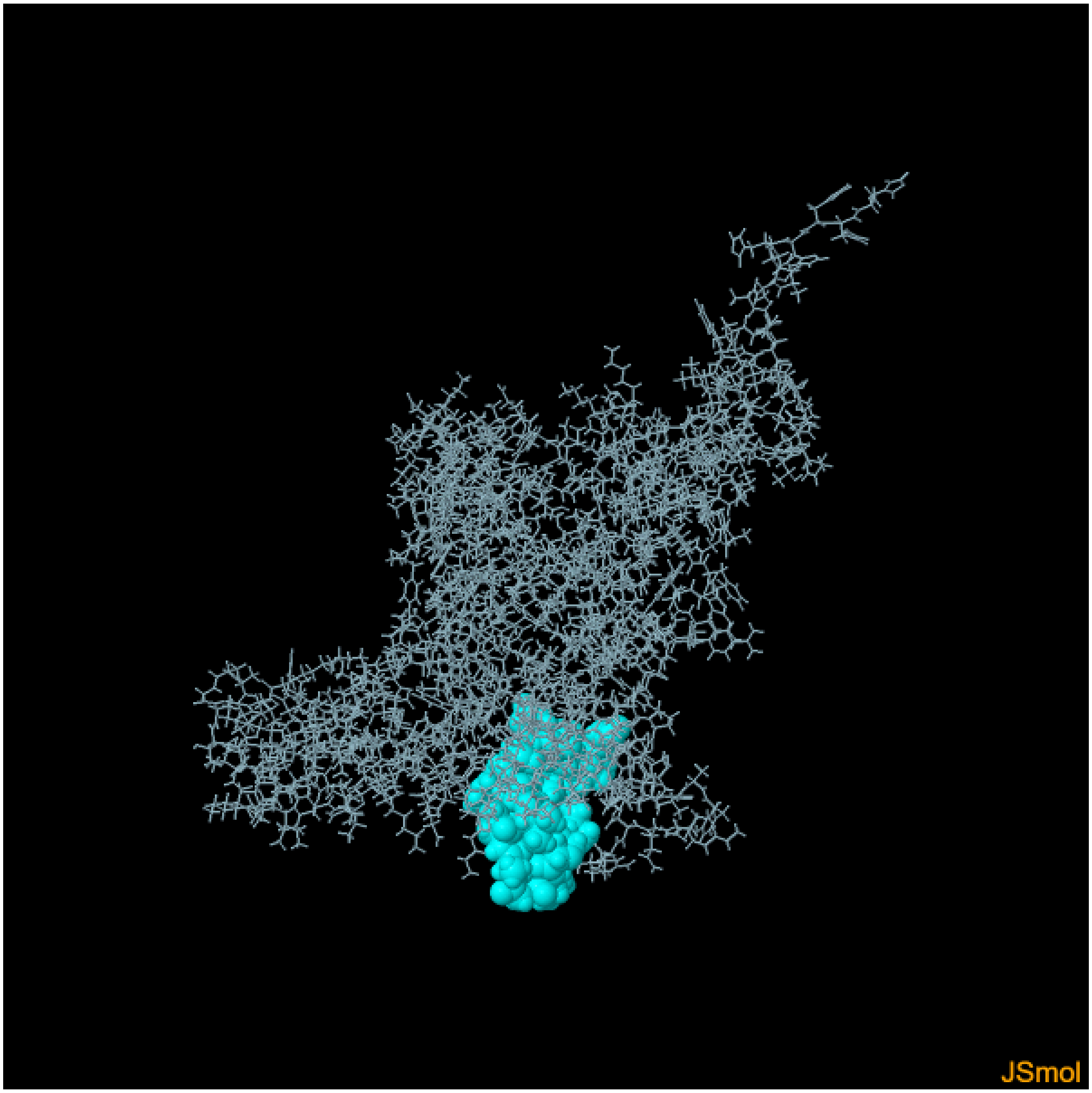

© 2005-2024 IEDB Home
