# Supplementary material for: Reverse vaccinology-based design of multivalent multiepitope mRNA vaccines targeting key viral proteins of Herpes Simplex Virus type-2
Source: Front Immunol. 2025 May 20;16:1586271. doi: 10.3389/fimmu.2025.1586271 (PMC12130045; doi:10.3389/fimmu.2025.1586271)
Supplement: Supplementary file 1 [file DataSheet1.zip › Supplementary Data_22-04-2025/Supplementary Data 2D - C4_2607.pdf]

ElliPro: Epitope 3D Structures for fileyf88rxxo.pdb

No.	Residues	Number of residues	Score
1	A:H395, A:H396, A:H397, A:H398	4	0.984

JSmol-Rendered PDB Structure

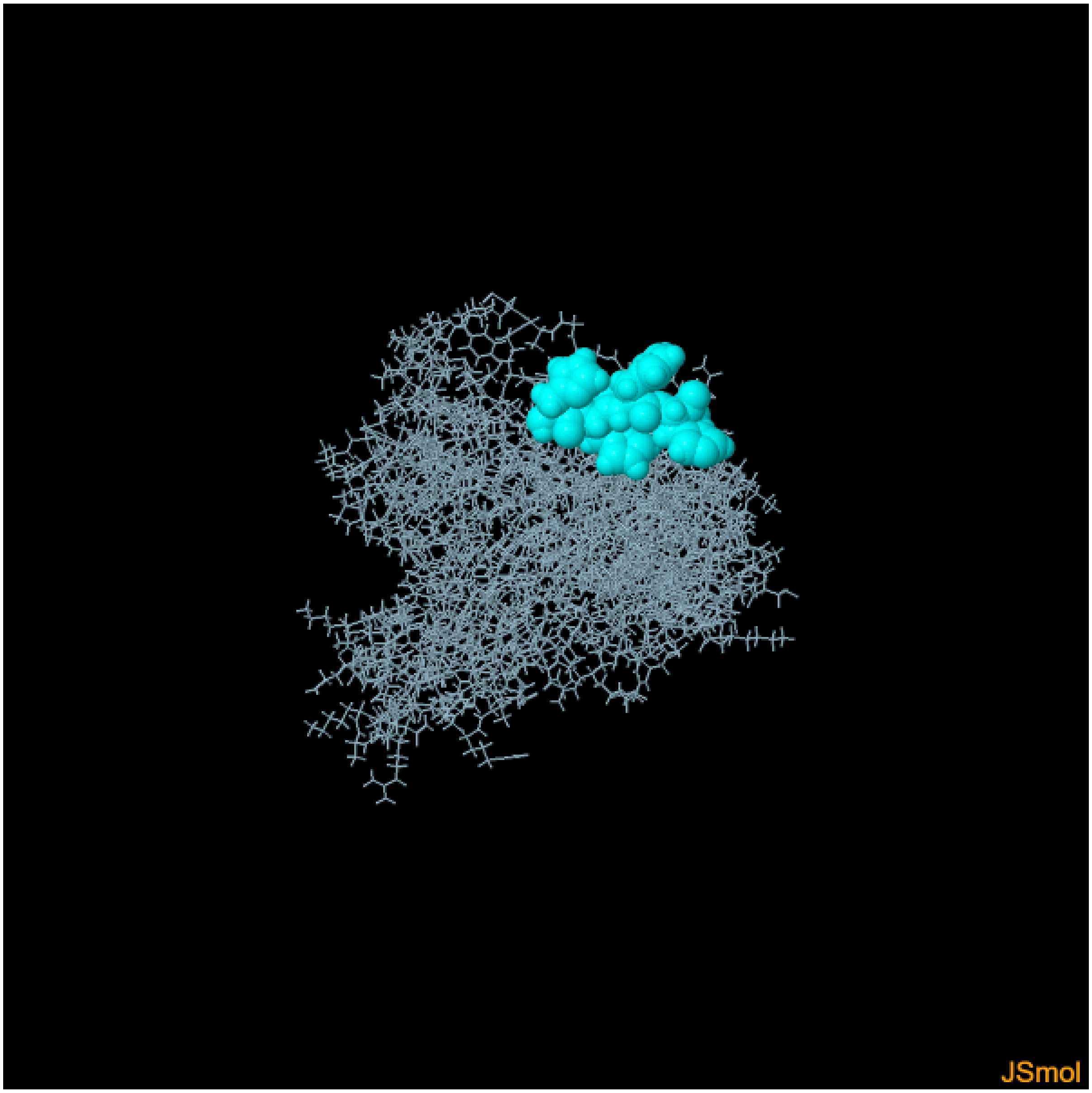

© 2005-2024 IEDB Home

ElliPro: Epitope 3D Structures for fileyf88rxxo.pdb

No.	Residues	Number of residues	Score
2	A:K132, A:A133, A:L135, A:E136, A:A137, A:A138, A:G139, A:A140, A:T141, A:V142, A:T143, A:V144, A:K145, A:E146, A:A147, A:K150, A:S172, A:C173, A:L174, A:P175, A:K176, A:E177, A:E178, A:Q179, A:I180, A:G181, A:K182, A:C183, A:S184, A:T185, A:R186, A:G187, A:R188, A:K189, A:C190, A:C191, A:R192, A:R193, A:K194, A:K195, A:E196, A:A197, A:A198, A:A199, A:K200, A:T201	46	0.789

JSmol-Rendered PDB Structure

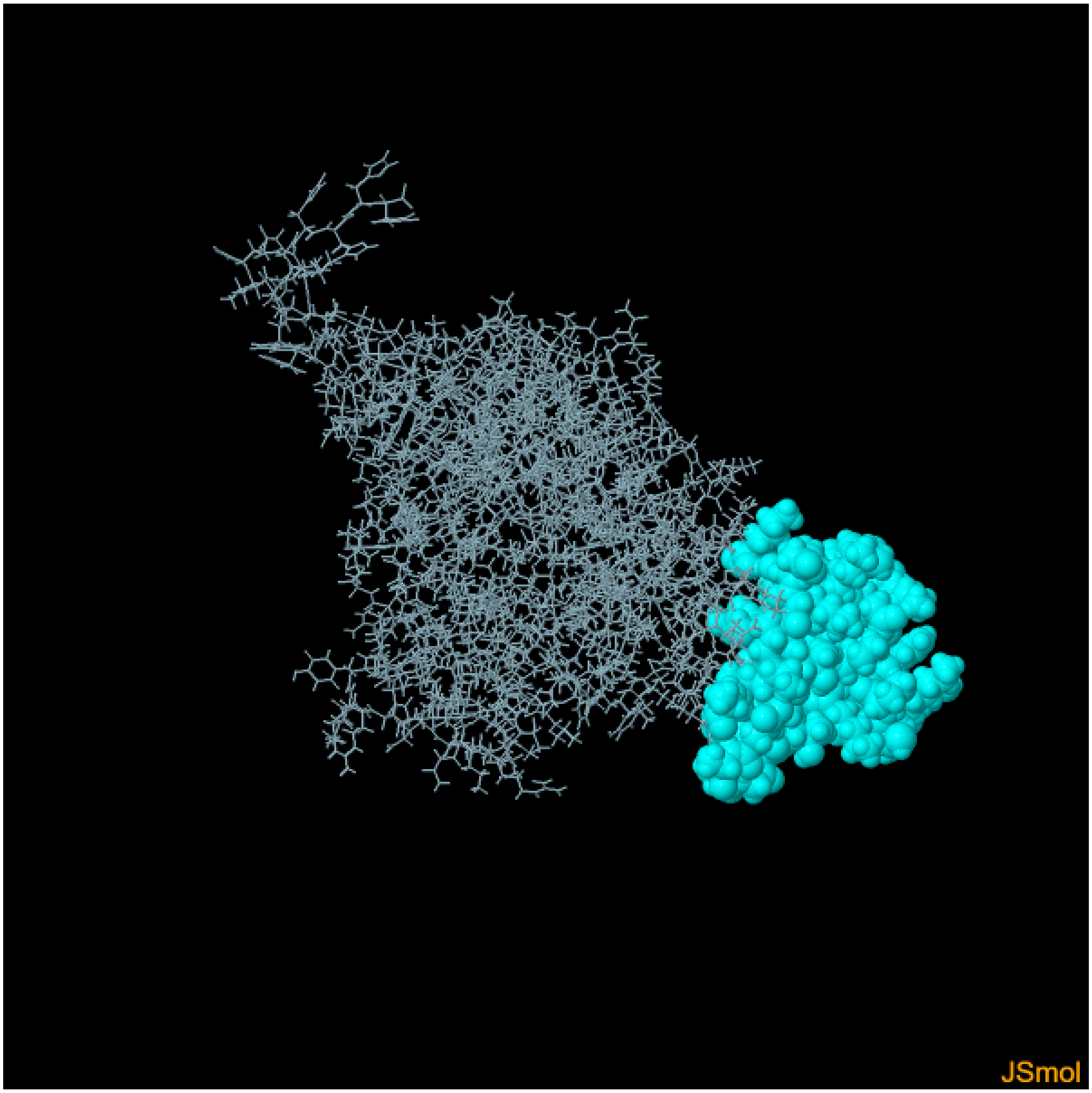

© 2005-2024 IEDB Home

ElliPro: Epitope 3D Structures for fileyf88rxxo.pdb

No.	Residues	Number of residues	Score
3	A:T32, A:L34, A:A378, A:K379, A:A380, A:K381, A:F382, A:V383, A:A384, A:A385, A:W386, A:T387, A:L388, A:K389, A:A390, A:A391, A:A392, A:H393, A:H394	19	0.78

JSmol-Rendered PDB Structure

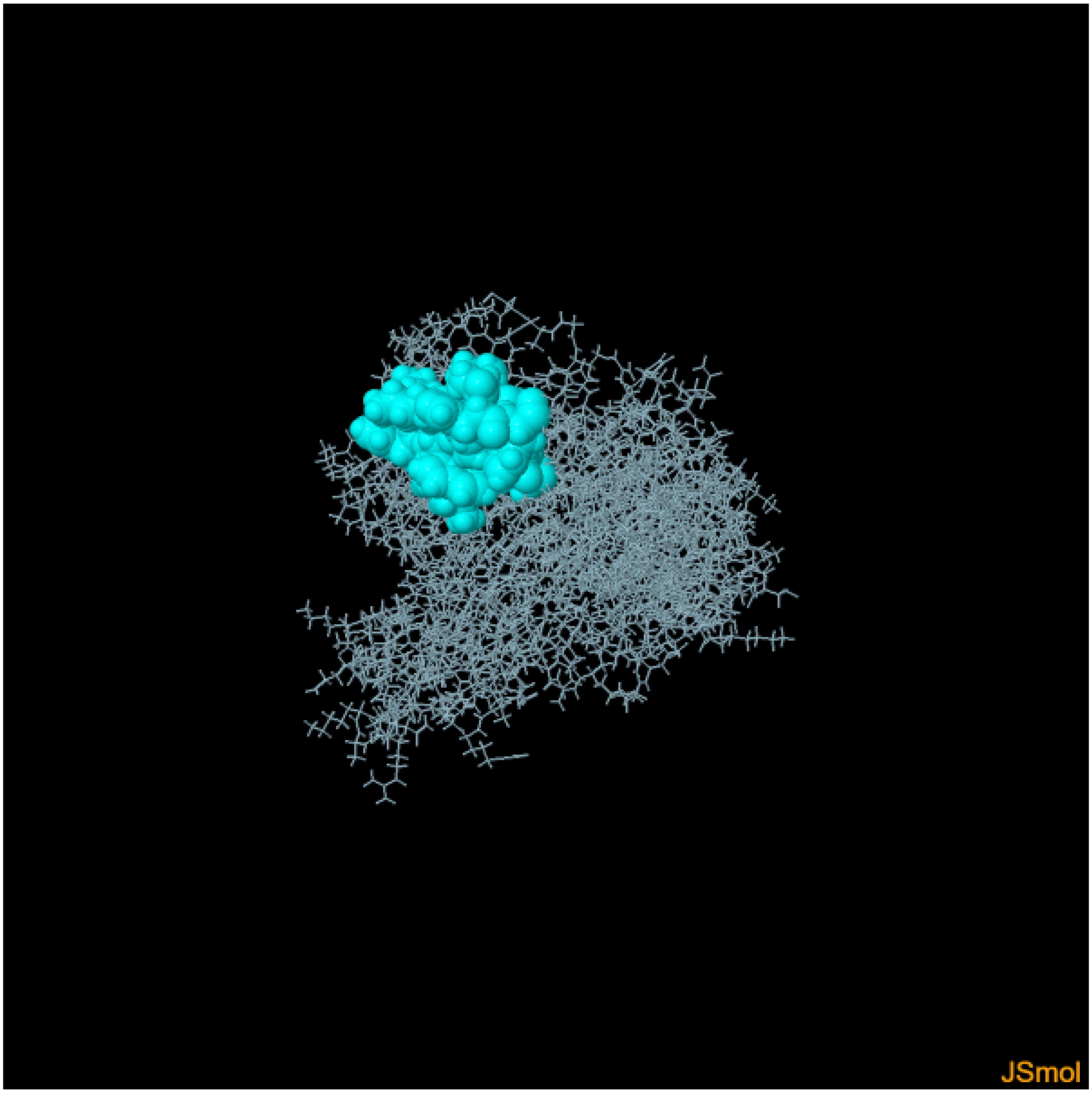

© 2005-2024 IEDB Home

ElliPro: Epitope 3D Structures for fileyf88rxxo.pdb

No.	Residues	Number of residues	Score
4	A:L4, A:P8, A:L9, A:V10, A:S11, A:S12, A:Q13, A:C14, A:V15, A:M16, A:A17, A:K18, A:L19, A:S20, A:T21, A:D22, A:E23, A:S37, A:D38, A:F39, A:V40, A:K41, A:K42, A:F43, A:E44, A:E45, A:T46, A:F47, A:E48, A:V49, A:T50, A:A51, A:A52, A:A53, A:P54, A:V55, A:A56, A:V57, A:A58, A:A59, A:A60, A:G61, A:A62	43	0.734

JSmol-Rendered PDB Structure

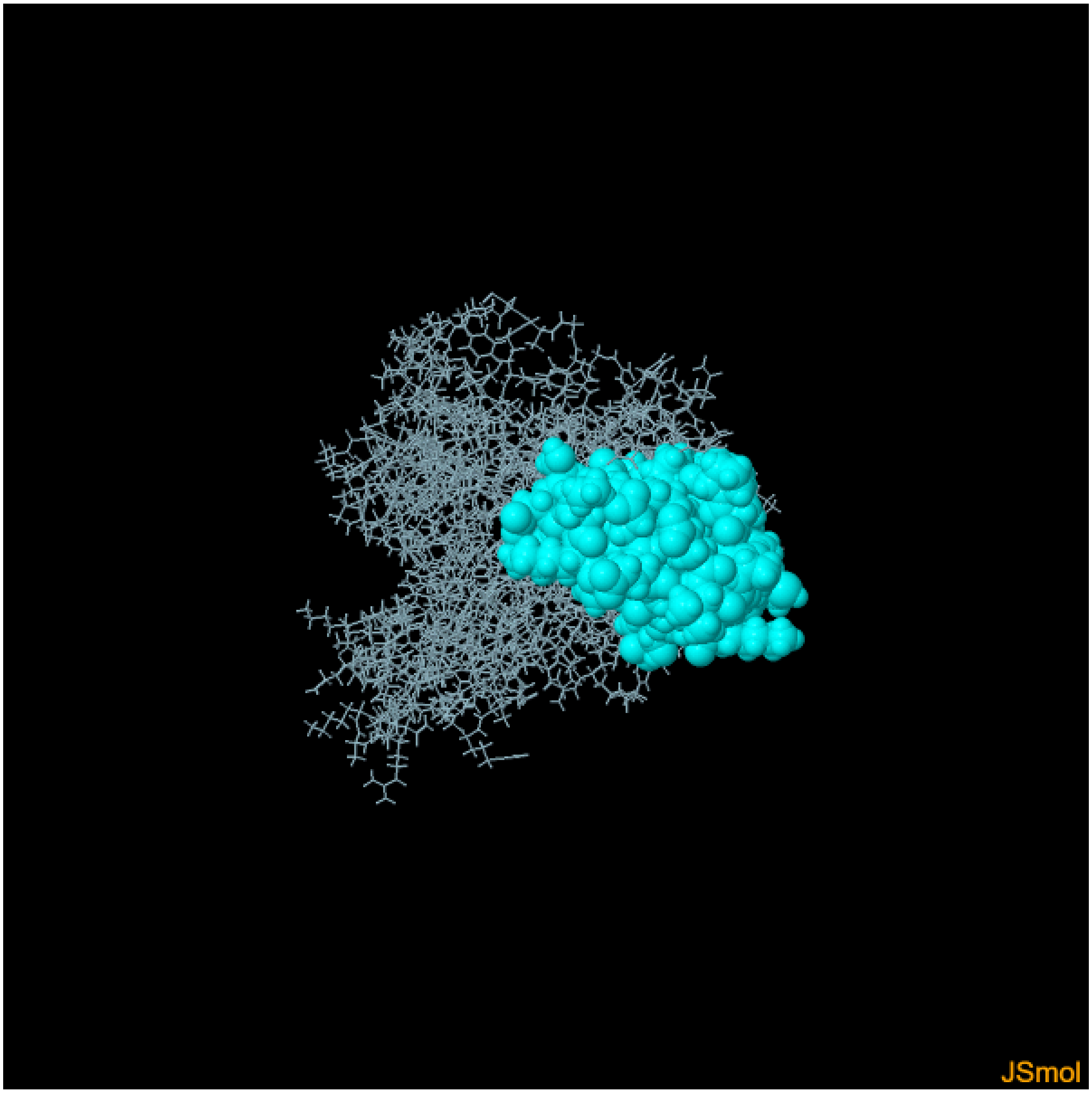

© 2005-2024 IEDB Home

ElliPro: Epitope 3D Structures for fileyf88rxxo.pdb

No.	Residues	Number of residues	Score
5	A:N154, A:K158, A:Y159, A:Y160, A:C161, A:R162, A:V163, A:R164, A:G165, A:G166, A:R167, A:C168, A:A169, A:D214, A:L215, A:T218, A:M219, A:L220, A:K221, A:K222, A:K223, A:V225, A:D226, A:L227, A:F229, A:G230, A:L231, A:K232, A:K233, A:R234, A:T235, A:A236, A:P237, A:M247, A:V248, A:L249, A:R250, A:K251, A:R252, A:N253, A:K254, A:A255, A:R256, A:S258, A:P259, A:A260, A:Y262, A:P276, A:A278, A:A279, A:R281, A:A282, A:G283, A:R284, A:R322, A:P323, A:T324, A:G325, A:A326, A:C327, A:V328, A:Y329, A:L330, A:E331, A:P332, A:G333, A:P334, A:G335, A:P336, A:G337, A:T338, A:M339, A:T340, A:K341, A:W342, A:Q343, A:E344, A:D346, A:E347, A:R350, A:A351, A:G354, A:P355, A:G356, A:P357, A:G358, A:G359, A:R360, A:V361, A:V362, A:F363, A:L364	92	0.644

JSmol-Rendered PDB Structure

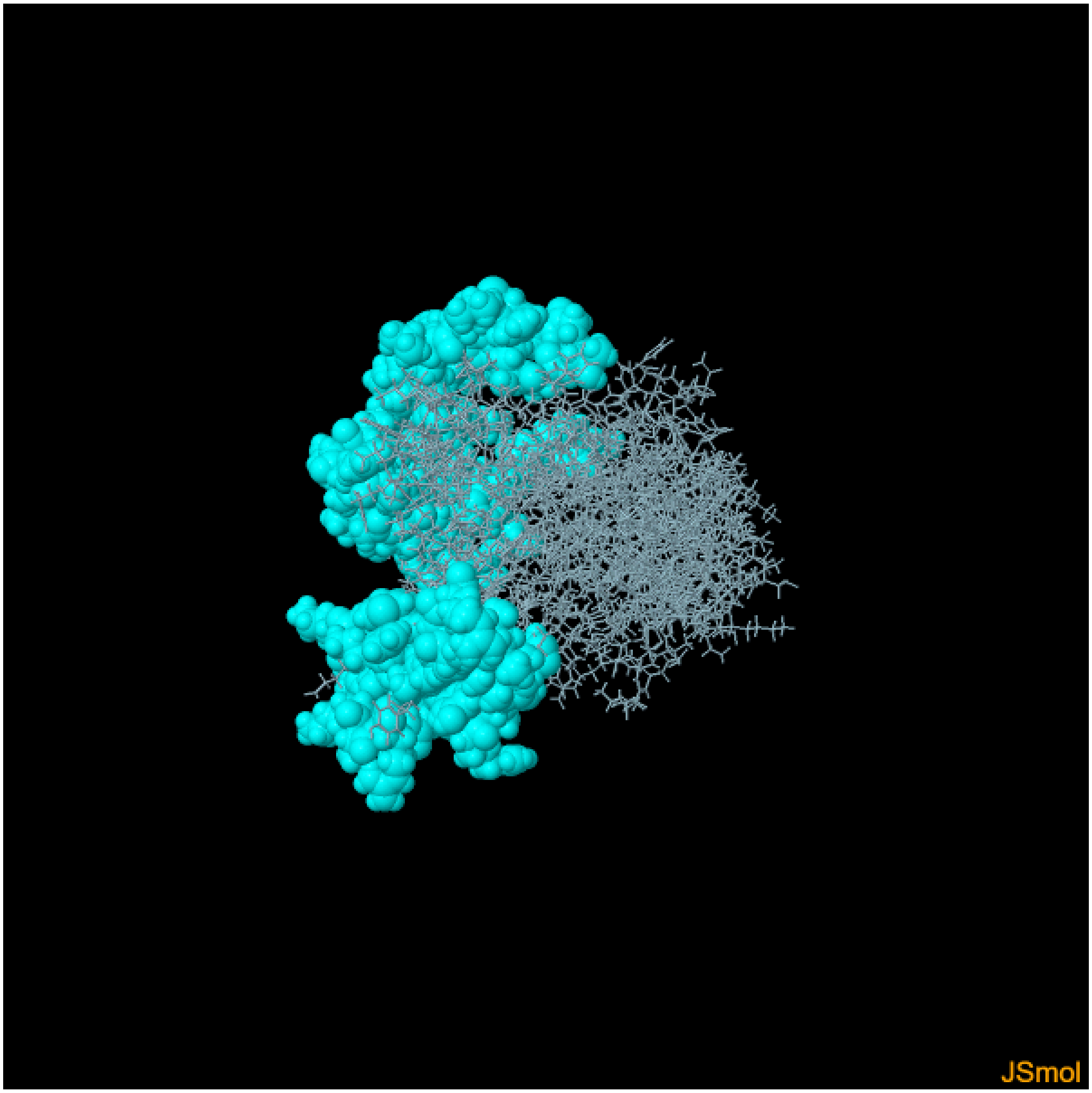

© 2005-2024 IEDB Home

ElliPro: Epitope 3D Structures for fileyf88rxxo.pdb

No.	Residues	Number of residues	Score
6	A:E74, A:Q75, A:S76, A:E77	4	0.587

JSmol-Rendered PDB Structure

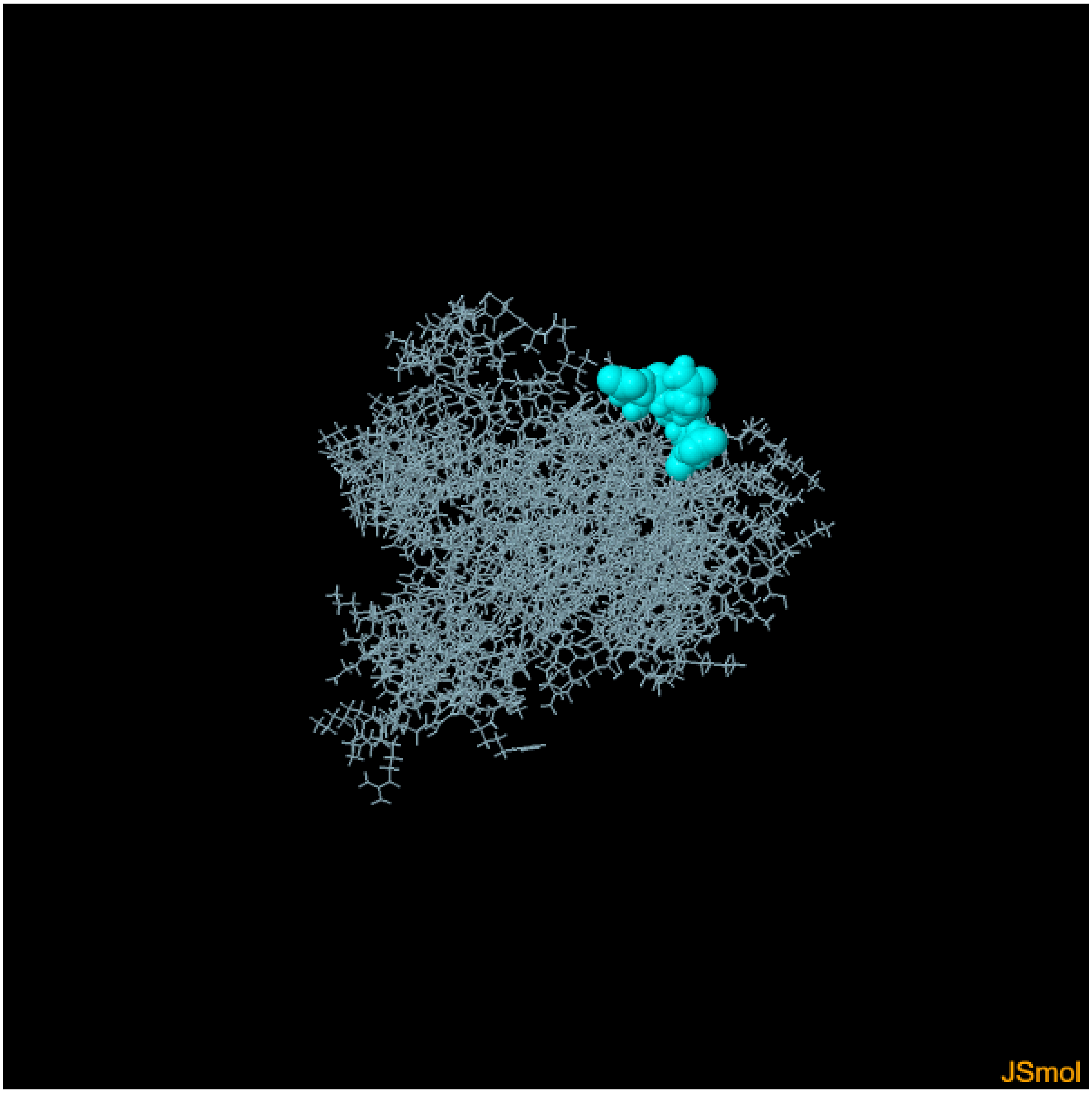

© 2005-2024 IEDB Home
