# Supplementary material for: Reverse vaccinology-based design of multivalent multiepitope mRNA vaccines targeting key viral proteins of Herpes Simplex Virus type-2
Source: Front Immunol. 2025 May 20;16:1586271. doi: 10.3389/fimmu.2025.1586271 (PMC12130045; doi:10.3389/fimmu.2025.1586271)
Supplement: Supplementary file 1 [file DataSheet1.zip › Supplementary Data_22-04-2025/Supplementary Data 2E - C5_2769.pdf]

ElliPro: Epitope 3D Structures for fileiqtdbq6t.pdb

No.	Residues	Number of residues	Score
1	A:K176, A:E177, A:E178, A:Q179, A:I180, A:G181, A:K182, A:C183, A:S184, A:T185, A:R186, A:G187, A:R188, A:P332, A:G333, A:P334, A:G335, A:P336, A:M348, A:L349, A:A351, A:E352, A:Y353, A:G354, A:P355, A:G356, A:P357, A:G358, A:G359, A:R360, A:V361, A:V362, A:F363, A:L364, A:P365, A:T366, A:I367, A:R368, A:Q370	39	0.759

JSmol-Rendered PDB Structure

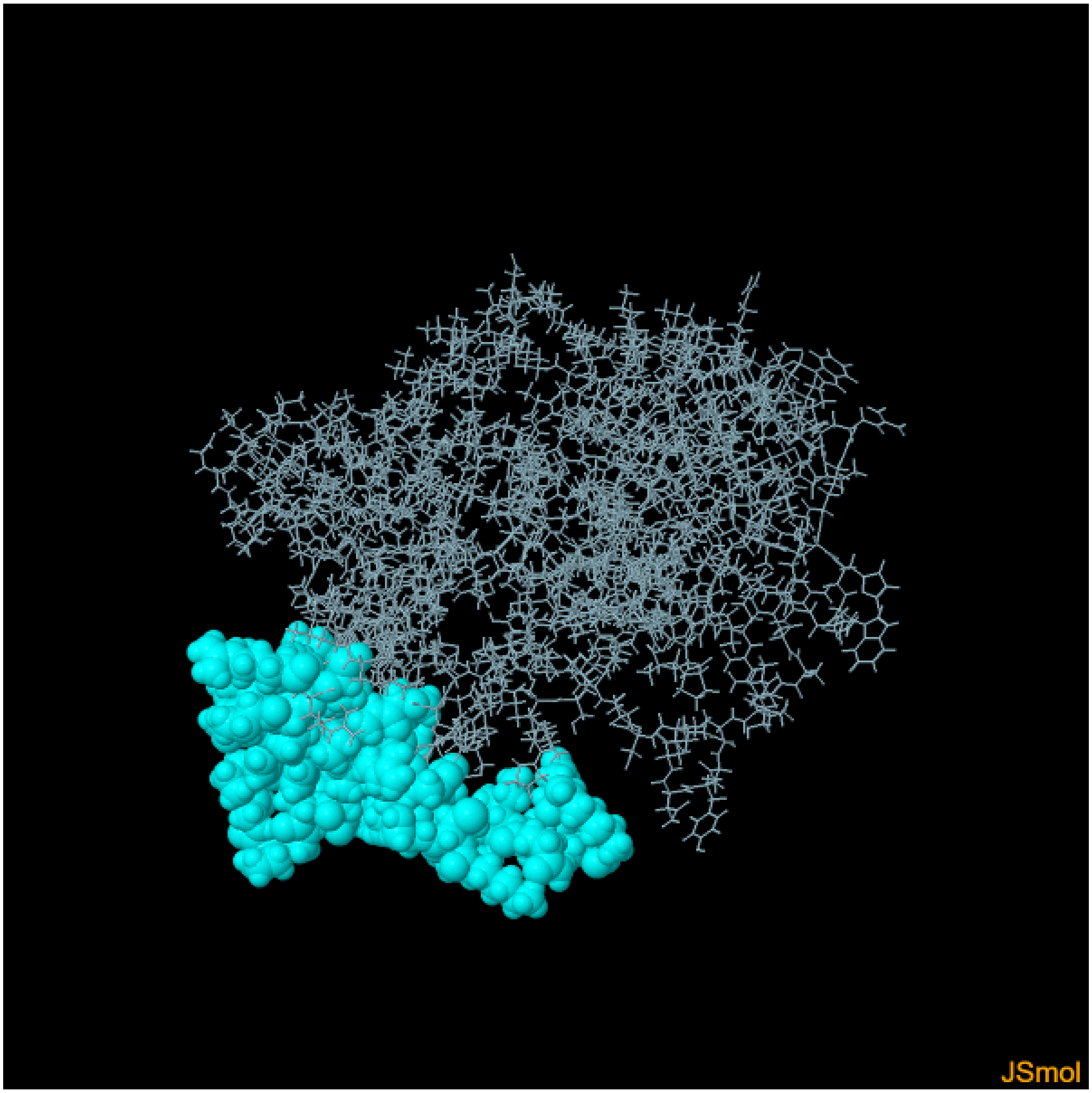

© 2005-2024 IEDB Home

ElliPro: Epitope 3D Structures for fileiqtdbq6t.pdb

No.	Residues	Number of residues	Score
2	A:R252, A:N253, A:K254, A:A255, A:R256, A:Y257, A:S258, A:P259, A:Y262, A:S309, A:N310, A:A311, A:S312, A:P313, A:A314, A:A315, A:Y316, A:N317, A:K318, A:Q319, A:T321	21	0.728

JSmol-Rendered PDB Structure

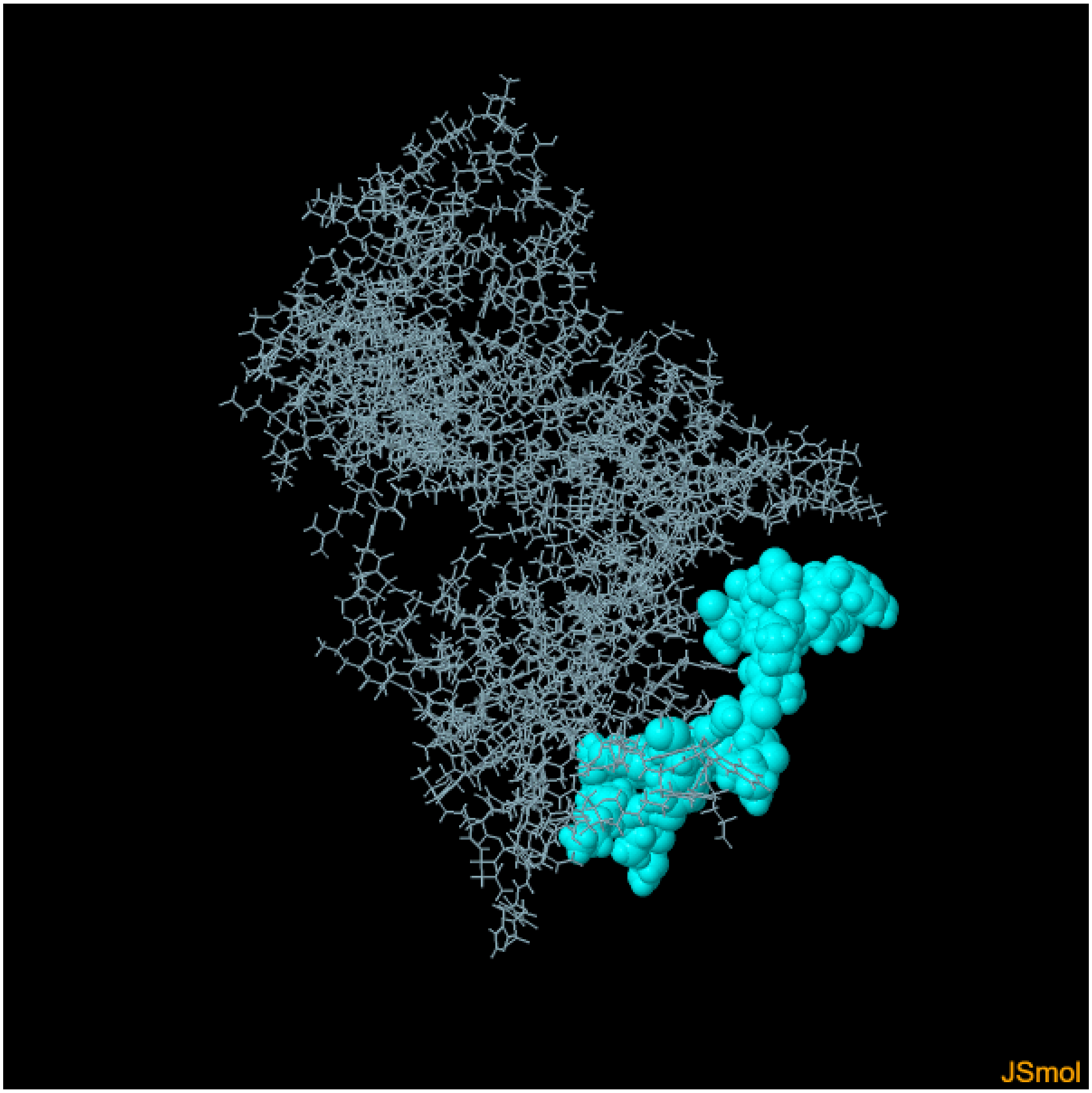

© 2005-2024 IEDB Home

ElliPro: Epitope 3D Structures for fileiqtdbq6t.pdb

No.	Residues	Number of residues	Score
3	A:F1, A:V2, A:F3, A:L4, A:V5, A:L6, A:L7, A:P8, A:L9, A:V10, A:S11, A:S12, A:Q13, A:C14, A:V15, A:M16, A:A17, A:K18, A:L19, A:M31, A:T32, A:L34, A:E35, A:S37, A:D38, A:F39, A:V40, A:K41, A:K42, A:F43, A:E44, A:E45, A:T46, A:F47, A:E48, A:V49, A:T50, A:A51, A:A52, A:A53, A:P54, A:V55, A:E83, A:A84, A:A85, A:G86, A:D87, A:K88	48	0.723

JSmol-Rendered PDB Structure

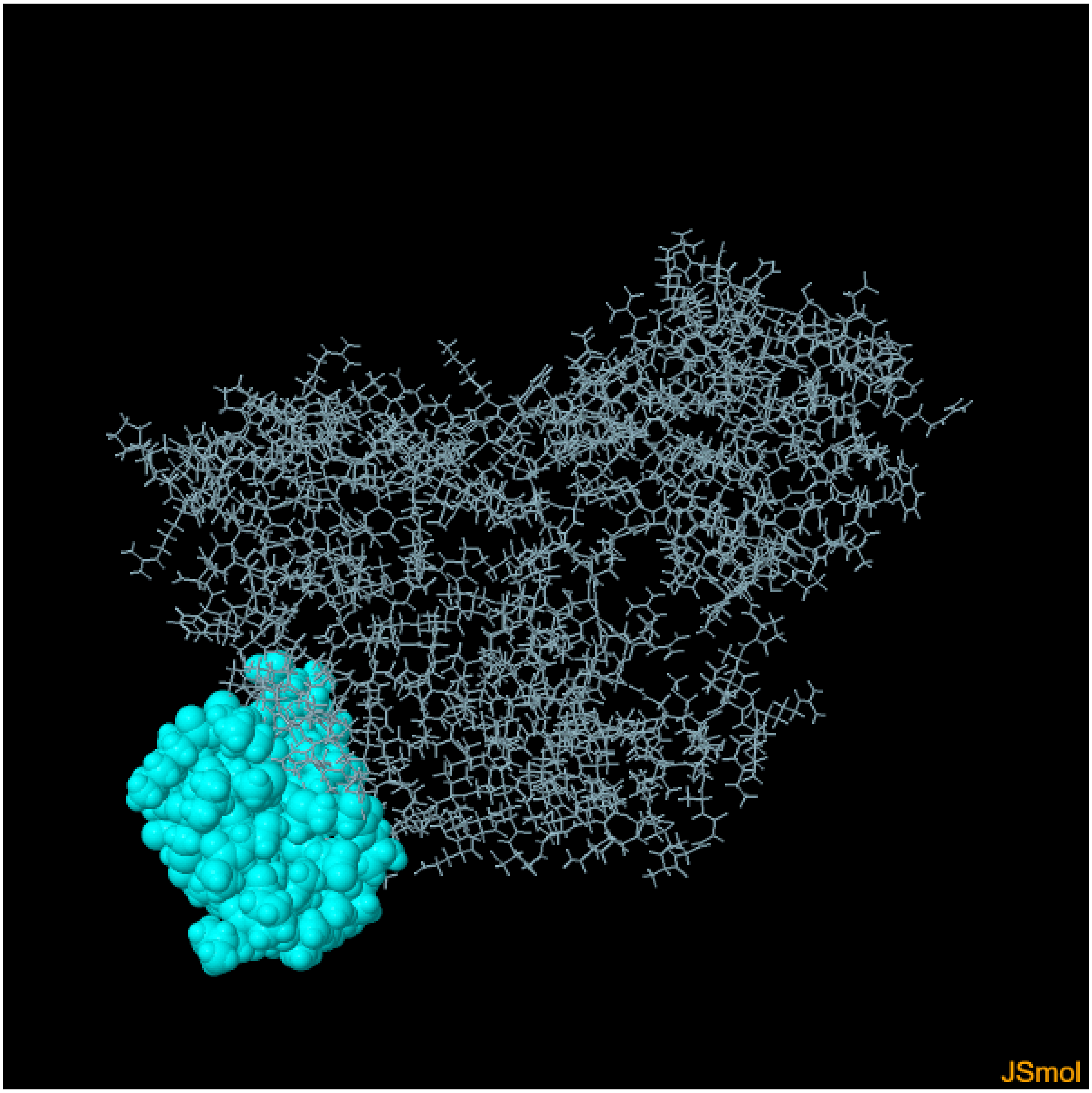

© 2005-2024 IEDB Home

ElliPro: Epitope 3D Structures for fileiqtdbq6t.pdb

No.	Residues	Number of residues	Score
4	A:D237, A:R250, A:A263, A:R266, A:P269, A:T273, A:L276, A:P277, A:A278, A:A279, A:Y280, A:A281, A:V282, A:D283, A:F284, A:I285, A:W286, A:T287, A:G288, A:N289, A:Q290, A:R291, A:T292, A:A293, A:P294, A:R295, A:A296, A:A297, A:R299, A:A300, A:G301, A:R302, A:F303, A:H304, A:W305, A:E306, A:R307, A:F308, A:A391, A:A392, A:H393, A:H394, A:H395, A:H396	44	0.722

JSmol-Rendered PDB Structure

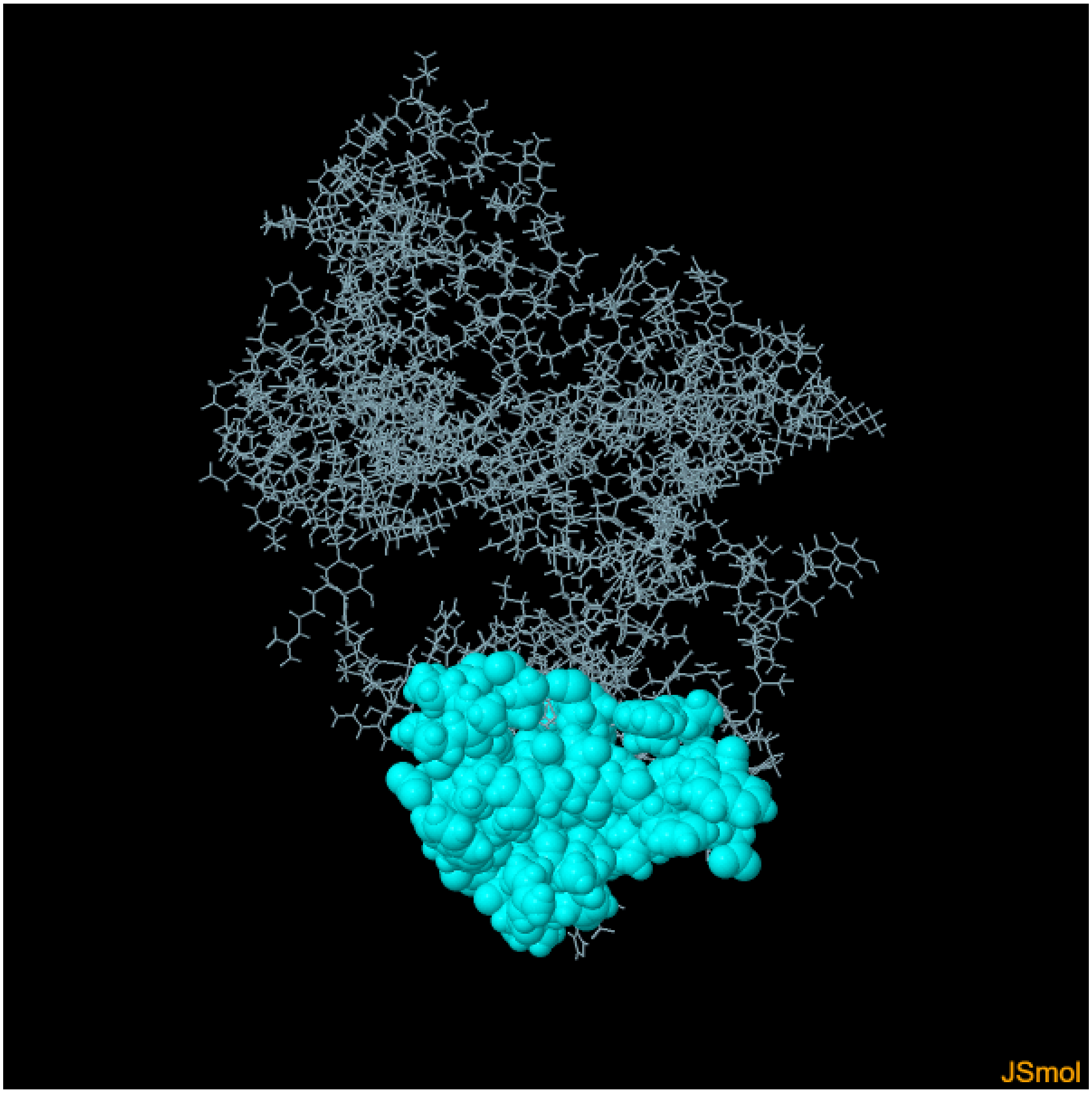

© 2005-2024 IEDB Home

ElliPro: Epitope 3D Structures for fileiqtdbq6t.pdb

No.	Residues	Number of residues	Score
5	A:W342, A:Q343, A:D346, A:E347, A:R350	5	0.702

JSmol-Rendered PDB Structure

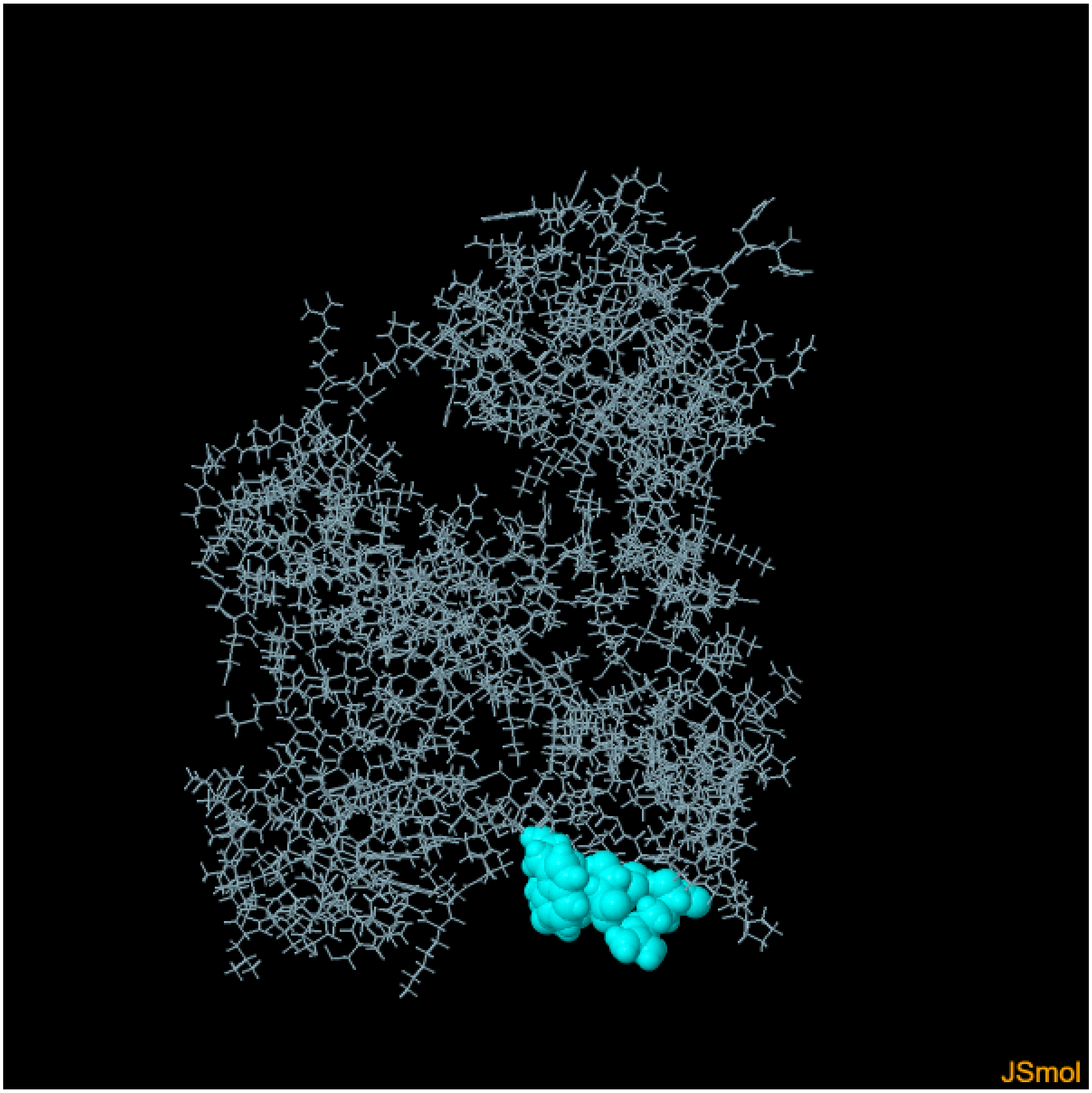

© 2005-2024 IEDB Home

ElliPro: Epitope 3D Structures for fileiqtdbq6t.pdb

No.	Residues	Number of residues	Score
6	A:G91, A:K94, A:V95, A:E98, A:I99, A:V100, A:S101, A:G102, A:L103, A:G104, A:L105, A:K106, A:E107, A:A108, A:D110, A:L111, A:D113, A:E130, A:A131, A:K132, A:A133, A:K134, A:L135, A:E136, A:A137, A:A138, A:G139, A:A140, A:T141, A:V142, A:T143, A:I217, A:T218, A:L220, A:K221, A:K222	36	0.685

JSmol-Rendered PDB Structure

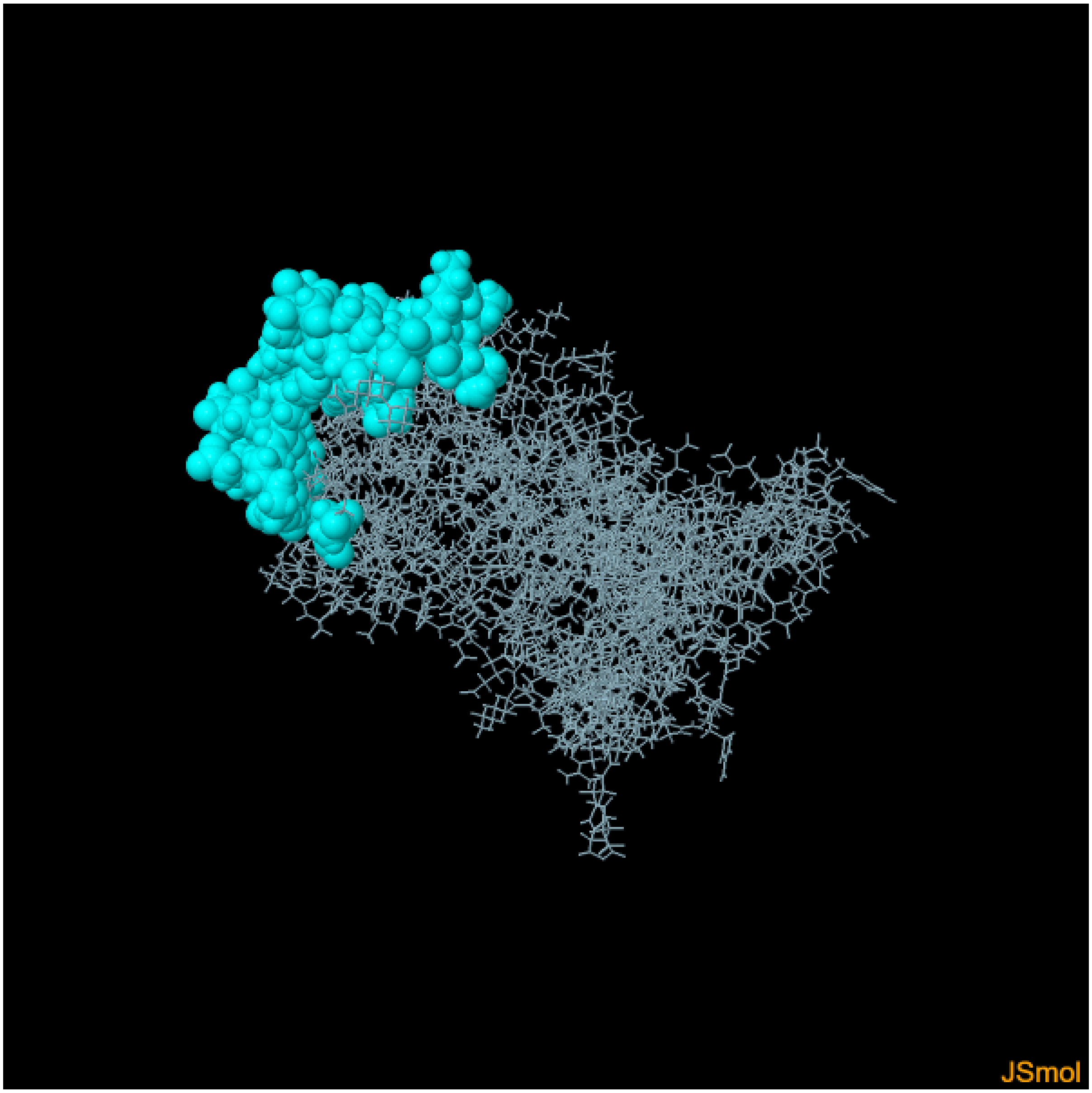

© 2005-2024 IEDB Home

ElliPro: Epitope 3D Structures for fileiqtdbq6t.pdb

No.	Residues	Number of residues	Score
7	A:R223, A:T224, A:A225, A:P226, A:S228, A:L229, A:S230, A:K232	8	0.579

JSmol-Rendered PDB Structure

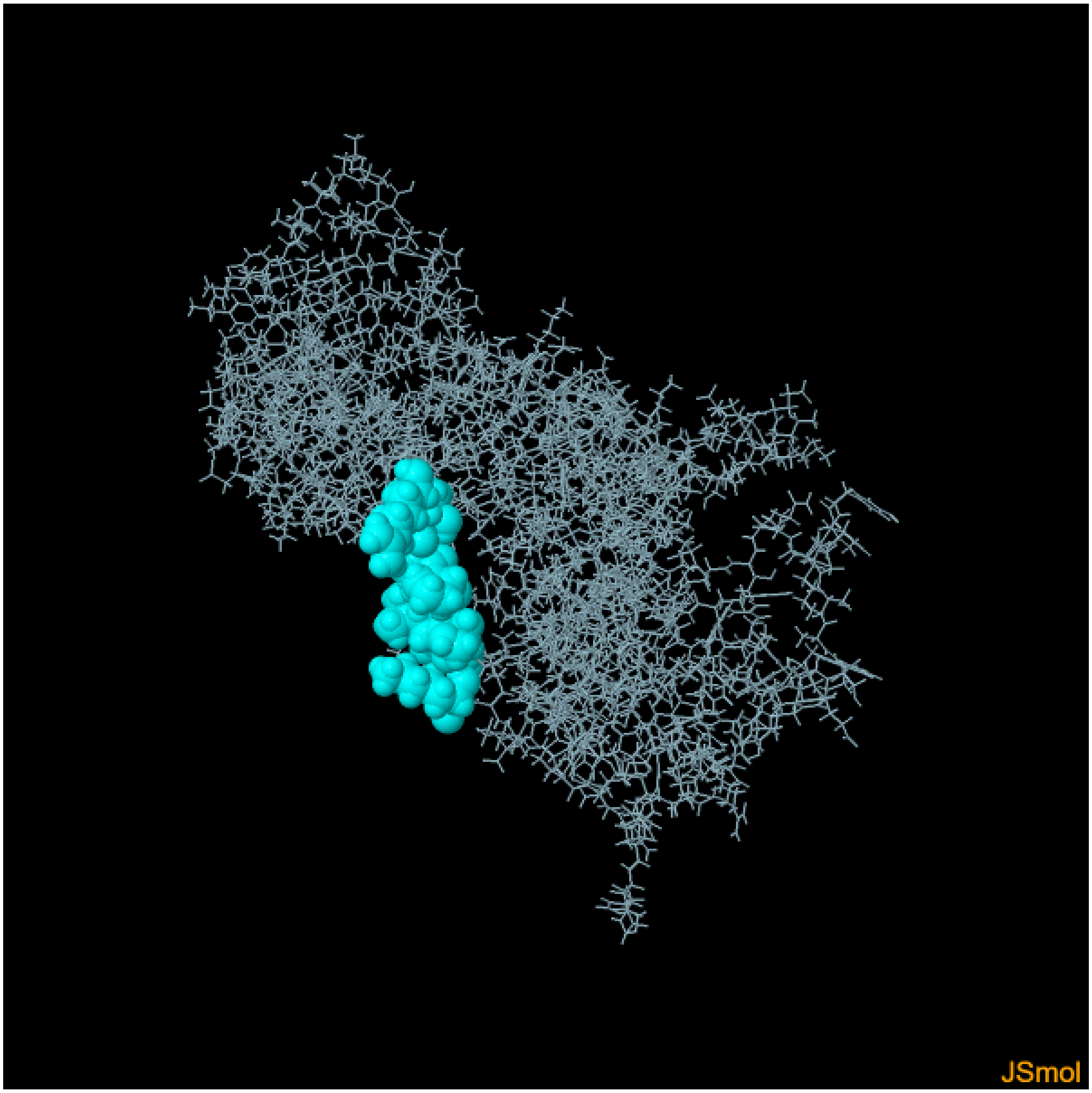

© 2005-2024 IEDB Home
