# Supplementary material for: Reverse vaccinology-based design of multivalent multiepitope mRNA vaccines targeting key viral proteins of Herpes Simplex Virus type-2
Source: Front Immunol. 2025 May 20;16:1586271. doi: 10.3389/fimmu.2025.1586271 (PMC12130045; doi:10.3389/fimmu.2025.1586271)
Supplement: Supplementary file 1 [file DataSheet1.zip › Supplementary Data_22-04-2025/Supplementary Data 3 - ProtParam.pdf]

Expasy **ProtParam (https://web.expasy.org/protparam)**

Home (/protparam/) Documentation (/protparam/protparam-doc.html)
Reference (/protparam/protpar-ref.html) Contact (/contact)

ProtParam - Results

User-provided sequence:

```
        10         20         30         40         50         60
MAKLSTDELL DAFKEMTLLE LSDFVKKFEE TFEVTAAAPV AVAAAGAAPA GAAVEAAEEQ

        70         80         90        100        110        120
SEFDVILEAA GDKKIGVIKV VREIVSGLGL KEAKDLVDGA PKPLLEKVAK EAADEAKAKL

       130        140        150        160        170        180
EAAGATVTVK EAAAKGIINT LQKYYCRVRG GRCAVLSCLP KEEQIGKCST RGRKCCRRKK

       190        200        210        220        230        240
EAAAKFIDLN ITMLKKTLGL LLAYRKKRTA PRSLSLKKKE VDLDFGLKKT NMVLRKRNKA

       250        260        270        280        290        300
RYSPAAYAYR RRFPAVITRV LPAAYAVDFI WTGNQRTAPR AAYRAGRFHW ERFSNASPAA

       310        320        330        340        350        360
YNKQSTRPTG ACVYLEPGPG PGTMTKWQEV DEMLRAEYGP GPGGRVVFLP TIRRQLALAE

       370        380
AAAKAKFVAA WTLKAAAHHH HHH
```

[Documentation (/protparam/protparam-doc.html) / Reference (/protparam/protpar-ref.html)]

Number of amino acids: 383

Molecular weight: 41864.73
Theoretical pI: 9.78

Amino acid composition: CSV format

Ala (A)	65	17.0%
Arg (R)	28	7.3%
Asn (N)	7	1.8%
Asp (D)	13	3.4%
Cys (C)	7	1.8%
Gln (Q)	7	1.8%
Glu (E)	28	7.3%
Gly (G)	25	6.5%
His (H)	7	1.8%
Ile (I)	12	3.1%
Leu (L)	34	8.9%
Lys (K)	39	10.2%
Met (M)	6	1.6%
Phe (F)	13	3.4%
Pro (P)	18	4.7%
Ser (S)	12	3.1%
Thr (T)	21	5.5%
Trp (W)	4	1.0%
Tyr (Y)	11	2.9%
Val (V)	26	6.8%
Pyl (O)	0	0.0%
Sec (U)	0	0.0%
(B)	0	0.0%
(Z)	0	0.0%
(X)	0	0.0%

Total number of negatively charged residues (Asp + Glu): 41
Total number of positively charged residues (Arg + Lys): 67

Atomic composition:

Carbon	C	1871
Hydrogen	H	3032
Nitrogen	N	538
Oxygen	O	524
Sulfur	S	13

Formula: $C_{1871}H_{3032}N_{538}O_{524}S_{13}$
Total number of atoms: 5978

Extinction coefficients:

Extinction coefficients are in units of $M^{-1} cm^{-1}$, at 280 nm measured in water.

Ext. coefficient 38765
Abs 0.1% (=1 g/l) 0.926, assuming all pairs of Cys residues form cystines

```
Ext. coefficient    38390
Abs 0.1% (=1 g/l)   0.917, assuming all Cys residues are reduced
```

Estimated half-life:

```
The N-terminal of the sequence considered is M (Met).
The estimated half-life is: 30 hours (mammalian reticulocytes, in vitro).
                            >20 hours (yeast, in vivo).
                            >10 hours (Escherichia coli, in vivo).
```

Instability index:

The instability index (II) is computed to be 34.75
This classifies the protein as stable.

Aliphatic index: 83.50
Grand average of hydropathicity (GRAVY):-0.259

Expasy (https://www.expasy.org) is operated by the SIB Swiss Institute of Bioinformatics (https://sib.swiss)
Terms of Use (https://www.expasy.org/terms-of-use) | Privacy policy (https://www.sib.swiss/privacy-policy)
