# Supplementary material for: Reverse vaccinology-based design of multivalent multiepitope mRNA vaccines targeting key viral proteins of Herpes Simplex Virus type-2
Source: Front Immunol. 2025 May 20;16:1586271. doi: 10.3389/fimmu.2025.1586271 (PMC12130045; doi:10.3389/fimmu.2025.1586271)
Supplement: Supplementary file 1 [file DataSheet1.zip › Supplementary Data_22-04-2025/Supplementary Data 4B.pdf]

NPS@: Network Protein Sequence @nalysis

This site is a fork of the original PRABI NPS@ server

[HOME] [DESCRIPTION] [HELP] [NEWS] [CONTACT] [Geno3D]

July 30, 2024: **NPS@** updated (see NEWS).

In your publication cite :
NPS@: Network Protein Sequence Analysis
TIBS 2000 March Vol. 25, No 3 [291]:147-150
Combet C., Blanchet C., Geourjon C. and Deléage G.

Job **SOPMA** (ID: bf1091808974) submitted to **NPS@** server on 20241204-065641.

Queued! Running..! Computed in 34 s !

SOPMA result for : C735xx0

Abstract Geourjon, C. & Deléage, G., SOPMA: Significant improvement in protein secondary structure prediction by consensus prediction from multiple alignments. Cabios, 1995, 11, 681-684.

```
         10        20        30        40        50        60        70
          |         |         |         |         |         |         |
FVFLVLLPLVSSQCVMAKLSTDELLDAFKEMTLLELSDFVKKFEETFEVTAAAPVAVAAAGAAPAGAAVE
chhhcccccccccccchhhhhhhhhhhhhhhhhhhhhhhhhhhhhhhhccchhhhhhhhccccccccchhh
AAEEQSEFDVILEAAGDKKIGVIKVVREIVSGLGLKEAKDLVDGAPKPLLEKVAKEAADEAKAKLEAAGA
hhhccchheeeeecccccchhhhhhhhhhhhhcchhhhhhhhhccchhhhhhhhhhhhhhhhhhhhhhcc
TVTVKEAAAKGIINTLQKYYCRVRGGRCAVLSCLPKEEQIGKCSTRGRKCCRRKKEAAAKFIDLNITMLK
heeeehhhhhcchhhhhhhhhhhhccchhhhhhhhhhhhhhhhccccccccccchhhhhhhhhhhhhhhh
KTLGLLLAYRKKRTAPRSLSLKKKEVDLDFGLKKTNMVLRKRNKARYSPAAYAVDFIWTGNQRTAPRAAY
hhhhhhhhhcccccccchhhhccchhhhhhcccccceeeeeccccccccccceeeeeeeccccccccchhh
RAGRFHWERFSNASPAAYAYRRRFPAVITRVLPAAYNKQSTRPTGACVYLEPGPGPGTMTKWQEVDEMLR
ccccceehhhhccccchhhhhcccccchhhhhhcchhcccccccccceeeeecccccccceehhhhhhhhhh
AEYGPGPGGRVVFLPTIRRQLALAEAAAKAKFVAAWTLKAAAHHHHHH
hcccccccceeeeecchhhhhhhhhhhhhhhhhhhhhhhhhhhhccccc
```

```
Sequence length :   398

SOPMA :
   Alpha helix     (Hh) :   216 is  54.27%
   3_10 helix      (Gg) :     0 is   0.00%
   Pi helix        (Ii) :     0 is   0.00%
   Beta bridge     (Bb) :     0 is   0.00%
   Extended strand (Ee) :    39 is   9.80%
   Beta turn       (Tt) :     0 is   0.00%
   Bend region     (Ss) :     0 is   0.00%
   Random coil     (Cc) :   143 is  35.93%
   Ambiguous states (?)  :     0 is   0.00%
   Other states         :     0 is   0.00%
```

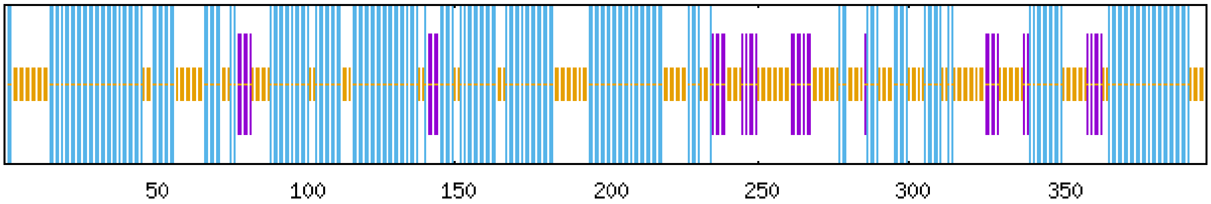

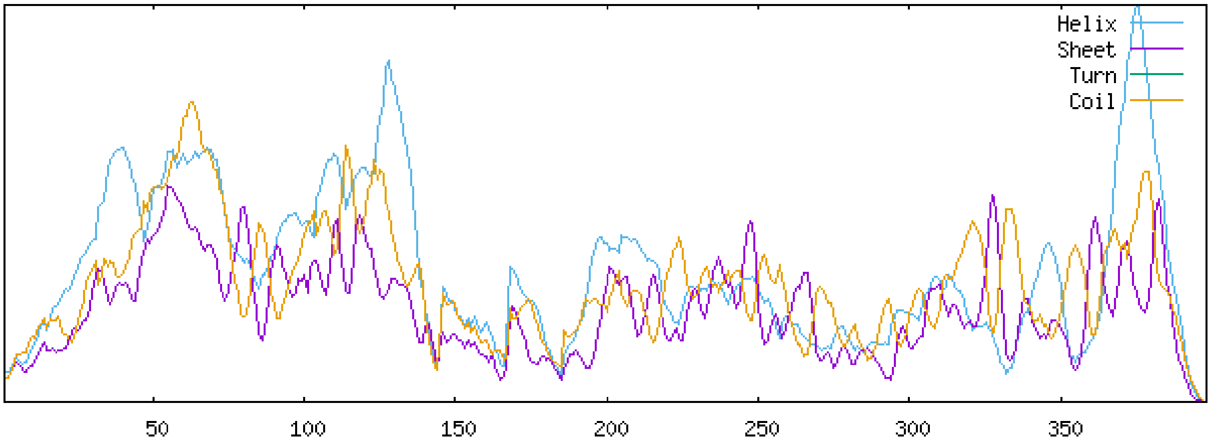


```
Parameters :
   Window width          :  17
   Similarity threshold :   8
   Number of states      :   3
```

Prediction result file (text): [SOPMA]
Intermediate result files (text): [PSI-BLAST on UniProtKB 50% identity] [KALIGN MSA in CLUSTAL W format]

Last modification time : *Wed Dec 4 06:57:18 2024.* ***Current time :*** *Wed Dec 4 06:57:18 2024.* **User :** public@14.139.125.18.

© 1998-2024 Legal notice

NPS@: Network Protein Sequence @nalysis

This site is a fork of the original PRABI NPS@ server

[HOME] [DESCRIPTION] [HELP] [NEWS] [CONTACT] [Geno3D]

July 30, 2024: **NPS@** updated (see NEWS).

In your publication cite :
NPS@: Network Protein Sequence Analysis
TIBS 2000 March Vol. 25, No 3 [291]:147-150
Combet C., Blanchet C., Geourjon C. and Deléage G.

Job **SOPMA** (ID: 3068262d1887) submitted to **NPS@** server on 20241204-065150.

Queued! Running.! Computed in 22 s !

SOPMA result for : C753xx0

Abstract Geourjon, C. & Deléage, G., SOPMA: Significant improvement in protein secondary structure prediction by consensus prediction from multiple alignments. Cabios, 1995, 11, 681-684.

```
         10        20        30        40        50        60        70
          |         |         |         |         |         |         |
FVFLVLLPLVSSQCVMAKLSTDELLDAFKEMTLLELSDFVKKFEETFEVTAAAPVAVAAAGAAPAGAAVE
cccccccccccccchhhhhhhhhhhhhhhhhhhhhhhhhhhhhhhhcccchhcchhhhccccccccchhh
AAEEQSEFDVILEAAGDKKIGVIKVVREIVSGLGLKEAKDLVDGAPKPLLEKVAKEAADEAKAKLEAAGA
hhhhhhhheeeeecccccchhhhhhhhhhhhhcchhhhhhhhhccchhhhhhhhhhhhhhhhhhhhhhcc
TVTVKEAAAKGIINTLQKYYCRVRGGRCAVLSCLPKEEQIGKCSTRGRKCCRRKKEAAAKFIDLNITMLK
heeeechhhhhcccccchhhhhhhhhhhhhhhhcchhhhhcccccccccccccchhhhhhhhhhhhhhhh
KTLGLLLAYRKKRTAPRSLSLKKKEVDLDFGLKKTNMVLRKRNKARYSPAAYAYRRRFPAVITRVLPAAY
hhhhhhhhhcccccccchhhhcccheeeeeccccceeeeecccccccccchhhhhhhccchhhhhhhccccc
AVDFIWTGNQRTAPRAAYRAGRFHWERFSNASPAAYNKQSTRPTGACVYLEPGPGPGTMTKWQEVDEMLR
eeeeeeecccccccchhhccccchhhhhcccccchhcccccccccceeeeecccccccceehhhhhhhhhh
AEYGPGPGGRVVFLPTIRRQLALAEAAAKAKFVAAWTLKAAAHHHHHH
hcccccccceeeeecchhhhhhhhhhhhhhhhhhhhhhhhhhhhccccc
```

```
Sequence length :   398

SOPMA :
   Alpha helix      (Hh) :   210 is  52.76%
   3_10  helix      (Gg) :     0 is   0.00%
   Pi helix         (Ii) :     0 is   0.00%
   Beta bridge      (Bb) :     0 is   0.00%
   Extended strand  (Ee) :    39 is   9.80%
   Beta turn        (Tt) :     0 is   0.00%
   Bend region      (Ss) :     0 is   0.00%
   Random coil      (Cc) :   149 is  37.44%
   Ambiguous states (?)  :     0 is   0.00%
   Other states          :     0 is   0.00%
```

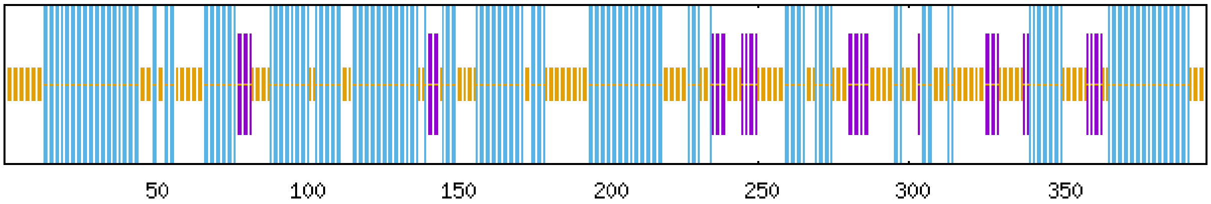

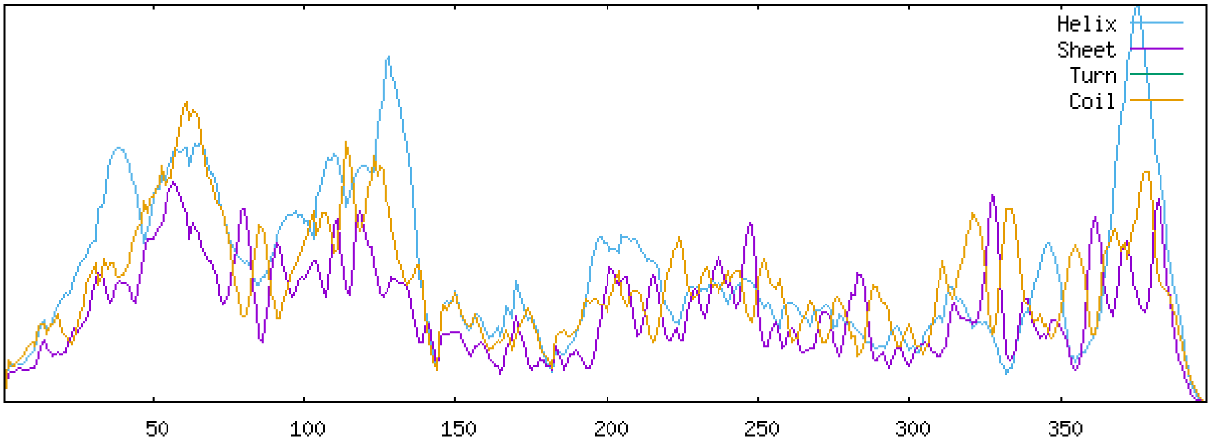


```
Parameters :
   Window width          :  17
   Similarity threshold  :   8
   Number of states      :   3
```

Prediction result file (text): [SOPMA]
Intermediate result files (text): [PSI-BLAST on UniProtKB 50% identity] [KALIGN MSA in CLUSTAL W format]

Last modification time : *Wed Dec 4 06:52:13 2024*. ***Current time :*** *Wed Dec 4 06:52:13 2024*. **User :** public@14.139.125.18.

© 1998-2024 Legal notice

NPS@: Network Protein Sequence @nalysis

This site is a fork of the original PRABI NPS@ server

[HOME] [DESCRIPTION] [HELP] [NEWS] [CONTACT] [Geno3D]

July 30, 2024: **NPS@** updated (see NEWS).

In your publication cite :
NPS@: Network Protein Sequence Analysis
TIBS 2000 March Vol. 25, No 3 [291]:147-150
Combet C., Blanchet C., Geourjon C. and Deléage G.

Job **SOPMA** (ID: 6c4e028fe289) submitted to **NPS@** server on 20241204-070545.

Queued! Running.! Computed in 22 s !

SOPMA result for : C2607x0

Abstract Geourjon, C. & Deléage, G., SOPMA: Significant improvement in protein secondary structure prediction by consensus prediction from multiple alignments. Cabios, 1995, 11, 681-684.

```
         10        20        30        40        50        60        70
          |         |         |         |         |         |         |
FVFLVLLPLVSSQCVMAKLSTDELLDAFKEMTLLELSDFVKKFEETFEVTAAAPVAVAAAGAAPAGAAVE
chhhcccccccccccchhhhhhhhhhhhhhhhhhhhhhhhhhhhhhhccchhhhhhhhccccccccchhh
AAEEQSEFDVILEAAGDKKIGVIKVVREIVSGLGLKEAKDLVDGAPKPLLEKVAKEAADEAKAKLEAAGA
hhhccchheeeeecccccchhhhhhhhhhhhhcchhhhhhhhhccchhhhhhhhhhhhhhhhhhhhhhcc
TVTVKEAAAKGIINTLQKYYCRVRGGRCAVLSCLPKEEQIGKCSTRGRKCCRRKKEAAAKTLGLLLAYRK
heeeehhhhhcchhhhhhhhhhhhccchhhhhhhhhhhhhhhhcccccccccchhhhhhhhhhhhhhhcc
KFIDLNITMLKKKEVDLDFGLKKRTAPRSLSLKKTNMVLRKRNKARYSPAAYAVDFIWTGNQRTAPRAAY
cheeeeeeehcccceeeeecccccccccchhhhhhheeeeccccccccccceeeeeeecccccccccchhh
RAGRFHWERFSNASPAAYAYRRRFPAVITRVLPAAYNKQSTRPTGACVYLEPGPGPGTMTKWQEVDEMLR
ccccceThhhhccccchhhhhccccchhhhhhcchhcccccccccceeeeecccccccceehhhhhhhhhh
AEYGPGPGGRVVFLPTIRRQLALAEAAAKAKFVAAWTLKAAAHHHHHH
hcccccccceeeeecchhhhhhhhhhhhhhhhhhhhhhhhhhhhccccc
```

```
Sequence length :   398

SOPMA :
   Alpha helix     (Hh) :   210 is  52.76%
   3_10 helix       (Gg) :     0 is   0.00%
   Pi helix        (Ii) :     0 is   0.00%
   Beta bridge     (Bb) :     0 is   0.00%
   Extended strand (Ee) :    45 is  11.31%
   Beta turn       (Tt) :     0 is   0.00%
   Bend region     (Ss) :     0 is   0.00%
   Random coil     (Cc) :   143 is  35.93%
   Ambiguous states (?)  :     0 is   0.00%
   Other states         :     0 is   0.00%
```

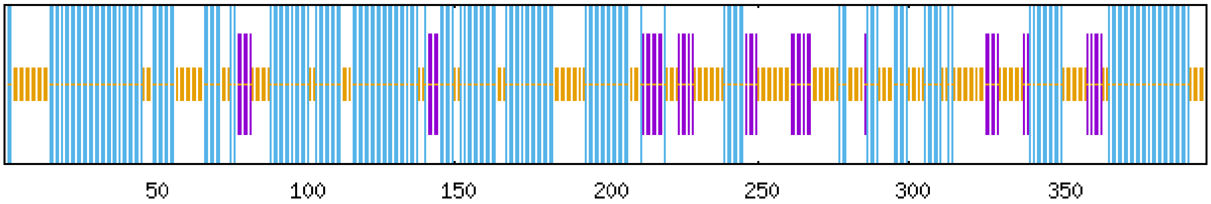

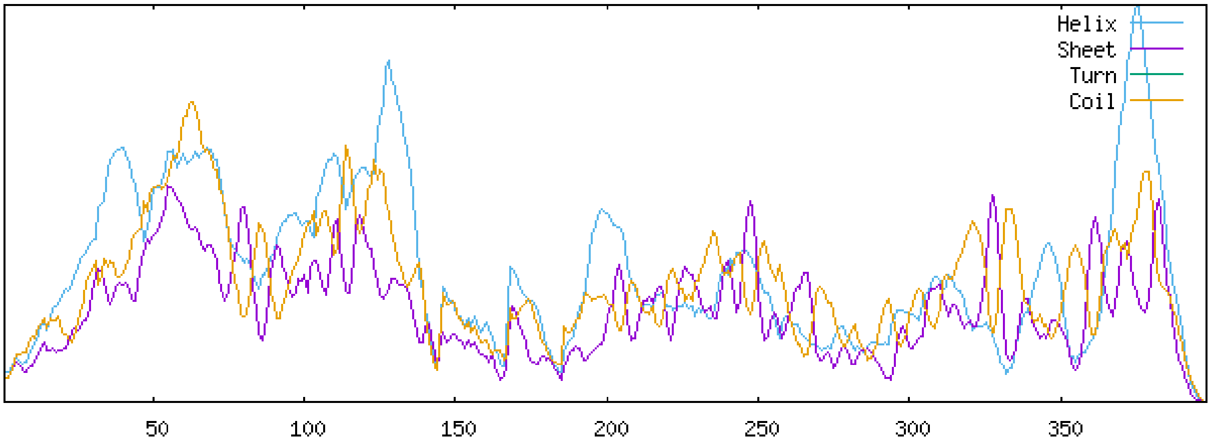


```
Parameters :
   Window width          :  17
   Similarity threshold  :   8
   Number of states      :   3
```

Prediction result file (text): [SOPMA]
Intermediate result files (text): [PSI-BLAST on UniProtKB 50% identity] [KALIGN MSA in CLUSTAL W format]

Last modification time : *Wed Dec 4 07:06:08 2024.* ***Current time :*** *Wed Dec 4 07:06:08 2024.* **User :** public@14.139.125.18.

© 1998-2024 Legal notice

NPS@: Network Protein Sequence @nalysis

This site is a fork of the original PRABI NPS@ server

[HOME] [DESCRIPTION] [HELP] [NEWS] [CONTACT] [Geno3D]

July 30, 2024: **NPS@** updated (see NEWS).

In your publication cite :
NPS@: Network Protein Sequence Analysis
TIBS 2000 March Vol. 25, No 3 [291]:147-150
Combet C., Blanchet C., Geourjon C. and Deléage G.

Job **SOPMA** (ID: 20e80bb85db8) submitted to **NPS@** server on 20241204-065447.

Queued! Running..! Computed in 34 s !

SOPMA result for : C2625x0

Abstract Geourjon, C. & Deléage, G., SOPMA: Significant improvement in protein secondary structure prediction by consensus prediction from multiple alignments. Cabios, 1995, 11, 681-684.

```
         10        20        30        40        50        60        70
          |         |         |         |         |         |         |
FVFLVLLPLVSSQCVMAKLSTDELLDAFKEMTLLELSDFVKKFEETFEVTAAAPVAVAAAGAAPAGAAVE
cccccccccccccchhhhhhhhhhhhhhhhhhhhhhhhhhhhhhhhcccchhcchhhhccccccccchhh
AAEEQSEFDVILEAAGDKKIGVIKVVREIVSGLGLKEAKDLVDGAPKPLLEKVAKEAADEAKAKLEAAGA
hhhhhhhheeeeecccccchhhhhhhhhhhhhcchhhhhhhhhccchhhhhhhhhhhhhhhhhhhhhhcc
TVTVKEAAAKGIINTLQKYYCRVRGGRCAVLSCLPKEEQIGKCSTRGRKCCRRKKEAAAKTLGLLLAYRK
heeeechhhhhcccccchhhhhhhhhhhhhhhhcchhhhhhccccccccccccchhhhhhhhhhhhhhhcc
KFIDLNITMLKKKEVDLDFGLKKRTAPRSLSLKKTNMVLRKRNKARYSPAAYAYRRRFPAVITRVLPAAY
cheeeeeeehcccceeeeecccccccccchhhhhhheeeeccccccccchhhhhhhccchhhhhhccccc
AVDFIWTGNQRTAPRAAYRAGRFHWERFSNASPAAYNKQSTRPTGACVYLEPGPGPGTMTKWQEVDEMLR
eeeeeeeccccccccHhhhccccceHhhhccccchhcccccccccceeeeeccccccccceehhhhhhhhhh
AEYGPGPGGRVVFLPTIRRQLALAEAAAKAKFVAAWTLKAAAHHHHHH
hcccccccceeeeecchhhhhhhhhhhhhhhhhhhhhhhhhhhhccccc

Sequence length :   398

SOPMA :
   Alpha helix     (Hh) :   204 is  51.26%
   3₁₀ helix       (Gg) :     0 is   0.00%
   Pi helix        (Ii) :     0 is   0.00%
   Beta bridge     (Bb) :     0 is   0.00%
   Extended strand (Ee) :    45 is  11.31%
   Beta turn       (Tt) :     0 is   0.00%
   Bend region     (Ss) :     0 is   0.00%
   Random coil     (Cc) :   149 is  37.44%
   Ambiguous states (?)  :     0 is   0.00%
   Other states          :     0 is   0.00%
```

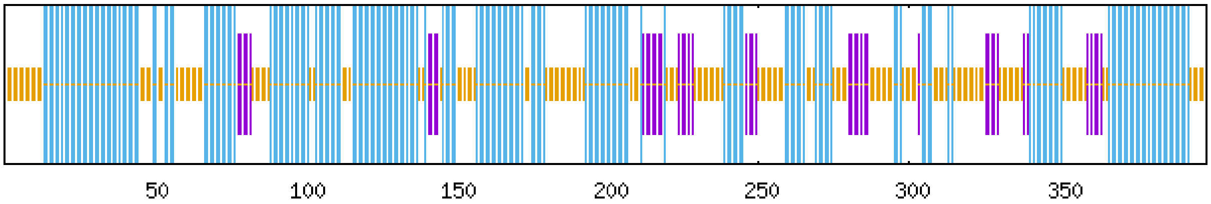

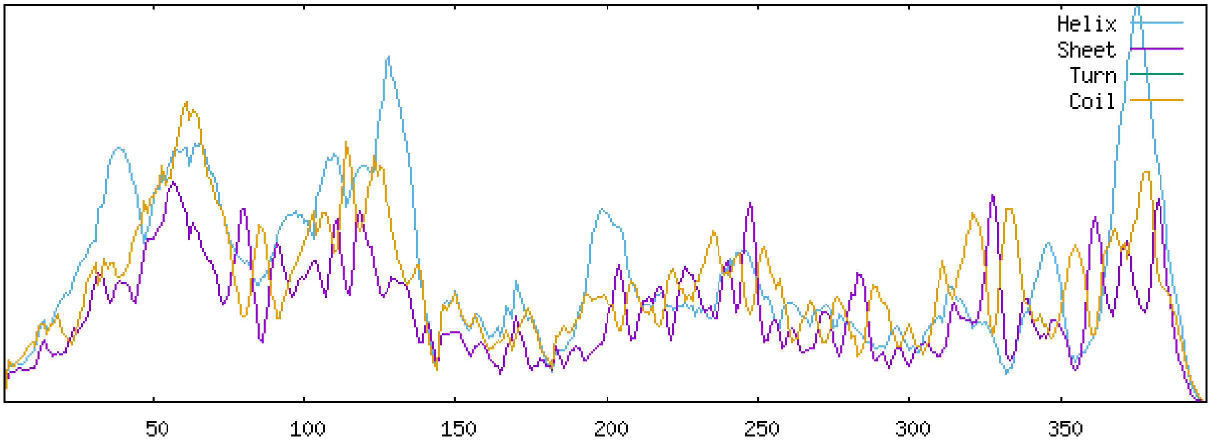


```
Parameters :
   Window width          :  17
   Similarity threshold  :   8
   Number of states      :   3
```

Prediction result file (text): [SOPMA]
Intermediate result files (text): [PSI-BLAST on UniProtKB 50% identity] [KALIGN MSA in CLUSTAL W format]

Last modification time : *Wed Dec 4 06:55:24 2024.* ***Current time :*** *Wed Dec 4 06:55:24 2024.* **User :** public@14.139.125.18.

© 1998-2024 Inserm Legal notice

NPS@: Network Protein Sequence @nalysis

This site is a fork of the original PRABI NPS@ server

[HOME] [DESCRIPTION] [HELP] [NEWS] [CONTACT] [Geno3D]

July 30, 2024: **NPS@** updated (see NEWS).

In your publication cite :
NPS@: Network Protein Sequence Analysis
TIBS 2000 March Vol. 25, No 3 [291]:147-150
Combet C., Blanchet C., Geourjon C. and Deléage G.

Job **SOPMA** (ID: a80a4025ecf0) submitted to **NPS@** server on 20241204-070728.

Queued! Running..! Computed in 32 s !

SOPMA result for : C2769x0

Abstract Geourjon, C. & Deléage, G., SOPMA: Significant improvement in protein secondary structure prediction by consensus prediction from multiple alignments. Cabios, 1995, 11, 681-684.

```
        10        20        30        40        50        60        70
         |         |         |         |         |         |         |
FVFLVLLPLVSSQCVMAKLSTDELLDAFKEMTLLELSDFVKKFEETFEVTAAAPVAVAAAGAAPAGAAVE
cccccccccccccchhhhhhhhhhhhhhhhhhhhhhhhhhhhhhhhcccchhcchhhhccccccccchhh
AAEEQSEFDVILEAAGDKKIGVIKVVREIVSGLGLKEAKDLVDGAPKPLLEKVAKEAADEAKAKLEAAGA
hhhhhhhheeeeecccccchhhhhhhhhhhhhcchhhhhhhhhccchhhhhhhhhhhhhhhhhhhhhhcc
TVTVKEAAAKGIINTLQKYYCRVRGGRCAVLSCLPKEEQIGKCSTRGRKCCRRKKEAAAKTLGLLLAYRK
heeeechhhhhcccccchhhhhhhhhhhhhhhhcchhhhhccccccccccccchhhhhhhhhhhhhhhhc
KFIDLNITMLKKRTAPRSLSLKKKEVDLDFGLKKTNMVLRKRNKARYSPAAYAYRRRFPAVITRVLPAAY
cheeeeeeeecccccccchhhcccheeeeeccccceeeeeccccccccchhhhhhhccchhhhhhhccccc
AVDFIWTGNQRTAPRAAYRAGRFHWERFSNASPAAYNKQSTRPTGACVYLEPGPGPGTMTKWQEVDEMLR
eeeeeeecccccccchhhccccceehhhhccccchhcccccccccceeeeecccccccceehhhhhhhhhh
AEYGPGPGGRVVFLPTIRRQLALAEAAAKAKFVAAWTLKAAAHHHHHH
hcccccccceeeeecchhhhhhhhhhhhhhhhhhhhhhhhhhhccccc

Sequence length :   398

SOPMA :
   Alpha helix     (Hh) :   201 is  50.50%
   3_10 helix       (Gg) :     0 is   0.00%
   Pi helix        (Ii) :     0 is   0.00%
   Beta bridge     (Bb) :     0 is   0.00%
   Extended strand (Ee) :    47 is  11.81%
   Beta turn       (Tt) :     0 is   0.00%
   Bend region     (Ss) :     0 is   0.00%
   Random coil     (Cc) :   150 is  37.69%
   Ambiguous states (?)  :     0 is   0.00%
   Other states          :     0 is   0.00%
```

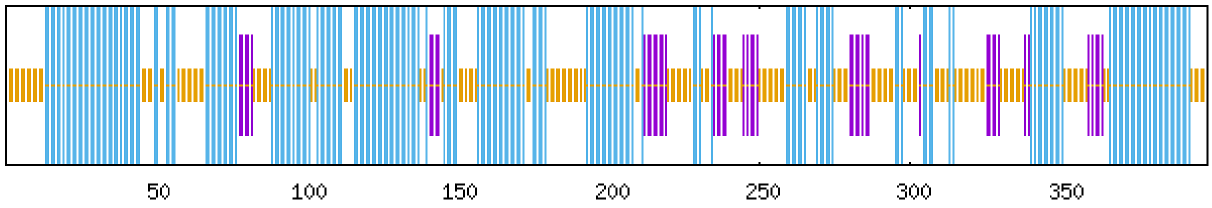

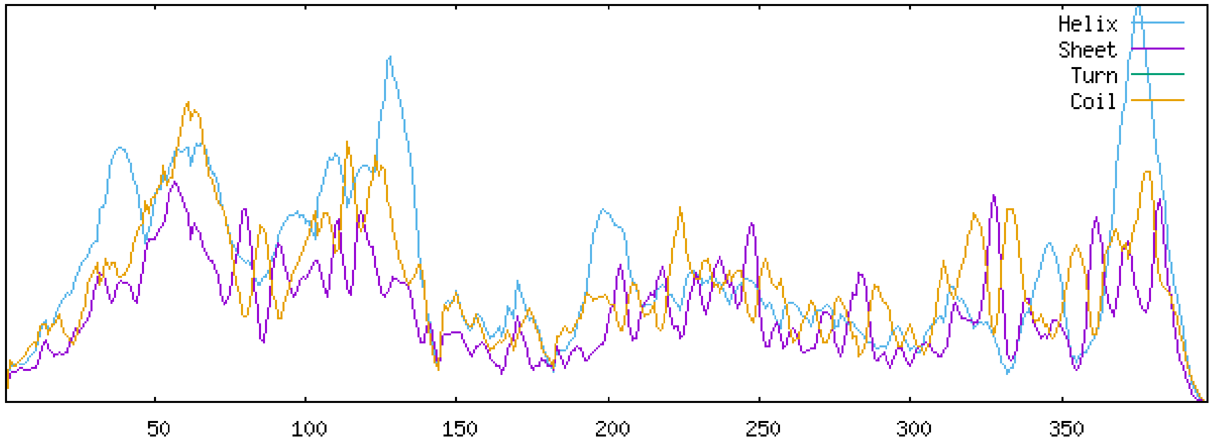


```
Parameters :
   Window width         :  17
   Similarity threshold :   8
   Number of states     :   3
```

Prediction result file (text): [SOPMA]
Intermediate result files (text): [PSI-BLAST on UniProtKB 50% identity] [KALIGN MSA in CLUSTAL W format]

Last modification time : *Wed Dec 4 07:08:00 2024.* ***Current time :*** *Wed Dec 4 07:08:00 2024.* **User :** public@14.139.125.18.

© 1998-2024 Legal notice
