# Supplementary material for: Reverse vaccinology-based design of multivalent multiepitope mRNA vaccines targeting key viral proteins of Herpes Simplex Virus type-2
Source: Front Immunol. 2025 May 20;16:1586271. doi: 10.3389/fimmu.2025.1586271 (PMC12130045; doi:10.3389/fimmu.2025.1586271)
Supplement: Supplementary file 1 [file DataSheet1.zip › Supplementary Data_22-04-2025/Supplementary Data 6.pdf]

ProSA-web
Protein Structure Analysis

Please upload a structure in PDB format: HELP

Choose File No file chosen

Alternatively you can specify a structure by entering its PDB code, chain identifier and NMR model number:

PDB CODE:

PDB CHAIN ID:

PDB MODEL NUMBER:

If you leave the fields for chain id or model number blank,
the first chain of the first model found in the PDB file will be analysed.

Analyse

Results for C735_2.pdb, chain A (398 aa)

Overall model quality

HELP

Z-Score: **-5.75**

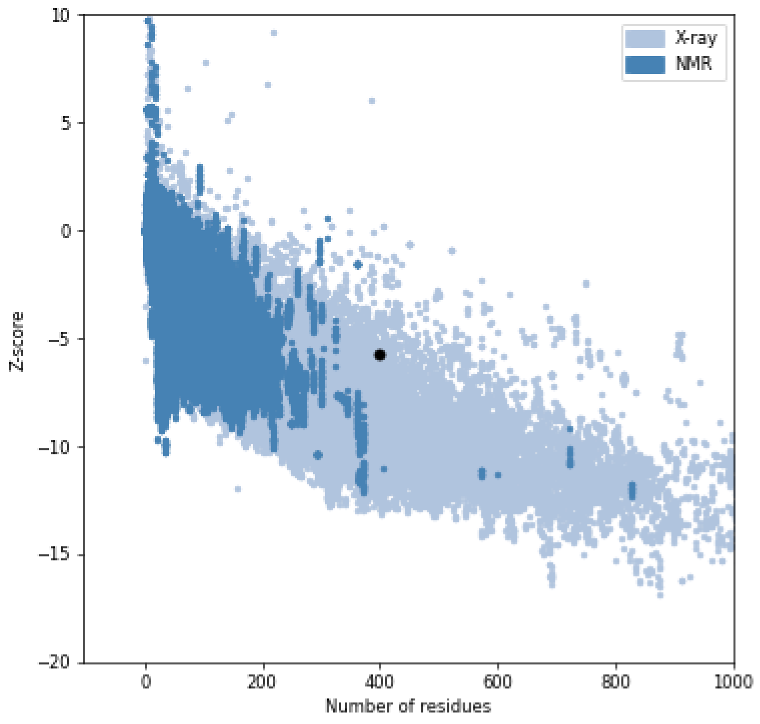

Local model quality

HELP
PNG

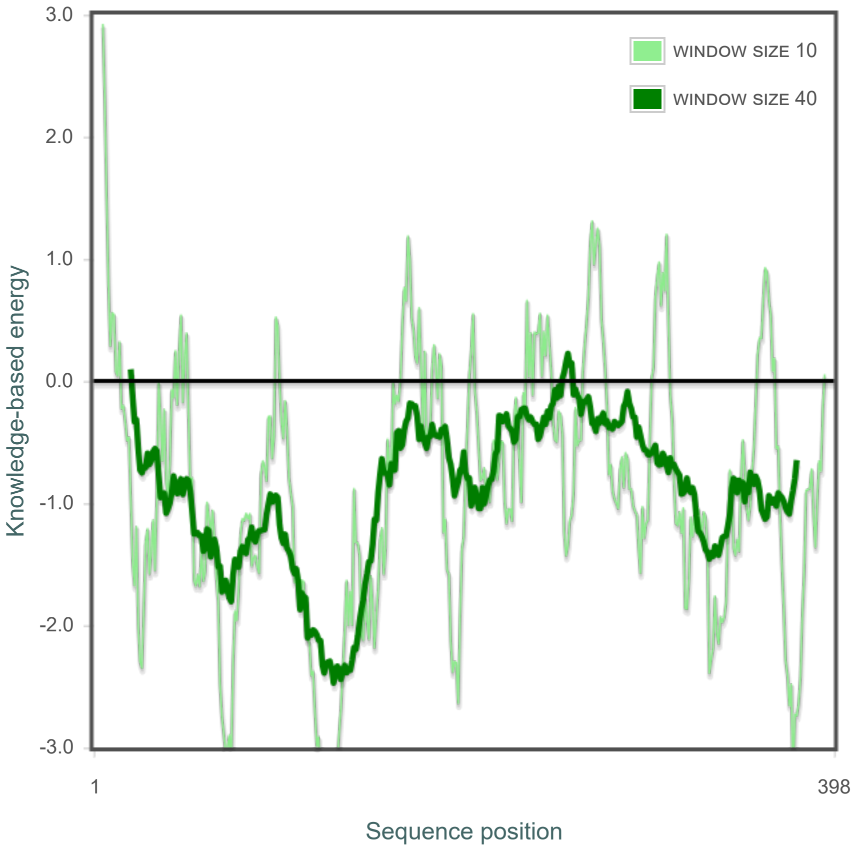

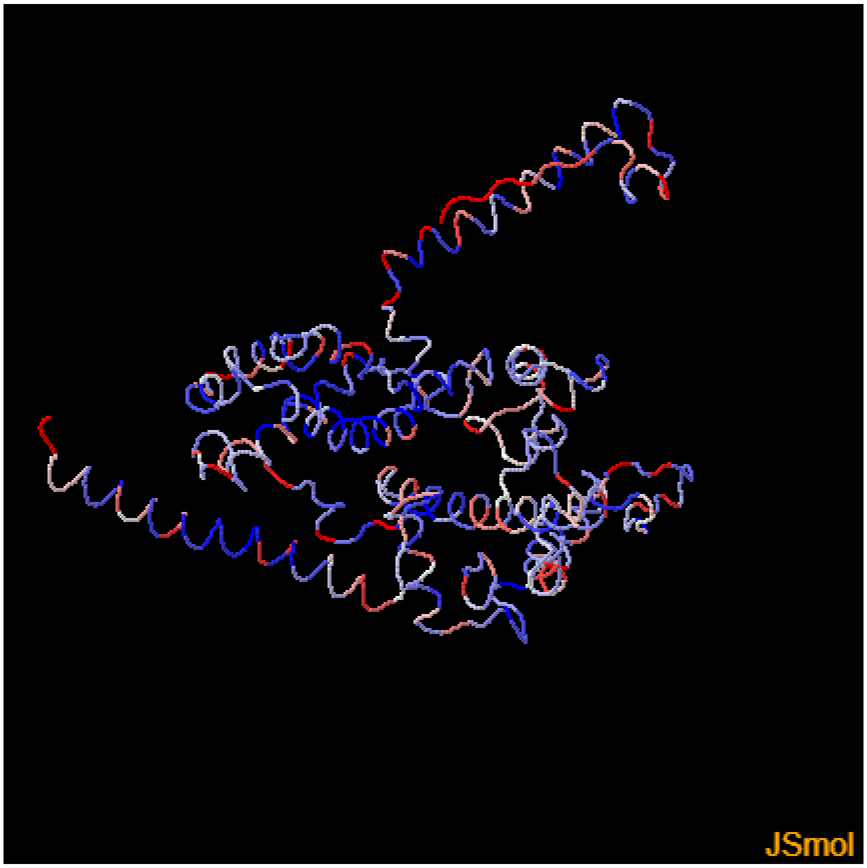

Lowest energy Highest energy

Please cite the following articles if you publish results using ProSA-web:

- Wiederstein & Sippl (2007)
  ProSA-web: interactive web service for the recognition of errors in three-dimensional structures of proteins.
  Nucleic Acids Research 35, W407-W410. [view]
- Sippl, M.J. (1993)
  Recognition of Errors in Three-Dimensional Structures of Proteins.
  Proteins 17, 355-362. [view]

This site is maintained by Markus Wiederstein. For comments and suggestions please contact prosa@came.sbg.ac.at.

ProSA-web
Protein Structure Analysis

Please upload a structure in PDB format: HELP

Choose File No file chosen

Alternatively you can specify a structure by entering its PDB code, chain identifier and NMR model number:

PDB CODE:

PDB CHAIN ID:

PDB MODEL NUMBER:

If you leave the fields for chain id or model number blank, the first chain of the first model found in the PDB file will be analysed.

Analyse

Results for C753_5.pdb, chain A (383 aa)

Overall model quality

HELP

Z-Score: **-5.98**

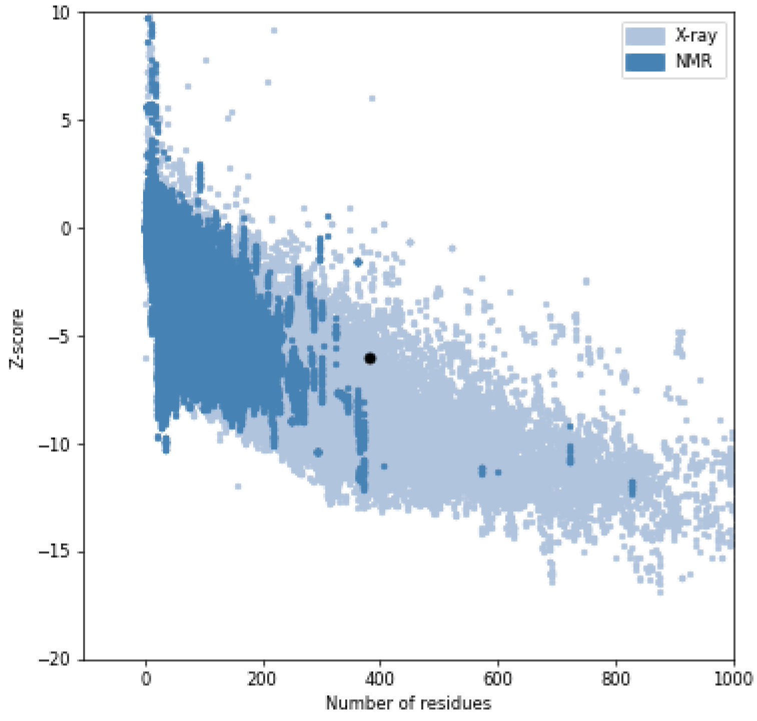

Local model quality

HELP
PNG

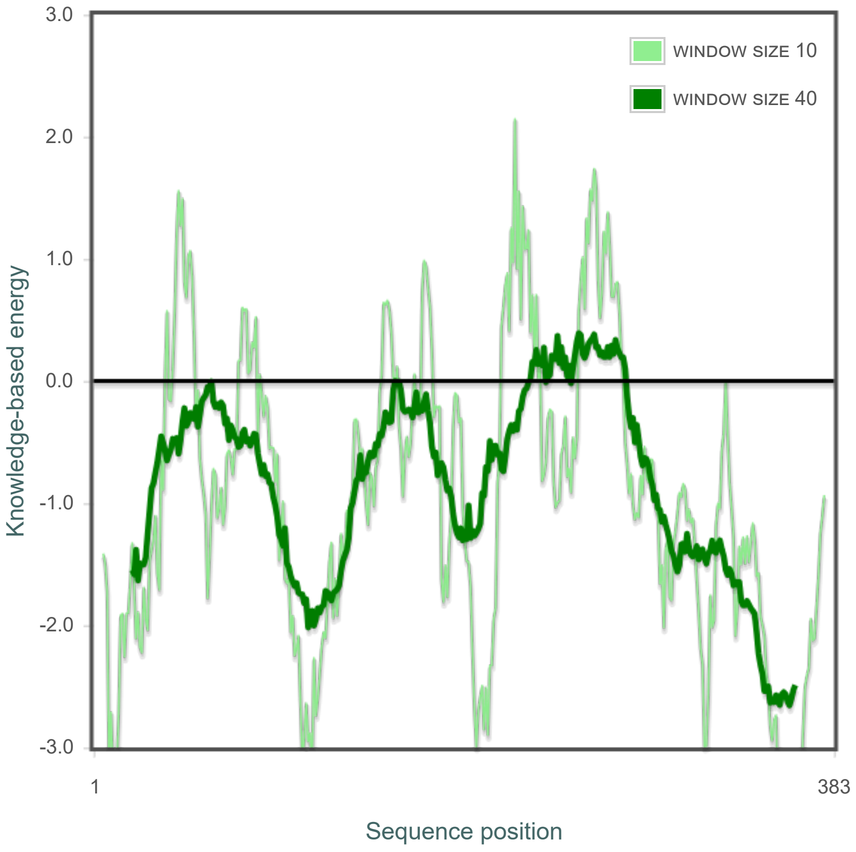

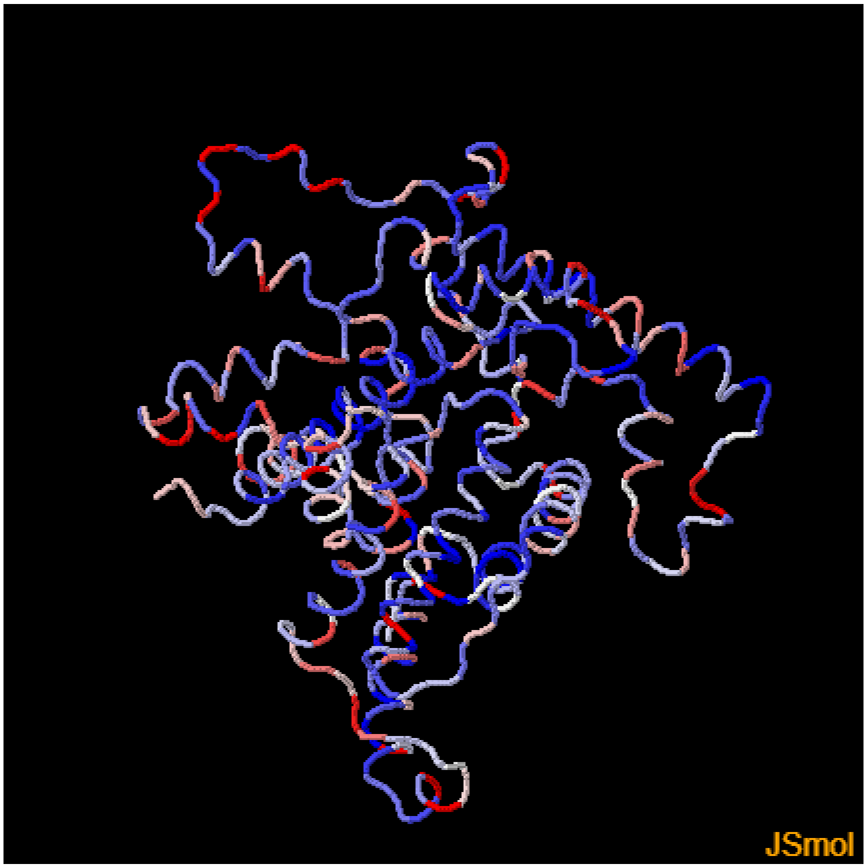

Lowest energy Highest energy

Please cite the following articles if you publish results using ProSA-web:

- Wiederstein & Sippl (2007)
  ProSA-web: interactive web service for the recognition of errors in three-dimensional structures of proteins.
  Nucleic Acids Research 35, W407-W410. [view]
- Sippl, M.J. (1993)
  Recognition of Errors in Three-Dimensional Structures of Proteins.
  Proteins 17, 355-362. [view]

This site is maintained by Markus Wiederstein. For comments and suggestions please contact prosa@came.sbg.ac.at.

ProSA-web
Protein Structure Analysis

Please upload a structure in PDB format: HELP

Choose File No file chosen

Alternatively you can specify a structure by entering its PDB code, chain identifier and NMR model number:

PDB CODE:

PDB CHAIN ID:

PDB MODEL NUMBER:

If you leave the fields for chain id or model number blank, the first chain of the first model found in the PDB file will be analysed.

Analyse

Results for C2607_5.pdb, chain A (398 aa)

Overall model quality HELP

Z-Score: **-5.2**

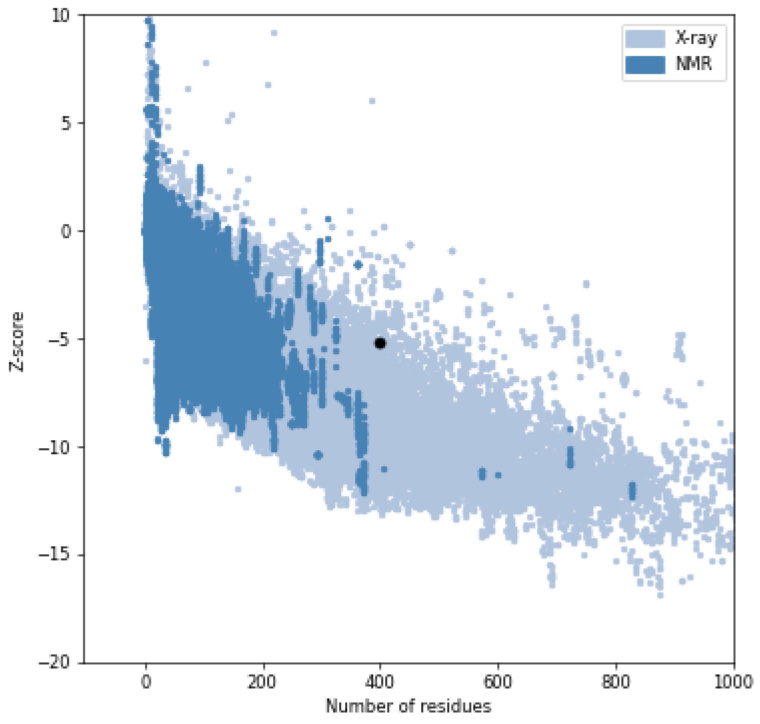

Local model quality

HELP
PNG

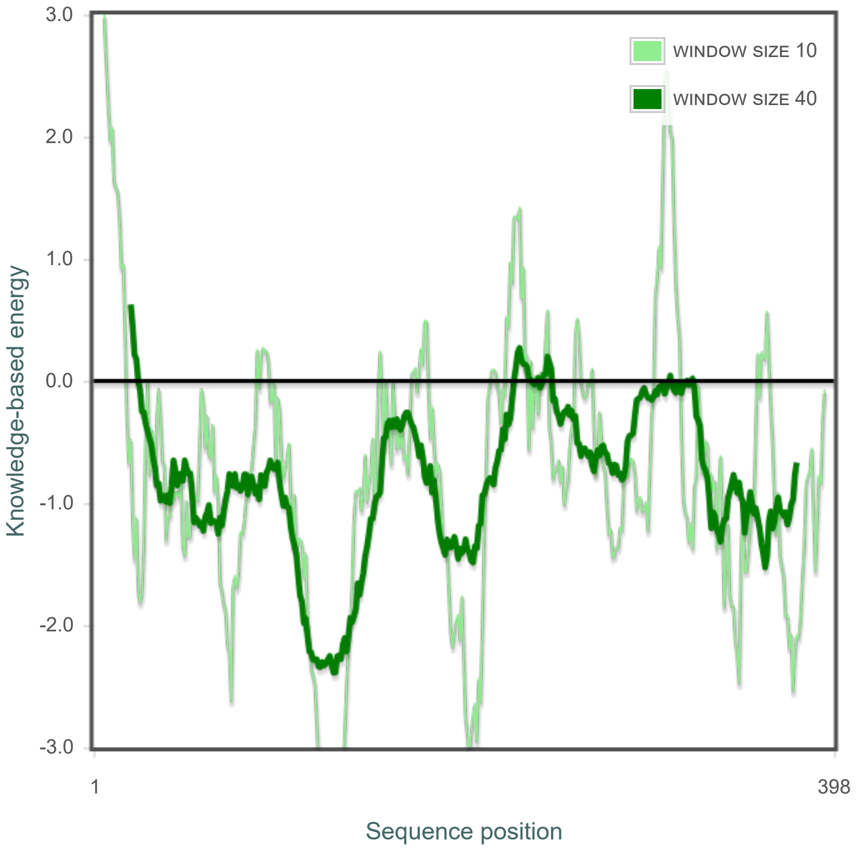

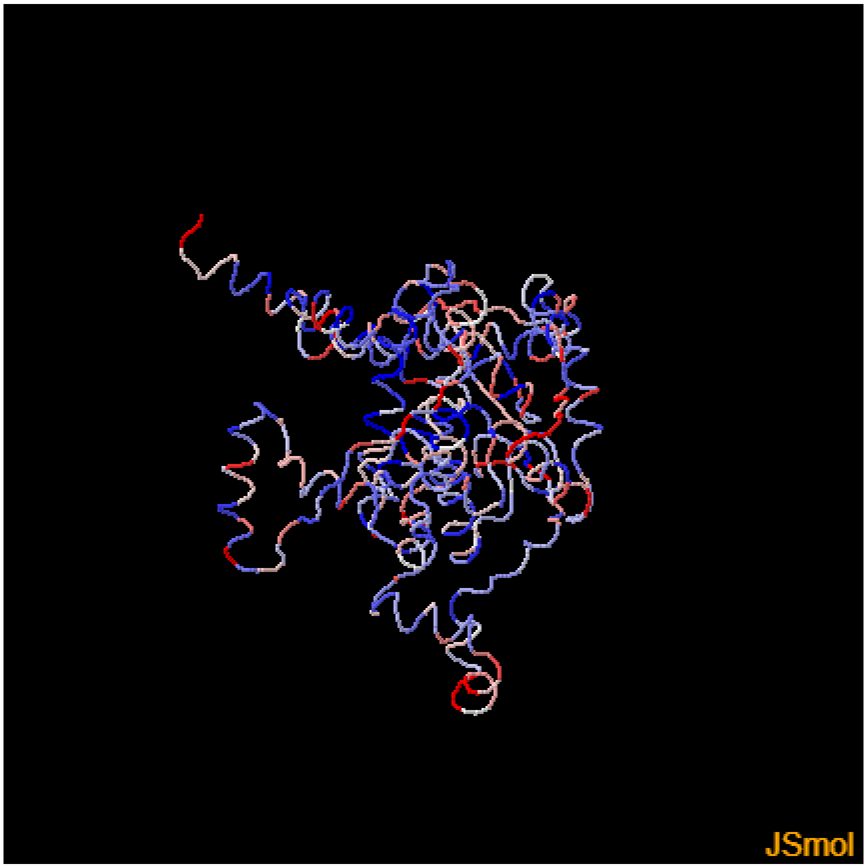

Lowest energy Highest energy

Please cite the following articles if you publish results using ProSA-web:

- Wiederstein & Sippl (2007)
  ProSA-web: interactive web service for the recognition of errors in three-dimensional structures of proteins.
  Nucleic Acids Research 35, W407-W410. [view]
- Sippl, M.J. (1993)
  Recognition of Errors in Three-Dimensional Structures of Proteins.
  Proteins 17, 355-362. [view]

This site is maintained by Markus Wiederstein. For comments and suggestions please contact prosa@came.sbg.ac.at.

ProSA-web
Protein Structure Analysis

Please upload a structure in PDB format: HELP

Choose File No file chosen

Alternatively you can specify a structure by entering its PDB code, chain identifier and NMR model number:

PDB CODE:

PDB CHAIN ID:

PDB MODEL NUMBER:

If you leave the fields for chain id or model number blank, the first chain of the first model found in the PDB file will be analysed.

Analyse

Results for C2625_4.pdb, chain A (398 aa)

Overall model quality HELP

Z-Score: **-7.09**

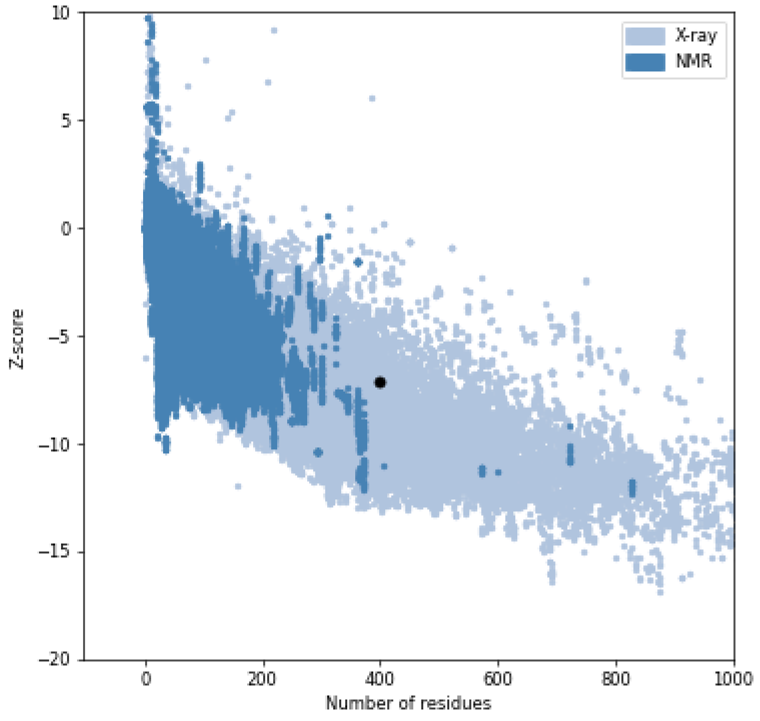

Local model quality

Help
PNG

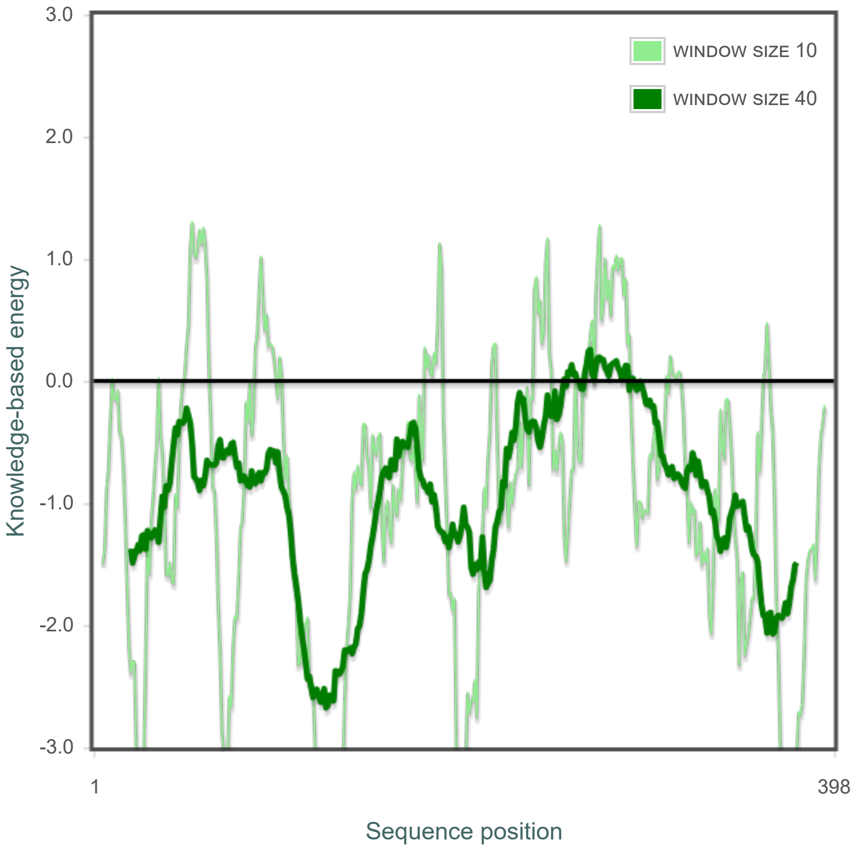

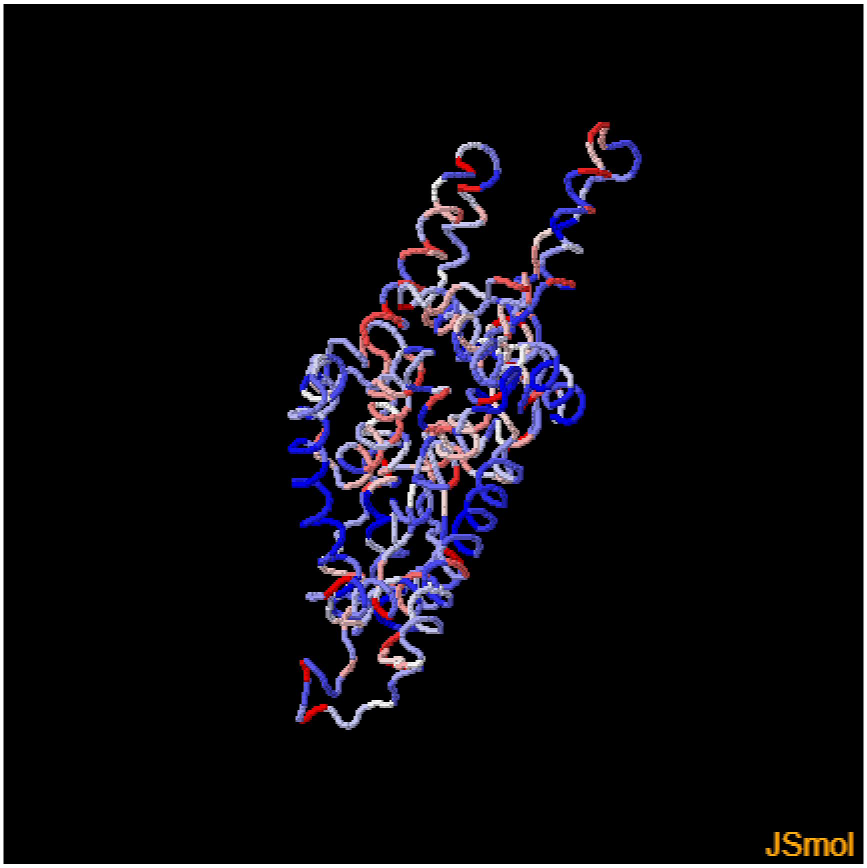

Lowest energy Highest energy

Please cite the following articles if you publish results using ProSA-web:

- Wiederstein & Sippl (2007)
  ProSA-web: interactive web service for the recognition of errors in three-dimensional structures of proteins.
  Nucleic Acids Research 35, W407-W410. [view]
- Sippl, M.J. (1993)
  Recognition of Errors in Three-Dimensional Structures of Proteins.
  Proteins 17, 355-362. [view]

This site is maintained by Markus Wiederstein. For comments and suggestions please contact prosa@came.sbg.ac.at.

ProSA-web
Protein Structure Analysis

Please upload a structure in PDB format: HELP

Choose File No file chosen

Alternatively you can specify a structure by entering its PDB code, chain identifier and NMR model number:

PDB CODE:

PDB CHAIN ID:

PDB MODEL NUMBER:

If you leave the fields for chain id or model number blank, the first chain of the first model found in the PDB file will be analysed.

Analyse

Results for C2769_1.pdb, chain A (398 aa)

Overall model quality

HELP

Z-Score: **-6.45**

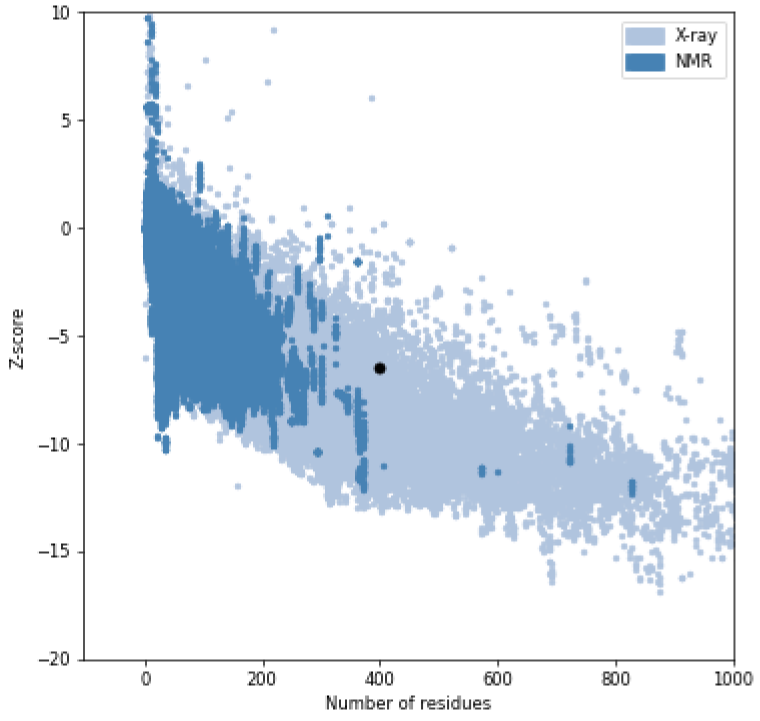

Local model quality

HELP
PNG

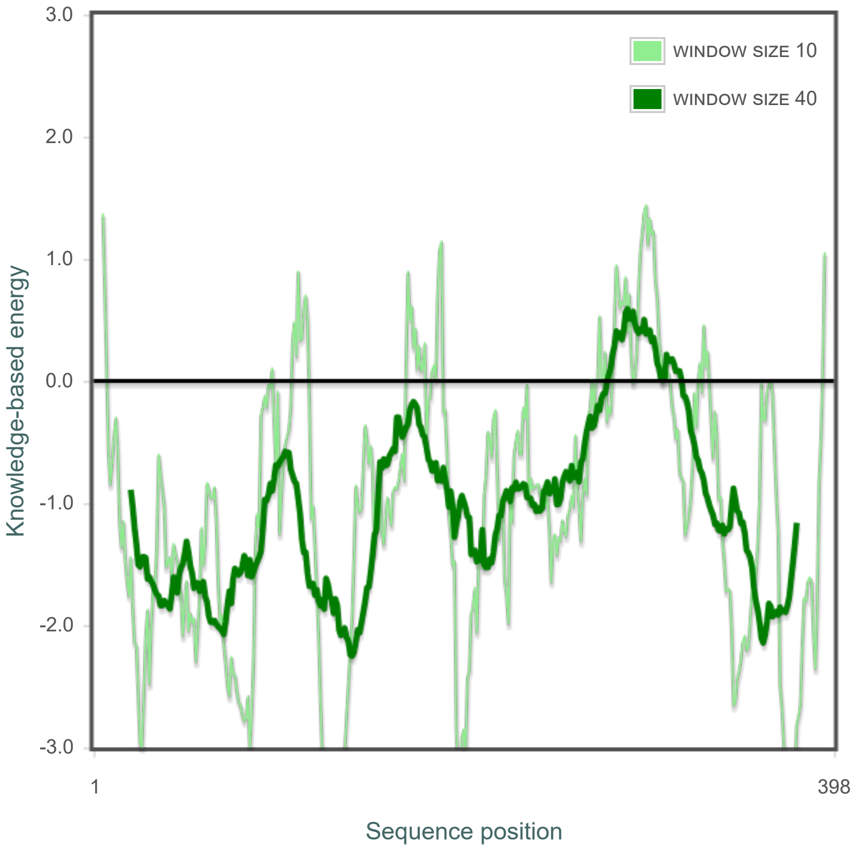

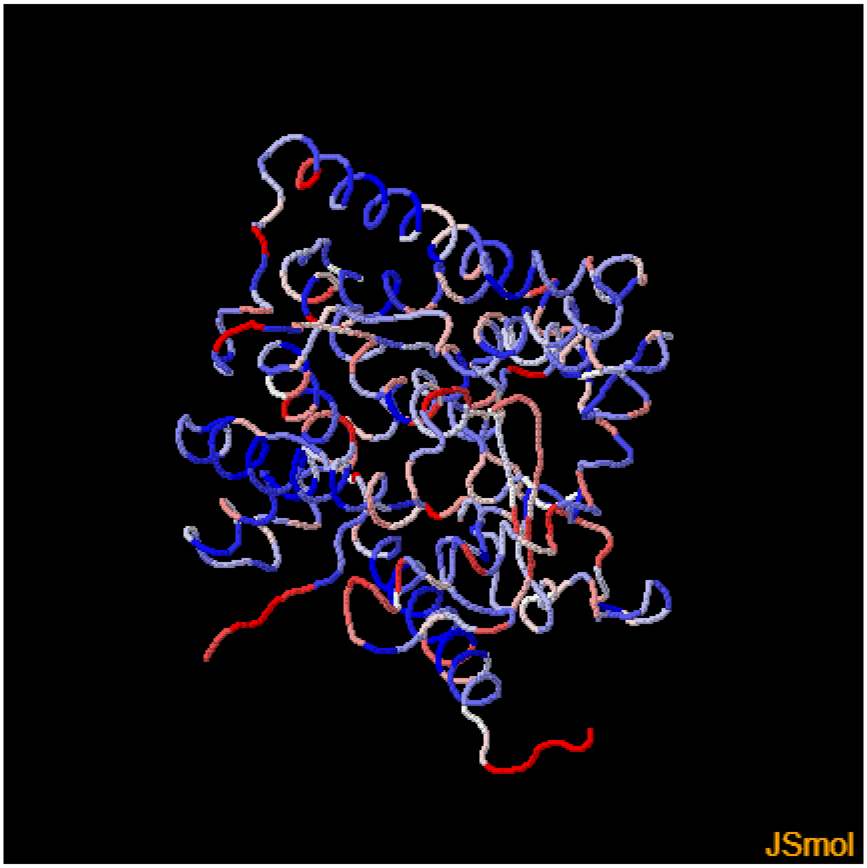

Lowest energy Highest energy

Please cite the following articles if you publish results using ProSA-web:

- Wiederstein & Sippl (2007)
  ProSA-web: interactive web service for the recognition of errors in three-dimensional structures of proteins.
  Nucleic Acids Research 35, W407-W410. [view]
- Sippl, M.J. (1993)
  Recognition of Errors in Three-Dimensional Structures of Proteins.
  Proteins 17, 355-362. [view]

This site is maintained by Markus Wiederstein. For comments and suggestions please contact prosa@came.sbg.ac.at.
