# Supplementary material for: Reverse vaccinology-based design of multivalent multiepitope mRNA vaccines targeting key viral proteins of Herpes Simplex Virus type-2
Source: Front Immunol. 2025 May 20;16:1586271. doi: 10.3389/fimmu.2025.1586271 (PMC12130045; doi:10.3389/fimmu.2025.1586271)
Supplement: Supplementary file 1 [file DataSheet1.zip › Supplementary Data_22-04-2025/Supplementary Data 7B.pdf]

NetSurfP 3.0 Analysis

C1_753

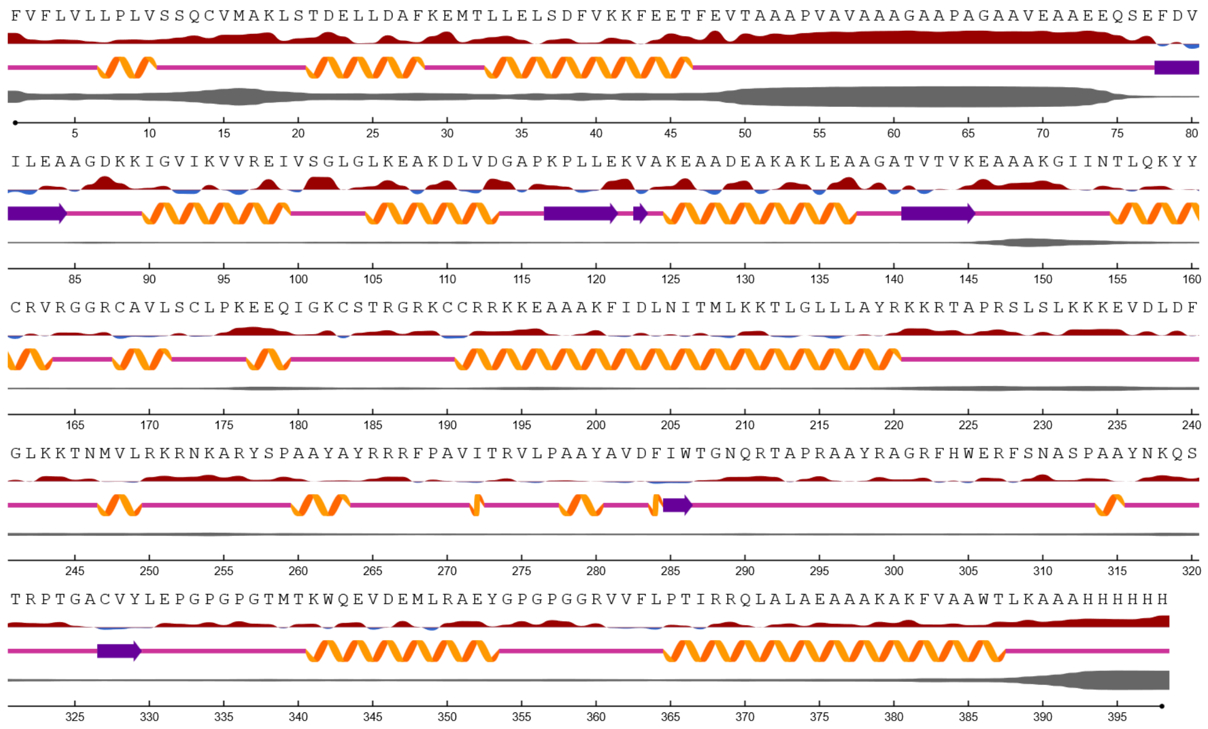

Relative Surface Accessibility: Red is exposed and blue is buried, thresholded at 25%.
Secondary Structure: Helix, Strand, Coil.
Disorder: Thickness of line equals probability of disordered residue.

C2_2625

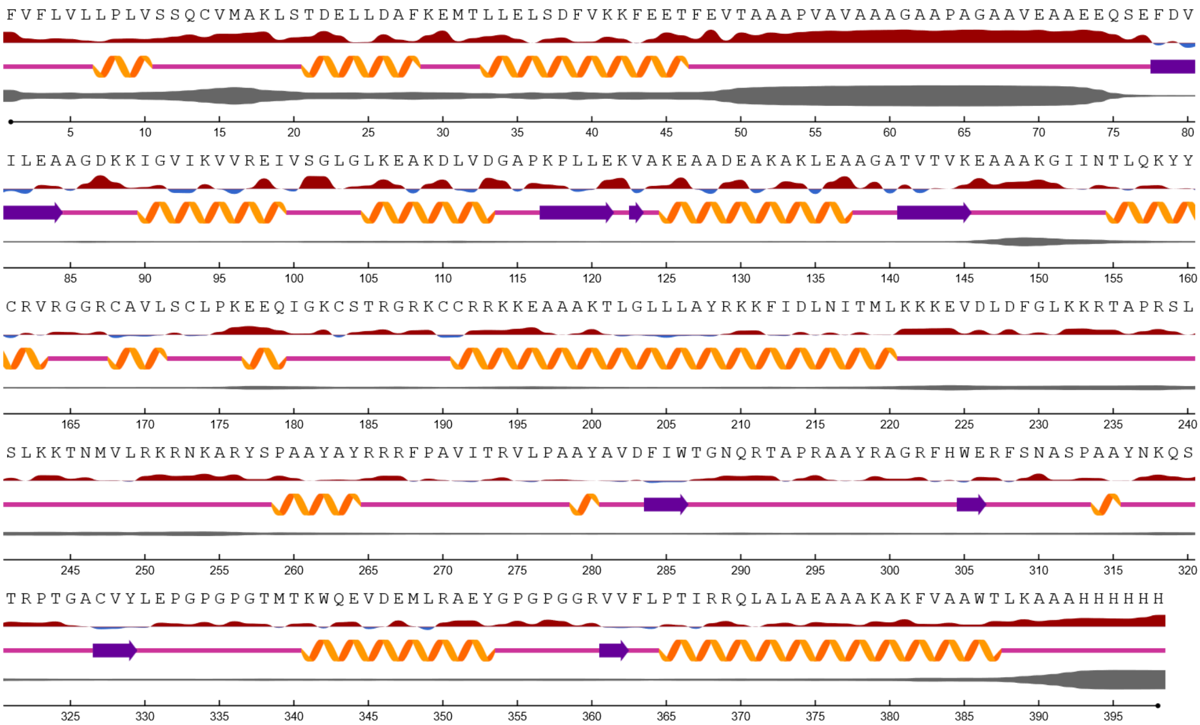

Relative Surface Accessibility: Red is exposed and blue is buried, thresholded at 25%.
Secondary Structure: Helix, Strand, Coil.
Disorder: Thickness of line equals probability of disordered residue.

C3_735

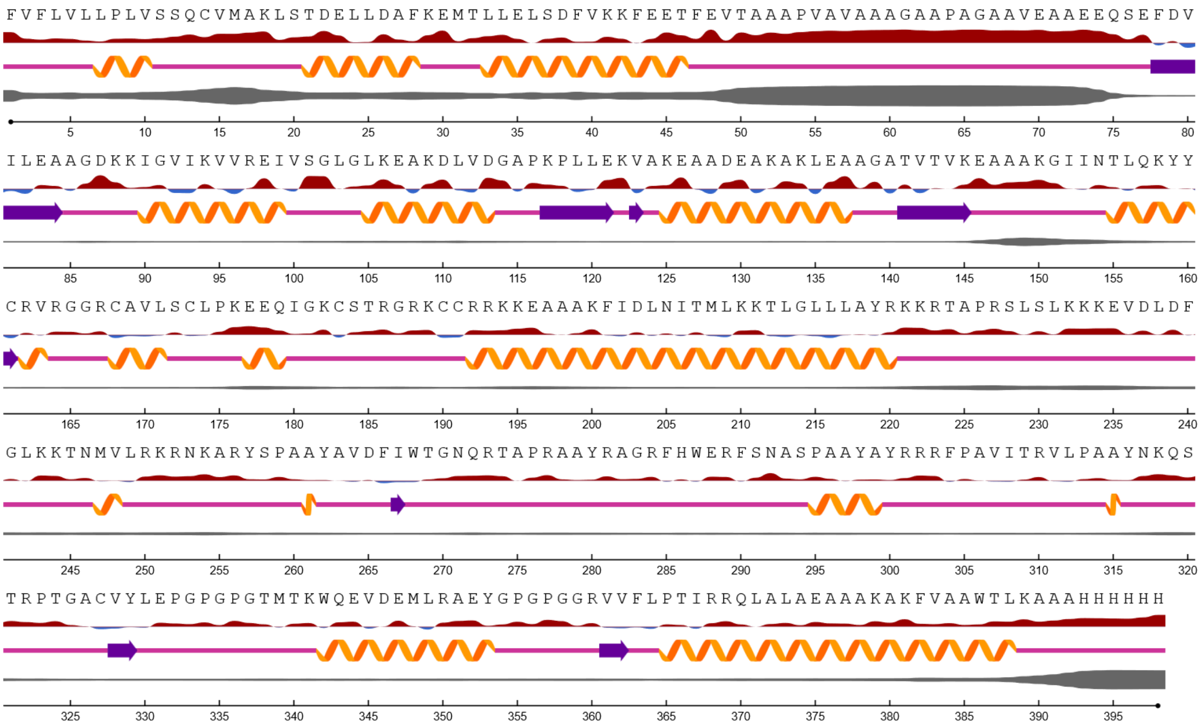

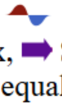
Relative Surface Accessibility: Red is exposed and blue is buried, thresholded at 25%.
Secondary Structure: Helix, Strand, Coil.
Disorder: Thickness of line equals probability of disordered residue.

C4_2607

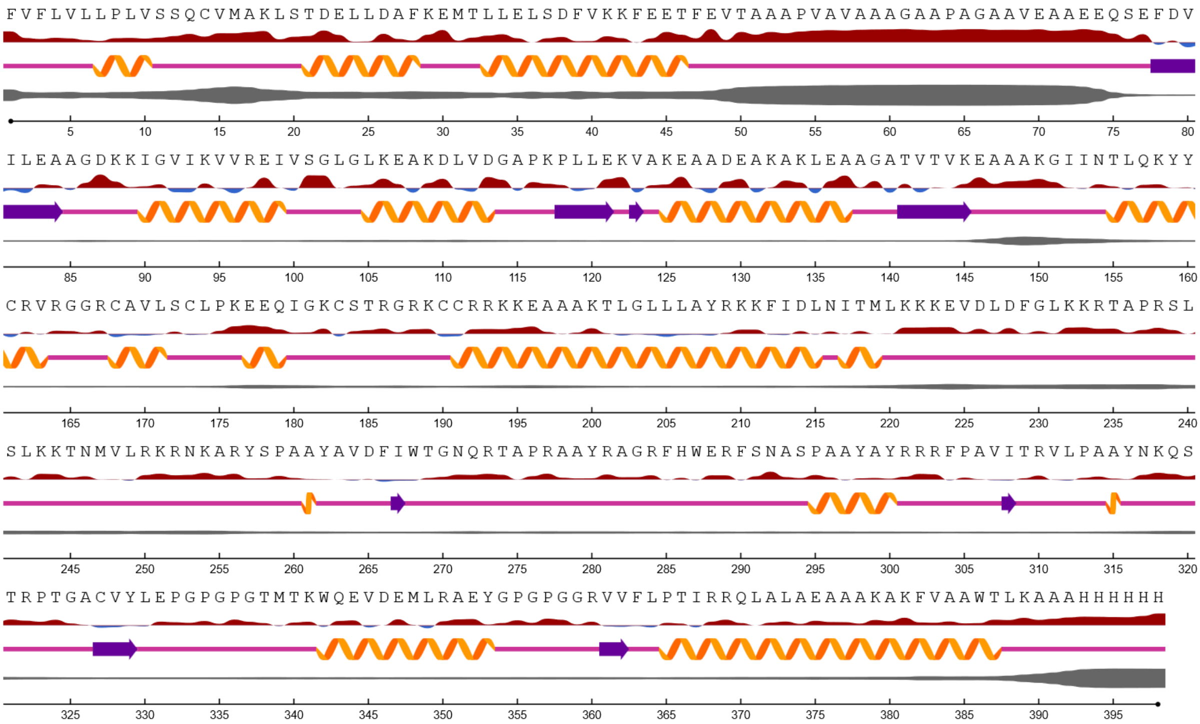

Relative Surface Accessibility: Red is exposed and blue is buried, thresholded at 25%.
Secondary Structure: Helix, Strand, Coil.
Disorder: Thickness of line equals probability of disordered residue.

C5_2769

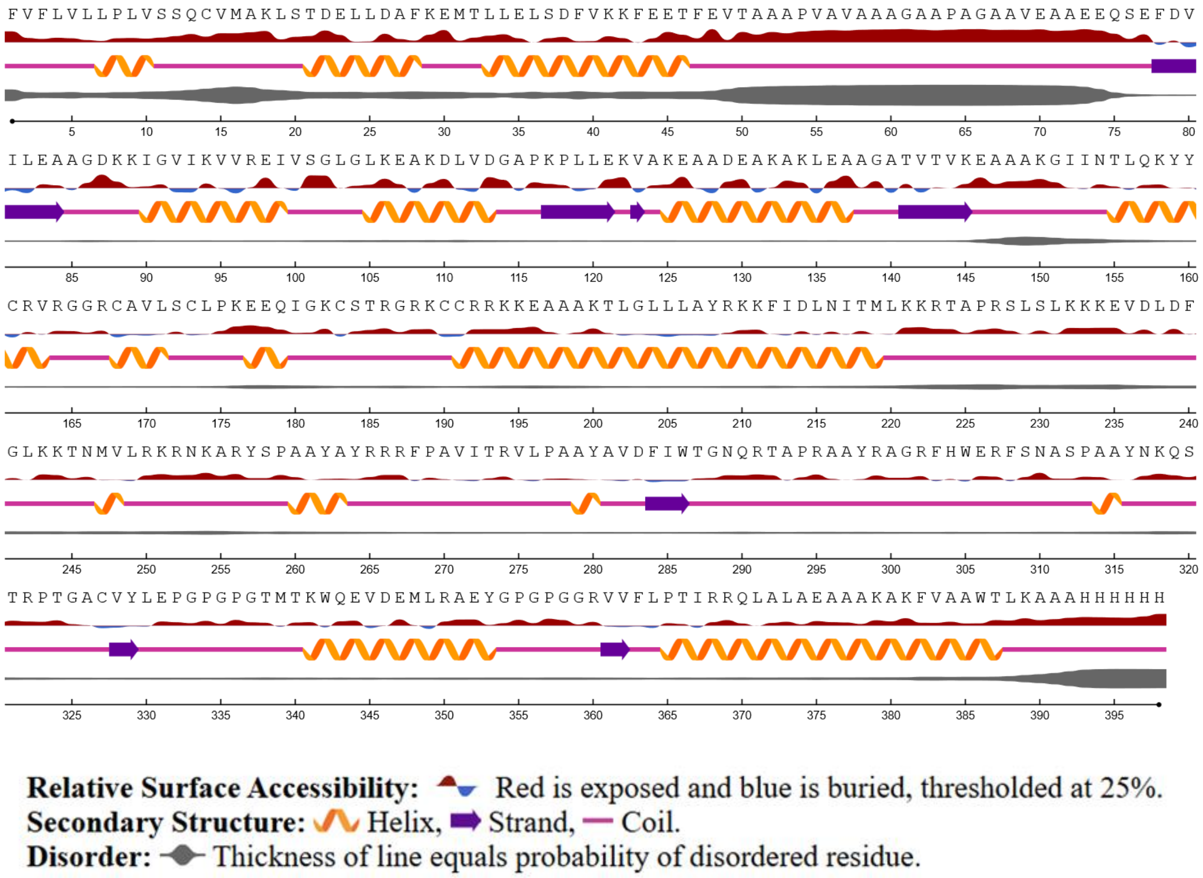
FVFLVLLPLVSSQCVMAKLSTDELLDAFKEMTLLELSDFVKKFEETFEVTAAAPVAVAAAGAAPAGAAVEAAEEQSEFDV
ILEAAGDKKIGVIKVVREIVSGLGLKEAKDLVDGAPKPLLEKVAKEAADEAKAKLEAAGATVTVKEAAAKGIINTLQKYY
CRVRGGRCAVLSCLPKEEQIGKCSTRGRKCCRRKKEAAAKTLGLLLAYRKKFIDLNITMLKKRTAPRSLSLKKKEVDLDF
GLKKTNMVLRKRNKARYSPAAYAYRRRFPAVITRVLPAAYAVDFIWTGNQRTAPRAAYRAGRFHWERFSNASPAAYNKQS
TRPTGACVYLEPGPGPGTMTKWQEVDEMLRAEYGPGPGGRVVFLPTIRRQLALAEAAAKAKFVAAWTLKAAAHHHHHH
Relative Surface Accessibility: Red is exposed and blue is buried, thresholded at 25%.
Secondary Structure: Helix, Strand, Coil.
Disorder: Thickness of line equals probability of disordered residue.
