# Supplementary material for: Reverse vaccinology-based design of multivalent multiepitope mRNA vaccines targeting key viral proteins of Herpes Simplex Virus type-2
Source: Front Immunol. 2025 May 20;16:1586271. doi: 10.3389/fimmu.2025.1586271 (PMC12130045; doi:10.3389/fimmu.2025.1586271)
Supplement: Supplementary file 1 [file DataSheet1.zip › Supplementary Data_22-04-2025/Supplementary Data 8.pdf]

Sequence Patches Heatmap Abpred pka
phosIDP Software About

Protein: 642802_C735
Predicted scaled solubility: 0.660
pI: 10.340

Job ID: 749d3eaab74792cdafc1

These results are shareable via the unique url for 7 days from creation date

POSSIBLE TM REGION PREDICTED
Kyle-Doolitle hydropathy value of 1.69 compared to threshold of 1.6
Protein-sol solubility prediction invalid for membrane proteins

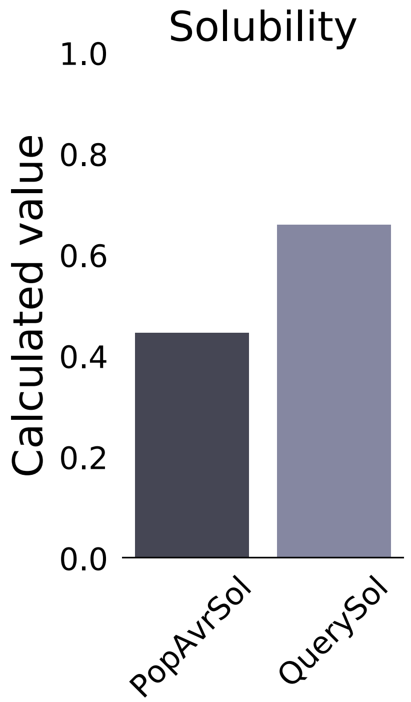

The scaled solubility value (QuerySol) is the predicted solubility. The population average for the experimental dataset (PopAvrSol) is 0.45, and therefore any scaled solubility value greater than 0.45 is predicted to have a higher solubility than the average soluble *E.coli* protein from the experimental solubility dataset Niwa *et al* 2009, and any protein with a lower scaled solubility value is predicted to be less soluble.

The protein-sol sequence algorithm calculated 35 sequence features. This includes the composition of the standard 20 amino acids and sequence length (`len`), as well as the following features which are calculated over a sliding 21 amino acid window.

There are 7 amino acid composite scores:

`KmR` = K minus R, `DmE`=DminusE, `KpR` = K plus R, `DpE` = D+E, `PmN` = K+R-D-E, `PpN` = K+R+D+E, `aro` = F + W + Y

We then calculate a further 7 sequence features:

`fld` = folding propensity Uversky *et al* 2000, `dis` = disorder propensity Linding *et al* 2003, `bet` = beta strand propensities Costantini *et al* 2006, `mem` = Kyte-Doolittle hydropathy Kyte and Doolittle 1982, `pI`, `ent` = sequence entropy, `abs` = absolute charge at pH 7.

Further information is available in the paper.

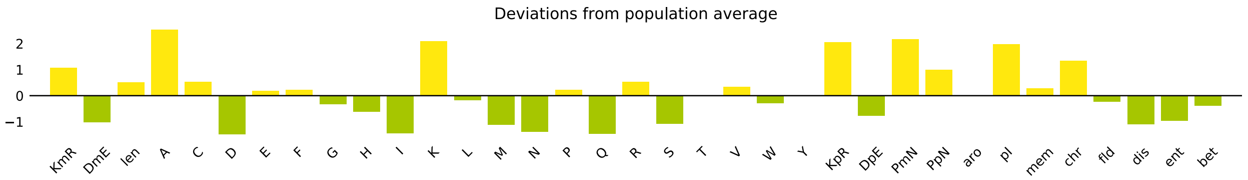

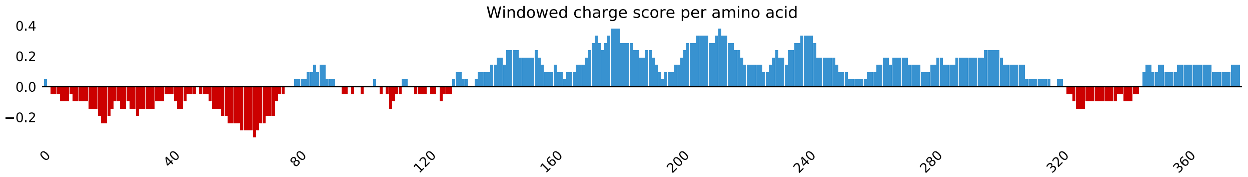

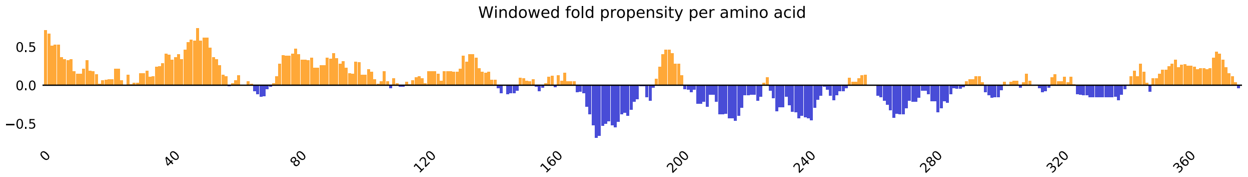

Download Prediction

Citation

Hebditch M, Carballo-Amador M.A., Charonis S, Curtis R, Warwicker J

Protein-Sol: a web tool for predicting protein solubility from sequence.

Bioinformatics (2017)

Sequence Patches Heatmap Abpred pka
phosIDP Software About

Protein: 642799_C753
Predicted scaled solubility: 0.660
pI: 10.340

Job ID: e7463388a2c247c0e2d6
These results are shareable via the unique url for 7 days from creation date

POSSIBLE TM REGION PREDICTED
Kyle-Doolitle hydropathy value of 1.69 compared to threshold of 1.6
Protein-sol solubility prediction invalid for membrane proteins

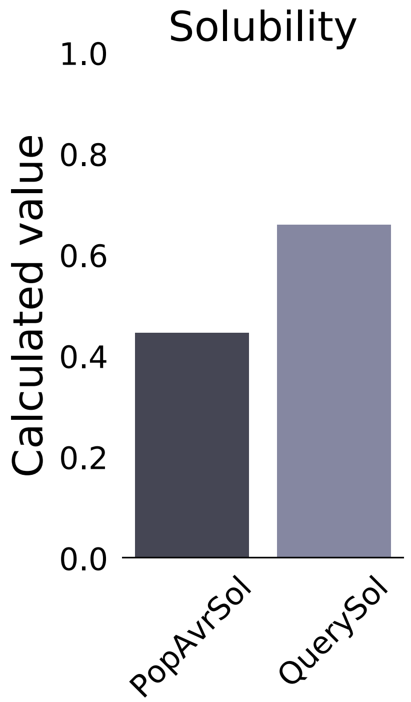

The scaled solubility value (QuerySol) is the predicted solubility. The population average for the experimental dataset (PopAvrSol) is 0.45, and therefore any scaled solubility value greater than 0.45 is predicted to have a higher solubility than the average soluble *E.coli* protein from the experimental solubility dataset Niwa *et al* 2009, and any protein with a lower scaled solubility value is predicted to be less soluble.

The protein-sol sequence algorithm calculated 35 sequence features. This includes the composition of the standard 20 amino acids and sequence length (`len`), as well as the following features which are calculated over a sliding 21 amino acid window.
There are 7 amino acid composite scores:
`KmR` = K minus R, `DmE`=DminusE, `KpR` = K plus R, `DpE` = D+E, `PmN` = K+R-D-E, `PpN` = K+R+D+E, `aro` = F + W + Y
We then calculate a further 7 sequence features:
`fld` = folding propensity Uversky *et al* 2000, `dis` = disorder propensity Linding *et al* 2003, `bet` = beta strand propensities Costantini *et al* 2006, `mem` = Kyte-Doolittle hydropathy Kyte and Doolittle 1982, `pI`, `ent` = sequence entropy, `abs` = absolute charge at pH 7.
Further information is available in the paper.

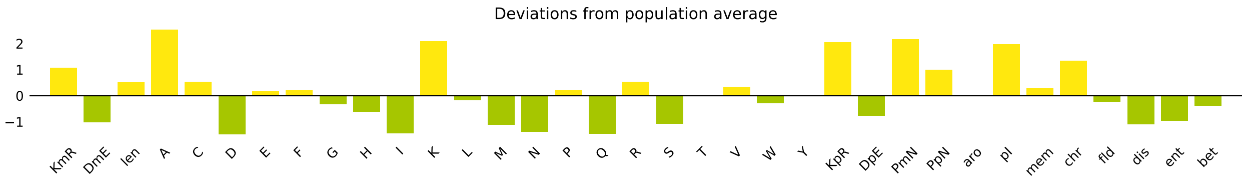

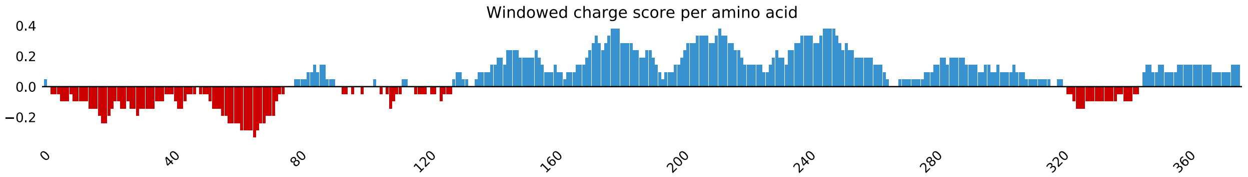

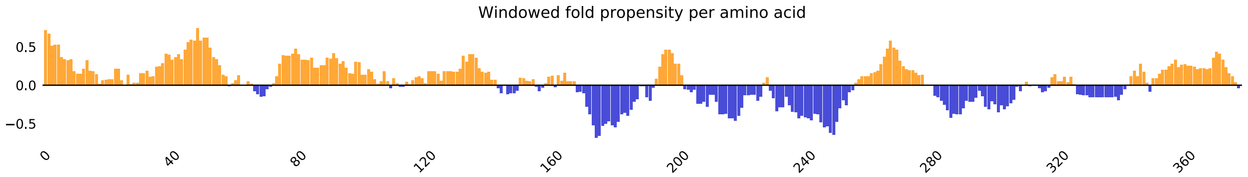

Download Prediction

Citation

Hebditch M, Carballo-Amador M.A., Charonis S, Curtis R, Warwicker J
Protein-Sol: a web tool for predicting protein solubility from sequence.
Bioinformatics (2017)

Sequence Patches Heatmap Abpred pka phosIDP Software About

Protein: 642803_C2607
Predicted scaled solubility: 0.660
pI: 10.340

Job ID: 33ee9a7de597cd5fa3d5
These results are shareable via the unique url for 7 days from creation date

POSSIBLE TM REGION PREDICTED
Kyle-Doolitle hydropathy value of 1.69 compared to threshold of 1.6
Protein-sol solubility prediction invalid for membrane proteins

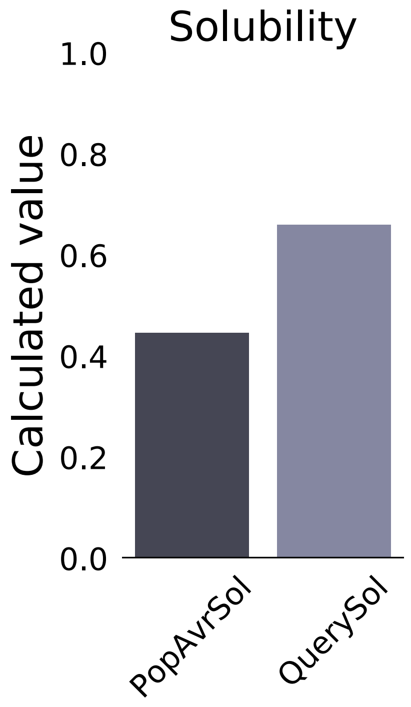

The scaled solubility value (QuerySol) is the predicted solubility. The population average for the experimental dataset (PopAvrSol) is 0.45, and therefore any scaled solubility value greater than 0.45 is predicted to have a higher solubility than the average soluble *E.coli* protein from the experimental solubility dataset Niwa *et al* 2009, and any protein with a lower scaled solubility value is predicted to be less soluble.

The protein-sol sequence algorithm calculated 35 sequence features. This includes the composition of the standard 20 amino acids and sequence length (`len`), as well as the following features which are calculated over a sliding 21 amino acid window.
There are 7 amino acid composite scores:
`KmR` = K minus R, `DmE`=DminusE, `KpR` = K plus R, `DpE` = D+E, `PmN` = K+R-D-E, `PpN` = K+R+D+E, `aro` = F + W + Y
We then calculate a further 7 sequence features:
`fld` = folding propensity Uversky *et al* 2000, `dis` = disorder propensity Linding *et al* 2003, `bet` = beta strand propensities Costantini *et al* 2006, `mem` = Kyte-Doolittle hydropathy Kyte and Doolittle 1982, `pI`, `ent` = sequence entropy, `abs` = absolute charge at pH 7.
Further information is available in the paper.

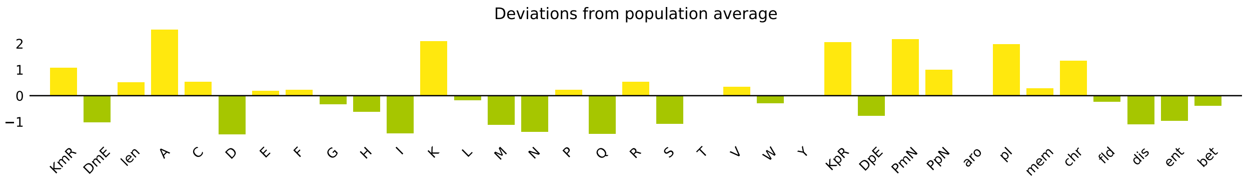

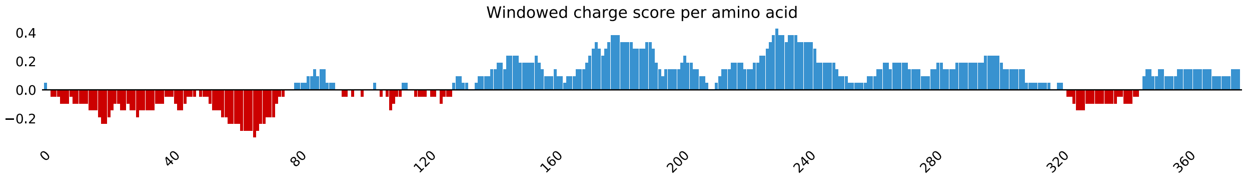

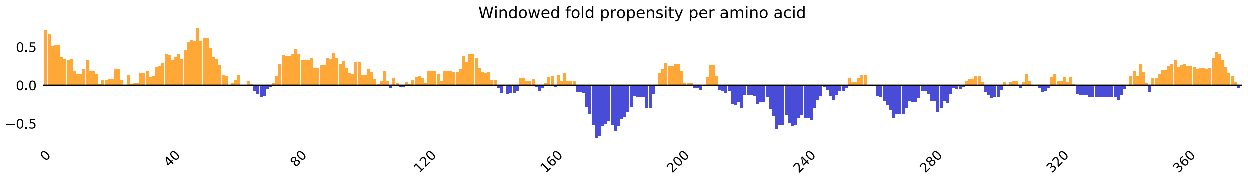

Download Prediction

Citation

Hebditch M, Carballo-Amador M.A., Charonis S, Curtis R, Warwicker J

Protein-Sol: a web tool for predicting protein solubility from sequence.

Bioinformatics (2017)

Sequence Patches Heatmap Abpred pka
phosIDP Software About

Protein: 642801_C2625
Predicted scaled solubility: 0.660
pI: 10.340

Job ID: f198305dbdb025a9444c
These results are shareable via the unique url for 7 days from creation date

POSSIBLE TM REGION PREDICTED
Kyle-Doolitle hydropathy value of 1.69 compared to threshold of 1.6
Protein-sol solubility prediction invalid for membrane proteins

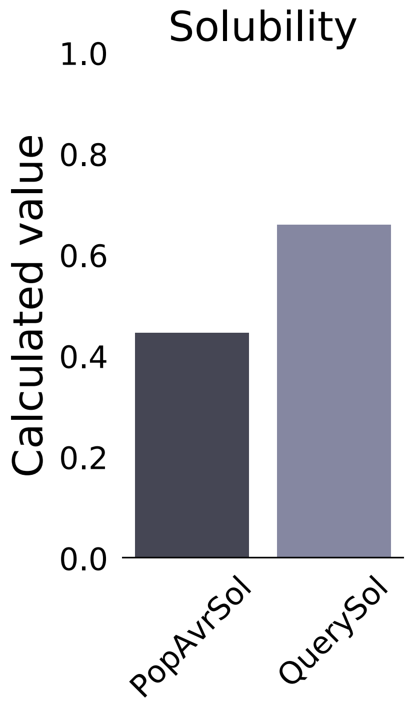

The scaled solubility value (QuerySol) is the predicted solubility. The population average for the experimental dataset (PopAvrSol) is 0.45, and therefore any scaled solubility value greater than 0.45 is predicted to have a higher solubility than the average soluble *E.coli* protein from the experimental solubility dataset Niwa *et al* 2009, and any protein with a lower scaled solubility value is predicted to be less soluble.

The protein-sol sequence algorithm calculated 35 sequence features. This includes the composition of the standard 20 amino acids and sequence length (`len`), as well as the following features which are calculated over a sliding 21 amino acid window.

There are 7 amino acid composite scores:

`KmR` = K minus R, `DmE`=DminusE, `KpR` = K plus R, `DpE` = D+E, `PmN` = K+R-D-E, `PpN` = K+R+D+E, `aro` = F + W + Y

We then calculate a further 7 sequence features:

`fld` = folding propensity Uversky *et al* 2000, `dis` = disorder propensity Linding *et al* 2003, `bet` = beta strand propensities Costantini *et al* 2006, `mem` = Kyte-Doolittle hydropathy Kyte and Doolittle 1982, `pI`, `ent` = sequence entropy, `abs` = absolute charge at pH 7.

Further information is available in the paper.

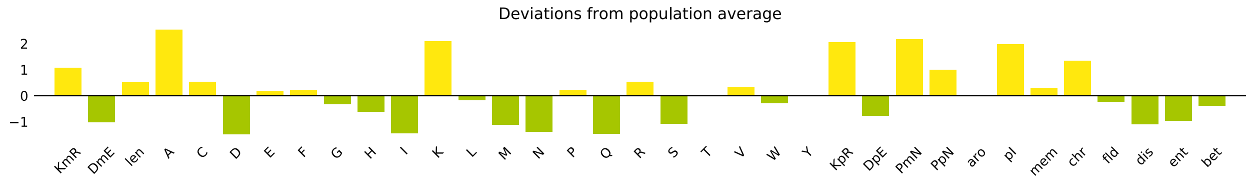

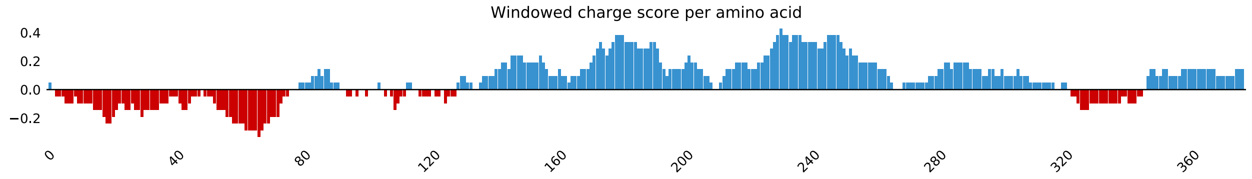

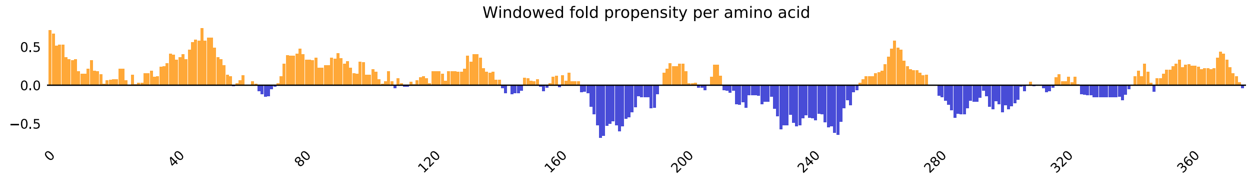

Download Prediction

Citation

Hebditch M, Carballo-Amador M.A., Charonis S, Curtis R, Warwicker J

Protein-Sol: a web tool for predicting protein solubility from sequence.

Bioinformatics (2017)

Sequence Patches Heatmap Abpred pka
phosIDP Software About

Protein: 642804_C2769
Predicted scaled solubility: 0.660
pI: 10.340

Job ID: 47d7ae8c4d43b58e6fb1
These results are shareable via the unique url for 7 days from creation date

POSSIBLE TM REGION PREDICTED
Kyle-Doolitle hydropathy value of 1.69 compared to threshold of 1.6
Protein-sol solubility prediction invalid for membrane proteins

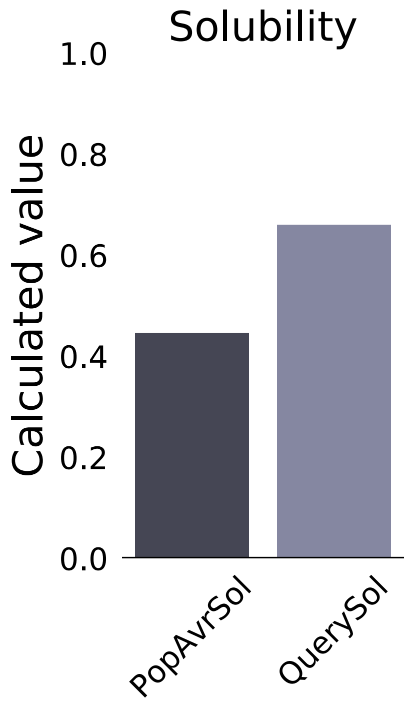

The scaled solubility value (QuerySol) is the predicted solubility. The population average for the experimental dataset (PopAvrSol) is 0.45, and therefore any scaled solubility value greater than 0.45 is predicted to have a higher solubility than the average soluble *E.coli* protein from the experimental solubility dataset Niwa *et al* 2009, and any protein with a lower scaled solubility value is predicted to be less soluble.

The protein-sol sequence algorithm calculated 35 sequence features. This includes the composition of the standard 20 amino acids and sequence length (`len`), as well as the following features which are calculated over a sliding 21 amino acid window.
There are 7 amino acid composite scores:
`KmR` = K minus R, `DmE`=DminusE, `KpR` = K plus R, `DpE` = D+E, `PmN` = K+R-D-E, `PpN` = K+R+D+E, `aro` = F + W + Y
We then calculate a further 7 sequence features:
`fld` = folding propensity Uversky *et al* 2000, `dis` = disorder propensity Linding *et al* 2003, `bet` = beta strand propensities Costantini *et al* 2006, `mem` = Kyte-Doolittle hydropathy Kyte and Doolittle 1982, `pI`, `ent` = sequence entropy, `abs` = absolute charge at pH 7.
Further information is available in the paper.

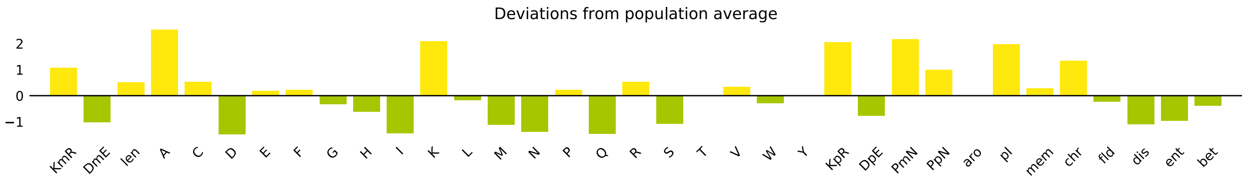

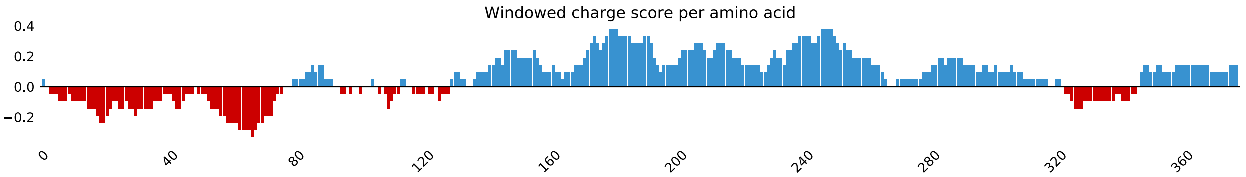

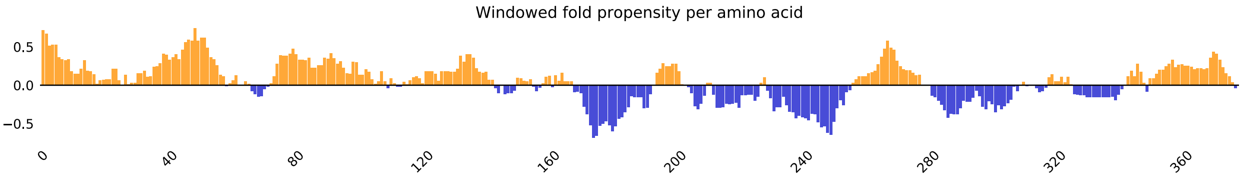

Download Prediction

Citation

Hebditch M, Carballo-Amador M.A., Charonis S, Curtis R, Warwicker J
Protein-Sol: a web tool for predicting protein solubility from sequence.
Bioinformatics (2017)
