# Supplementary material for: Reverse vaccinology-based design of multivalent multiepitope mRNA vaccines targeting key viral proteins of Herpes Simplex Virus type-2
Source: Front Immunol. 2025 May 20;16:1586271. doi: 10.3389/fimmu.2025.1586271 (PMC12130045; doi:10.3389/fimmu.2025.1586271)
Supplement: Supplementary file 1 [file DataSheet1.zip › Supplementary Data_22-04-2025/Supplementary Data 9.pdf]

GenSmart Optimization Report

(Tool Version Beta 1.0)

**Job ID:**	20241116063649970278
**Date:**	2024-11-16 06:36:49
**Gene Name:**	753
**Expression Host Organism:**	Human
**Sequence Type:**	Protein
**Size:**	383aa
**Excluded enzyme sites:**	

Original Sequence (Original Sequence Length: 383aa, GC%:):

MAKLSTDELLDAFKEMTLLELSDFVKKFEETFEVTAAAPVAVAAAGAAPAGAAVEAAEEQSEFDVILEAAGDKKIGVIKVVREIVSGLGLKEAKDLVDGAPKPLLEKVAKEAADEAKAKL
EAAGATVTVKEAAAKGIINTLQKYYCRVRGGRCAVLSCLPKEEQIGKCSTRGRKCCRRKKEAAAKFIDLNITMLKKTLGLLLAYRKKRTAPRSLSLKKKEVDLDFGLKKTNMVLRKRNKA
RYSPAAYAYRRRFPAVITRVLPAAYAVDFIWTGNQRTAPRAAYRAGRFHWERFSNASPAAYNKQSTRPTGACVYLEPGPGPGTMTKWQEVDEMLRAEYGPGPGGRVVFLPTIRRQL
ALAEAAAKAKFVAAWTLKAAAHHHHHH

Optimized Sequence (Optimized Sequence Length: 1149bp, GC%:60.75%):

ATGGCCAAACTGAGCACCGACGAGCTGCTGGACGCCTTCAAGGAGATGACCCTGCTCGAGCTGTCTGATTTTGTGAAGAAGTTTGAGGAGACATTCGAGGTGACCGCCGCCG
CTCCTGTGGCCGTGGCCGCCGCAGGAGCTGCTCCTGCTGGCGCCGCCGTGGAAGCCGCCGAAGAGCAGAGCGAGTTCGACGTGATCCTGGAAGCCGCTGGCGACAAGAA
GATCGGCGTGATCAAGGTGGTGCGGGAAATCGTGTCCGGCCTCGGCCTGAAGGAAGCCAAGGACCTGGTGGACGGCGCTCCAAAGCCTCTGCTGGAAAAGGTGGCCAAGG
AAGCCGCTGATGAGGCCAAAGCCAAGCTGGAAGCCGCCGGCGCTACAGTGACCGTGAAGGAGGCCGCTGCCAAAGGCATCATCAACACCCTGCAGAAATACTACTGCAGAG
TGCGGGGCGGACGGTGCGCCGTGCTGTCCTGCCTGCCTAAGGAAGAGCAAATCGGAAAGTGCAGCACAAGAGGCAGAAAATGTTGTAGACGGAAAAAGGAGGCTGCCGCC
AAGTTCATCGACCTGAATATCACCATGCTGAAAAAGACCCTGGGCCTGCTGCTGGCCTACCGGAAGAAGAGAACCGCCCCCAGAAGCCTGAGCCTGAAGAAAAAGGAAGTGG
ACCTGGATTTCGGCCTGAAGAAGACCAACATGGTCCTGCGGAAGCGGAACAAGGCCAGATACAGCCCTGCCGCCTACGCCTACAGAAGGCGCTTCCCCGCCGTGATTACAA
GAGTGCTGCCTGCCGCTTATGCCGTTGACTTCATCTGGACCGGCAACCAGAGAACAGCCCCCAGAGCCGCTTATCGGGCCGGACGGTTCCACTGGGAGAGATTCAGCAATG
CCAGCCCCGCTGCCTACAACAAGCAGTCTACAAGACCTACCGGAGCATGCGTGTACCTGGAACCTGGCCCTGGCCCAGGCACCATGACCAAGTGGCAGGAGGTGGATGAGA
TGCTGAGAGCCGAGTACGGCCCCGGACCTGGCGGCAGAGTCGTCTTTCTGCCCACCATCAGAAGACAGCTGGCCCTGGCCGAGGCTGCCGCCAAAGCCAAGTTCGTGGCC
GCATGGACACTGAAGGCCGCCGCCCACCACCACCACCACCAT

GC Content Adjustment:

GC Content Adjustment

After GenSmart Codon Optimization

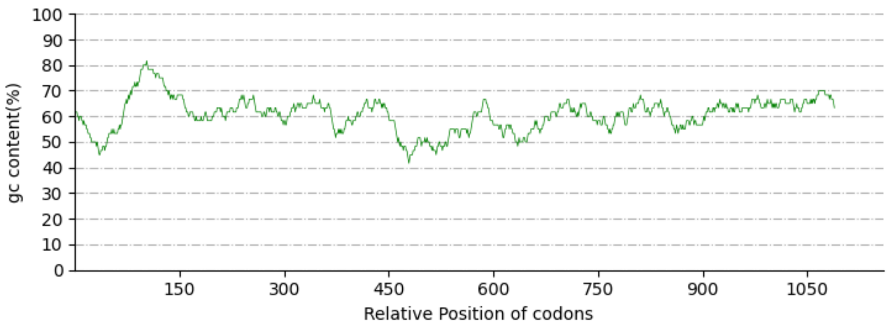

Average GC content:60.75%

Figure 2. The ideal percentage range of GC content is between 30-70 %. Peaks of %GC content in a 60 bp window have been removed.

Conclusion

A wide variety of factors regulate and influence gene expression levels, and our GenSmart codon optimization tool takes into consideration as many of them as possible, but not limited to, codon usage, GC-content, mRNA secondary structure of the genes (e.g., mRNA free energy), cis-acting mRNA destabilizing motifs, RNase splicing sites, and repetitive element.

We are honored to deliver the analysis that you requested. We hope that you are pleased with your GenScript GenSmart codon optimization tool results.

**Job ID:**	20241116063649970278
**Date:**	2024-11-16 06:36:49
**Gene Name:**	2625
**Expression Host Organism:**	Human
**Sequence Type:**	Protein
**Size:**	383aa
**Excluded enzyme sites:**	

Original Sequence (Original Sequence Length: 383aa, GC%:):

MAKLSTDELLDAFKEMTLLELSDFVKKFEETFEVTAAAPVAVAAAGAAPAGAAVEAAEEQSEFDVILEAAGDKKIGVIKVVREIVSGLGLKEAKDLVDGAPKPLLEKVAKEAADEAKAKL
EAAGATVTVKEAAAKGIINTLQKYYCRVRGGRCAVLSCLPKEEQIGKCSTRGRKCCRRKKEAAAKTLGLLLAYRKKFIDLNITMLKKKEVDLDFGLKKRTAPRSLSLKKTNMVLRKRNKA
RYSPAAYAYRRRFPAVITRVLPAAYAVDFIWTGNQRTAPRAAYRAGRFHWERFSNASPAAYNKQSTRPTGACVYLEPGPGPGTMTKWQEVDEMLRAEYGPGPGGRVVFLPTIRRQL
ALAEAAAKAKFVAAWTLKAAAHHHHHH

Optimized Sequence (Optimized Sequence Length: 1149bp, GC%:60.75%):

ATGGCCAAGCTGAGCACCGACGAGCTGCTGGACGCCTTCAAGGAGATGACCCTGCTGGAACTGAGCGACTTTGTGAAGAAATTCGAGGAAACCTTCGAGGTGACCGCCGCC
GCCCCTGTGGCCGTGGCCGCCGCAGGTGCCGCCCCTGCCGGAGCCGCTGTCGAGGCTGCTGAGGAGCAAAGCGAGTTCGACGTGATCCTGGAGGCCGCCGGCGATAAGA
AGATCGGCGTGATTAAGGTGGTGCGGGAAATCGTGTCCGGCCTGGGACTGAAGGAAGCTAAGGACCTGGTGGACGGCGCTCCCAAGCCTCTGCTCGAGAAGGTGGCTAAG
GAAGCCGCAGATGAGGCTAAAGCCAAGCTGGAAGCTGCCGGCGCCACAGTGACAGTCAAGGAGGCCGCCGCTAAAGGCATCATCAACACCTTGCAGAAGTACTACTGCAGA
GTGCGGGGCGGCCGGTGCGCCGTCCTGTCCTGCCTGCCTAAGGAAGAGCAGATCGGCAAGTGTAGCACAAGAGGCAGAAAGTGCTGTAGAAGGAAGAAGGAAGCCGCCGC
CAAGACCCTGGGCCTGCTGCTGGCTTATCGCAAAAAATTCATCGACCTGAACATCACCATGCTGAAGAAGAAGGAAGTGGACCTGGATTTCGGCCTCAAAAAGAGAACCGCC
CCCAGAAGCCTGTCTCTGAAGAAAACCAACATGGTGCTGCGGAAGCGGAATAAGGCCAGATACAGCCCTGCCGCCTACGCCTACAGACGGAGATTCCCCGCTGTTATCACCC
GGGTGCTGCCTGCCGCCTATGCCGTGGATTTTATCTGGACAGGCAACCAGAGAACAGCCCCTAGAGCCGCCTACCGGGCCGGCAGATTCCACTGGGAGAGATTTTCTAATG
CCAGCCCTGCCGCTTACAACAAGCAGAGCACAAGACCTACCGGCGCCTGCGTGTACCTGGAACCTGGCCCCGGCCCAGGAACCATGACAAAGTGGCAGGAGGTGGACGAG
ATGCTGAGAGCCGAGTACGGCCCTGGCCCAGGAGGAAGAGTGGTGTTCCTGCCCACCATCCGGAGACAGCTGGCCCTGGCCGAGGCCGCCGCCAAAGCCAAGTTCGTGGC
CGCTTGGACCCTGAAAGCCGCAGCCCACCACCACCATCACCAC

GC Content Adjustment:

GC Content Adjustment

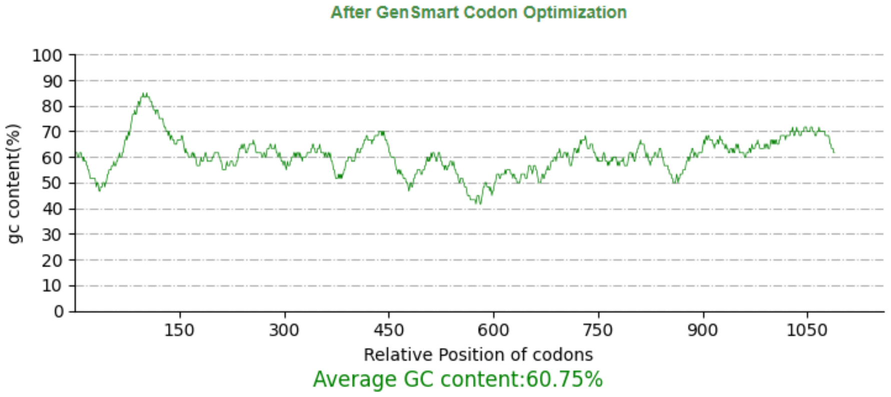

Figure 2. The ideal percentage range of GC content is between 30-70 %. Peaks of %GC content in a 60 bp window have been removed.

Conclusion

A wide variety of factors regulate and influence gene expression levels, and our GenSmart codon optimization tool takes into consideration as many of them as possible, but not limited to, codon usage, GC-content, mRNA secondary structure of the genes (e.g., mRNA free energy), cis-acting mRNA destabilizing motifs, RNase splicing sites, and repetitive element.

We are honored to deliver the analysis that you requested. We hope that you are pleased with your GenScript GenSmart codon optimization tool results.

**Job ID:**	20241116063649970278
**Date:**	2024-11-16 06:36:49
**Gene Name:**	735
**Expression Host Organism:**	Human
**Sequence Type:**	Protein
**Size:**	383aa
**Excluded enzyme sites:**	

Original Sequence (Original Sequence Length: 383aa, GC%:):

MAKLSTDELLDAFKEMTLLELSDFVKKFEETFEVTAAAPVAVAAAGAAPAGAAVEAAEEQSEFDVILEAAGDKKIGVIKVVREIVSGLGLKEAKDLVDGAPKPLLEKVAKEAADEAKAKL
EAAGATVTVKEAAAKGIINTLQKYYCRVRGGRCAVLSCLPKEEQIGKCSTRGRKCCRRKKEAAAKFIDLNITMLKKTLGLLLAYRKKRTAPRSLSLKKKEVDLDFGLKKTNMVLRKRNKA
RYSPAAYAVDFIWTGNQRTAPRAAYRAGRFHWERFSNASPAAYAYRRRFPAVITRVLPAAYNKQSTRPTGACVYLEPGPGPGTMTKWQEVDEMLRAEYGPGPGGRVVFLPTIRRQL
ALAEAAAKAKFVAAWTLKAAAHHHHHH

Optimized Sequence (Optimized Sequence Length: 1149bp, GC%:60.92%):

ATGGCCAAGCTGAGCACTGATGAGCTGCTGGACGCCTTCAAGGAGATGACATTGCTCGAGCTGAGCGACTTCGTGAAAAAGTTCGAGGAAACCTTCGAGGTGACCGCCGCC
GCTCCCGTGGCCGTGGCAGCCGCTGGCGCCGCCCCTGCCGGAGCCGCTGTCGAGGCCGCCGAAGAGCAGTCCGAGTTCGACGTGATCCTGGAAGCCGCCGGCGACAAGA
AAATCGGCGTGATTAAGGTCGTCCGGGAAATCGTGTCCGGCCTGGGACTGAAGGAAGCAAAGGACCTGGTGGACGGCGCCCCAAAGCCTCTGCTGGAGAAGGTGGCCAAA
GAGGCCGCCGACGAGGCCAAAGCCAAGCTGGAGGCTGCCGGCGCCACCGTGACAGTGAAGGAAGCCGCCGCGAAGGGCATCATCAACACCCTGCAAAAGTACTACTGCAG
AGTGCGGGGCGGACGCTGCGCCGTGCTGTCTTGTCTGCCTAAGGAAGAGCAGATCGGCAAGTGTAGCACAAGAGGCAGAAAGTGCTGCAGAAGAAAGAAAGAAGCCGCCG
CTAAGTTCATCGACCTGAATATCACCATGCTGAAGAAGACACTGGGCCTGCTGCTGGCCTACCGGAAGAAGCGGACAGCCCCGCGGAGCCTGTCTCTGAAGAAGAAAGAAGT
GGATCTGGATTTTGGCCTCAAAAAGACCAACATGGTGCTGAGAAAGAGAAACAAGGCCAGATACAGCCCTGCTGCCTATGCCGTGGACTTCATCTGGACCGGCAATCAGAGA
ACCGCCCCTAGAGCCGCTTATAGGGCCGGCAGATTTCACTGGGAGAGATTCAGCAACGCCAGCCCCGCTGCCTACGCCTACAGACGGCGGTTCCCCGCCGTTATCACAAGA
GTGCTGCCCGCCGCTTACAACAAGCAGAGCACCAGACCTACCGGCGCATGCGTGTACCTGGAACCCGGCCCTGGACCTGGGACCATGACCAAGTGGCAGGAGGTGGATGA
GATGCTGCGGGCCGAGTACGGCCCAGGCCCTGGAGGCAGAGTGGTGTTCCTGCCTACAATCAGAAGGCAGCTGGCCCTGGCTGAGGCCGCCGCTAAGGCCAAATTTGTGG
CCGCCTGGACCCTGAAGGCCGCTGCCCACCATCACCACCACCAC

GC Content Adjustment:

GC Content Adjustment

After GenSmart Codon Optimization

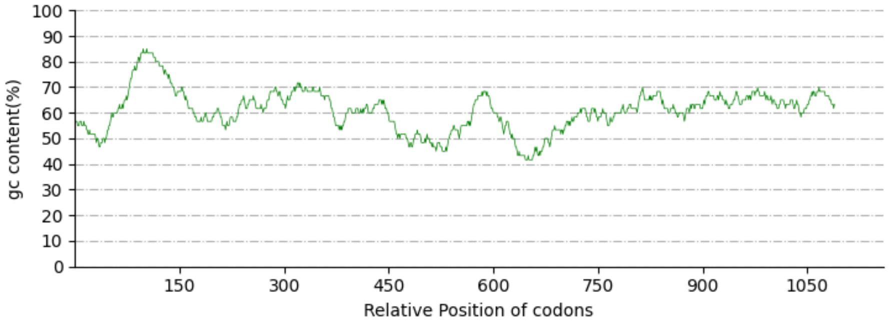

Average GC content:60.92%

Figure 2. The ideal percentage range of GC content is between 30-70 %. Peaks of %GC content in a 60 bp window have been removed.

Conclusion

A wide variety of factors regulate and influence gene expression levels, and our GenSmart codon optimization tool takes into consideration as many of them as possible, but not limited to, codon usage, GC-content, mRNA secondary structure of the genes (e.g., mRNA free energy), cis-acting mRNA destabilizing motifs, RNase splicing sites, and repetitive element.

We are honored to deliver the analysis that you requested. We hope that you are pleased with your GenScript GenSmart codon optimization tool results.

**Job ID:**	20241116063649970278
**Date:**	2024-11-16 06:36:49
**Gene Name:**	2607
**Expression Host Organism:**	Human
**Sequence Type:**	Protein
**Size:**	383aa
**Excluded enzyme sites:**	

Original Sequence (Original Sequence Length: 383aa, GC%:):

MAKLSTDELLDAFKEMTLLELSDFVKKFEETFEVTAAAPVAVAAAGAAPAGAAVEAAEEQSEFDVILEAAGDKKIGVIKVVREIVSGLGLKEAKDLVDGAPKPLLEKVAKEAADEAKAKL
EAAGATVTVKEAAAKGIINTLQKYYCRVRGGRCAVLSCLPKEEQIGKCSTRGRKCCRRKKEAAAKTLGLLLAYRKKFIDLNITMLKKKEVDLDFGLKKRTAPRSLSLKKTNMVLRKRNKA
RYSPAAYAVDFIWTGNQRTAPRAAYRAGRFHWERFSNASPAAYAYRRRFPAVITRVLPAAYNKQSTRPTGACVYLEPGPGPGTMTKWQEVDEMLRAEYGPGPGGRVVFLPTIRRQL
ALAEAAAKAKFVAAWTLKAAAHHHHHH

Optimized Sequence (Optimized Sequence Length: 1149bp, GC%:60.40%):

ATGGCCAAGCTGAGCACTGATGAGCTGCTGGATGCCTTCAAGGAGATGACCCTGCTGGAACTGAGCGACTTCGTGAAGAAGTTTGAGGAAACCTTCGAGGTGACCGCTGCCG
CTCCCGTGGCCGTTGCTGCCGCCGGAGCCGCTCCAGCCGGCGCTGCCGTCGAGGCCGCTGAGGAACAGTCCGAGTTCGACGTGATCCTGGAAGCTGCAGGCGACAAGAAG
ATCGGAGTGATCAAGGTGGTGCGGGAAATCGTGTCCGGCCTCGGCCTGAAGGAAGCCAAGGACCTGGTCGACGGCGCCCCTAAGCCTCTGCTGGAGAAGGTGGCTAAAGA
GGCCGCTGATGAGGCCAAGGCCAAGCTGGAAGCCGCCGGCGCCACCGTGACAGTGAAAGAAGCTGCCGCTAAGGGCATCATCAATACCCTGCAGAAATACTACTGCAGAGT
GCGGGGCGGACGGTGCGCCGTGCTGTCTTGTCTGCCTAAGGAAGAGCAAATCGGCAAGTGTAGCACAAGAGGCAGAAAGTGCTGCCGCAGAAAGAAGGAGGCTGCAGCCA
AGACACTGGGCCTGCTGCTCGCCTACAGAAAGAAATTCATCGACCTGAATATCACCATGCTGAAGAAGAAAGAGGTGGACCTGGACTTCGGCCTGAAAAAACGGACCGCCCC
AAGAAGCCTGTCTTTGAAGAAAACCAACATGGTGCTGCGGAAGCGGAACAAGGCCAGATACAGCCCTGCCGCTTACGCCGTTGATTTTATCTGGACAGGCAACCAGAGAACC
GCCCCTAGAGCTGCATACCGGGCCGGCAGATTCCACTGGGAGAGATTCAGCAACGCCAGCCCCGCCGCCTATGCCTACAGGAGACGGTTTCCTGCCGTGATTACCAGAGTG
CTGCCCGCCGCCTACAACAAGCAGAGCACCAGACCTACCGGTGCCTGCGTGTACCTGGAACCTGGCCCTGGACCCGGAACAATGACAAAGTGGCAGGAGGTCGACGAGAT
GCTGAGAGCCGAGTACGGCCCTGGCCCCGGCGGCAGAGTGGTGTTCCTGCCTACAATCAGAAGACAGCTGGCCCTGGCCGAGGCCGCCGCCAAAGCCAAGTTCGTGGCCG
CCTGGACCCTGAAGGCCGCCGCCCACCACCACCACCACCAT

GC Content Adjustment:

GC Content Adjustment

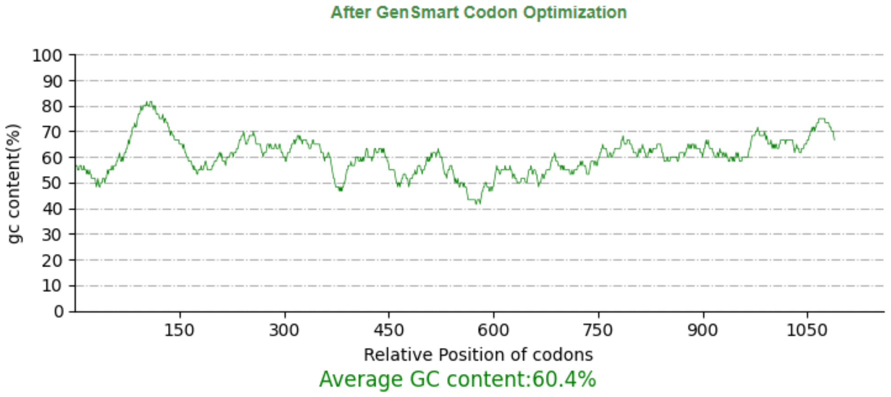

Figure 2. The ideal percentage range of GC content is between 30-70 %. Peaks of %GC content in a 60 bp window have been removed.

Conclusion

A wide variety of factors regulate and influence gene expression levels, and our GenSmart codon optimization tool takes into consideration as many of them as possible, but not limited to, codon usage, GC-content, mRNA secondary structure of the genes (e.g., mRNA free energy), cis-acting mRNA destabilizing motifs, RNase splicing sites, and repetitive element.

We are honored to deliver the analysis that you requested. We hope that you are pleased with your GenScript GenSmart codon optimization tool results.

**Job ID:**	20241116063649970278
**Date:**	2024-11-16 06:36:49
**Gene Name:**	2769
**Expression Host Organism:**	Human
**Sequence Type:**	Protein
**Size:**	383aa
**Excluded enzyme sites:**	

Original Sequence (Original Sequence Length: 383aa, GC%:):

MAKLSTDELLDAFKEMTLLELSDFVKKFEETFEVTAAAPVAVAAAGAAPAGAAVEAAEEQSEFDVILEAAGDKKIGVIKVVREIVSGLGLKEAKDLVDGAPKPLLEKVAKEAADEAKAKL
EAAGATVTVKEAAAKGIINTLQKYYCRVRGGRCAVLSCLPKEEQIGKCSTRGRKCCRRKKEAAAKTLGLLLAYRKKFIDLNITMLKKRTAPRSLSLKKKEVDLDFGLKKTNMVLRKRNKA
RYSPAAYAYRRRFPAVITRVLPAAYAVDFIWTGNQRTAPRAAYRAGRFHWERFSNASPAAYNKQSTRPTGACVYLEPGPGPGTMTKWQEVDEMLRAEYGPGPGGRVVFLPTIRRQL
ALAEAAAKAKFVAAWTLKAAAHHHHHH

Optimized Sequence (Optimized Sequence Length: 1149bp, GC%:60.40%):

ATGGCCAAGCTGTCCACAGACGAGCTGCTGGATGCCTTCAAGGAAATGACACTGCTCGAACTGAGCGACTTCGTGAAGAAGTTTGAGGAAACCTTCGAGGTGACCGCCGCTG
CCCCTGTGGCCGTGGCCGCCGCCGGCGCCGCCCCTGCCGGCGCCGCCGTGGAAGCCGCTGAGGAACAGAGCGAGTTCGACGTGATCCTGGAAGCCGCCGGAGATAAGAA
AATCGGAGTGATTAAGGTGGTGCGGGAAATCGTGTCCGGCCTTGGACTGAAGGAGGCCAAGGACCTGGTGGACGGCGCTCCAAAGCCTCTGTTGGAGAAGGTCGCTAAGGA
GGCTGCAGATGAGGCCAAAGCCAAACTGGAGGCCGCCGGCGCCACCGTGACCGTGAAGGAAGCTGCCGCCAAGGGCATCATCAACACCCTGCAGAAGTACTACTGCAGAG
TGCGGGGTGGCAGATGCGCCGTCCTGTCTTGTCTGCCTAAAGAAGAGCAAATCGGCAAGTGCAGCACAAGAGGCAGAAAGTGCTGTAGAAGGAAGAAGGAAGCCGCTGCCA
AGACCCTGGGCCTGCTGCTCGCCTATAGAAAGAAGTTCATCGACCTGAACATCACCATGCTGAAGAAAAGAACCGCCCCTAGAAGCCTGAGCCTGAAAAAGAAAGAGGTGGA
CCTGGACTTCGGCCTGAAGAAAACCAATATGGTGCTGAGAAAGCGGAACAAGGCTAGATACAGCCCCGCTGCCTACGCCTACCGGAGACGGTTCCCCGCTGTCATCACAAGA
GTGCTGCCAGCCGCTTATGCCGTGGACTTCATCTGGACTGGCAACCAGAGAACAGCCCCCAGAGCCGCTTACAGAGCCGGCAGATTCCACTGGGAGCGGTTTAGCAATGCC
TCTCCAGCGGCCTACAACAAGCAGAGCACCCGGCCTACAGGCGCCTGCGTGTACCTGGAACCTGGCCCTGGACCTGGCACCATGACCAAGTGGCAGGAGGTTGATGAGATG
CTGCGGGCCGAGTACGGCCCTGGACCCGGCGGCCGCGTTGTGTTCCTGCCCACCATCAGAAGGCAGCTGGCCCTGGCCGAGGCTGCCGCTAAAGCCAAGTTTGTGGCCGC
CTGGACACTGAAGGCCGCCGCCCACCACCACCATCACCAC

GC Content Adjustment:

GC Content Adjustment

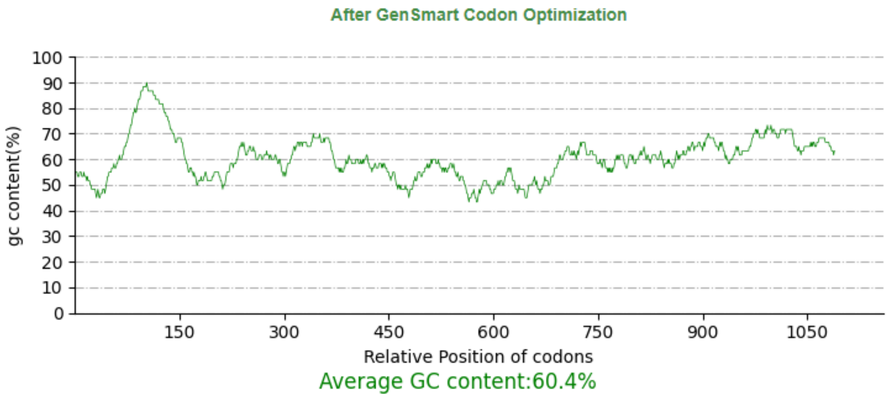

Figure 2. The ideal percentage range of GC content is between 30-70 %. Peaks of %GC content in a 60 bp window have been removed.

Conclusion

A wide variety of factors regulate and influence gene expression levels, and our GenSmart codon optimization tool takes into consideration as many of them as possible, but not limited to, codon usage, GC-content, mRNA secondary structure of the genes (e.g., mRNA free energy), cis-acting mRNA destabilizing motifs, RNase splicing sites, and repetitive element.

We are honored to deliver the analysis that you requested. We hope that you are pleased with your GenScript GenSmart codon optimization tool results.

GenRCA Rare Codon Analysis Report

Gene Name: 753

Reference Source: Codon Usage Database - Kazusa

Expression Host Organism: Homo sapiens (human)

DNA Sequence Length: 1149

DNA Sequence:

ATGGCCAAACTGAGCACCGACGAGCTGCTGGACGCCTTCAAGGAGATGACCCTGCTCGAG
CTGTCTGATTTTGTGAAGAAGTTTGAGGAGACATTCGAGGTGACCGCCGCCGCTCCTGTG
GCCGTGGCCGCCGCAGGAGCTGCTCCTGCTGGCGCCGCCGTGGAAGCCGCCGAAGAGCAG
AGCGAGTTCGACGTGATCCTGGAAGCCGCTGGCGACAAGAAGATCGGCGTGATCAAGGTG
GTGCGGGAAATCGTGTCCGGCCTCGGCCTGAAGGAAGCCAAGGACCTGGTGGACGGCGCT
CCAAAGCCTCTGCTGGAAAAGGTGGCCAAGGAAGCCGCTGATGAGGCCAAAGCCAAGCTG
GAAGCCGCCGGCGCTACAGTGACCGTGAAGGAGGCCGCTGCCAAAGGCATCATCAACACC
CTGCAGAAATACTACTGCAGAGTGCGGGGCGGACGGTGCGCCGTGCTGTCCTGCCTGCCT
AAGGAAGAGCAAATCGGAAAGTGCAGCACAAGAGGCAGAAAATGTTGTAGACGGAAAAAG
GAGGCTGCCGCCAAGTTCATCGACCTGAATATCACCATGCTGAAAAAGACCCTGGGCCTG
CTGCTGGCCTACCGGAAGAAGAGAACCGCCCCCAGAAGCCTGAGCCTGAAGAAAAAGGAA
GTGGACCTGGATTTCGGCCTGAAGAAGACCAACATGGTCCTGCGGAAGCGGAACAAGGCC
AGATACAGCCCTGCCGCCTACGCCTACAGAAGGCGCTTCCCCGCCGTGATTACAAGAGTG
CTGCCTGCCGCTTATGCCGTTGACTTCATCTGGACCGGCAACCAGAGAACAGCCCCCAGA
GCCGCTTATCGGGCCGGACGGTTCCACTGGGAGAGATTCAGCAATGCCAGCCCCGCTGCC
TACAACAAGCAGTCTACAAGACCTACCGGAGCATGCGTGTACCTGGAACCTGGCCCTGGC
CCAGGCACCATGACCAAGTGGCAGGAGGTGGATGAGATGCTGAGAGCCGAGTACGGCCCC
GGACCTGGCGGCAGAGTCGTCTTTCTGCCCACCATCAGAAGACAGCTGGCCCTGGCCGAG
GCTGCCGCCAAAGCCAAGTTCGTGGCCGCATGGACACTGAAGGCCGCCGCCCACCACCAC
CACCACCAT

Protein Sequence:

MAKLSTDELLDAFKEMTLLELSDFVKKFEETFEVTAAAPVAVAAAGAAPAGAAVEAAEEQ
SEFDVILEAAGDKKIGVIKVVREIVSGLGLKEAKDLVDGAPKPLLEKVAKEAADEAKAKL
EAAGATVTVKEAAAKGIINTLQKYYCRVRGGRCAVLSCLPKEEQIGKCSTRGRKCCRRKK
EAAAKFIDLNITMLKKTLGLLLAYRKKRTAPRSLSLKKKEVDLDFGLKKTNMVLRKRNKA
RYSPAAYAYRRRFPAVITRVLPAAYAVDFIWTGNQRTAPRAAYRAGRFHWERFSNASPAA
YNKQSTRPTGACVYLEPGPGPGTMTKWQEVDEMLRAEYGPGPGGRVVFLPTIRRQLALAE
AAAKAKFVAAWTLKAAAHHHHHH

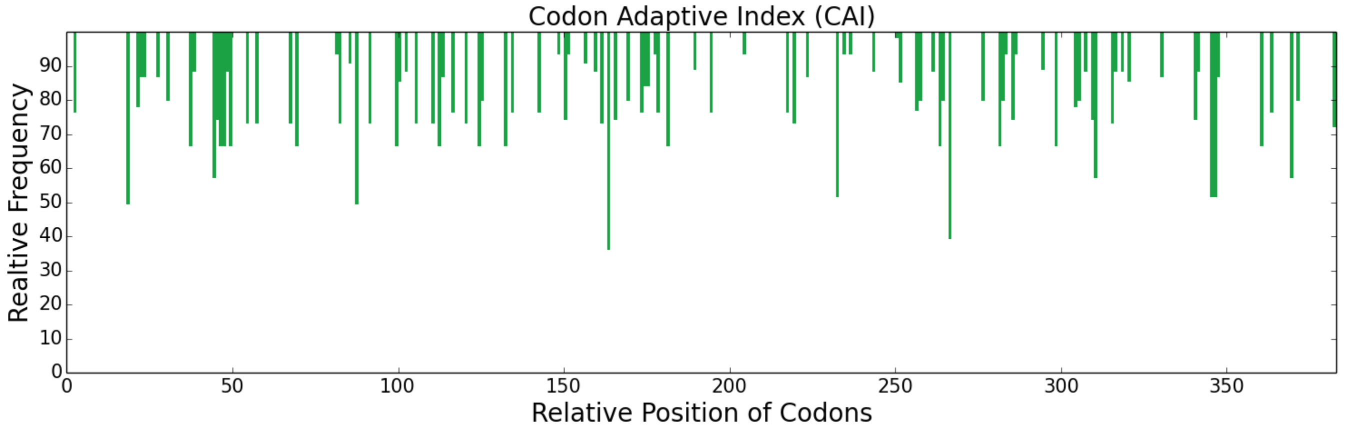

Negative CIS Elements	Negative repeat Elements
0	0

Indices based on non-uniform usage of synonymous codon				
Index	Description	Range	Value	Reference
RSCU	Relative Synonymous Codon Usage	0~6	2.26	[1]
ENC	Effective Number of Codons	20~61	31.83	[2, 3]
RCBS	Relative Codon Bias Strength	≥0	0.77	[4]
DCBS	Directional Codon Bias Score	≥1	2.53	[5]
CDC	Codon Deviation Coefficient	0~1	0.31	[6]
MILC	Measure Independent of Length and Composition	-1~1	-0.01	[7]
ICDI	Intrinsic Codon Deviation Index	0~1	0.4	[8]
SCUO	Synonymous Codon Usage Order	0~1	0.43	[9, 10]
Ew	Weighted Sum of Relative Entropy	0~1	0.54	[11]
P	Codon Preference	≥1	1.55	[12]
MCB	Maximum-likelihood Codon Bias	≥0	1.94	[13]

Indices based on codon frequency in a reference set of genes				
Index	Description	Range	Value	Reference
CAI	Codon Adaptation Index	0~1	0.93	[1]
CFD	Codon Frequency Distribution	0~1	0	[2]
FOP	Frequency of Optimal Codons	0~1	0.73	[3, 4]
COUSIN59	Codon Usage Similarity Index	∞	3.21	[5]
COUSIN18			3.75	
CBI	Codon Bias Index	-1~1	0.61	[6]
Dmean	Mean Dissimilarity-based Index	0~2	0.2	[7]

RCA	Relative Codon Adaptation	≥0	1.25	[8]
CUFS	Codon Usage Frequency Similarity	0~√2	0.39	[9]
B	Codon Usage Bias	0~2	0.63	[10]

Indices based on adaptation to the tRNA levels and their supply				
Index	Description	Range	Value	Reference
tAI	tRNA Adaptation Index	0~1	0.41	[1]
gtAI	Genetic tRNA Adaptation Index	0~1	0.46	[2]
P2	P2 Index	0~1	0.42	[3]

Indices based on complex patterns of codon usage				
Index	Description	Range	Value	Reference
GC3	GC Content at the Third Position of Synonymous Codons	0~1	0.74	[1]
GC	GC Content		0.61	
GC1	GC Content at the First Position of Synonymous Codons		0.61	
GC2	GC Content at the Second Position of Synonymous Codons		0.47	
ENcp	Effective Number of Codon Pairs	20~61	25.25	[2]
CPS	Codon Pair Score	-1~1	-0.13	[3, 4]
Codon Volatility	Codon Volatility	0.5~1	0.74	[5]

Gene Name: 2625

Reference Source: Codon Usage Database - Kazusa

Expression Host Organism: Homo sapiens (human)

DNA Sequence Length: 1149

DNA Sequence:

ATGGCCAAGCTGAGCACCGACGAGCTGCTGGACGCCTTCAAGGAGATGACCCTGCTGGAA
CTGAGCGACTTTGTGAAGAAATTCGAGGAAACCTTCGAGGTGACCGCCGCCGCCCCTGTG
GCCGTGGCCGCCGCAGGTGCCGCCCCTGCCGGAGCCGCTGTCGAGGCTGCTGAGGAGCAA
AGCGAGTTCGACGTGATCCTGGAGGCCGCCGGCGATAAGAAGATCGGCGTGATTAAGGTG
GTGCGGGAAATCGTGTCCGGCCTGGGACTGAAGGAAGCTAAGGACCTGGTGGACGGCGCT
CCCAAGCCTCTGCTCGAGAAGGTGGCTAAGGAAGCCGCAGATGAGGCTAAAGCCAAGCTG
GAAGCTGCCGGCGCCACAGTGACAGTCAAGGAGGCCGCCGCTAAAGGCATCATCAACACC
TTGCAGAAGTACTACTGCAGAGTGCGGGGCGGCCGGTGCGCCGTCCTGTCCTGCCTGCCT
AAGGAAGAGCAGATCGGCAAGTGTAGCACAAGAGGCAGAAAGTGCTGTAGAAGGAAGAAG
GAAGCCGCCGCCAAGACCCTGGGCCTGCTGCTGGCTTATCGCAAAAAATTCATCGACCTG
AACATCACCATGCTGAAGAAGAAGGAAGTGGACCTGGATTTCGGCCTCAAAAAGAGAACC
GCCCCCAGAAGCCTGTCTCTGAAGAAAACCAACATGGTGCTGCGGAAGCGGAATAAGGCC
AGATACAGCCCTGCCGCCTACGCCTACAGACGGAGATTCCCCGCTGTTATCACCCGGGTG
CTGCCTGCCGCCTATGCCGTGGATTTTATCTGGACAGGCAACCAGAGAACAGCCCCTAGA
GCCGCCTACCGGGCCGGCAGATTCCACTGGGAGAGATTTTCTAATGCCAGCCCTGCCGCT
TACAACAAGCAGAGCACAAGACCTACCGGCGCCTGCGTGTACCTGGAACCTGGCCCCGGC
CCAGGAACCATGACAAAGTGGCAGGAGGTGGACGAGATGCTGAGAGCCGAGTACGGCCCT
GGCCCAGGAGGAAGAGTGGTGTTCCTGCCCACCATCCGGAGACAGCTGGCCCTGGCCGAG
GCCGCCGCCAAAGCCAAGTTCGTGGCCGCTTGGACCCTGAAAGCCGCAGCCCACCACCAC
CATCACCAC

Protein Sequence:

MAKLSTDELLDAFKEMTLLELSDFVKKFEETFEVTAAAPVAVAAAGAAPAGAAVEAAEEQ
SEFDVILEAAGDKKIGVIKVVREIVSGLGLKEAKDLVDGAPKPLLEKVAKEAADEAKAKL
EAAGATVTVKEAAAKGIINTLQKYYCRVRGGRCAVLSCLPKEEQIGKCSTRGRKCCRRKK
EAAAKTLGLLLAYRKKFIDLNITMLKKKEVDLDFGLKKRTAPRSLSLKKTNMVLRKRNKA
RYSPAAYAYRRRFPAVITRVLPAAYAVDFIWTGNQRTAPRAAYRAGRFHWERFSNASPAA
YNKQSTRPTGACVYLEPGPGPGTMTKWQEVDEMLRAEYGPGPGGRVVFLPTIRRQLALAE
AAAKAKFVAAWTLKAAAHHHHHH

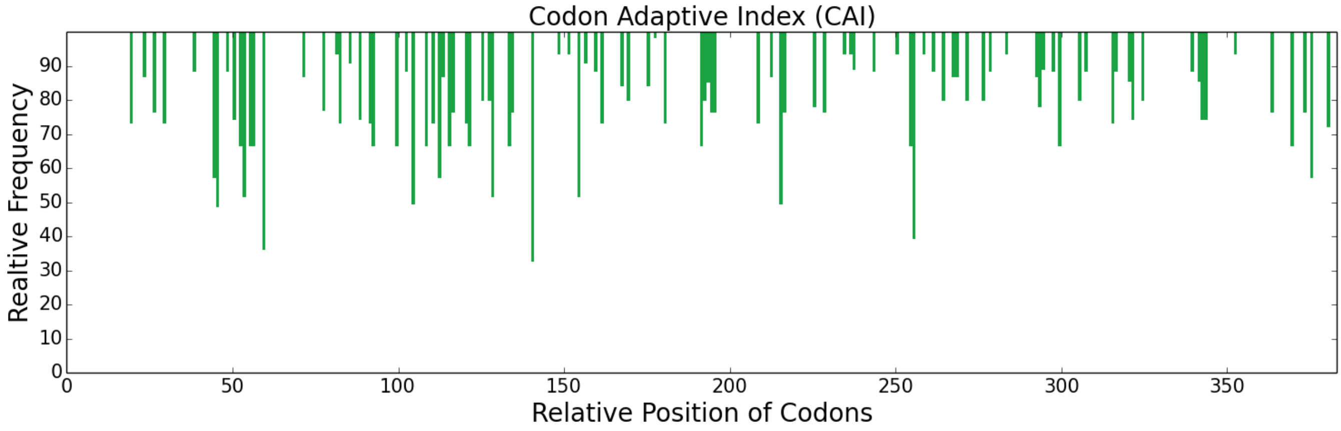

Negative CIS Elements	Negative repeat Elements
0	0

Indices based on non-uniform usage of synonymous codon

Index	Description	Range	Value	Reference
RSCU	Relative Synonymous Codon Usage	0~6	2.24	[1]
ENC	Effective Number of Codons	20~61	31.84	[2, 3]
RCBS	Relative Codon Bias Strength	≥0	0.75	[4]
DCBS	Directional Codon Bias Score	≥1	2.53	[5]
CDC	Codon Deviation Coefficient	0~1	0.31	[6]
MILC	Measure Independent of Length and Composition	-1~1	-0.02	[7]
ICDI	Intrinsic Codon Deviation Index	0~1	0.4	[8]
SCUO	Synonymous Codon Usage Order	0~1	0.42	[9, 10]
Ew	Weighted Sum of Relative Entropy	0~1	0.55	[11]
P	Codon Preference	≥1	1.53	[12]
MCB	Maximum-likelihood Codon Bias	≥0	1.92	[13]

Indices based on codon frequency in a reference set of genes

Index	Description	Range	Value	Reference
CAI	Codon Adaptation Index	0~1	0.93	[1]
CFD	Codon Frequency Distribution	0~1	0	[2]
FOP	Frequency of Optimal Codons	0~1	0.73	[3, 4]
COUSIN59	Codon Usage Similarity Index	∞	3.22	[5]
COUSIN18			3.76	
CBI	Codon Bias Index	-1~1	0.61	[6]
Dmean	Mean Dissimilarity-based Index	0~2	0.2	[7]

RCA	Relative Codon Adaptation	≥0	1.25	[8]
CUFS	Codon Usage Frequency Similarity	0~√2	0.39	[9]
B	Codon Usage Bias	0~2	0.64	[10]

Indices based on adaptation to the tRNA levels and their supply				
Index	Description	Range	Value	Reference
tAI	tRNA Adaptation Index	0~1	0.41	[1]
gtAI	Genetic tRNA Adaptation Index	0~1	0.46	[2]
P2	P2 Index	0~1	0.42	[3]

Indices based on complex patterns of codon usage				
Index	Description	Range	Value	Reference
GC3	GC Content at the Third Position of Synonymous Codons	0~1	0.75	[1]
GC	GC Content		0.61	
GC1	GC Content at the First Position of Synonymous Codons		0.61	
GC2	GC Content at the Second Position of Synonymous Codons		0.47	
ENcp	Effective Number of Codon Pairs	20~61	27.19	[2]
CPS	Codon Pair Score	-1~1	-0.13	[3, 4]
Codon Volatility	Codon Volatility	0.5~1	0.74	[5]

Gene Name: 735

Reference Source: Codon Usage Database - Kazusa

Expression Host Organism: Homo sapiens (human)

DNA Sequence Length: 1149

DNA Sequence:

ATGGCCAAGCTGAGCACTGATGAGCTGCTGGACGCCTTCAAGGAGATGACATTGCTCGAG
CTGAGCGACTTCGTGAAAAAGTTCGAGGAAACCTTCGAGGTGACCGCCGCCGCTCCCGTG
GCCGTGGCAGCCGCTGGCGCCGCCCCTGCCGGAGCCGCTGTCGAGGCCGCCGAAGAGCAG
TCCGAGTTCGACGTGATCCTGGAAGCCGCCGGCGACAAGAAAATCGGCGTGATTAAGGTC
GTCCGGGAAATCGTGTCCGGCCTGGGACTGAAGGAAGCAAAGGACCTGGTGGACGGCGCC
CCAAAGCCTCTGCTGGAGAAGGTGGCCAAAGAGGCCGCCGACGAGGCCAAAGCCAAGCTG
GAGGCTGCCGGCGCCACCGTGACAGTGAAGGAAGCCGCCGCGAAGGGCATCATCAACACC
CTGCAAAAGTACTACTGCAGAGTGCGGGGCGGACGCTGCGCCGTGCTGTCTTGTCTGCCT
AAGGAAGAGCAGATCGGCAAGTGTAGCACAAGAGGCAGAAAGTGCTGCAGAAGAAAGAAA
GAAGCCGCCGCTAAGTTCATCGACCTGAATATCACCATGCTGAAGAAGACACTGGGCCTG
CTGCTGGCCTACCGGAAGAAGCGGACAGCCCCGCGGAGCCTGTCTCTGAAGAAGAAAGAA
GTGGATCTGGATTTTGGCCTCAAAAAGACCAACATGGTGCTGAGAAAGAGAAACAAGGCC
AGATACAGCCCTGCTGCCTATGCCGTGGACTTCATCTGGACCGGCAATCAGAGAACCGCC
CCTAGAGCCGCTTATAGGGCCGGCAGATTTCACTGGGAGAGATTCAGCAACGCCAGCCCC
GCTGCCTACGCCTACAGACGGCGGTTCCCCGCCGTTATCACAAGAGTGCTGCCCGCCGCT
TACAACAAGCAGAGCACCAGACCTACCGGCGCATGCGTGTACCTGGAACCCGGCCCTGGA
CCTGGGACCATGACCAAGTGGCAGGAGGTGGATGAGATGCTGCGGGCCGAGTACGGCCCA
GGCCCTGGAGGCAGAGTGGTGTTCCTGCCTACAATCAGAAGGCAGCTGGCCCTGGCTGAG
GCCGCCGCTAAGGCCAAATTTGTGGCCGCCTGGACCCTGAAGGCCGCTGCCCACCATCAC
CACCACCAC

Protein Sequence:

MAKLSTDELLDAFKEMTLLELSDFVKKFEETFEVTAAAPVAVAAAGAAPAGAAVEAAEEQ
SEFDVILEAAGDKKIGVIKVVREIVSGLGLKEAKDLVDGAPKPLLEKVAKEAADEAKAKL
EAAGATVTVKEAAAKGIINTLQKYYCRVRGGRCAVLSCLPKEEQIGKCSTRGRKCCRRKK
EAAAKFIDLNITMLKKTLGLLLAYRKKRTAPRSLSLKKKEVDLDFGLKKTNMVLRKRNKA
RYSPAAYAVDFIWTGNQRTAPRAAYRAGRFHWERFSNASPAAYAYRRRFPAVITRVLPAA
YNKQSTRPTGACVYLEPGPGPGTMTKWQEVDEMLRAEYGPGPGGRVVFLPTIRRQLALAE
AAAKAKFVAAWTLKAAAHHHHHH

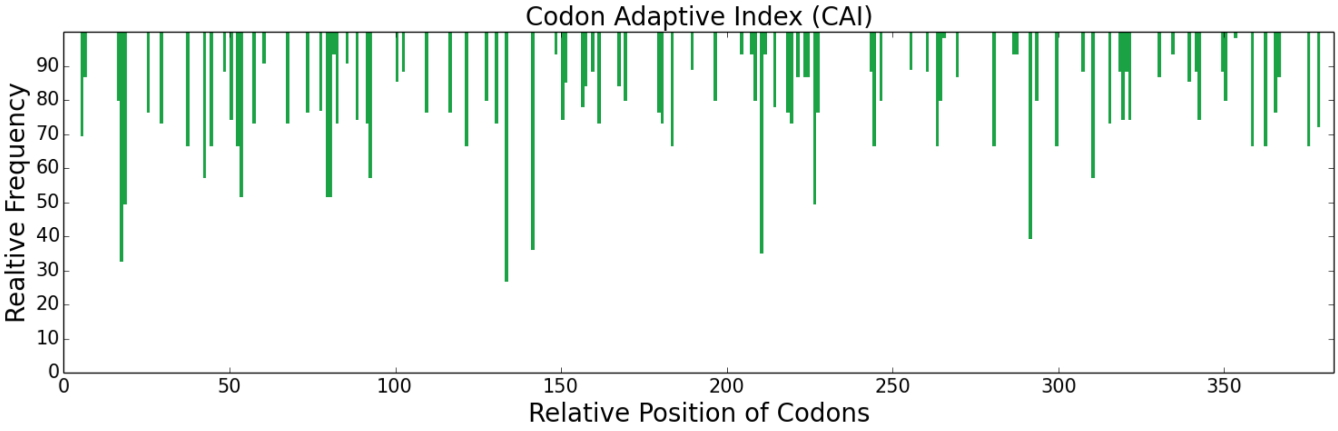

Negative CIS Elements	Negative repeat Elements
0	0

Indices based on non-uniform usage of synonymous codon				
Index	Description	Range	Value	Reference
RSCU	Relative Synonymous Codon Usage	0~6	2.21	[1]
ENC	Effective Number of Codons	20~61	32.24	[2, 3]
RCBS	Relative Codon Bias Strength	≥0	0.69	[4]
DCBS	Directional Codon Bias Score	≥1	2.57	[5]
CDC	Codon Deviation Coefficient	0~1	0.3	[6]
MILC	Measure Independent of Length and Composition	-1~1	-0.05	[7]
ICDI	Intrinsic Codon Deviation Index	0~1	0.4	[8]
SCUO	Synonymous Codon Usage Order	0~1	0.41	[9, 10]
Ew	Weighted Sum of Relative Entropy	0~1	0.57	[11]
P	Codon Preference	≥1	1.48	[12]
MCB	Maximum-likelihood Codon Bias	≥0	1.88	[13]

Indices based on codon frequency in a reference set of genes				
Index	Description	Range	Value	Reference
CAI	Codon Adaptation Index	0~1	0.92	[1]
CFD	Codon Frequency Distribution	0~1	0	[2]
FOP	Frequency of Optimal Codons	0~1	0.73	[3, 4]
COUSIN59	Codon Usage Similarity Index	∞	3.24	[5]
COUSIN18			3.76	
CBI	Codon Bias Index	-1~1	0.61	[6]
Dmean	Mean Dissimilarity-based Index	0~2	0.2	[7]

RCA	Relative Codon Adaptation	≥0	1.24	[8]
CUFS	Codon Usage Frequency Similarity	0~√2	0.37	[9]
B	Codon Usage Bias	0~2	0.63	[10]

Indices based on adaptation to the tRNA levels and their supply				
Index	Description	Range	Value	Reference
tAI	tRNA Adaptation Index	0~1	0.41	[1]
gtAI	Genetic tRNA Adaptation Index	0~1	0.46	[2]
P2	P2 Index	0~1	0.41	[3]

Indices based on complex patterns of codon usage				
Index	Description	Range	Value	Reference
GC3	GC Content at the Third Position of Synonymous Codons	0~1	0.75	[1]
GC	GC Content		0.61	
GC1	GC Content at the First Position of Synonymous Codons		0.6	
GC2	GC Content at the Second Position of Synonymous Codons		0.47	
ENcp	Effective Number of Codon Pairs	20~61	28.3	[2]
CPS	Codon Pair Score	-1~1	-0.15	[3, 4]
Codon Volatility	Codon Volatility	0.5~1	0.74	[5]

Gene Name: 2607

Reference Source: Codon Usage Database - Kazusa

Expression Host Organism: Homo sapiens (human)

DNA Sequence Length: 1149

DNA Sequence:

ATGGCCAAGCTGAGCACTGATGAGCTGCTGGATGCCTTCAAGGAGATGACCCTGCTGGAA
CTGAGCGACTTCGTGAAGAAGTTTGAGGAAACCTTCGAGGTGACCGCTGCCGCTCCCGTG
GCCGTTGCTGCCGCCGGAGCCGCTCCAGCCGGCGCTGCCGTCGAGGCCGCTGAGGAACAG
TCCGAGTTCGACGTGATCCTGGAAGCTGCAGGCGACAAGAAGATCGGAGTGATCAAGGTG
GTGCGGGAAATCGTGTCCGGCCTCGGCCTGAAGGAAGCCAAGGACCTGGTCGACGGCGCC
CCTAAGCCTCTGCTGGAGAAGGTGGCTAAAGAGGCCGCTGATGAGGCCAAGGCCAAGCTG
GAAGCCGCCGGCGCCACCGTGACAGTGAAAGAAGCTGCCGCTAAGGGCATCATCAATACC
CTGCAGAAATACTACTGCAGAGTGCGGGGCGGACGGTGCGCCGTGCTGTCTTGTCTGCCT
AAGGAAGAGCAAATCGGCAAGTGTAGCACAAGAGGCAGAAAGTGCTGCCGCAGAAAGAAG
GAGGCTGCAGCCAAGACACTGGGCCTGCTGCTCGCCTACAGAAAGAAATTCATCGACCTG
AATATCACCATGCTGAAGAAGAAAGAGGTGGACCTGGACTTCGGCCTGAAAAAACGGACC
GCCCCAAGAAGCCTGTCTTTGAAGAAAACCAACATGGTGCTGCGGAAGCGGAACAAGGCC
AGATACAGCCCTGCCGCTTACGCCGTTGATTTTATCTGGACAGGCAACCAGAGAACCGCC
CCTAGAGCTGCATACCGGGCCGGCAGATTCCACTGGGAGAGATTCAGCAACGCCAGCCCC
GCCGCCTATGCCTACAGGAGACGGTTTCCTGCCGTGATTACCAGAGTGCTGCCCGCCGCC
TACAACAAGCAGAGCACCAGACCTACCGGTGCCTGCGTGTACCTGGAACCTGGCCCTGGA
CCCGGAACAATGACAAAGTGGCAGGAGGTCGACGAGATGCTGAGAGCCGAGTACGGCCCT
GGCCCCGGCGGCAGAGTGGTGTTCCTGCCTACAATCAGAAGACAGCTGGCCCTGGCCGAG
GCCGCCGCCAAAGCCAAGTTCGTGGCCGCCTGGACCCTGAAGGCCGCCGCCCACCACCAC
CACCACCAT

Protein Sequence:

MAKLSTDELLDAFKEMTLLELSDFVKKFEETFEVTAAAPVAVAAAGAAPAGAAVEAAEEQ
SEFDVILEAAGDKKIGVIKVVREIVSGLGLKEAKDLVDGAPKPLLEKVAKEAADEAKAKL
EAAGATVTVKEAAAKGIINTLQKYYCRVRGGRCAVLSCLPKEEQIGKCSTRGRKCCRRKK
EAAAKTLGLLLAYRKKFIDLNITMLKKKEVDLDFGLKKRTAPRSLSLKKTNMVLRKRNKA
RYSPAAYAVDFIWTGNQRTAPRAAYRAGRFHWERFSNASPAAYAYRRRFPAVITRVLPAA
YNKQSTRPTGACVYLEPGPGPGTMTKWQEVDEMLRAEYGPGPGGRVVFLPTIRRQLALAE
AAAKAKFVAAWTLKAAAHHHHHH

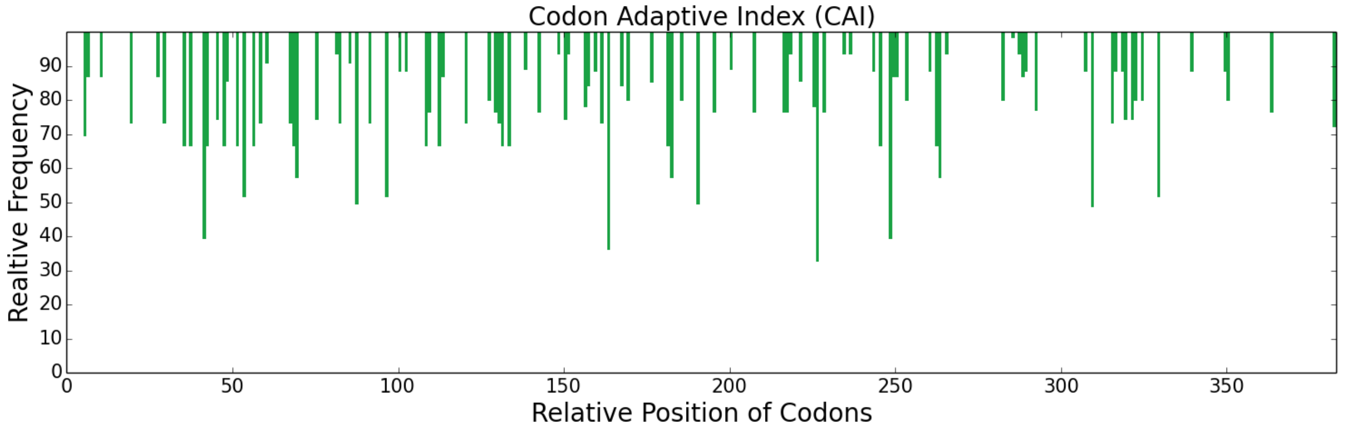

Negative CIS Elements	Negative repeat Elements
0	0

Indices based on non-uniform usage of synonymous codon				
Index	Description	Range	Value	Reference
RSCU	Relative Synonymous Codon Usage	0~6	2.22	[1]
ENC	Effective Number of Codons	20~61	31.83	[2, 3]
RCBS	Relative Codon Bias Strength	≥0	0.74	[4]
DCBS	Directional Codon Bias Score	≥1	2.56	[5]
CDC	Codon Deviation Coefficient	0~1	0.31	[6]
MILC	Measure Independent of Length and Composition	-1~1	-0.04	[7]
ICDI	Intrinsic Codon Deviation Index	0~1	0.41	[8]
SCUO	Synonymous Codon Usage Order	0~1	0.42	[9, 10]
Ew	Weighted Sum of Relative Entropy	0~1	0.56	[11]
P	Codon Preference	≥1	1.52	[12]
MCB	Maximum-likelihood Codon Bias	≥0	1.88	[13]

Indices based on codon frequency in a reference set of genes				
Index	Description	Range	Value	Reference
CAI	Codon Adaptation Index	0~1	0.92	[1]
CFD	Codon Frequency Distribution	0~1	0	[2]
FOP	Frequency of Optimal Codons	0~1	0.73	[3, 4]
COUSIN59	Codon Usage Similarity Index	∞	3.24	[5]
COUSIN18			3.83	
CBI	Codon Bias Index	-1~1	0.6	[6]
Dmean	Mean Dissimilarity-based Index	0~2	0.21	[7]

RCA	Relative Codon Adaptation	≥0	1.24	[8]
CUFS	Codon Usage Frequency Similarity	0~√2	0.38	[9]
B	Codon Usage Bias	0~2	0.63	[10]

Indices based on adaptation to the tRNA levels and their supply				
Index	Description	Range	Value	Reference
tAI	tRNA Adaptation Index	0~1	0.41	[1]
gtAI	Genetic tRNA Adaptation Index	0~1	0.46	[2]
P2	P2 Index	0~1	0.44	[3]

Indices based on complex patterns of codon usage				
Index	Description	Range	Value	Reference
GC3	GC Content at the Third Position of Synonymous Codons	0~1	0.74	[1]
GC	GC Content		0.6	
GC1	GC Content at the First Position of Synonymous Codons		0.6	
GC2	GC Content at the Second Position of Synonymous Codons		0.47	
ENcp	Effective Number of Codon Pairs	20~61	27.35	[2]
CPS	Codon Pair Score	-1~1	-0.15	[3, 4]
Codon Volatility	Codon Volatility	0.5~1	0.74	[5]

Gene Name: 2769

Reference Source: Codon Usage Database - Kazusa

Expression Host Organism: Homo sapiens (human)

DNA Sequence Length: 1149

DNA Sequence:

```
ATGGCCAAGCTGTCCACAGACGAGCTGCTGGATGCCTTCAAGGAAATGACACTGCTCGAA
CTGAGCGACTTCGTGAAGAAGTTTGAGGAAACCTTCGAGGTGACCGCCGCTGCCCCTGTG
GCCGTGGCCGCCGCCGGCGCCGCCCCTGCCGGCGCCGCCGTGGAAGCCGCTGAGGAACAG
AGCGAGTTCGACGTGATCCTGGAAGCCGCCGGAGATAAGAAAATCGGAGTGATTAAGGTG
GTGCGGGAAATCGTGTCCGGCCTTGGACTGAAGGAGGCCAAGGACCTGGTGGACGGCGCT
CCAAAGCCTCTGTTGGAGAAGGTCGCTAAGGAGGCTGCAGATGAGGCCAAAGCCAAACTG
GAGGCCGCCGGCGCCACCGTGACCGTGAAGGAAGCTGCCGCCAAGGGCATCATCAACACC
CTGCAGAAGTACTACTGCAGAGTGCGGGGTGGCAGATGCGCCGTCCTGTCTTGTCTGCCT
AAAGAAGAGCAAATCGGCAAGTGCAGCACAAGAGGCAGAAAGTGCTGTAGAAGGAAGAAG
GAAGCCGCTGCCAAGACCCTGGGCCTGCTGCTCGCCTATAGAAAGAAGTTCATCGACCTG
AACATCACCATGCTGAAGAAAAGAACCGCCCCTAGAAGCCTGAGCCTGAAAAAGAAAGAG
GTGGACCTGGACTTCGGCCTGAAGAAAACCAATATGGTGCTGAGAAAGCGGAACAAGGCT
AGATACAGCCCCGCTGCCTACGCCTACCGGAGACGGTTCCCCGCTGTCATCACAAGAGTG
CTGCCAGCCGCTTATGCCGTGGACTTCATCTGGACTGGCAACCAGAGAACAGCCCCCAGA
GCCGCTTACAGAGCCGGCAGATTCCACTGGGAGCGGTTTAGCAATGCCTCTCCAGCGGCC
TACAACAAGCAGAGCACCCGGCCTACAGGCGCCTGCGTGTACCTGGAACCTGGCCCTGGA
CCTGGCACCATGACCAAGTGGCAGGAGGTTGATGAGATGCTGCGGGCCGAGTACGGCCCT
GGACCCGGCGGCCGCGTTGTGTTCCTGCCCACCATCAGAAGGCAGCTGGCCCTGGCCGAG
GCTGCCGCTAAAGCCAAGTTTGTGGCCGCCTGGACACTGAAGGCCGCCGCCCACCACCAC
CATCACCAC
```

Protein Sequence:

```
MAKLSTDELLDAFKEMTLLELSDFVKKFEETFEVTAAAPVAVAAAGAAPAGAAVEAAEEQ
SEFDVILEAAGDKKIGVIKVVREIVSGLGLKEAKDLVDGAPKPLLEKVAKEAADEAKAKL
EAAGATVTVKEAAAKGIINTLQKYYCRVRGGRCAVLSCLPKEEQIGKCSTRGRKCCRRKK
EAAAKTLGLLLAYRKKFIDLNITMLKKRTAPRSLSLKKKEVDLDFGLKKTNMVLRKRNKA
RYSPAAYAYRRRFPAVITRVLPAAYAVDFIWTGNQRTAPRAAYRAGRFHWERFSNASPAA
YNKQSTRPTGACVYLEPGPGPGTMTKWQEVDEMLRAEYGPGPGGRVVFLPTIRRQLALAE
AAAKAKFVAAWTLKAAAHHHHHH
```

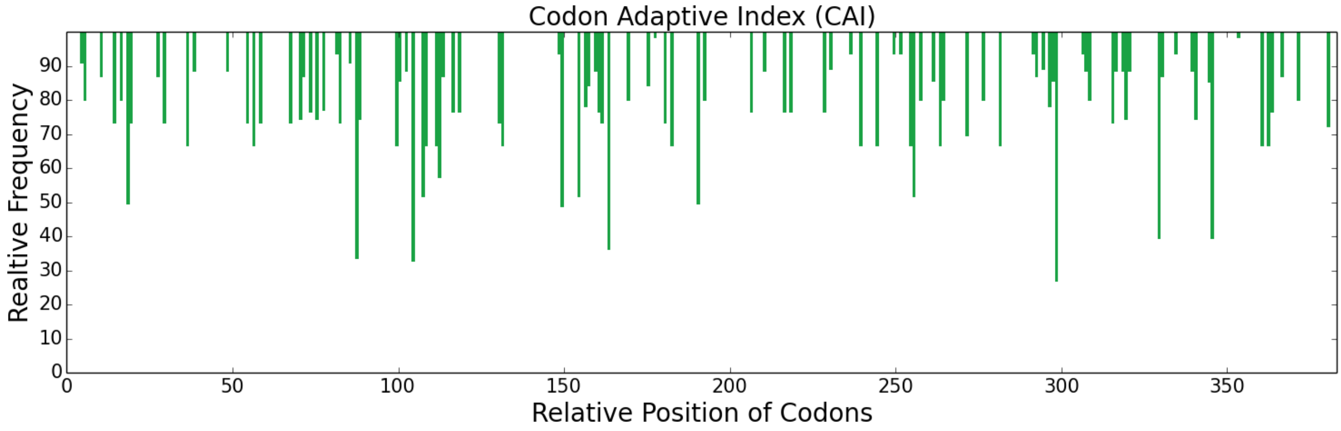

Negative CIS Elements	Negative repeat Elements
0	0

Indices based on non-uniform usage of synonymous codon				
Index	Description	Range	Value	Reference
RSCU	Relative Synonymous Codon Usage	0~6	2.17	[1]
ENC	Effective Number of Codons	20~61	32.66	[2, 3]
RCBS	Relative Codon Bias Strength	≥0	0.7	[4]
DCBS	Directional Codon Bias Score	≥1	2.53	[5]
CDC	Codon Deviation Coefficient	0~1	0.3	[6]
MILC	Measure Independent of Length and Composition	-1~1	-0.06	[7]
ICDI	Intrinsic Codon Deviation Index	0~1	0.38	[8]
SCUO	Synonymous Codon Usage Order	0~1	0.4	[9, 10]
Ew	Weighted Sum of Relative Entropy	0~1	0.58	[11]
P	Codon Preference	≥1	1.48	[12]
MCB	Maximum-likelihood Codon Bias	≥0	1.81	[13]

Indices based on codon frequency in a reference set of genes				
Index	Description	Range	Value	Reference
CAI	Codon Adaptation Index	0~1	0.92	[1]
CFD	Codon Frequency Distribution	0~1	0	[2]
FOP	Frequency of Optimal Codons	0~1	0.72	[3, 4]
COUSIN59	Codon Usage Similarity Index	∞	3.16	[5]
COUSIN18			3.71	
CBI	Codon Bias Index	-1~1	0.59	[6]
Dmean	Mean Dissimilarity-based Index	0~2	0.2	[7]

RCA	Relative Codon Adaptation	≥0	1.24	[8]
CUFS	Codon Usage Frequency Similarity	0~√2	0.37	[9]
B	Codon Usage Bias	0~2	0.61	[10]

Indices based on adaptation to the tRNA levels and their supply				
Index	Description	Range	Value	Reference
tAI	tRNA Adaptation Index	0~1	0.41	[1]
gtAI	Genetic tRNA Adaptation Index	0~1	0.46	[2]
P2	P2 Index	0~1	0.42	[3]

Indices based on complex patterns of codon usage				
Index	Description	Range	Value	Reference
GC3	GC Content at the Third Position of Synonymous Codons	0~1	0.74	[1]
GC	GC Content		0.6	
GC1	GC Content at the First Position of Synonymous Codons		0.6	
GC2	GC Content at the Second Position of Synonymous Codons		0.47	
ENcp	Effective Number of Codon Pairs	20~61	26.42	[2]
CPS	Codon Pair Score	-1~1	-0.11	[3, 4]
Codon Volatility	Codon Volatility	0.5~1	0.74	[5]
