# Supplementary material for: Reverse vaccinology-based design of multivalent multiepitope mRNA vaccines targeting key viral proteins of Herpes Simplex Virus type-2
Source: Front Immunol. 2025 May 20;16:1586271. doi: 10.3389/fimmu.2025.1586271 (PMC12130045; doi:10.3389/fimmu.2025.1586271)
Supplement: Supplementary file 1 [file DataSheet1.zip › Supplementary Data_22-04-2025/Supplementary Data 7C.pdf]

jobid=6805D1EE0015B9B1DC623ED6&wait=20 Server Output - DTU Health Tech

---

Asn-Xaa-Ser/Thr sequons in the sequence output below are highlighted in **blue**.
Asparagines predicted to be N-glycosylated are highlighted in **red**.

Output for 'construct753'

```
Name:  construct753     Length:  397
FVFLVLPLVSSQCVMAKLSTDELLDAFKEMTLLELSDFVKKFEETFEVTAAAPVAVAAAGAAPAGAAVEAAEEQSEFDVI      80
LEAAGDKKIGVIKVVREIVSGLGLKEAKDLVDGAPKPLLEKVAKEAADEAKAKLEAAGATVTVKEAAAKGIINTLQKYYC     160
RVRGGRCAVLSCLPKEEQIGKCSTRGRKCCRRKKEAAAKFIDLNITMLKKTLGLLLAYRKKRTAPRSLSLKKKEVDLDFG     240
LKKTNMVLRKRNKARYSPAAYAYRRRFPAVITRVLPAAYAVDFIWTGNQRTAPRAAYRAGRFHWERFSNASPAAYNKQST     320
RPTGACVYLEPGPGPGTMTKWQEVDEMLRAEYGPGPGGRVVFLPTIRRQLALAEAAAKAKFVAAWTLKAAAHHHHHH        400
................................................................................      80
................................................................................     160
...........................................N....................................     240
................................................................................     320
.............................................................................        400

(Threshold=0.5)
----------------------------------------------------------------------
SeqName      Position  Potential   Jury    N-Glyc
                                 agreement result
----------------------------------------------------------------------
construct753   204 NITM   0.6426     (8/9)    +
construct753   309 NASP   0.0998     (9/9)    ---
----------------------------------------------------------------------
```

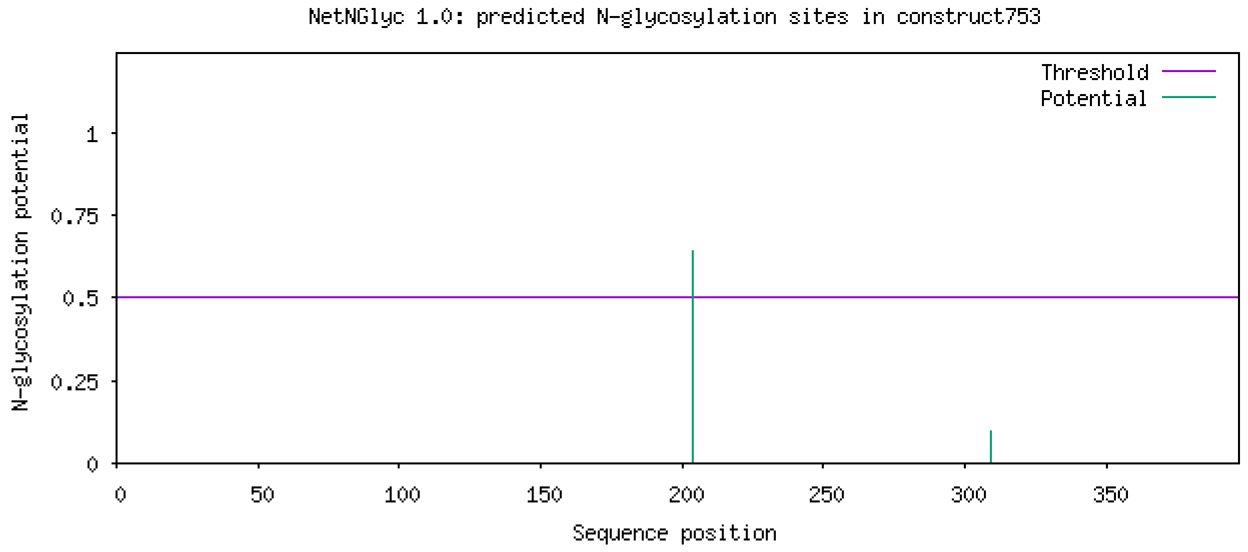

[Graphics in PostScript]

Output for 'construct2625'

```
Name:  construct2625    Length:  397
FVFLVLPLVSSQCVMAKLSTDELLDAFKEMTLLELSDFVKKFEETFEVTAAAPVAVAAAGAAPAGAAVEAAEEQSEFDVI      80
LEAAGDKKIGVIKVVREIVSGLGLKEAKDLVDGAPKPLLEKVAKEAADEAKAKLEAAGATVTVKEAAAKGIINTLQKYYC     160
RVRGGRCAVLSCLPKEEQIGKCSTRGRKCCRRKKEAAAKTLGLLLAYRKKFIDLNITMLKKKEVDLDFGLKKRTAPRSLS     240
LKKTNMVLRKRNKARYSPAAYAYRRRFPAVITRVLPAAYAVDFIWTGNQRTAPRAAYRAGRFHWERFSNASPAAYNKQST     320
RPTGACVYLEPGPGPGTMTKWQEVDEMLRAEYGPGPGGRVVFLPTIRRQLALAEAAAKAKFVAAWTLKAAAHHHHHH        400
................................................................................      80
................................................................................     160
......................................................N.........................     240
................................................................................     320
.............................................................................        400
```

(Threshold=0.5)

SeqName	Position	Potential	Jury agreement	N-Glyc result
construct2625	215 NITM	0.6417	(8/9)	+
construct2625	309 NASP	0.0998	(9/9)	---

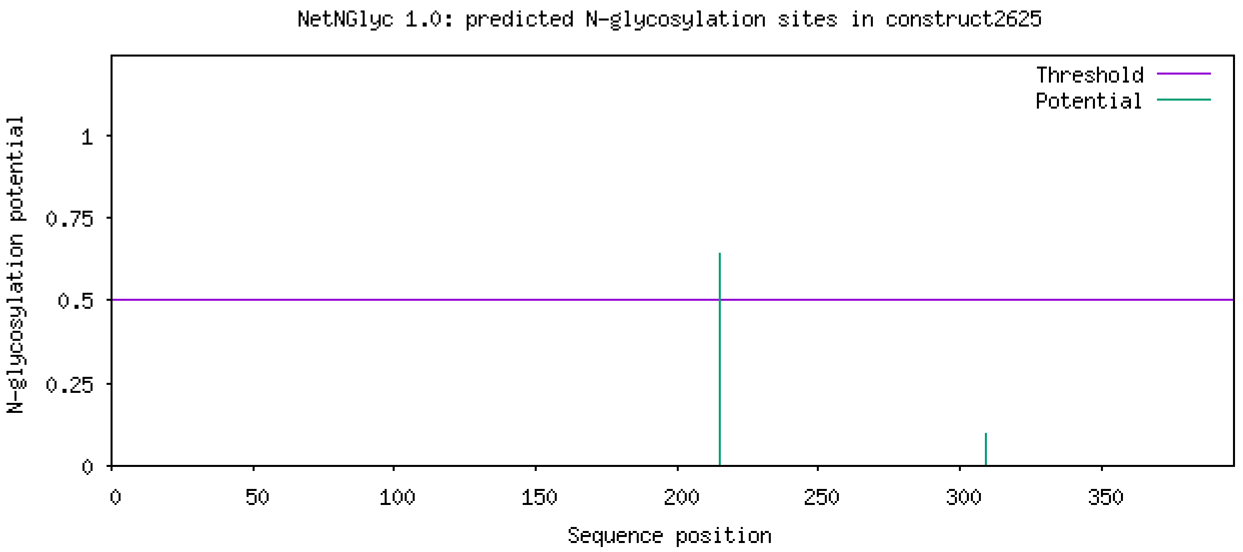

[Graphics in PostScript]

Output for 'construct735'

```
Name:  construct735     Length:  397
FVFLVLPLVSSQCVMAKLSTDELLDAFKEMTLLELSDFVKKFEETFEVTAAAPVAVAAAGAAPAGAAVEAAEEQSEFDVI      80
LEAAGDKKIGVIKVVREIVSGLGLKEAKDLVDGAPKPLLEKVAKEAADEAKAKLEAAGATVTVKEAAAKGIINTLQKYYC     160
RVRGGRCAVLSCLPKEEQIGKCSTRGRKCCRRKKEAAAKFIDLNITMLKKTLGLLLAYRKKRTAPRSLSLKKKEVDLDFG     240
LKKTNMVLRKRNKARYSPAAYAVDFIWTGNQRTAPRAAYRAGRFHWERFSNASPAAYAYRRRFPAVITRVLPAAYNKQST     320
RPTGACVYLEPGPGPGTMTKWQEVDEMLRAEYGPGPGGRVVFLPTIRRQLALAEAAAKAKFVAAWTLKAAAHHHHHH        400
................................................................................      80
................................................................................     160
...........................................N....................................     240
................................................................................     320
.............................................................................        400
```

(Threshold=0.5)

```
SeqName      Position  Potential   Jury    N-Glyc
                                 agreement result
----------------------------------------------------------------------
construct735   204 NITM    0.6426     (8/9)    +
construct735   291 NASP    0.0997     (9/9)    ---
----------------------------------------------------------------------
```

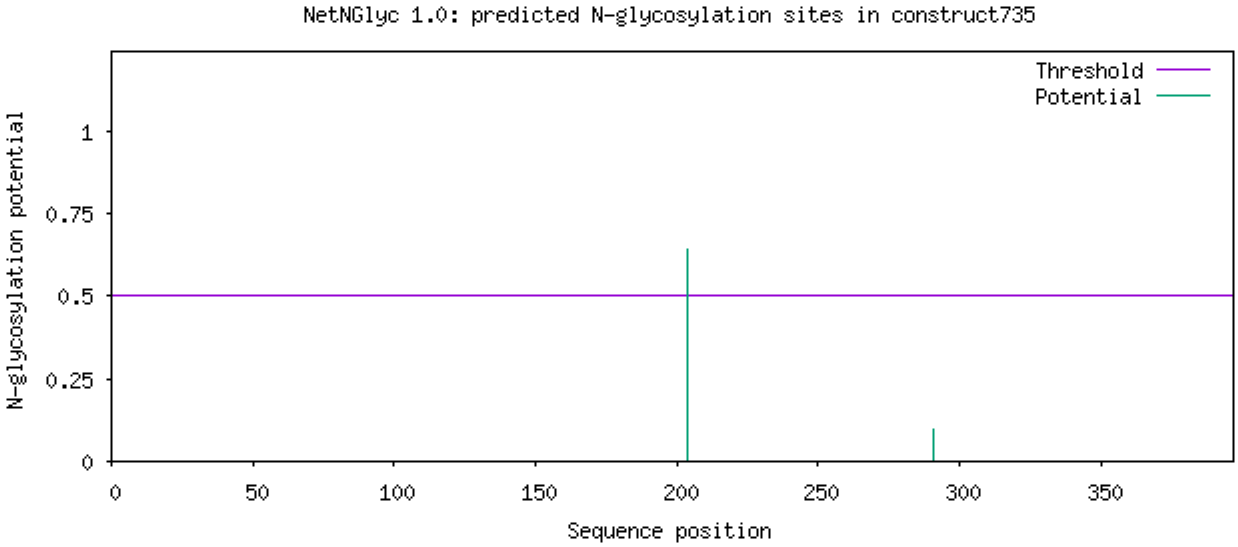

[Graphics in PostScript]

Output for 'construct2607'

```
Name:  construct2607    Length:  397
FVFLVLPLVSSQCVMAKLSTDELLDAFKEMTLLELSDFVKKFEETFEVTAAAPVAVAAAGAAPAGAAVEAAEEQSEFDVI      80
LEAAGDKKIGVIKVVREIVSGLGLKEAKDLVDGAPKPLLEKVAKEAADEAKAKLEAAGATVTVKEAAAKGIINTLQKYYC     160
RVRGGRCAVLSCLPKEEQIGKCSTRGRKCCRRKKEAAAKTLGLLLAYRKKFIDLNITMLKKKEVDLDFGLKKRTAPRSLS     240
LKKTNMVLRKRNKARYSPAAYAVDFIWTGNQRTAPRAAYRAGRFHWERFSNASPAAYAYRRRFPAVITRVLPAAYNKQST     320
RPTGACVYLEPGPGPGTMTKWQEVDEMLRAEYGPGPGGRVVFLPTIRRQLALAEAAAKAKFVAAWTLKAAAHHHHHH        400
................................................................................      80
................................................................................     160
......................................................N.........................     240
................................................................................     320
.............................................................................        400

(Threshold=0.5)
----------------------------------------------------------------------
SeqName      Position  Potential   Jury    N-Glyc
                                 agreement result
----------------------------------------------------------------------
construct2607  215 NITM    0.6417     (8/9)    +
construct2607  291 NASP    0.0997     (9/9)    ---
----------------------------------------------------------------------
```

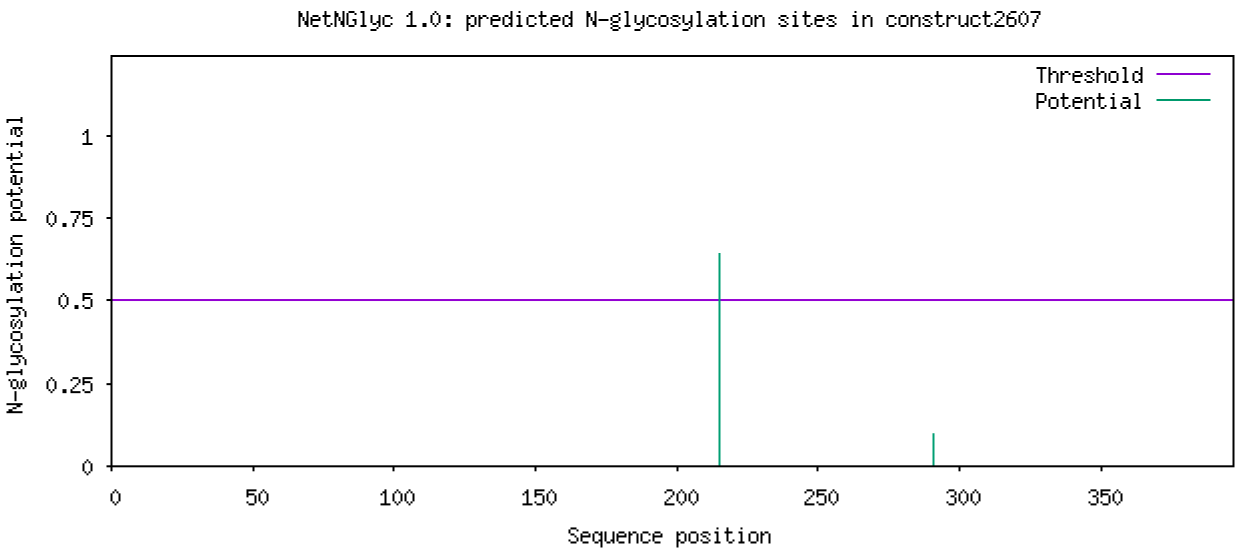

[Graphics in PostScript]

Output for 'construct2769'

```
Name:  construct2769    Length:  397
FVFLVLPLVSSQCVMAKLSTDELLDAFKEMTLLELSDFVKKFEETFEVTAAAPVAVAAAGAAPAGAAVEAAEEQSEFDVI      80
LEAAGDKKIGVIKVVREIVSGLGLKEAKDLVDGAPKPLLEKVAKEAADEAKAKLEAAGATVTVKEAAAKGIINTLQKYYC     160
RVRGGRCAVLSCLPKEEQIGKCSTRGRKCCRRKKEAAAKTLGLLLAYRKKFIDLNITMLKKRTAPRSLSLKKKEVDLDFG     240
LKKTNMVLRKRNKARYSPAAYAYRRRFPAVITRVLPAAYAVDFIWTGNQRTAPRAAYRAGRFHWERFSNASPAAYNKQST     320
RPTGACVYLEPGPGPGTMTKWQEVDEMLRAEYGPGPGGRVVFLPTIRRQLALAEAAAKAKFVAAWTLKAAAHHHHHH        400
................................................................................      80
................................................................................     160
......................................................N.........................     240
................................................................................     320
.............................................................................        400

(Threshold=0.5)
----------------------------------------------------------------------
SeqName      Position  Potential   Jury    N-Glyc
                                agreement  result
----------------------------------------------------------------------
construct2769  215 NITM   0.6438     (8/9)   +
construct2769  309 NASP   0.0998     (9/9)   ---
----------------------------------------------------------------------
```

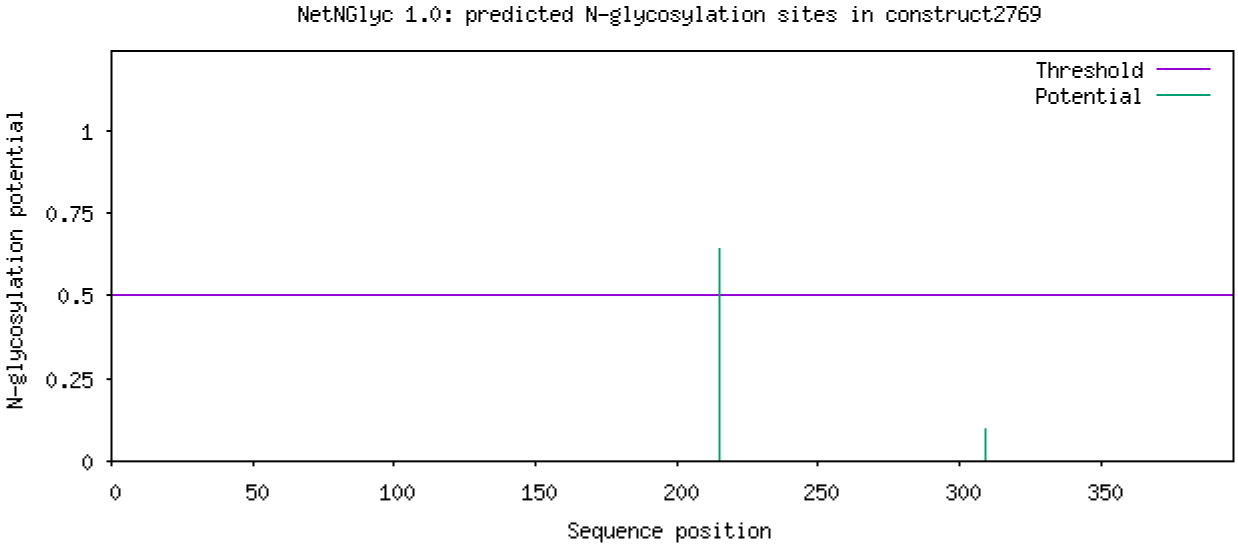

[Graphics in PostScript]

Go [back].
