# Supplementary material for: Reverse vaccinology-based design of multivalent multiepitope mRNA vaccines targeting key viral proteins of Herpes Simplex Virus type-2
Source: Front Immunol. 2025 May 20;16:1586271. doi: 10.3389/fimmu.2025.1586271 (PMC12130045; doi:10.3389/fimmu.2025.1586271)
Supplement: Supplementary file 1 [file DataSheet1.zip › Supplementary Tables_22-04-2025/Supplementary_Material - Tables.docx]

Supplementary Tables

**Supplementary Table 1:**  Protein sequence ID retrieved from NCBI and UniProt across HG52, SD90e and S333 strains of HSV-2 along with sequence length and molecular weight.

| **Strain** | **Protein name** | **Accession ID** | **Sequence length (amino acids)** | **Molecular Weight (kDa)** |
| --- | --- | --- | --- | --- |
| HG52 | gB | P08666 | 904 | 100.217 |
|  | RIR1 | AEV91378.1 | 1142 | 124.923 |
|  | ICP0 | AEV91338.2 | 834 | 82.669 |
|  | VP23 | AEV91356.1 | 318 | 34.343 |
| SD90e | gB | AHG54691.1 | 901 | 99.976 |
|  | RIR1 | AHG54703.1 | 1141 | 124.785 |
|  | ICP0 | AHG54722.1 | 806 | 80.268 |
|  | VP23 | AHG54681.1 | 319 | 34.343 |
| S333 | gB | P06763 | 904 | 100.186 |
|  | RIR1 | AKC42803.1 | 1144 | 125.093 |
|  | ICP0 | SPT06165.1 | 814 | 80.99 |
|  | VP23 | AKC42781.1 | 319 | 34.343 |

**Supplementary Table 2:** comparison between secondary structure predictions of top 5 constructs done in PSIPRED and SOPMA.

| **Vaccine constructs** | **PSIPRED** | | | **SOPMA** | | |
| --- | --- | --- | --- | --- | --- | --- |
|  | **Alpha Helix (%)** | **Strand (%)** | **Coil (%)** | **Alpha Helix (%)** | **Strand (%)** | **Coil (%)** |
| **C1_753** | 53.02 | 11.65 | 35.43 | 52.76 | 9.80 | 37.44 |
| **C2_2625** | 57.76 | 12.06 | 36.18 | 51.26 | 11.31 | 37.44 |
| **C3_735** | 51.51 | 12.06 | 36.43 | 54.27 | 9.80 | 35.93 |
| **C4_2607** | 51.51 | 12.06 | 36.43% | 52.76 | 11.31 | 35.93 |
| **C5_2769** | 52.51 | 11.81 | 35.68% | 50.50 | 11.81 | 37.69 |

**Supplementary Table 3:** Summarizes the codon optimisation analysis of all the five vaccine constructs.

| **Vaccine constructs** | **Codon Adapatation Index (CAI)** | **tRNA Adaptation Index (tAI)** | **Effective Number of Codons (ENC)** | **GC content** |
| --- | --- | --- | --- | --- |
| C1_753 | 0.93 | 0.41 | 31.83 | 60.75% |
| C2_2625 | 0.93 | 0.41 | 31.84 | 60.75% |
| C3_735 | 0.92 | 0.41 | 32.24 | 60.92% |
| C4_2607 | 0.92 | 0.41 | 31.83 | 60.40% |
| C5_2769 | 0.92 | 0.41 | 32.66 | 60.40% |
