# Supplementary figures and images for: Reverse vaccinology-based design of multivalent multiepitope mRNA vaccines targeting key viral proteins of Herpes Simplex Virus type-2
Source: Front Immunol. 2025 May 20;16:1586271. doi: 10.3389/fimmu.2025.1586271 (PMC12130045; doi:10.3389/fimmu.2025.1586271)

PSIPRED 2.0 Secondary Structure Analysis.

C735

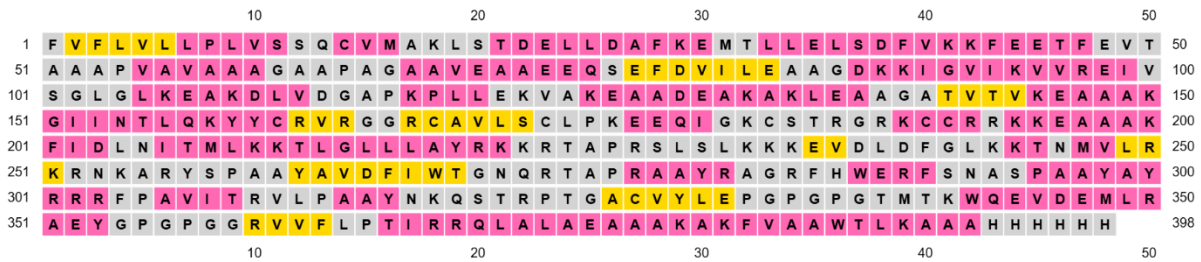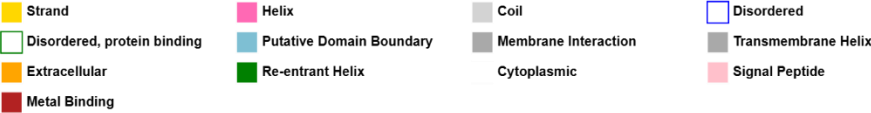

C753

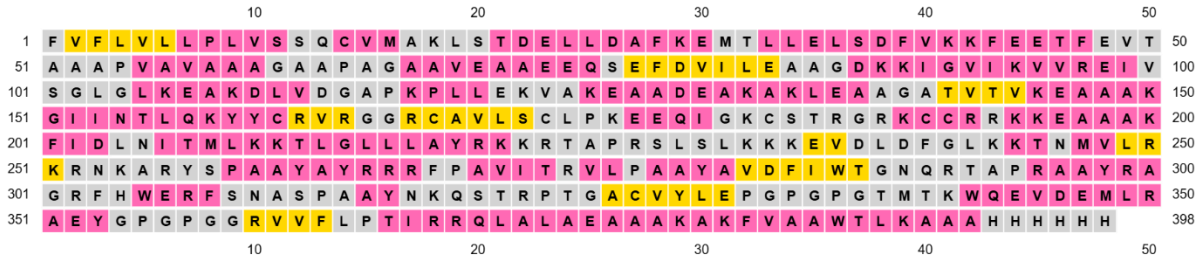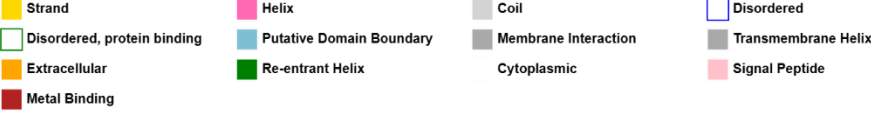

C2607

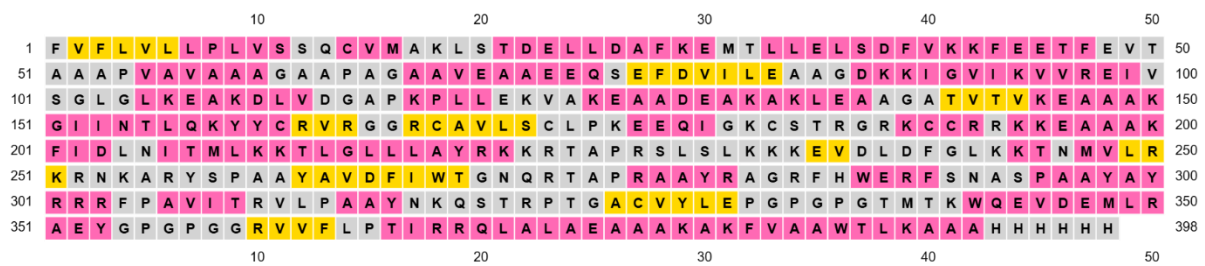

C2769

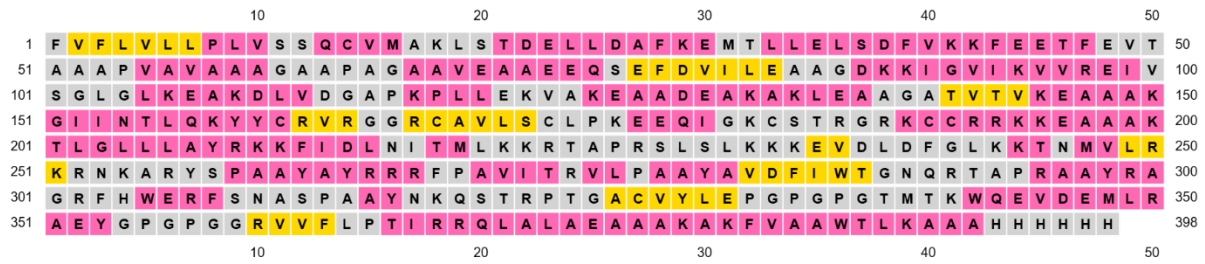

Supplement: Supplementary file 1 [file DataSheet1.zip › Supplementary Data_22-04-2025/Supplementary Data 4A.pdf]
